# Supplementary material for: Public interest in biodiversity and climate change: A comparative culturomics study of China and the UK
Source: PLoS One. 2026 Jan 14;21(1):e0338006. doi: 10.1371/journal.pone.0338006 (PMC12803463; doi:10.1371/journal.pone.0338006)
Supplement: S1 Text — (DOCX) [file pone.0338006.s001.docx]

**Supplementary Information**

**Contents**

Supplementary text

**Text S1:** Website address of Chinese biodiversity news texts (Search volume peaks)

**Text S2:** Website address of Chinese biodiversity news texts (Regular periods)

**Text S3:** Website address of British biodiversity news texts (Search volume peaks)

**Text S4:** Website address of British biodiversity news texts (Regular periods)

**Text S5:** Website address of Chinese climate change news texts (Search volume peaks)

**Text S6:** Website address of Chinese climate change news texts (Regular periods)

**Text S7:** Website address of British climate change news texts (Search volume peaks)

**Text S8:** Website address of British climate change news texts (Regular periods)

**Text S9:** Chinese local government news

Supplementary tables

**Table S1:** Count of analysed time periods of peak and regular time in China and the UK

**Table S2:** Total text words, primary codes categories and codes numbers of peak and regular time in China and the UK

**Table S3:** Code system of biodiversity

**Table S4:** Code system of climate change

**SUPPLEMENTARY TEXT**

Some news links were no longer accessible and have been removed. Please refer directly to the excerpted news texts in Supporting Information 2 and 3. Notably, certain Chinese news websites may be inaccessible outside mainland China due to regional access restrictions and may require a China-based IP address.

**Text S1:** Website address of Chinese biodiversity news texts (Search volume peaks)

1. 2018/04/16-2018/04/22

<https://weibo.com/ttarticle/p/show?id=2309404231577981848519>

<http://www.hinews.cn/news/system/2018/04/18/031442118.shtml>

<https://www.jfdaily.com/news/detail?id=86857>

<https://news.sciencenet.cn/htmlnews/2018/4/409167.shtm>

(Require a China-based IP address)

<https://www.dy001.cn/2018/0419/39557.shtml>

<https://weibo.com/ttarticle/p/show?id=2309404229371115249872>

<https://mp.weixin.qq.com/s?__biz=MjM5MDg5NDMzMQ==&mid=2650069391&idx=1&sn=82ffed3b86275011e1d1358a7bb035c3&chksm=bebda81b89ca210dc7e369eb7ef4a6f88268e8abe1d03ceff3293c0f0c70a0086dd2038c9983&scene=27>

<http://www.360doc.com/content/18/0420/23/40252042_747415232.shtml>

<http://k.sina.com.cn/article_6459704664_181074558001005mkr.html>

1. 2019/05/27-2019/06/02

<https://wxd.sznews.com/BaiDuBaiJia/20190601/content_325038.html>

<https://baijiahao.baidu.com/s?id=1634693190014346648&wfr=spider&for=pc>

<https://www.thepaper.cn/newsDetail_forward_3540903>

<https://www.cqcb.com/county/qianjiang/qianjiangxinwen/2019-05-27/1645215.html>

(Require a China-based IP address)

<https://www.sohu.com/a/316870570_120029441>

<https://www.etycx.com/news_article/pid-2676.html>

1. 2020/05/18-2020/05/24

<https://baijiahao.baidu.com/s?id=1667410202010864496&wfr=spider&for=pc>

<http://news.cctv.com/2020/05/22/ARTIvIrDGgb4oLnnxUryHBae200522.shtml>

<https://baijiahao.baidu.com/s?id=1667378864904108274&wfr=spider&for=pc>

<https://baijiahao.baidu.com/s?id=1667367674240547763&wfr=spider&for=pc>

<https://baijiahao.baidu.com/s?id=1667440883492765141&wfr=spider&for=pc>

<https://baijiahao.baidu.com/s?id=1667218369339449741&wfr=spider&for=pc>

<https://www.thepaper.cn/newsDetail_forward_7524613>

<https://new.qq.com/rain/a/20200521A0I6UQ00>

1. 2021/05/17-2021/05/23

<http://news.cctv.com/2021/05/22/ARTILjKRvW94CGCeIfu7fDa1210522.shtml>

<https://baijiahao.baidu.com/s?id=1700428075675260498&wfr=spider&for=pc>

<http://news.cctv.com/2021/05/22/ARTIhaSbyRtbrtj8OefIUcIw210522.shtml>

<https://sghexport.shobserver.com/html/baijiahao/2021/05/22/440708.html>

<http://k.sina.com.cn/article_6824573189_196c6b905020013n49.html>

<https://baijiahao.baidu.com/s?id=1700458450037402005&wfr=spider&for=pc>

<https://baijiahao.baidu.com/s?id=1700443874977614574&wfr=spider&for=pc>

<https://baijiahao.baidu.com/s?id=1700411483987963956&wfr=spider&for=pc>

1. 2021/10/11-2021/10/17

<https://baijiahao.baidu.com/s?id=1713697904404347598&wfr=spider&for=pc>

<https://www.mee.gov.cn/ywdt/zbft/202110/t20211016_956781.shtml>

<https://baijiahao.baidu.com/s?id=1713522571299231670&wfr=spider&for=pc>

<https://baijiahao.baidu.com/s?id=1713704259143847485&wfr=spider&for=pc>

<https://baijiahao.baidu.com/s?id=1713748306593016816&wfr=spider&for=pc>

<https://baijiahao.baidu.com/s?id=1713340687074856283&wfr=spider&for=pc>

<https://baijiahao.baidu.com/s?id=1713411488019875631&wfr=spider&for=pc>

<https://baijiahao.baidu.com/s?id=1713662990360423141&wfr=spider&for=pc>

<https://baijiahao.baidu.com/s?id=1713394021693218230&wfr=spider&for=pc>

<https://baijiahao.baidu.com/s?id=1713298564116049968&wfr=spider&for=pc>

1. 2022/05/16-2022/05/22

<https://baijiahao.baidu.com/s?id=1733344725190182150&wfr=spider&for=pc>

<https://www.spp.gov.cn/spp/zdgz/202205/t20220522_557493.shtml>

<https://m.gmw.cn/baijia/2022-05/21/35753106.html>

<https://www.xiancn.com/content/2022-05/21/content_6560225.htm>

(Require a China-based IP address)

<https://baijiahao.baidu.com/s?id=1733416134251094772&wfr=spider&for=pc>

<https://m.thepaper.cn/baijiahao_18210991>

<https://baijiahao.baidu.com/s?id=1733312382893416356&wfr=spider&for=pc>

<https://sthjt.yn.gov.cn/ywdt/xxywrdjj/202205/t20220520_230014_wap.html>

**Text S2:** Website address of Chinese biodiversity news texts (Regular periods)

1. 2011.1.1-2013.12.31

<https://news.sina.com.cn/o/2013-05-23/110727204283.shtml>

<https://news.sina.com.cn/o/2013-11-01/004128587170.shtml>

<http://world.people.com.cn/n/2013/0717/c1002-22231376.html>

<https://www.163.com/news/article/8M2S1KQV000125LI.html>

<https://www.cas.cn/xw/cmsm/201212/t20121219_3724231.shtml>

<https://news.ifeng.com/c/7fZetgvW6A1>

<http://news.cntv.cn/20120523/107303.shtml>

<https://heilongjiang.dbw.cn/system/2012/09/17/054240565.shtml>

(Require a China-based IP address)

1. 2014.1.1-2016.12.31

<http://news.cctv.com/2016/12/18/ARTITV6oweSzF6qAlxLk8fxz161218.shtml>

<https://shuangyashan.dbw.cn/system/2016/12/19/057481690.shtml>

<http://news.cctv.com/2016/05/22/ARTIFDgkqDv58MW0fVgzsGBY160522.shtml>

<http://www.xinhuanet.com/world/2016-12/18/c_1120139140.htm>

<https://www.jiemian.com/article/459789.html>

<https://news.sina.com.cn/green/2015-12-02/doc-ifxmaznc5867631.shtml>

<https://news.sciencenet.cn/htmlnews/2015/5/319394.shtm>

<https://finance.sina.com.cn/roll/20150524/055922252126.shtml>

<https://3g.163.com/travel/article/C169SEK500064M1B.html>

1. 2017.1.1-2019.12.31

<http://news.cctv.com/2019/09/16/ARTIMKu6m2CiDe88hdz1yL16190916.shtml>

<https://baijiahao.baidu.com/s?id=1642392917017878431&wfr=spider&for=pc>

<http://news.cctv.com/2019/09/05/ARTIfVO8pu8xYW51DffMlyBt190905.shtml>

<https://baijiahao.baidu.com/s?id=1643842473310644880&wfr=spider&for=pc>

<https://baijiahao.baidu.com/s?id=1650084987689597389&wfr=spider&for=pc>

<https://baijiahao.baidu.com/s?id=1629398150970793350&wfr=spider&for=pc>

<https://baijiahao.baidu.com/s?id=1647811138074388537&wfr=spider&for=pc>

<https://baijiahao.baidu.com/s?id=1650549286505490746&wfr=spider&for=pc>

<http://finance.sina.com.cn/roll/2019-05-07/doc-ihvhiqax7167038.shtml>

1. 2020.1.1-2022.11.1

<http://k.sina.com.cn/article_3164957712_bca56c1002001oy9f.html>

<https://baijiahao.baidu.com/s?id=1709865763398080778&wfr=spider&for=pc>

<https://new.qq.com/rain/a/20220817A0BERC00>

<https://weibo.com/ttarticle/p/show?id=2309404598744653103621&sudaref=www.baidu.com&display=0&retcode=6102>

<http://k.sina.com.cn/article_3164957712_bca56c1002001oy9f.html>

**Text S3:** Website address of British biodiversity news texts (Search volume peaks)

1. 2011.3

<https://theecologist.org/2011/mar/23/why-invasive-plants-are-second-biggest-threat-biodiversity-after-habitat-loss>

<https://www.bbc.co.uk/blogs/natureuk/2011/03/how-not-to-make-your-garden-wi.shtml>

<https://www.bbc.co.uk/blogs/thereporters/richardblack/2011/03/the_united_nations_environment.html>

<https://www.bbc.com/news/uk-northern-ireland-12894548>

<https://www.bbc.co.uk/news/science-environment-12718251>

<https://theecologist.org/2011/mar/11/hen-and-hammock-changing-world-one-garden-time>

<https://theecologist.org/2011/mar/25/palm-oil-giants-target-africa-land-grab-following-indonesia-deforestation-ban>

<https://theecologist.org/2011/mar/10/study-spiders-shows-species-may-be-able-adapt-global-warming>

<https://theecologist.org/2011/mar/22/super-nature-creating-wildlife-garden>

<https://theecologist.org/2011/mar/29/europe-moves-ban-imports-tar-sands-oil-canada>

2. 2020.6

<https://www.bbc.co.uk/news/science-environment-53008292>

<https://www.shoosmiths.co.uk/insights/articles/biodiversity-net-gain>

<https://www.unep-wcmc.org/en/news/drought--desertification-and-drylands-biodiversity>

<https://www.unep-wcmc.org/en/news/colombia-highlights-the-importance-of-biocultural-diversity>

<https://theedinburghreporter.co.uk/2020/06/how-forest-loss-has-changed-biodiversity-over-the-last-150-years/>

<https://www.digitalcameraworld.com/news/canon-teaches-young-people-wildlife-photography-to-help-preserve-biodiversity>

<https://www.gov.uk/government/news/bird-survey-shows-thriving-ecosystem-at-coal-authority-sites>

3. 2021.2

<https://www.varsity.co.uk/science/20795>

<https://www.traffic.org/news/post-2020-global-biodiversity-framework-must-focus-on-sustainable-use-strong-indicators-and-the-links-with-human-health/>

<https://www.etfstrategy.com/ossiam-launches-food-for-biodiversity-etf-focused-on-habitat-preservation-xetra-f4de-f4du-ucits-49490/>

<https://www.unep-wcmc.org/en/news/three-more-countries-start-national-ecosystem-assessments>

<https://www.nhm.ac.uk/discover/news/2021/february/we-need-to-act-now-to-save-nature.html>

<https://environment-analyst.com/global/106629/un-calls-for-end-to-war-on-nature>

<https://www.unep-wcmc.org/en/news/successful-protected-areas-are-a-matter-of-quality--not-just-quantity>

<https://www.thecanary.co/discovery/analysis-discovery/2021/02/18/explosive-study-shows-the-wildlife-trade-plays-a-major-role-in-the-rapid-loss-of-the-worlds-species/>

4. 2022.3

<https://www.bbc.co.uk/news/science-environment-60737448>

<https://www.traffic.org/news/wildlife-trade-and-the-post-2020-global-biodiversity-framework/>

<https://www.weforum.org/agenda/2022/03/high-tech-maps-biodiversity/>

<https://news.hackney.gov.uk/23m-secured-to-improve-facilities-walking-routes-and-biodiversity-at-west-reservoir/>

<https://www.britishecologicalsociety.org/biodiversity-loss-has-knock-on-effects-on-global-markets/>

<https://www.weforum.org/agenda/2022/03/wetlands-climate-change/>

<https://www.ecotricity.co.uk/our-news/2022/ecotricity-explains-boosting-biodiversity-at-our-green-gas-mills>

<https://www.britishecologicalsociety.org/research-demonstrates-value-injurious-weeds-can-bring-to-both-pollinators-and-biodiversity/>

<https://www.theactuary.com/news/2022/03/15/biodiversity-loss-become-material-risk-global-businesses-2024>

<https://www.cbi.org.uk/articles/putting-biodiversity-at-the-heart-of-business-agenda-2/>

5. 2022.5

<https://www.wildlifetrusts.org/news/national-highways-and-wildlife-trusts-announce-biodiversity-boost-across-england>

<https://www.gov.uk/government/news/50-projects-receive-up-to-100000-each-to-boost-investment-in-nature>

<https://www.bbc.com/news/uk-england-norfolk-61425244>

<https://research.senedd.wales/research-articles/cop15-time-to-tackle-the-nature-emergency/>

<https://labourlist.org/2022/05/we-need-decisive-action-on-biodiversity-loss-and-the-uk-must-lead-the-way/>

<https://www.thegrocer.co.uk/cop27/why-birds-eye-is-choosing-now-to-mount-a-biodiversity-campaign/667703.article>

<https://www.unep-wcmc.org/en/news/securing-a-shared-future-for-all-life-on-earth>

<https://www.pbctoday.co.uk/news/digital-construction/bim-news/national-tree-map-helps-improve-biodiversity-in-london/111377/>

<https://www.gov.uk/government/news/new-funding-to-protect-biodiversity-in-uk-overseas-territories>

**Text S4:** Website address of British biodiversity news texts (Regular periods)

1. 2011.1.1-2013.12.31

<https://www.bbc.co.uk/news/world-latin-america-13186823>

<https://theecologist.org/2012/mar/20/why-bees-biodiversity-benefit-indigenous-wildflowers>

<https://theecologist.org/2012/sep/17/get-grip-population-growth-impacts-biodiversity>

<https://theecologist.org/2012/mar/09/oil-deal-threatens-ugandan-biodiversity>

<https://www.bbc.com/news/science-environment-25186871>

<https://www.bbc.com/news/world-asia-india-19947269>

<https://www.bbc.com/news/science-environment-14912813>

<https://www.bristol.ac.uk/news/2013/9064.html>

<https://theecologist.org/2011/apr/13/environmental-damage-and-human-rights-abuses-blight-global-tea-sector>

1. 2014.1.1-2016.12.31

<https://www.bbc.com/news/science-environment-26140827>

<https://www.bbc.com/news/science-environment-32781136>

<https://www.bbc.com/news/science-environment-36805227>

<https://www.unep-wcmc.org/en/news/the-a-z-of-biodiversity>

<https://www.bbc.com/news/science-environment-36035968>

<https://www.bbc.com/news/science-environment-25827837>

<https://www.nhm.ac.uk/discover/news/2016/july/biodiversity-breaching-safe-limits-worldwide.html>

<https://theecologist.org/2015/dec/28/bullfighting-conserving-spains-biodiversity-ban-natures-peril>

<https://www.theparliamentmagazine.eu/news/article/worlds-poor-most-affected-by-biodiversity-and-ecosystem-loss>

<https://www.unep-wcmc.org/en/news/predicting-the-impact-of-land-use-change-on-biodiversity>

1. 2017.1.1-2019.12.31

<https://www.nhm.ac.uk/discover/news/2019/may/one-million-animals-and-plants-face-extinction.html>

<https://www.weforum.org/agenda/2019/05/biodiversity-loss-pollution-climate-change-report/>

<https://www.weforum.org/agenda/2019/02/future-of-food-under-severe-threat-as-species-diversity-disappears-un>

<https://www.gov.uk/government/news/government-sets-out-vision-for-a-safe-and-biodiverse-railway-lineside>

<https://www.openaccessgovernment.org/biodiversity-in-uk/64782/>

<https://www.countrylife.co.uk/news/state-nature-britains-biodiversity-threat-can-yet-saved-205064>

<https://www.bbc.com/news/science-environment-46028862>

<https://www.gov.uk/guidance/how-to-benefit-species-and-habitats-biodiversity-in-your-woodland>

<https://www.unep-wcmc.org/en/news/new-global-forest-watch-biodiversity-maps-help-prioritise-areas-for-conservation>

<https://www.unep-wcmc.org/en/news/biodiversity-indicators-partnership-launches-a-new-platform-for-visualising-biodiversity-indicators>

1. 2020.1.1-2022.11.1

<https://www.gov.uk/government/news/pm-commits-to-protect-30-of-uk-land-in-boost-for-biodiversity>

<https://theecologist.org/2021/oct/11/britain-faces-biodiversity-collapse>

<https://www.gov.uk/government/news/uk-takes-lead-to-seek-global-action-on-nature-at-cop15-biodiversity-conference>

<https://www.nhm.ac.uk/discover/news/2022/june/cop15-explained.html>

<https://www.nhm.ac.uk/discover/news/2022/october/wildlife-populations-crashed-by-69-within-less-than-a-lifetime.html>

<https://www.nhm.ac.uk/discover/news/2022/november/destruction-forests-and-grasslands-biggest-cause-of-biodiversity-loss.html>

<https://www.gov.uk/government/speeches/weve-overexploited-the-planet-now-we-need-to-change-if-were-to-survive>

<https://www.weforum.org/agenda/2022/07/biodiversity-ecosystem-credit-debt/>

<https://www.omfif.org/2021/01/time-to-align-finance-with-biodiversity-objectives/>

**Text S5:** Website address of Chinese climate change news texts (Search volume peaks)

1. 2015.11.30-2015.12.6

<https://www.thepaper.cn/newsDetail_forward_1404500>

<http://news.cntv.cn/2015/12/02/ARTI1449018949491658.shtml>

<http://news.cntv.cn/2015/12/05/ARTI1449293549257280.shtml>

<https://news.sina.com.cn/o/2015-12-01/doc-ifxmainy1510844.shtml>

<http://news.hsw.cn/system/2015/1201/332011.shtml>

<https://www.163.com/news/article/B9M5AAEC00014AED.html>

<http://www.xinhuanet.com/world/2015-12/02/c_128491135.htm>

<http://finance.sina.com.cn/roll/20151202/153723912732.shtml?qq-pf-to=pcqq.c2c>

<https://m.jiemian.com/article/455378.html>

<http://www.p5w.net/news/gncj/201512/t20151201_1279392.htm>

(Require a China-based IP address)

1. 2017.12.4-2017.12.10

<http://www.hinews.cn/news/system/2017/12/06/031348544.shtml>

<http://www.hinews.cn/news/system/2017/12/09/031352068.shtml>

<http://www.china.com.cn/news/world/2017-12/05/content_41967099.htm>

<http://k.sina.com.cn/article_6403268733_17daa207d001003r9m.html>

<http://www.chinanews.com.cn/gj/2017/12-06/8393872.shtml>

<http://www.cma.gov.cn/2011xwzx/2011xqxxw/2011xqxyw/201712/t20171205_457476.html?from=singlemessage>

1. 2018.2.5-2018.2.11

<http://k.sina.com.cn/article_6450855291_180803d7b001003hr9.html?from=science>

<https://baijiahao.baidu.com/s?id=1591618387613820687&wfr=spider&for=pc>

<https://qitihui.com/archives/142562.html>

(Require a China-based IP address)

<https://www.antpedia.com/news/60/n-1462560.html>

<https://wiki.antpedia.com/article-402978>

<https://news.sina.com.cn/o/2018-02-07/doc-ifyreuzn4268763.shtml>

<https://www.tianqi.com/news/214047.html>

(Require a China-based IP address)

<http://mt.sohu.com/20180208/n530481354.shtml>

1. 2021.11.15-2021.11.21

<https://baijiahao.baidu.com/s?id=1716758321471418688&wfr=spider&for=pc>

<https://baijiahao.baidu.com/s?id=1716486654887319437&wfr=spider&for=pc>

<http://k.sina.com.cn/article_1784473157_6a5ce64502002cp7p.html>

<http://news.hexun.com/2021-11-18/204772177.html>

<https://weibo.com/ttarticle/p/show?id=2309404705905672585785>

<https://card.weibo.com/article/m/show/id/2309404705261431685385>

<https://news.china.com/internationalzq/13004216/20211116/40287338_1.html>

1. 2022.2.7-2022.2.14

<http://k.sina.com.cn/article_7517400647_1c0126e4705902qmhn.html>

<https://baijiahao.baidu.com/s?id=1724458887040675098&wfr=spider&for=pc>

<https://www.thepaper.cn/newsDetail_forward_16597729>

<https://zhidao.baidu.com/daily/view?id=252453>

<http://www.northnews.cn/news/8710/0211/2067898.html>

(Require a China-based IP address)

<https://www.sohu.com/a/522155393_488177>

**Text S6:** Website address of Chinese climate change news texts (Regular periods)

1. 2011.1.1-2013.12.31

<http://www.cma.gov.cn/2011xwzx/2011xqxxw/2011xqxyw/201202/t20120224_162468.html>

<https://news.sina.com.cn/o/2012-08-21/165825008490.shtml>

<http://green.sina.com.cn/news/roll/2012-06-05/103124538330.shtml>

<http://inews.nmgnews.com.cn/system/2012/04/23/010756438_01.shtml>

<https://news.sina.com.cn/o/2012-07-12/111424761432.shtml>

<https://www.chinanews.com/ny/2012/02-24/3695864.shtml>

<http://star.news.sohu.com/20120802/n349630699.shtml>

1. 2014.1.1-2016.12.31

<http://www.gov.cn/xinwen/2016-11/02/content_5127313.htm>

<http://news.cctv.com/2016/03/10/ARTIw5vrXPyentoPaEQtFzYv160310.shtml>

<http://news.cctv.com/2016/08/30/ARTIreWjrqi9CIz09B5EMSYy160830.shtml?ivk_sa=1023197a>

<http://www.xinhuanet.com/world/2016-11/25/c_129378736.htm>

<http://www.cma.gov.cn/2011xzt/2016zt/20161102/2015112704/201611/t20161110_341531.html>

<http://www.tanjiaoyi.com/article-11870-2.html>

<https://www.163.com/news/article/ALBRH4CQ00014Q4P.html>

<https://news.sina.com.cn/o/2016-03-07/doc-ifxqafrm7133549.shtml>

1. 2017.1.1-2019.12.31

<https://www.thepaper.cn/newsDetail_forward_5086690>

<https://baijiahao.baidu.com/s?id=1654222609019028220&wfr=spider&for=pc>

<https://page.om.qq.com/page/Oj85ym2VOTYH8SWn2VpQAQ2Q0>

<http://www.gov.cn/xinwen/2019-11/27/content_5456146.htm>

<https://news.sciencenet.cn/htmlnews/2019/12/434328.shtm>

<https://www.jfdaily.com/news/detail.do?id=192902>

<http://news.cctv.com/2019/07/29/ARTI0PF5PXzzZ3GlK3zuyK2Q190729.shtml>

<https://baijiahao.baidu.com/s?id=1605323892021008423&wfr=spider&for=pc>

<https://www.douban.com/note/728220127/?_i=783902753uoXLO>

1. 2020.1.1-20222.11.1

<https://baijiahao.baidu.com/s?id=1748196372683233465&wfr=spider&for=pc>

<https://baijiahao.baidu.com/s?id=1747841937149640149&wfr=spider&for=pc>

<https://new.qq.com/rain/a/20221026A06PLD00>

<https://news.sina.com.cn/o/2022-10-27/doc-imqqsmrp3956777.shtml>

<https://sdxw.iqilu.com/share/YS0yMS0xMjMwODkxNQ.html>

<https://baijiahao.baidu.com/s?id=1745089121693499169&wfr=spider&for=pc>

<https://baijiahao.baidu.com/s?id=1746560094578179569&wfr=spider&for=pc>

<https://baijiahao.baidu.com/s?id=1741180006283777182&wfr=spider&for=pc>

<https://baijiahao.baidu.com/s?id=1739023829394936065&wfr=spider&for=pc>

<https://www.jfdaily.com/news/detail?id=523095>

**Text S7:** Website address of British climate change news texts (Search volume peaks)

1. 2019.4

<https://www.mirror.co.uk/3am/celebrity-news/emma-thompson-joins-climate-change-14431549>

[Thousands of climate change protesters descend on London | Metro News](https://metro.co.uk/2019/04/15/thousands-climate-change-protestors-descend-london-shut-capital-9203391/)

<https://www.cityam.com/climate-change-protests-122-arrests-extinction-rebellions/>

<https://www.dailyrecord.co.uk/news/scottish-news/climate-change-activists-climb-glasgows-14315549>

<https://www.standard.co.uk/news/uk/scores-of-london-students-descend-on-parliament-square-for-climate-change-strike-a4116721.html>

<https://www.vogue.co.uk/article/greta-thunberg-london-speech-climate-change-protests>

<https://socialistworker.co.uk/news/extinction-rebellion-has-shifted-politics-on-the-climate/>

1. 2019.9

<https://inews.co.uk/news/environment/climate-change-summit-un-2019-new-york-global-action-342540>

<https://www.refinery29.com/en-gb/2019/09/8469745/how-to-help-climate-change-greta-thunberg>

<https://www.bbc.co.uk/news/science-environment-49817804>

<https://www.wwf.org.uk/updates/communities-coping-climate-change>

<https://www.nme.com/news/the-simpsons-predicted-greta-thunbergs-climate-change-speech-in-2007-2551972>

<https://www.nme.com/features/greta-thunberg-full-speech-to-the-un-2019-climate-change-summit-2550824>

<https://www.greenpeace.org.uk/news/top-3-things-you-need-to-know-about-the-climate-strike-on-20-september/>

<https://www.bbc.com/news/uk-england-49593380>

1. 2021.11

<https://www.bbc.co.uk/news/science-environment-59308958>

<https://www.bcs.org/articles-opinion-and-research/the-tech-to-fight-climate-change-is-already-here-now-it-needs-urgent-investment-and-support/>

<https://www.scotsman.com/news/environment/cop26-climate-change-will-see-320-million-people-worldwide-facing-starvation-this-decade-report-warns-3449974>

<https://www.bbc.com/news/science-environment-59220687>

<https://www.theweek.co.uk/news/environment/954661/climate-change-the-worst-offenders>

<https://www.scotsman.com/news/environment/what-is-the-15-degrees-climate-change-pledge-what-happens-if-we-pass-15-degrees-of-global-warming-3455958>

<https://www.stylist.co.uk/news/cop26-glasgow-climate-change-conference-key-moments/582952>

<https://www.bbc.co.uk/news/business-59136214>

1. 2021.11

<https://www.bbc.co.uk/news/science-environment-59308958>

<https://www.bcs.org/articles-opinion-and-research/the-tech-to-fight-climate-change-is-already-here-now-it-needs-urgent-investment-and-support/>

<https://www.scotsman.com/news/environment/cop26-climate-change-will-see-320-million-people-worldwide-facing-starvation-this-decade-report-warns-3449974>

<https://www.bbc.com/news/science-environment-59220687>

<https://www.theweek.co.uk/news/environment/954661/climate-change-the-worst-offenders>

<https://www.scotsman.com/news/environment/what-is-the-15-degrees-climate-change-pledge-what-happens-if-we-pass-15-degrees-of-global-warming-3455958>

<https://www.stylist.co.uk/news/cop26-glasgow-climate-change-conference-key-moments/582952>

<https://www.bbc.co.uk/news/business-59136214>

**Text S8:** Website address of British climate change news texts (Regular periods)

1. 2011.1.1-2013.12.31

[IPCC climate change report: Humans are causing global warming but we STILL can't explain why Earth's barely got any hotter in the last 15 years | Daily Mail Online](https://www.dailymail.co.uk/sciencetech/article-2434628/IPCC-climate-change-report-Humans-causing-global-warming-STILL-explain-Earths-barely-got-hotter-15-years.html)

[Global warming pause 'central' to IPCC climate report - BBC News](https://www.bbc.com/news/science-environment-24173504)

[Climate change 'hiatus': Scientists seek to qualify evidence of apparent global warming slowdown | The Independent | The Independent](https://www.independent.co.uk/news/science/climate-change-hiatus-scientists-seek-to-qualify-evidence-of-apparent-global-warming-slowdown-8829762.html)

[Climate change 'driving spread of crop pests' - BBC News](https://www.bbc.com/news/science-environment-23899019)

[What climate change? Fewer people than EVER believe the world is really warming up | UK | News | Express.co.uk](https://www.express.co.uk/news/uk/430649/What-climate-change-Fewer-people-than-EVER-believe-the-world-is-really-warming-up)

[Climate change models may not be accurate after all as study finds most widely overestimated global warming | Daily Mail Online](https://www.dailymail.co.uk/news/article-2419557/Climate-change-models-accurate-study-finds-widely-overestimated-global-warming.html)

['Changes will be coming soon' Climate change to devastate ecosystems by 2047 | Nature | News | Express.co.uk](https://www.express.co.uk/news/nature/435594/Changes-will-be-coming-soon-Climate-change-to-devastate-ecosystems-by-2047)

[Coastal wildlife species 'at risk due to climate change' - BBC News](https://www.bbc.com/news/uk-23803520)

<https://www.dailymail.co.uk/sciencetech/article-2403062/How-Greenland-greener-Climate-change-accelerate-spread-forests--kill-arctic-animals-experts-predict.html>

[John Kerry says there is 'irrefutable and alarming evidence' that climate change is real | Daily Mail Online](https://www.dailymail.co.uk/news/article-2408979/John-Kerry-says-irrefutable-alarming-evidence-climate-change-real.html)

1. 2014.1.1-2016.12.31

[COP21: What does the Paris climate agreement mean for me? - BBC News](https://www.bbc.co.uk/news/science-environment-35092127)

[COP21: Rallies call for Paris climate change action - BBC News](https://www.bbc.co.uk/news/world-34956825)

[Paris climate change talks: Lord Stern calls on rich countries to help poor nations cope with global warming | The Independent | The Independent](https://www.independent.co.uk/climate-change/news/paris-climate-change-talks-lord-stern-calls-on-rich-countries-to-help-poor-nations-cope-with-global-warming-a6747466.html)

[Almost all climate scientists agree: climate change is real! (theecologist.org)](https://theecologist.org/2016/apr/19/almost-all-climate-scientists-agree-climate-change-real)

[Trump: The best thing ever for climate change? - BBC News](https://www.bbc.com/news/science-environment-38034165)

<https://www.bbc.com/news/science-environment-26756005>

[Climate change: 'Monumental' deal to cut HFCs, fastest growing greenhouse gases - BBC News](https://www.bbc.co.uk/news/science-environment-37665529)

[Paris climate change agreement: China and US ratify deal as Barack Obama hails 'moment we decided to save our planet' | The Independent | The Independent](https://www.independent.co.uk/climate-change/news/paris-climate-change-agreement-china-and-us-ratify-landmark-deal-to-fight-climate-change-a7223746.html)

[Climate change: 2015 'shattered' global temperature record by wide margin - BBC News](https://www.bbc.com/news/science-environment-35354579)

[Climate change a serious security threat, warns Obama - BBC News](https://www.bbc.com/news/world-us-canada-32818355)

1. 2017.1.1-2019.12.31

<https://www.bbc.co.uk/news/science-environment-45775309>

<https://www.imperial.ac.uk/news/188517/changes-needed-middle-century-climate-experts/>

<https://www.thescottishfarmer.co.uk/news/16968975.un-issues-climate-change-warming/>

<https://www.bbc.co.uk/news/science-environment-46582025>

<https://www.bbc.com/news/world-us-canada-45859325>

<https://www.imperial.ac.uk/news/194271/climate-change-policies-need-improve-uk/>

<https://www.bbc.com/news/science-environment-48122911>

<https://www.bbc.com/news/science-environment-46347453>

<https://www.bbc.com/news/science-environment-38652746>

<https://www.wired.co.uk/article/climate-change-strike-protest-children-social-media>

1. 2020.1.1-2022.11.1

<https://www.independent.co.uk/news/uk/met-office-london-heathrow-prime-minister-greenpeace-imperial-college-london-b2126614.html>

<https://www.bbc.com/news/uk-59056725>

<https://www.bbc.co.uk/news/science-environment-59049770>

<https://www.bbc.co.uk/news/science-environment-59067471>

<https://www.bbc.co.uk/news/science-environment-52370221>

<https://www.bbc.com/news/science-environment-62915648>

<https://www.imperial.ac.uk/news/229754/major-un-report-concludes-world-off-track/>

<https://www.weforum.org/agenda/2021/07/record-breaking-temperatures-climate-change-crisis-language-emergency>

**Text S9:** Chinese local government news

Reporters learned yesterday that, to implement the Three-Year Action Plan for High-Quality Eco-Environment, Lianyungang City will fully launch the construction of an “eco-port city.”

Code: ● Domestic environmental governance\Local government

China 2018.04.16-2018.04.22 Position: 37 - 37

It is understood that Lianyungang will strengthen control over ecological red lines; on the premise that the area of ecological red lines does not decrease and management categories are not downgraded, it will enhance coastline protection and restoration, coordinate land–sea management, strictly enforce sea-use area control indicators for construction projects, and keep the natural shoreline retention rate at no less than 31%.

Code: ● Domestic environmental governance\Local government

China 2018.04.16-2018.04.22 Position: 38 - 38

Lianyungang will coordinate ecological resources, fully initiate the building of a green port city, and establish a “four-horizontal, three-vertical” framework.

Code: ● Domestic environmental governance\Local government

China 2018.04.16-2018.04.22 Position: 39 - 39

Lianyungang will also advance the establishment of eco-civilization demonstration projects, leveraging its mountain-and-sea scenery; landforms of mountains, waters, forests, fields, lakes and seas; and ecological assets such as islands and wetlands, in order to enhance its core competitiveness.

Code: ● Domestic environmental governance\Local government

China 2018.04.16-2018.04.22 Position: 40 - 40

Yang Qifeng, Deputy Director-General of the Gansu Provincial Department of Agriculture and Animal Husbandry, led a delegation including Qi Jun, Deputy Director of the Gansu Provincial Foreign Affairs Office; Li Huiliang, Division Chief of the Publicity Department of the CPC Gansu Provincial Committee; Tian Julan, Principal Staff Member of the News and Culture Division of the Provincial Foreign Affairs Office; Yang Xiaoqing, Director of the Gannan Prefecture Agriculture and Animal Husbandry Bureau; Zhang Zihua, Researcher of the Foreign Economic Division of the Provincial Department of Agriculture and Animal Husbandry; as well as Jiao Weizhong, Deputy Secretary of the Diem County CPC Committee and County Magistrate, and other responsible officials from relevant departments and units to attend the meeting.

Code: ● Domestic environmental governance\Local government

China 2018.04.16-2018.04.22 Position: 73 - 73

The first local regulation in China dedicated to biodiversity conservation—the Yunnan Provincial Regulation on Biodiversity Conservation—was promulgated and implemented. Nine major plateau lakes, including Dianchi, Fuxian Lake, Erhai and Lugu Lake, now follow a “one lake, one regulation” approach, and nine protected areas, such as Lashi Lake in Lijiang and Dashanbao in Zhaotong, have realized a “one area, one law” framework.

Code: ● Domestic environmental governance\Local government

China 2020.05.18-2020.05.24 Position: 21 - 21

International Day for Biological Diversity: Kunming harnesses the power of the “cloud” to support biodiversity conservation.

Code: ● Domestic environmental governance\Local government

China 2020.05.18-2020.05.24 Position: 30 - 30

Beijing releases interim results of its biodiversity survey, discovering 70 species newly recorded for the city.

Code: ● Domestic environmental governance\Local government

China 2021.05.17-2021.05.23 Position: 9 - 9

Yesterday, the Beijing Municipal Ecology and Environment Bureau released interim results of the biodiversity survey. According to Beijing Youth Daily, the city launched the survey in 2020 and has so far recorded 5,086 species in the field, identifying 70 species newly recorded for Beijing, including 12 species newly recorded for China. During the 14th Five-Year Plan period, the city plans to achieve full coverage of survey grids and dynamically clarify the citywide baseline status of biodiversity.

Code: ● Domestic environmental governance\Local government

China 2021.05.17-2021.05.23 Position: 10 - 10

The entire city has been divided into 212 grids to conduct the survey.

Code: ● Domestic environmental governance\Local government

China 2021.05.17-2021.05.23 Position: 11 - 11

To mark the International Day for Biological Diversity, the Provincial Government Information Office held a press conference, inviting leaders from the Provincial Department of Science and Technology, the Provincial Department of Ecology and Environment, the Provincial Department of Agriculture and Rural Affairs, and the Provincial Forestry and Grassland Administration to brief the media on major scientific achievements in the field of biodiversity, the List of New and Newly Recorded Species in Yunnan (1992–2020), the development of Yunnan’s seed industry, and achievements in the breeding and protection of new forestry plant varieties, and to take questions from reporters.

Code: ● Domestic environmental governance\Local government

China 2021.05.17-2021.05.23 Position: 16 - 16

Jiang Weiliang, a Level-II Inspector at the Provincial Department of Agriculture and Rural Affairs, gave a briefing.

Code: ● Domestic environmental governance\Local government

China 2022.05.16-2022.05.22 Position: 39 - 39

Shandong Provincial Department of Ecology and Environment: Carry out in-depth biodiversity surveys and steadily advance the protection of germplasm resources.

Code: ● Domestic environmental governance\Local government

China 2022.05.16-2022.05.22 Position: 56 - 56

This morning, the Shandong Provincial Government Information Office held a press conference at which multiple departments, including the Shandong Provincial Department of Ecology and Environment, introduced the province’s work related to biodiversity conservation.

Code: ● Domestic environmental governance\Local government

China 2022.05.16-2022.05.22 Position: 57 - 57

On May 20, the Yunnan Provincial Department of Ecology and Environment held a press event for the 2022 “May 22 International Day for Biological Diversity.”

Code: ● Domestic environmental governance\Local government

China 2022.05.16-2022.05.22 Position: 61 - 61

Haikou, Xinhua News Agency, April 22 (Reporter Zhao Yeping): According to the Hainan Provincial Department of Land, Environment and Resources, over recent decades more than 200 species in Hainan have been on the brink of extinction, such as Keteleeria hainanensis, Dacrydium pierrei, Hydnocarpus hainanensis, Madhuca pasquieri (and the Hainan endemic Madhuca hainanensis), Podocarpus imbricatus, Cephalotaxus hainanensis and Nypa fruticans; at least six plant species have disappeared, including Gymnosporia hainanensis and Sciaphila tenella.

Code: ● Domestic environmental governance\Local government

China 2011.1.1-2013.12.31 Position: 18 - 18

Despite Hainan’s ongoing measures to strengthen biodiversity conservation, it still faces great pressure and severe challenges—such as degradation of ecosystem service functions, loss of biological resources, and invasions of alien species—which have sounded the alarm for biodiversity conservation in Hainan.

Code: ● Domestic environmental governance\Local government

China 2011.1.1-2013.12.31 Position: 21 - 21

“Rich biodiversity is fundamental to ensuring Hainan’s ecological security and is the material foundation for Hainan’s green rise,” said Mao Dongli, Deputy Director-General of the Hainan Provincial Department of Land, Environment and Resources.

Code: ● Domestic environmental governance\Local government

China 2011.1.1-2013.12.31 Position: 22 - 22

At the press conference, Zhu Xiaohua—Chief Spokesperson of the Municipal CPC Committee, a Standing Committee member, and head of the Publicity Department—briefed the attending reporters on the overall situation of Shuangyashan City.

Code: ● Domestic environmental governance\Local government

China 2011.1.1-2013.12.31 Position: 58 - 58

At this side event, the experience from Jingdong County, Pu’er City, Yunnan, China, won high recognition from guests from various countries.

Code: ● Domestic environmental governance\Local government

China 2014.1.1-2016.12.31 Position: 15 - 15

County head Hu Qiwu said that, in order to protect unique ecological resources, Pu’er City and Jingdong County have formulated very strict regulations, set ecological protection red lines, and implemented a “one-vote veto” system for officials regarding ecological and environmental work.

Code: ● Domestic environmental governance\Local government

China 2014.1.1-2016.12.31 Position: 15 - 15

Executive Secretary of the Convention on Biological Diversity: Yunnan will showcase biodiversity to the world.

Code: ● Domestic environmental governance\Local government

China 2017.1.1-2019.12.31 Position: 25 - 25

The 15th meeting of the Conference of the Parties to the Convention on Biological Diversity (COP15) will be held in Kunming, Yunnan, China, in 2020.

Code: ● Domestic environmental governance\Local government

China 2017.1.1-2019.12.31 Position: 26 - 26

On the 5th, Pașca Palmer came to Kunming to guide preparations for the conference.

Code: ● Domestic environmental governance\Local government

China 2017.1.1-2019.12.31 Position: 26 - 26

According to Pașca Palmer, the 196 Parties to the CBD will gather in Kunming next October to deliberate on the post-2020 global biodiversity framework and set new global biodiversity targets for 2030.

Code: ● Domestic environmental governance\Local government

China 2017.1.1-2019.12.31 Position: 31 - 31

The 15th meeting of the Conference of the Parties to the CBD will be held in Kunming, China, in 2020.

Code: ● Domestic environmental governance\Local government

China 2017.1.1-2019.12.31 Position: 34 - 34

Biodiversity photo and eco-environment comic exhibition opens in Kunming, Yunnan.

Code: ● Domestic environmental governance\Local government

China 2017.1.1-2019.12.31 Position: 36 - 36

On the morning of January 17, the exhibition hosted by the Provincial Department of Ecology and Environment opened at the Kunming City Museum, showcasing to the public Yunnan’s colorful biodiversity and allowing visitors to appreciate the province’s ecological beauty.

Code: ● Domestic environmental governance\Local government

China 2017.1.1-2019.12.31 Position: 37 - 37

Gao Zhengwen, a member of the Party Leadership Group and Deputy Director-General of the Provincial Department of Ecology and Environment, attended the opening ceremony and delivered remarks. Also present were heads of relevant divisions and affiliated institutions of the department; representatives from the Kunming City Museum, the School of Fine Arts of Yunnan Arts University, the Yunnan Plateau Ecology Environmental Protection Foundation, and the Yunnan Ethnic Culture and Art Promotion Association; as well as faculty and student representatives from the School of Fine Arts of Yunnan Arts University and Kunming Fengyuan Primary School, artists, contributors and institutional representatives of exhibited works, provincial green schools and green communities, university student associations, and some news media.

Code: ● Domestic environmental governance\Local government

China 2017.1.1-2019.12.31 Position: 38 - 38

Chen Li, Director of the Publicity and Education Division of the Department of Ecology and Environment, presided over the opening ceremony.

Code: ● Domestic environmental governance\Local government

China 2017.1.1-2019.12.31 Position: 38 - 38

All regions and departments have adopted active and effective measures to foster, guide, and support all sectors of society in undertaking biodiversity conservation efforts.

Code: ● Domestic environmental governance\Local government

China 2020.1.1-2022.11.1 Position: 6 - 6

**SUPPLEMENTARY TABLES**

**Table S1.** Analyzed time periods of peak and regular time in China and the UK (Bio means “biodiversity”, CC means “climate change”).

|  | **Bio peak** | **Bio regular** | **CC peak** | **CC regular** | **Total periods** |
| --- | --- | --- | --- | --- | --- |
| **China** | **6** | **4** | **5** | **4** | **19** |
| **UK** | **5** | **4** | **4** | **4** | **17** |

**Table S2.** Total text words, primary codes categories and codes numbers of peak and regular time in China and the UK (Bio means “biodiversity”, CC means “climate change”).

|  | **Bio peak** | **Bio regular** | **CC peak** | **CC regular** |
| --- | --- | --- | --- | --- |
| **CN text words** | **28729** | **21647** | **27351** | **21418** |
| **CN primary codes categories** | **18** | **16** | **17** | **17** |
| **CN total codes numbers** | **811** | **671** | **779** | **651** |
| **UK text words** | **13570** | **11042** | **8500** | **11309** |
| **UK primary codes categories** | **18** | **16** | **14** | **20** |
| **UK total codes numbers** | **937** | **755** | **804** | **783** |

**Table S3.** Code system of biodiversity

| Code System |
| --- |
| Domestic environmental governance |
| Domestic environmental governance\Two-mountain theory |
| Domestic environmental governance\Chinese biodiversity Conservation Strategy and Action Plan |
| Domestic environmental governance\China environmental leadership |
| Domestic environmental governance\Ecological civilization |
| Domestic environmental governance\Ecological red lines |
| Domestic environmental governance\Belt and Road Routes |
| Domestic environmental governance\Green development |
| Domestic environmental governance\Cross-sectoral cooperation |
| Domestic environmental governance\Conservation demonstration |
| Domestic environmental governance\Creative narrative |
| Domestic environmental governance\Conservation measures |
| Domestic environmental governance\Conservation measures\Species conservation |
| Domestic environmental governance\Conservation measures\Habitat conservation |
| Domestic environmental governance\Local government |
| Domestic environmental governance\International Day for Biological Diversity |
| Domestic environmental governance\Compensation mechanism |
| Domestic environmental governance\Policy |
| Domestic environmental governance\Policy\Network Rail |
| Domestic environmental governance\Kunming Declaration |
| Domestic environmental governance\Prosecuting/cracking down on illegal crimes |
| Domestic environmental governance\Celebrity |
| Domestic environmental governance\NPPF |
| Domestic environmental governance\Environment Bill |
| Domestic environmental governance\Biodiversity Action Plan (2015-2020) |
| Domestic environmental governance\Hackney Council |
| Domestic environmental governance\UK environmental leadship |
| Domestic environmental governance\Intensive farming |
| Domestic environmental governance\Blue Belt programme |
| International environmental  governance |
| International environmental  governance\Convention on Biological Diversity |
| International environmental  governance\Convention on Biological Diversity\GBF |
| International environmental  governance\Convention on Biological Diversity\Aichi targets |
| International environmental  governance\Convention on Biological Diversity\Convention on Biological Diversity(COP15) |
| International environmental  governance\Conservation measures |
| International environmental  governance\Conservation measures\Habitat conservation |
| International environmental  governance\Conservation measures\Species conservation |
| International environmental  governance\International environmental policy |
| International environmental  governance\International cooperation |
| International environmental  governance\United Nations |
| International environmental  governance\United Nations\Environment and sustainable development Conferences |
| International environmental  governance\United Nations\United Nations Earth Programme |
| International environmental  governance\United Nations\UNEP |
| International environmental  governance\United Nations\Knowledge platform |
| International environmental  governance\United Nations\Leaders Pledge for Nature |
| International environmental  governance\United Nations\FAO |
| International environmental  governance\IUCN |
| International environmental  governance\IUCN\World Conservation Congress |
| International environmental  governance\Education |
| International environmental  governance\WWF |
| International environmental  governance\IPBES |
| International environmental  governance\European Union |
| International environmental  governance\European Union\Sand oil trade |
| International environmental  governance\World Day to Combat Desertification and Drought |
| International environmental  governance\Visionary Perspective Plan |
| International environmental  governance\World Environment Day |
| International environmental  governance\World Database on Protected Areas |
| International environmental  governance\National Ecosystem Assessments |
| International environmental  governance\TNFD |
| International environmental  governance\International Biodiversity Day |
| International environmental  governance\Incentives and capacity building |
| International environmental  governance\Assessment |
| International environmental  governance\Financial support |
| International environmental  governance\BIP |
| International environmental  governance\World Economic Forum |
| International environmental  governance\OMFIF |
| International environmental  governance\China environmental leadship |
| Nature’s contributions to people |
| Nature’s contributions to people\Negative contribution |
| Nature’s contributions to people\Negative contribution\Non-material |
| Nature’s contributions to people\Negative contribution\Non-material\Learning and inspiration |
| Nature’s contributions to people\Negative contribution\Non-material\Learning and inspiration\Tourist |
| Nature’s contributions to people\Negative contribution\Material and assistance |
| Nature’s contributions to people\Negative contribution\Material and assistance\Assistance |
| Nature’s contributions to people\Negative contribution\Material and assistance\Assistance\Forest decline |
| Nature’s contributions to people\Negative contribution\Material and assistance\Food security problem |
| Nature’s contributions to people\Negative contribution\Material and assistance\Food security problem\Food reduction |
| Nature’s contributions to people\Negative contribution\Material and assistance\Food security problem\Food reduction\Fishery |
| Nature’s contributions to people\Negative contribution\Material and assistance\Economic risk |
| Nature’s contributions to people\Negative contribution\Regulation of environmental process |
| Nature’s contributions to people\Negative contribution\Regulation of environmental process\Disease |
| Nature’s contributions to people\Negative contribution\Regulation of environmental process\Nature disaster |
| Nature’s contributions to people\Negative contribution\Regulation of environmental process\Shortage of fresh water |
| Nature’s contributions to people\Negative contribution\Regulation of environmental process\Climatic instability |
| Nature’s contributions to people\Negative contribution\Regulation of environmental process\Pollination loss |
| Nature’s contributions to people\Negative contribution\Regulation of environmental process\Soil health problem |
| Nature’s contributions to people\Negative contribution\Regulation of environmental process\habitat loss |
| Nature’s contributions to people\Negative contribution\Regulation of environmental process\habitat loss\Desertificastion and drought |
| Nature’s contributions to people\Negative contribution\Species extinction/endangered species |
| Nature’s contributions to people\Positive contribution |
| Nature’s contributions to people\Positive contribution\Non-material |
| Nature’s contributions to people\Positive contribution\Non-material\Learning and inspiration |
| Nature’s contributions to people\Positive contribution\Non-material\Physical and psychological experiences |
| Nature’s contributions to people\Positive contribution\Regulation of environmental process |
| Nature’s contributions to people\Positive contribution\Regulation of environmental process\Habitat creation and maintenance |
| Nature’s contributions to people\Positive contribution\Regulation of environmental process\Regulation of detrimental  organisms and biological  processes |
| Nature’s contributions to people\Positive contribution\Regulation of environmental process\Climate regulation |
| Nature’s contributions to people\Positive contribution\Regulation of environmental process\Water regulation |
| Nature’s contributions to people\Positive contribution\Regulation of environmental process\Soil protection |
| Nature’s contributions to people\Positive contribution\Regulation of environmental process\Pollination |
| Nature’s contributions to people\Positive contribution\Material and assistance |
| Nature’s contributions to people\Positive contribution\Material and assistance\Food and feed |
| Nature’s contributions to people\Positive contribution\Material and assistance\Energy |
| Nature’s contributions to people\Positive contribution\Material and assistance\Medicine |
| Nature’s contributions to people\Positive contribution\Material and assistance\Economic development |
| Biodiversity threat |
| Biodiversity threat\Indirect driver |
| Biodiversity threat\Indirect driver\Eeconomic and technological |
| Biodiversity threat\Indirect driver\Eeconomic and technological\Wildlife trade |
| Biodiversity threat\Indirect driver\Demographic and socioculture |
| Biodiversity threat\Indirect driver\Institutions and governance |
| Biodiversity threat\Indirect driver\Demographic  and  sociocultural |
| Biodiversity threat\Indirect driver\Demographic  and  sociocultural\Population |
| Biodiversity threat\Direct driver |
| Biodiversity threat\Direct driver\Invasive species |
| Biodiversity threat\Direct driver\Pollution |
| Biodiversity threat\Direct driver\Land/sea-use change |
| Biodiversity threat\Direct driver\Land/sea-use change\Human activity |
| Biodiversity threat\Direct driver\Land/sea-use change\Human activity\Infrastructure |
| Biodiversity threat\Direct driver\Land/sea-use change\Human activity\Forestry |
| Biodiversity threat\Direct driver\Land/sea-use change\Human activity\Agriculture |
| Biodiversity threat\Direct driver\Land/sea-use change\Human activity\Industry |
| Biodiversity threat\Direct driver\Direct exploitation |
| Biodiversity threat\Direct driver\Pests and diseases |
| Transformative change |
| Stakeholder |
| Stakeholder\Private sector |
| Stakeholder\Private sector\Sand oil company |
| Stakeholder\Private sector\Palm oil company |
| Stakeholder\Private sector\Green product company |
| Stakeholder\Private sector\Digital company |
| Stakeholder\Private sector\ETF company |
| Stakeholder\Private sector\Organic farming |
| Stakeholder\Private sector\Household company |
| Stakeholder\Private sector\Gas company |
| Stakeholder\Private sector\Banking industry |
| Stakeholder\Private sector\Environment consultancy |
| Stakeholder\Private sector\Food company |
| Stakeholder\Private sector\Energy sector |
| Stakeholder\Civil society |
| Stakeholder\Civil society\Young People |
| Stakeholder\Civil society\Indigenous peoples and local communities |
| Stakeholder\Civil society\NGO |
| Stakeholder\Civil society\Farmer |
| Stakeholder\Civil society\Zoo |
| Stakeholder\Civil society\Botanical garden |
| Stakeholder\Civil society\NPO |
| Stakeholder\Civil society\Garden |
| Sustainable Development Goals |
| Sustainable Development Goals\Sustainable development |
| Sustainable Development Goals\SDG2:End hunger |
| Sustainable Development Goals\SDG2:End hunger\ETF |
| Sustainable Development Goals\SDG4:Ensure education |
| Sustainable Development Goals\SDG12:Ensure sustainable consumption |
| Sustainable Development Goals\SDG15:Protect, restore and promote sustainable use of terrestri |
| Sustainable Development Goals\SDG17:Global Partnership |
| Sustainable Development Goals\SDG1:End poverty |
| Sustainable Development Goals\SDG7:Ensure energy |
| Sustainable Development Goals\SDG8:Promote employment |
| Sustainable Development Goals\SDG10:Reduce inequality within and among countries |
| Sustainable Development Goals\SDG11:Make cities and human settlements sustainable |
| Sustainable Development Goals\SDG16:Promote peaceful and inclusive societies |
| Climate change |
| Nexus thinking |
| Science research and technology |
| Urban biodiversity |
| Urban biodiversity\Green space |
| Biodiversity hotspot |
| Conservation effectiveness |
| Conservation effectiveness\Positive |
| Conservation effectiveness\Negative |
| Biodiversity value |
| Biodiversity value\Relationnal value |
| Biodiversity value\Intrinsic value |
| Biodiversity value\Instrumental value |
| Biodiversitry |
| Biodiversitry\Ecosystem diversity |
| Biodiversitry\Species diversity |
| Biodiversitry\Genetic variability |
| Worldview and values |
| Worldview and values\Welfare |
| Charismatic species |
| Science popularization |
| Protect urgency |

**Table S4.** Code system of climate change

| Code System |
| --- |
| Domestic environmental governance |
| Domestic environmental governance\China environmental leadership |
| Domestic environmental governance\Green development |
| Domestic environmental governance\Belt and Road Initiative |
| Domestic environmental governance\Ecological civilization |
| Domestic environmental governance\Meteorological support |
| Domestic environmental governance\Carbon peaking and carbon neutrality |
| Domestic environmental governance\The 13th Five-Year Plan |
| Domestic environmental governance\Cross-sectoral cooperation |
| Domestic environmental governance\Local government |
| Domestic environmental governance\Beijing Winter Olympics |
| Domestic environmental governance\The 12th Five-Year Plan |
| Domestic environmental governance\G20 |
| Domestic environmental governance\Carbon-sink market |
| Domestic environmental governance\Policy |
| Domestic environmental governance\Policy\Policy and Action Report on Climate Change |
| Domestic environmental governance\Climate change adaptation strategies |
| Domestic environmental governance\UK environmental leadship |
| Domestic environmental governance\Financial support |
| Domestic environmental governance\Committee on Climate Change:climate targets |
| International environmental  governance |
| International environmental  governance\UNFCCC |
| International environmental  governance\UNFCCC\UNFCCC(COP21) |
| International environmental  governance\UNFCCC\UNFCCC(COP21)\Paris Agreement |
| International environmental  governance\UNFCCC\UNFCCC(COP21)\Paris Agreement\1.5℃ goal |
| International environmental  governance\UNFCCC\UNFCCC(COP26) |
| International environmental  governance\UNFCCC\Climate action summit |
| International environmental  governance\UN |
| International environmental  governance\UN\UNEP |
| International environmental  governance\UN\WHO |
| International environmental  governance\UN\WMO |
| International environmental  governance\IPCC |
| International environmental  governance\International cooperation |
| International environmental  governance\Donor-Advised Fund |
| International environmental  governance\World meteorological day |
| International environmental  governance\WWF |
| International environmental  governance\Climate Action Tracker |
| International environmental  governance\Financial support |
| International environmental  governance\Financial support\Jeff Bezos Earth Fund |
| International environmental  governance\Earth Day |
| International environmental  governance\Pacific Islands Forum |
| International environmental  governance\Montreal Protocol |
| International environmental  governance\Carbon markets |
| Climate change impacts |
| Climate change impacts\Physical climate conditions (+) |
| Climate change impacts\Physical climate conditions (+)\Global warming |
| Climate change impacts\Physical climate conditions (+)\Extreme hot |
| Climate change impacts\Physical climate conditions (+)\Loss of glaciers |
| Climate change impacts\Physical climate conditions (+)\Sea level rise |
| Climate change impacts\Physical climate conditions (+)\El Niño |
| Climate change impacts\Physical climate conditions (+)\Wildfires |
| Climate change impacts\Physical climate conditions (+)\Drought |
| Climate change impacts\Physical climate conditions (+)\volcanic activity |
| Climate change impacts\Physical climate conditions (+)\Air pollution |
| Climate change impacts\Physical climate conditions (+)\Air pollution\Haze |
| Climate change impacts\Physical climate conditions (+)\Ice Age |
| Climate change impacts\Physical climate conditions (+)\Extreme cooling |
| Climate change impacts\Physical climate conditions (+)\La Niña |
| Climate change impacts\Physical climate conditions (+)\Typhoon |
| Climate change impacts\Physical climate conditions (+)\Extreme snowfall |
| Climate change impacts\Physical climate conditions (+)\Flood |
| Climate change impacts\Physical climate conditions (+)\Extreme rainfall |
| Climate change impacts\Physical climate conditions (+)\Low wind speed |
| Climate change impacts\Related losses and damges |
| Climate change impacts\Related losses and damges\Water availability and food production |
| Climate change impacts\Related losses and damges\Water availability and food production\Water shortage |
| Climate change impacts\Related losses and damges\Water availability and food production\Food security problem |
| Climate change impacts\Related losses and damges\Water availability and food production\Food security problem\Farmland destruction |
| Climate change impacts\Related losses and damges\Water availability and food production\Food security problem\Hungry |
| Climate change impacts\Related losses and damges\Water availability and food production\Food security problem\Pests |
| Climate change impacts\Related losses and damges\Health and well-being |
| Climate change impacts\Related losses and damges\Health and well-being\Negative population growth |
| Climate change impacts\Related losses and damges\Health and well-being\War |
| Climate change impacts\Related losses and damges\Health and well-being\Rising prices |
| Climate change impacts\Related losses and damges\Health and well-being\Immigration |
| Climate change impacts\Related losses and damges\Health and well-being\Death |
| Climate change impacts\Related losses and damges\Health and well-being\Disease |
| Climate change impacts\Related losses and damges\Cities, settlements and infrastructure |
| Climate change impacts\Related losses and damges\Cities, settlements and infrastructure\Settlement damage |
| Climate change impacts\Related losses and damges\Cities, settlements and infrastructure\Power supply problem |
| Climate change impacts\Related losses and damges\Cities, settlements and infrastructure\Economy |
| Climate change impacts\Related losses and damges\Cities, settlements and infrastructure\City disapperance |
| Climate change impacts\Related losses and damges\Cities, settlements and infrastructure\Flood damage in costal area |
| Climate change impacts\Related losses and damges\Cities, settlements and infrastructure\Mountain area |
| Climate change impacts\Related losses and damges\Cities, settlements and infrastructure\National security |
| Climate change impacts\Related losses and damges\Cities, settlements and infrastructure\Flood |
| Biodiversity |
| Biodiversity\Terrestrial |
| Biodiversity\Terrestrial\Forest |
| Biodiversity\Terrestrial\Citrus tree propagation |
| Biodiversity\Terrestrial\Birds |
| Biodiversity\Terrestrial\Purple heather |
| Biodiversity\Terrestrial\Pollination |
| Biodiversity\Terrestrial\Insect |
| Biodiversity\Terrestrial\Greenland |
| Biodiversity\Terrestrial\Sakura |
| Biodiversity\Freshwater |
| Biodiversity\Marine |
| Biodiversity\Marine\Polar bear |
| Biodiversity\Marine\Coral |
| Biodiversity\Marine\Coastal wildlife |
| Climate threat and reason |
| Climate threat and reason\Greenhouse gas emission |
| Climate threat and reason\Land use change |
| Climate threat and reason\Land use change\Glacier positive feedback |
| Climate threat and reason\Unsustainable energy use |
| Climate threat and reason\Solar activity |
| Climate threat and reason\Environmentally unfriendly food |
| Mitigation and Adaptation |
| Mitigation and Adaptation\Urgency |
| Mitigation and Adaptation\Mitigation |
| Mitigation and Adaptation\Mitigation\Emission cut |
| Mitigation and Adaptation\Mitigation\Emission cut\Net zero |
| Mitigation and Adaptation\Mitigation\Emission cut\Public transport |
| Mitigation and Adaptation\Mitigation\New energy |
| Mitigation and Adaptation\Mitigation\Land conservation and restoration |
| Mitigation and Adaptation\Mitigation\Land conservation and restoration\Forest |
| Mitigation and Adaptation\Mitigation\Green product |
| Mitigation and Adaptation\Mitigation\Stop deforestation |
| Mitigation and Adaptation\Mitigation\Suck CO2 |
| Mitigation and Adaptation\Mitigation\‘Clean’ technologies |
| Mitigation and Adaptation\Adaptation |
| Mitigation and Adaptation\Adaptation\Climate monitoring and prediction |
| Mitigation and Adaptation\Adaptation\Improve air quality |
| Mitigation and Adaptation\Adaptation\Disaster risk management capacity |
| Mitigation and Adaptation\Adaptation\Building adaptable cities |
| Mitigation and Adaptation\Adaptation\Carbon emission accounting |
| Mitigation and Adaptation\Adaptation\Food supply |
| Approach effectiveness |
| Approach effectiveness\Negative |
| Approach effectiveness\Negative\China bashing |
| Approach effectiveness\Postive |
| Sustainable Development Goals |
| Sustainable Development Goals\SDG1:End poverty |
| Sustainable Development Goals\SDG2:End hunger |
| Sustainable Development Goals\SDG8:Promote employment |
| Sustainable Development Goals\SDG12:Ensure sustainable consumption |
| Sustainable Development Goals\SDG13:Take urgent action to combat climate change |
| Stakeholder |
| Stakeholder\Private sector |
| Stakeholder\Private sector\Emerging economies |
| Stakeholder\Private sector\Enviromental protection enterprise |
| Stakeholder\Private sector\Google |
| Stakeholder\Civil society |
| Stakeholder\Civil society\Individuals and communities |
| Stakeholder\Civil society\NGO |
| Stakeholder\Civil society\NGO\TNC |
| Stakeholder\Civil society\NGO\National Trust |
| Stakeholder\Civil society\Public welfare |
| Stakeholder\Civil society\Youth |
| Stakeholder\Civil society\Children |
| Environmental injustice |
| Nexus thinking |
| Conspiracy theory |
| Conspiracy theory\Koch family |
| Conspiracy theory\Global warming slowdown |
| Conspiracy theory\Opposition |
| Transformative change |
| Science research |
| Worldview and values |
| Worldview and values\Literature |
| Celebrities |
| Celebrities\Joey Barton |
| Celebrities\Greta Thunberg |
| Celebrities\Emma Thompson |
| Celebrities\The Simpsons |
| Celebrities\Obama |
| Celebrities\Trump |
| Celebrities\Queen |
| Political agenda |
| Political agenda\American arms race |
| Concept of climate change |
| Climate change protest |
| Climate change protest\Climate strike |
| Climate change protest\Extinction Rebellion |
| Financial crisis |
| Brexit |
| COVID |
