# Supplementary material for: Public interest in biodiversity and climate change: A comparative culturomics study of China and the UK
Source: PLoS One. 2026 Jan 14;21(1):e0338006. doi: 10.1371/journal.pone.0338006 (PMC12803463; doi:10.1371/journal.pone.0338006)
Supplement: S2 Text — (DOCX) [file pone.0338006.s002.docx]

**News in China**

**Biodiversity temporal pattern news**

2011.1.1-2013.12.31

1.

Environmentalists Call for Strengthened Biodiversity Protection on International Day for Biological Diversity

May 22 is the International Day for Biological Diversity. On this day, environmentalists from the United Nations Environment Programme (UNEP), China’s Ministry of Environmental Protection, university environmental societies, and other domestic and international organizations called for stronger biodiversity protection.

“We are not qualified to be the Earth’s partners or friends—we are the Earth’s descendants. Without nature, there is no biodiversity. Humans are also one species within biodiversity. But it seems that humans have become a deadly species, so I hope everyone will attach great importance to understanding biodiversity.”

This appeal was issued in Beijing on May 22 by Zhang Xinsheng, President of the IUCN Council.

Biodiversity refers to the totality of ecosystems formed by living organisms and the environment, as well as the ecological processes associated with them. It is the condition for human survival, the foundation for sustainable economic and social development, and the guarantee of ecological and food security.

Statistics show that about 36.3% of species worldwide are on the verge of extinction, and more than half of China’s mammal populations have sharply declined. Currently, one species disappears from the world every hour. Zhang Shigang, UNEP’s representative in China, pointed out that human activities are the main cause of large-scale biodiversity loss. “We see that humans have gained more net benefits from natural habitats and biodiversity, but this usually comes with the reduction of other ecosystem services, without repair or necessary investment in the functions damaged. This has led to various environmental problems—climate, atmosphere, water, soil—and frequent natural disasters.”

2.

Global Scientists Issue the “Kunming Declaration” to Promote Biodiversity Protection

Kunming, October 31 (China News Service, Reporter Gu Yihang) — On the 31st, at the Fifth International Barcode of Life Conference held in Kunming, more than 400 scientists from 43 countries jointly issued the “Kunming Declaration,” calling for strengthened international cooperation in the field of DNA barcoding to promote global biodiversity conservation and development.

DNA barcoding is a new technology that uses standard DNA fragments to rapidly identify species. In recent years, it has become a research hotspot and frontier in life sciences and biotechnology. At this conference in Kunming, “biodiversity conservation” became the focus of attention for scientists from various countries.

Li Dezhu, Director of the Kunming Institute of Botany, Chinese Academy of Sciences, said: “Against the backdrop of global climate anomalies, biodiversity is under severe threat. In recent years, DNA barcoding technology has greatly enhanced humanity’s ability to monitor, understand, and use biodiversity, providing new approaches for biodiversity research and protection. Scientists also recognize that international cooperation will greatly promote the development of barcoding technology.”

During the five-day seminar, scientists reached consensus and issued the “Kunming Declaration” on “using DNA barcoding technology to promote international cooperation in biodiversity protection” and “the important role of Yunnan in global biodiversity conservation.”

The declaration was jointly drafted by Chinese scientist Li Dezhu, American scientist David Castle, Canadian scientist Robert Hanner, and British scientist Pete Hollingsworth. It proposed further strengthening global cooperation on barcoding technology; formulating and unifying technical standards for barcoding research; promoting cooperation among governments, intergovernmental organizations, the private sector, and NGOs in the international barcoding initiative; and strengthening global talent-training systems in barcoding technology.

3.

Hainan Faces Severe Biodiversity Conservation Challenges: Over 200 Species on the Brink of Extinction

Haikou, April 22 (Xinhua, Reporter Zhao Yeping) — According to the Hainan Provincial Department of Land, Environment and Resources, over the past few decades, more than 200 species in Hainan have been on the brink of extinction, such as Hainan oilsong (Keteleeria hainanensis), lu jun pine, Calophyllum hainanense, Dalbergia odorifera, Pinus fenzeliana, Taxus chinensis var. mairei, and nipa palm; at least six plant species have disappeared, including Vatica hainanensis and Euphorbia nematocypha.

Surveys show that among Hainan’s 362 bird species, more than 100 have become hard to find. Many species that were once common have now become rare and endangered. In addition, over-harvesting of wild orchids and wild Dalbergia odorifera, and over-logging of protected plants such as Dracaena cambodiana, Adenanthera microsperma, and Aquilaria sinensis have led to declining populations in the wild.

The situation of invasive alien species is also worrying. Of the more than 180 invasive plants common in China, Hainan has more than 90. Chromolaena odorata and Ageratum conyzoides have spread widely along forest edges, wastelands, and roadsides across the island. Eichhornia crassipes (water hyacinth) and the African giant snail have already harmed some water bodies.

Hainan ranks among the top in China in terms of species richness and endemic taxa, and is regarded as a natural gene bank for China and the world. Despite multiple measures taken to strengthen biodiversity protection, Hainan still faces enormous pressure and severe challenges, such as ecosystem service degradation, loss of biological resources, and invasive species. These have sounded an alarm for biodiversity protection in Hainan.

“Rich biodiversity is the fundamental guarantee for Hainan’s ecological security and the material basis for Hainan’s green rise,” said Mao Dongli, Deputy Director of the Hainan Provincial Department of Land, Environment and Resources.

Recently, the Hainan Provincial Biodiversity Conservation Strategy and Action Plan project was launched in Haikou. This new measure aims to achieve the protection and sustainable use of biodiversity and build Hainan into a national demonstration zone for biodiversity conservation.

4.

Latin America Has Lost 30% of Its Biodiversity Over the Past 20 Years

Beijing, July 17 (People’s Daily Online) — According to Spain’s Rebellion newspaper today, machines are uprooting big trees, ponds are being filled, and forests, buffer zones, mountains, and riverbanks are being eroded. As populations grow, natural areas are being turned into housing and cities, forcing species that once lived in these ecosystems to migrate. With these scenes becoming more frequent, nature is being destroyed.

According to the latest report released this week by the United Nations at the Ninth International Conference on Environment and Development held in Cuba, Latin America has lost 30% of its biodiversity over the past 20 years.

Margarita Astralaga, Director of UNEP’s Regional Office for Latin America and the Caribbean, said at the meeting: “The conclusion is that we have reached a dead end.” She questioned the continent’s development model and consumption patterns.

Although Latin American countries have adopted environmental policies, such as ending emissions of ozone-depleting substances or stopping the use of leaded fuels, these have not been enough to halt environmental destruction. Experts warn that the risks are now greater, as environmental issues are increasingly tied to climate change.

In the Caribbean, one of the most serious damages is coral bleaching. The report estimates that if the current decline continues, these corals will die by 2070. The disappearance of these ecosystems will eliminate natural barriers against hurricanes, while also damaging tourism and fisheries. Another risk is growing ecological losses in Patagonia, Bolivia, and Chile.

Brazil’s Amazon rainforest is also under threat. In May this year alone, 464.96 square kilometers were lost—4.7 times more than the same period last year. Brazil’s National Institute for Space Research warned that the data are incomplete because of fog: at the time of measurement, fog covered 42% of the forest area, compared with 32% a year earlier.

5.

Study Finds Earth’s Biodiversity Increases with Climate Warming

A study re-examining fossil and environmental data from the geological record reports that Earth’s biodiversity has increased as the planet has warmed. An earlier influential study covering the same geological periods had concluded that biodiversity decreased with rising temperatures, a result that contrasted with most ecological studies showing that species richness consistently declines near the poles in both marine and terrestrial environments.

The researchers argued that it seemed unlikely that high temperatures would suppress biodiversity over time while simultaneously enhancing it spatially. They reanalyzed patterns of marine invertebrate biodiversity over the past 540 million years. The team separated out the most reliable predictors of biodiversity from poorly understood variables that could obscure interpretation. They found that biodiversity changed in step with temperature throughout Earth’s history: higher temperatures were associated with both increased extinction rates and increased speciation rates.

The researchers concluded that although global warming historically promoted extinctions, the appearance of new species exceeded losses, leading to an overall increase in biodiversity. They cautioned, however, that the current trend—a combination of rising temperatures and declining biodiversity—appears inconsistent with the long-term equilibrium sustained throughout Earth’s history.

6.

Scientists Reveal Evolutionary History of Plateau Amphibians to Advance Biodiversity Conservation

As human impacts on the environment intensify, global biodiversity is facing a severe crisis. On the 18th, the Kunming Institute of Zoology, Chinese Academy of Sciences, announced that its scientists had made progress in studying the evolutionary history of plateau wood frog populations distributed on the northeastern edge of the Qinghai–Tibet Plateau. The findings reveal how organisms respond to climatic and environmental change, providing important guidance for species diversity and ecological protection in northwestern China.

Today, human activity is driving unprecedented levels of species extinction, with biodiversity and ecosystems vanishing at an alarming pace. Protecting and managing biodiversity to prevent rapid loss has become a global consensus and research priority. Determining biodiversity patterns and understanding the mechanisms that generate and sustain them are prerequisites for effective conservation strategies.

Recently, the team led by Academician Zhang Yaping and Researcher Che Jing made new progress in reconstructing the evolutionary history of plateau wood frogs along the northeastern edge of the Qinghai–Tibet Plateau.

According to the project’s researchers: “Compared with known responses of plants, small rodents, and birds to environmental fluctuations in the Late Cenozoic—especially since the Quaternary glaciations—plateau wood frogs displayed a different evolutionary pattern.”

The study found multiple glacial refugia in the northeastern margins of the plateau and in northern Qilian Mountains. Northern frog populations were highly differentiated, with groups isolated around inland rivers such as the Shiyang River and Heihe River along the northern slopes of the Qilian Mountains. In contrast, frog populations south of the Qilian Mountains experienced significant demographic expansions. Among them, the Hengduan Mountain populations clearly formed by expanding southward after the Last Glacial Maximum.

7.

EU Unveils 10-Year Biodiversity Protection Strategy

Over the past year, several countries have introduced biodiversity protection strategies. On the 3rd, the European Union released its 10-year plan entitled Our Life Insurance, Our Natural Capital: An EU Biodiversity Strategy to 2020. The strategy highlights the EU’s deep concern and strong commitment to biodiversity issues.

The EU now regards biodiversity protection as a major issue essential for survival and development. On the one hand, biodiversity is closely tied to economic development and employment in the EU—for example, one in every six jobs is related to the environment and biodiversity. On the other hand, the EU believes that its biodiversity faces a severe crisis, causing serious economic and social losses. According to EU statistics, reduced insect pollination due to biodiversity loss alone costs the EU an estimated €15 billion annually.

The EU’s 10-year strategy notes that biodiversity protection can bring enormous returns. Numerically, if the world meets its current biodiversity targets, the cumulative economic gains by 2050 could range from $2 trillion to $6 trillion. The strategy also cites concrete examples: Denmark’s Skjern River restoration project produced direct economic benefits of 67 million to 228 million Danish kroner (1 DKK ≈ 0.2 USD). Likewise, between 25% and 50% of pharmaceutical industry profits are linked to biodiversity, as is a significant proportion of the natural cosmetics sector.

8.

UNEP: Global Biodiversity Critically Important to Humanity

Nairobi, May 22 (Xinhua, Reporters Song Chen and Guo Qian) — Jacqueline Alder, Head of the Freshwater and Marine Ecosystem Branch of the United Nations Environment Programme (UNEP), recently stated that global biodiversity is facing severe challenges. Humanity must pursue sustainable development paths that protect global biological and ecological systems while achieving economic and social progress.

On the eve of International Day for Biological Diversity (May 22), Alder told Xinhua in Nairobi that many industries worldwide rely on resources from ecosystems, making ecosystem protection vitally important to human development. She emphasized that protecting biodiversity is a major “challenge” requiring correct approaches to balance development with biodiversity conservation and achieve harmony between humans and nature. UNEP’s advocacy of a “green economy” focuses on sustainable use of natural resources, promoting biodiversity conservation alongside economic development.

This year’s theme for International Day for Biological Diversity is “Marine Biodiversity.” Alder explained that UNEP is taking measures to protect marine life, such as “Blue Carbon” initiatives. “Blue carbon” refers to carbon stored in coastal and marine ecosystems. Compared with mature tropical forests, coastal ecosystems can capture and transfer greater amounts of carbon from the ocean and atmosphere. UNEP’s 2009 report noted that marine organisms absorb and sequester half of the world’s total greenhouse gases absorbed by all organisms. Thus, protecting marine biodiversity can reduce global fossil-fuel carbon emissions and slow climate warming. Conversely, once coastal ecosystems are damaged, they cease absorbing CO₂ and release centuries of stored carbon, becoming new sources of emissions. Therefore, protection of coastal ecosystems should receive strong international and national attention.

9.

Press Conference Held Following Northeast Asia Wetland Biodiversity Forum in Shuangyashan

Shuangyashan, September 17 (Northeast Net) — On the afternoon of September 16, following the conclusion of the China–Northeast Asia Wetland Biodiversity Conservation Forum in Shuangyashan, the forum’s organizing committee held a press conference at Dongshan International Hotel.

Participants included Zhu Xiaohua, Chief Spokesperson of the CPC Municipal Committee, Standing Committee member, and Propaganda Minister; Ma Jianzhang, Academician of the Chinese Academy of Engineering and professor at Northeast Forestry University; and Wang Shijun, Chief Spokesperson and Secretary-General of the Municipal Government. They presented an overview of Shuangyashan and reported on both academic and non-academic outcomes of the forum.

With the theme “Wetland Protection, Ecological Civilization, Harmonious Development,” the China–Northeast Asia Wetland Biodiversity Conservation Forum was a high-level, high-standard meeting that produced fruitful results. Through the forum, Shuangyashan’s municipal leadership aimed to build a platform for openness and cooperation among wetland cities, strengthen academic discussions and experience-sharing on wetland conservation and use, promote wetland resource protection and urban development, and further awaken people’s sense of responsibility—toward nature, by cherishing wetlands and protecting ecosystems; toward sustainable development, by conserving resources and using them wisely; and toward future generations, by caring for the environment and pursuing harmony.

At the press conference, Zhu Xiaohua introduced the city’s general situation: “Shuangyashan has a long history, rich ecology, abundant resources, and fertile products. It is hailed as ‘the capital of the ancient Yilou kingdom, city of wetlands, base of coal and power, and granary of the North.’ The city’s profile can be summarized as: its advantage lies in resources, its specialty in ecology, its foundation in agriculture, its support in industry, and its heritage in culture.”

10.

Five Major Problems Threaten China’s Biodiversity Protection

This year marks the first year of full implementation of China’s National Biodiversity Conservation Strategy and Action Plan (2011–2030). On July 3, Jin Jianming, Director of the Expert Committee of the China Biodiversity Conservation and Green Development Foundation and Academician of the Chinese Academy of Engineering, told a reporter from China Economic Times that, in addition to raising public awareness and improving the legal framework, five major problems currently threaten biodiversity conservation. He emphasized the urgent need to strengthen biodiversity protection, management, supervision, and public outreach.

China is one of the world’s 12 megadiverse countries, but due to multiple factors, the overall trend of biodiversity decline has not been effectively curbed. Key problems include:

Accelerated urbanization and industrialization threatening habitats;

Disorderly development activities causing over-exploitation of biological resources;

Environmental pollution severely impacting ecosystems;

The threat of invasive alien species;

Climate change significantly affecting species survival and sustainable use of biological resources.

On September 15, 2010, the State Council’s executive meeting reviewed and in principle approved the China National Biodiversity Conservation Strategy and Action Plan (2011–2030). This milestone event, following China’s accession to the Convention on Biological Diversity in 1992, elevated biodiversity protection to a national strategic level.

To further mobilize public participation in biodiversity conservation, the China Biodiversity Conservation and Green Development Foundation, together with the China Society for Strategy and Management Research, will jointly host a conference in Beijing on July 8 in response to the action plan.

2014.1.1-2016.12.31

1.

China Contributes to Global Biodiversity Conservation

The 13th Conference of the Parties (COP13) to the Convention on Biological Diversity (CBD) concluded on the 17th in Cancún, Mexico. At this conference, the organizers announced that China would host the 15th Conference of the Parties in 2020. This exciting news not only affirms China’s biodiversity conservation efforts but also highlights China’s determination to contribute to global biodiversity protection.

Mountains, rivers, forests, grasses, fish, insects, birds, and beasts — our Earth is vibrant and full of charm because of diverse ecosystems and abundant species resources. However, due to the overexploitation of biological resources by humans, global biodiversity is facing unprecedented challenges. Ecosystem degradation, species extinction, and the loss of genetic resources have already become worldwide problems.

Emma Gómez from Mexico’s National Genetic Resources Center remarked that, as one of the world’s 12 megadiverse countries, China is an important stronghold for biodiversity protection and is home to many unique species. China therefore bears greater pressure and responsibility in biodiversity conservation.

According to Li Junsheng, Deputy Director of the Institute of Environmental Ecology, Chinese Research Academy of Environmental Sciences, although conflicts between biodiversity protection and socioeconomic development remain prominent in China, the country has achieved positive results in four main areas: the basic formation of a conservation network, significant progress in ecosystem protection and restoration, deepened implementation of international conventions, and a marked increase in public awareness.

China signed the Convention on Biological Diversity on June 11, 1992, and ratified it on November 7, 1992, making it one of the earliest signatories. Since then, China has gradually developed a system of policies and regulations on biodiversity, with the promulgation of laws such as the Wildlife Protection Law, Environmental Protection Law, and Nature Reserve Regulations providing a legal foundation for biodiversity conservation and sustainable use.

2.

China Takes the Lead in Biodiversity Protection

The 13th Conference of the Parties to the Convention on Biological Diversity (CBD), hereafter referred to as the Cancún Conference, concluded in Mexico. At this conference, China successfully obtained the hosting rights for the 15th meeting in 2020, which will be the first time China hosts this event.

The CBD is an international convention dedicated to protecting Earth’s biological resources. Together with the UN Framework Convention on Climate Change (UNFCCC) and the UN Convention to Combat Desertification (UNCCD), it is regarded as one of the three major UN environmental conventions. “Protecting biodiversity means protecting our planet,” Erik Solheim, UN Under-Secretary-General and Executive Director of UNEP, told reporters.

At the opening ceremony, Mexican President Peña Nieto emphasized: “We must change our way of life to stop biodiversity loss, otherwise such loss will forever alter our lives.”

During the Cancún Conference, the Chinese delegation hosted a side event titled “China TEEB Actions and Local Practices.” TEEB (The Economics of Ecosystems and Biodiversity) is an international initiative launched by UNEP in 2008. By assessing the value of ecosystems and biodiversity, it helps society understand their importance and promotes the mainstreaming of biodiversity into national, sectoral, and local policies.

At this side event, experiences from Jingdong County, Pu’er City, Yunnan, won high recognition from international participants. Jingdong is China’s first TEEB demonstration county, home to one-third of the country’s species, making it one of the most biodiverse counties in China. County magistrate Hu Qiwu explained that in order to protect unique ecological resources, Pu’er and Jingdong have enacted strict regulations, established ecological redlines, and adopted a “one-vote veto system” on ecological performance for officials.

3.

International Day for Biological Diversity: A Look at Rare and Endangered Wildlife

May 22 is the International Day for Biological Diversity. Generally, biodiversity consists of three components: genetic diversity, species diversity, and ecosystem diversity. Among these, species diversity is the core. Currently, more than 2 million species are known to science, and it is this rich diversity of life forms that fills our planet with vitality.

However, species diversity worldwide is under severe threat. According to the IUCN Red List of Threatened Species, 593 species of birds, more than 400 species of mammals, 209 species of amphibians and reptiles, and more than 20,000 species of higher plants are now on the brink of extinction.

Under human interference, species extinction in the past century has occurred at a rate 1,000 times faster than natural extinction. Statistics show that about 75 species vanish worldwide every day, meaning roughly 3 species are lost forever every hour. The destruction of biodiversity will have profound impacts on Earth’s environment and cause immeasurable harm to the conditions for human survival and development. Countries around the world and many NGOs are making tireless efforts to conserve biodiversity.

4.

Why Do Western Media “Turn a Blind Eye” to the Biodiversity Conference?

From December 2 to 17, the 13th Conference of the Parties to the Convention on Biological Diversity was held in Cancún. Late one night near the end of the conference, after 11:00 p.m., the venue was still full of delegates from more than 190 countries and regions, debating every tiny detail of the convention.

In stark contrast, there was hardly a trace of Western media coverage. Even by the close of the meeting, many conference materials remained uncollected, and few visitors stopped at national exhibition booths. “You’re a journalist from China? At this point, there aren’t many reporters still here, so experts are happy to be interviewed,” one volunteer said with a smile, pointing the reporter toward a breakout room.

Back in 1992, the UN Conference on Environment and Development in Rio de Janeiro adopted two legally binding treaties: the UN Framework Convention on Climate Change (UNFCCC) and the Convention on Biological Diversity (CBD). In recent years, climate change and greenhouse gas emissions have dominated international attention. Annual UN climate conferences attract intense media focus, especially from Western outlets, with mainstream media fully present and reporters turning out in large numbers.

So why is it that the equally important UN biodiversity conferences receive little attention, with hardly any Western reporting available online?

According to Xu Jing, a biodiversity expert at the Chinese Research Academy of Environmental Sciences, while more than 190 parties have joined the CBD, the United States still has not. The reason is that joining the CBD would increase America’s international obligations, impose some restrictions on its biotechnology industry, and require sharing benefits from biodiversity resources with other countries — outcomes that clearly do not align with the interests of certain U.S. corporations.

5.

China’s Biodiversity Conservation Achievements: 2,729 Nature Reserves Established

It is an undeniable fact that China’s ecological diversity faces immense challenges. During the 13th Five-Year Plan period, as urbanization accelerates, forests, farmland, and other ecological spaces are expected to face further pressure.

China is one of the most biodiverse countries in the world, possessing all terrestrial ecosystem types, more than 35,000 species of higher plants (third worldwide), over 8,000 vertebrate species, and 28,000 recorded marine species. China’s genetic resources are also rich — it is the center of origin for rice, soybeans, and other major crops, and ranks first globally in cultivated plants and domesticated animals.

Specifically, China’s biodiversity conservation network is now largely in place. Terrestrial protected areas cover more than 1.7 million square kilometers, about 18% of the country’s land area, surpassing the CBD’s 2020 target of 17%. China has established 2,729 nature reserves with a total area of 1.47 million square kilometers, covering 14.8% of its land territory — higher than the global average of 12.7%. These reserves protect 85% of terrestrial ecosystem types and wild fauna and flora. By the end of 2014, China had 68 marine protected areas totaling 7,115 square kilometers, including 17 national-level reserves covering 5,089 square kilometers, as well as 58 nationally designated marine special protected areas (marine parks).

In addition, Qiu Qiwen noted that ecosystem protection and restoration have achieved remarkable results. Investments of 362.6 billion yuan were made in the Natural Forest Protection Program, effectively safeguarding about 1.05 million square kilometers of natural forests. Over the past decade, China’s forest area increased by 100,000 square kilometers, grassland vegetation cover in key ecological function zones rose by 11%, more than 2,800 square kilometers of mangroves and other degraded wetlands were restored, and 720,000 square kilometers of land were placed under soil erosion control and protection.

6.

Biodiversity “Barometer” Sounds the Alarm

Biodiversity refers to the ecological complex formed by animals, plants, microorganisms, and the environment, together with all associated ecological processes. To understand the state of biodiversity, one must assess the status of species. From this perspective, species status serves as a “barometer” of biodiversity.

It is estimated that there are between 50 and 100 million species worldwide, but only about 1.9 million have been scientifically identified and described. Moreover, species are unevenly distributed, with most concentrated in tropical and subtropical regions of a handful of countries. Twelve countries—including Brazil, Colombia, Ecuador, Peru, Mexico, the Democratic Republic of the Congo, Madagascar, Australia, and China—harbor roughly 60–70% of global species.

However, due to rapid economic development and population growth, human demands on nature are increasing, resulting in severe biodiversity degradation worldwide.

In addition, global warming is now an undeniable fact. Rising temperatures lead some plant and animal species to decline in population, shift their ranges, and suffer ecosystem degradation; some endangered species face inevitable extinction.

As an indicator measuring population trends of thousands of vertebrate species, the Living Planet Index (LPI) declined by 52% between 1970 and 2010. In other words, the number of mammals, birds, reptiles, and fish on Earth today is on average only about half of what it was 40 years ago.

Undoubtedly, global biodiversity is facing the most severe challenges in history.

7.

Ministry of Environmental Protection: Seven Major Biodiversity Conservation Projects to Be Implemented

China will implement seven major projects—covering in-situ conservation, ex-situ conservation, conservation linked with poverty alleviation, and others—focusing on priority areas for biodiversity protection and strengthening overall biodiversity efforts.

At a press briefing on China’s biodiversity conservation status, Qiu Qiwen explained that China is one of the most biodiverse countries in the world, with all terrestrial ecosystem types, more than 35,000 species of higher plants (ranking third globally), over 8,000 vertebrate species, and 28,000 recorded marine species. China’s genetic resources are also abundant, serving as the center of origin for major crops such as rice and soybeans, and ranking first globally in cultivated plants and domesticated animals.

“Rich biodiversity provides important support for maintaining regional ecological security and promoting sustainable social development,” Qiu said.

China was one of the first countries to join the Convention on Biological Diversity. It also established the world’s first National Committee on Biodiversity Conservation, coordinated nationwide biodiversity protection, and released and implemented both the China National Biodiversity Conservation Strategy and Action Plan (2011–2030) and the “China Action Plan for the UN Decade on Biodiversity.” Thanks to joint efforts from all regions, departments, and sectors of society, biodiversity conservation in China has achieved remarkable results.

At present, China has built a conservation network system with nature reserves as its backbone, supplemented by scenic areas, forest parks, and other types of protected sites. Terrestrial protected areas now cover more than 1.7 million square kilometers, about 18% of the country’s land area—exceeding the CBD’s 2020 target of 17%. A total of 2,729 nature reserves have been established, covering 1.47 million square kilometers (14.8% of land area), higher than the global average of 12.7%. About 85% of terrestrial ecosystems and wild species are now under effective protection.

8.

Biodiversity: Don’t Wait Until It’s Too Late

Plants and animals go extinct one after another, corn is the only crop left to survive, the Earth is engulfed in dust storms, and humanity faces doomsday… This is a scene from the science fiction film Interstellar. Could this day really arrive?

Scientific research suggests the outlook is grim.

Extinction Trends Continue

Once, mammoths, mastodons, moose, saber-toothed cats, jaguars, and many other large mammals thrived on this planet. But after modern humans spread across the globe, most of these animals disappeared forever. Sadly, recent studies show that the extinction trend among large mammals continues, while the survival of smaller species is also under threat.

According to Australia’s The Conversation, a paper published in Science Advances analyzed the survival status, threats, and ecological contributions of 74 of the world’s largest terrestrial herbivores (over 100 kg). It found that 60% of these species face the risk of extinction. These include iconic species such as elephants, hippos, rhinos, European bison, and water buffalo, as well as lesser-known species like the takin, banteng, various wild bovids of highland and lowland regions, and the Mindoro dwarf buffalo.

The disappearance of large mammals is only part of the picture. A study published last month in Nature revealed that over the past 500 years, humans have reduced the total biomass of wild terrestrial plants and animals by 10% and the total number of species by 14%, with most of these losses occurring in the past century.

This conclusion was based on analysis of more than 1 million biodiversity change records covering 27,000 species across 70 countries. The average global extinction rate of 14% masks stark differences: in some regions biodiversity has been relatively well preserved, while in others—such as Western Europe—20–30% of species have been lost.

9.

Cherish Biodiversity

Biodiversity is life; biodiversity is also our life.

May 22 is the United Nations’ International Day for Biological Diversity. The complexity and variety expressed in the forms, levels, and assemblages of life on Earth are the result of billions of years of evolutionary development, and they constitute the ecological foundation for humanity’s survival and continuation.

The “International Year of Biodiversity” launched in Berlin in 2010 had a thought-provoking theme: “Biodiversity is life; biodiversity is our life.” This theme fundamentally inspires us to reflect on the entirety of human history.

When thinking about history, people often focus on dramatic, story-like events and processes centered on individuals, which then become the content of social, political, intellectual, and cultural histories. Yet in terms of humanity’s two million years of existence, the true foundation has not been those events, but rather the evolving relationship between humans, the societies they have built, and the natural environment and ecosystems in which they exist, along with the consequences of that evolution.

At this level, the famous rulers, heroes, philosophers, and villains who dominate our history books seem small and powerless, their roles insignificant. Compared with the slow current of this deeper historical river, their dramatic performances are but fleeting ripples on the surface.

10.

Biodiversity Flourishes in China’s Ecologically Fragile Areas

Recently, Qinghai Province released an explanation of its “Ecological Redline Construction Plan.” Under this policy framework, the government will delineate ecological redlines focusing on key ecological function zones and using nature reserves as the foundation, in order to strictly protect important natural ecological spaces and their ecosystems.

As an important ecological security barrier for the nation, Qinghai’s redline designation will further strengthen the protection and management of nature reserves and promote biodiversity conservation on the Qinghai–Tibet Plateau.

Qinghai is one of China’s key biodiversity reservoirs and the source of three major Asian rivers—the Yellow River, the Yangtze River, and the Mekong River—earning it the title “Water Tower of China and Asia.”

Since the 1970s, combined impacts of climate change and overgrazing have led to rapid shrinkage of grasslands and forests in the Sanjiangyuan (Three-River Source) region, with wetlands and water bodies reduced by 375 square kilometers—equivalent to 58 West Lakes.

“Less water, degraded grasslands, stronger sandstorms, rising rodent damage, and an imbalanced ecosystem all threaten biodiversity,” said Li Ruofan, Director of the Sanjiangyuan National Nature Reserve Administration.

In 2005, the Sanjiangyuan Nature Reserve launched China’s largest ecological “Project of the Century” — the Sanjiangyuan Ecological Protection and Construction Project.

Since the establishment of Dinghushan Nature Reserve in Guangdong in 1956, China’s first, the country has created 2,740 nature reserves covering 1.47 million square kilometers. Representative reserves now protect more than 90% of terrestrial ecosystems and 89% of nationally protected wildlife species.

2017.1.1-2019.12.31

1.

Severe Global Alien Species Invasion Threatens Biodiversity

The Intergovernmental Science-Policy Platform on Biodiversity and Ecosystem Services (IPBES) plans to prepare a report that will provide a comprehensive overview of the world’s most threatening invasive alien species. Currently, at least one-quarter of the 100 most dangerous invasive species worldwide have already spread into Chile.

According to reports, IPBES is an independent body established by the United Nations Environment Programme. More than 70 researchers from around the world will collaborate over the next three years, conducting rigorous reviews of real-world conditions across all regions, including areas inhabited by Indigenous peoples, to compile this report. Researcher Aníbal Pauchard is one of the lead authors.

Pauchard said: “We will engage with Indigenous communities to collect more information. In addition to climate change, invasive species are threatening the planet’s biodiversity.”

He pointed out that invasive alien species also damage human quality of life. “For example, mosquitoes—most of which are invasive—become disease vectors thousands of kilometers away from their native habitats. Or invasive weeds, which may trigger wildfires.” The report aims not only to explain the biological damage caused by invasive species, but also to examine their impacts on human life quality, thereby providing information to support public policy for protecting people, flora, and fauna.

Meanwhile, the report notes that Chile hosts at least 25 of the world’s top 100 invasive species, including water hyacinth, Argentine ants, goats, Mediterranean mussels, black rats, rainbow trout, wild boar, red-eared sliders, and domestic cats.

2.

Experts at Home and Abroad Offer Advice on Global Biodiversity Conservation

At the Biodiversity Conservation Sub-Forum of the First National Park Forum in Xining, Qinghai, Chinese and international experts offered suggestions for global biodiversity protection, focusing on transboundary conservation, models, and management methods.

“Biodiversity in nature provides vital services and underpins modern society. Yet nature and biodiversity are disappearing at an alarming rate,” said Zhou Fei, Deputy Chief Representative of WWF Beijing Office. “We must set higher goals to reverse the curve of global biodiversity loss.”

According to WWF’s Living Planet Report 2018, populations of fish, birds, mammals, amphibians, and reptiles worldwide declined on average by 60% between 1970 and 2014, with extinction rates accelerating.

“Promoting transboundary conservation can reconnect fragmented habitats, maintain genetic exchange among species, and is crucial for biodiversity protection,” said Zhang Xiwu, Deputy Director of Beijing Normal University’s National Park Research Institute. He suggested that global biodiversity conservation requires improved cooperation mechanisms and joint actions to achieve holistic protection of species and habitats.

China is one of the most biodiverse countries in the world, with forests, shrublands, meadows, grasslands, deserts, and wetlands covering its landmass. It ranks third in higher plant diversity, hosts 13.7% of the world’s vertebrate species, and accounts for 14% of known fungi.

“Transformative change is needed for biodiversity conservation. Goals for 2030 or beyond may only be achieved through revolutionary changes in economic, social, political, and technological spheres,” said Yang Rui, Dean of Tsinghua University’s National Park Research Institute. “Such change requires a fundamental reorganization of technological, economic, and social factors, including paradigms, goals, and values.”

3.

Chinese and African Scholars Call for Joint Protection of Africa’s Biodiversity

The “ANSO International Conference on Biodiversity of Arid and Wetland Areas” opened on the 4th at Maasai Mara University in Kenya, hosted by the Alliance of International Science Organizations (ANSO) under the Belt and Road Initiative. More than 100 experts and scholars from over 10 countries, including China, Kenya, and South Africa, called for joint efforts to address the challenges to biodiversity protection and sustainable development in Africa posed by human activity and climate change.

At the opening ceremony, Bai Chunli, President of the Chinese Academy of Sciences, stated that biodiversity in arid zones and wetlands is key to Africa’s sustainable development, essential for food security, water supply, and climate change mitigation. He emphasized that scientific and technological innovation is critical to halting biodiversity degradation. The conference aims to create a platform for China-Africa academic institutions to share biodiversity conservation experiences and jointly confront challenges.

“Biodiversity is vital for human survival, a key resource for sustainable socioeconomic development, and an important field of cooperation among ANSO members,” Bai said. He noted that this was ANSO’s first academic meeting held outside China, with biodiversity and sustainable development as its theme.

Felix Dakora, President of the African Academy of Sciences, remarked that while biodiversity is one of Africa’s greatest strengths, it is now seriously affected by climate change, desertification, and water pollution. He called the conference a valuable opportunity to discuss responses to these challenges.

Britia Godrick, Vice-Chancellor of Maasai Mara University, added that during the three-day event, scholars, policymakers, think tank experts, and business leaders would engage in scientific discussions on innovative measures to combat ecosystem degradation.

4.

CBD Executive Secretary: Yunnan Will Showcase Biodiversity to the World

The 15th Conference of the Parties (COP15) to the Convention on Biological Diversity (CBD) will be held in Kunming, Yunnan, in 2020. On the 5th, CBD Executive Secretary Elizabeth Maruma Mrema visited Kunming to guide preparations.

Mrema noted that humanity and nature are currently facing major challenges such as biodiversity loss, climate change, and marine plastic pollution. “The theme of next year’s conference is ‘Ecological Civilization: Building a Shared Future for All Life on Earth.’ Through this theme, we hope to inspire people to change their behavior and lifestyles, advocate ecological civilization, and promote harmony between humans and nature.”

“China has proposed the concept of green development, harmony between humans and nature, and has made commendable achievements in biodiversity conservation and ecological civilization,” she added. “At next year’s conference, we hope China will share its experiences with the world, while also learning from other countries.”

Yunnan, the host province, is one of the world’s 34 biodiversity hotspots and ranks first in China for biodiversity resources. Currently, Yunnan is striving to be a leader in ecological civilization, and this year became the first province in China to enact local biodiversity conservation legislation.

Having visited Yunnan twice, Mrema said she was impressed by the province’s biodiversity richness: “Here I see that economic development and environmental protection are not in conflict. Yunnan’s policies demonstrate that both can proceed hand in hand.”

She explained that in October next year, representatives from 196 CBD parties will gather in Kunming to negotiate the “Post-2020 Global Biodiversity Framework” and set new biodiversity targets for 2030. She expressed confidence that delegates would leave with a deep impression of Yunnan’s biodiversity and conservation measures.

5.

China’s Biodiversity Protection Efforts Gain Attention at Paris Peace Forum

During the second Paris Peace Forum, China’s Ministry of Ecology and Environment hosted a roundtable on November 12 titled “CBD COP15 — Biodiversity’s Last Chance,” attracting over 200 participants. China’s biodiversity conservation efforts received broad international attention.

The 15th Conference of the Parties to the Convention on Biological Diversity (COP15) will be held in Kunming, China, in 2020. Participants in the roundtable included Huang Runqiu, Director of the COP15 Executive Committee and Vice Minister of China’s Ministry of Ecology and Environment; former French Prime Minister Jean-Pierre Raffarin; and Brune Poirson, Secretary of State to the French Minister for the Ecological and Inclusive Transition. Discussions focused on conference preparations and the development and implementation of the Post-2020 Global Biodiversity Framework.

Huang Runqiu emphasized that COP15 is a milestone in the CBD’s implementation process. It will not only chart a new blueprint for biodiversity conservation in the next decade but also strengthen the commitment of stakeholders to framework implementation. As host country, China is ready to work closely with the CBD Secretariat, fully consider the concerns of all parties, coordinate diverse interests, and strive for an ambitious yet realistic framework.

Raffarin praised the organization and preparations for COP15, stating that ecological protection requires both innovation and stronger environmental education for younger generations. He commended China’s strong emphasis on ecological civilization and its active role in global biodiversity conservation.

Poirson remarked that China has demonstrated strong leadership in preparing for COP15, mobilizing society-wide efforts to advance biodiversity protection. She added that France looks forward to working with China to ensure the conference’s success.

6.

Yunnan Biodiversity Photo and Eco-Environment Cartoon Exhibition Opens in Kunming

On the morning of November 17, the Yunnan Provincial Department of Ecology and Environment launched the Yunnan Biodiversity Photo and Eco-Environment Cartoon Exhibition at the Kunming Museum, offering citizens a comprehensive showcase of the province’s rich biodiversity and the beauty of its ecological environment.

Attendees at the opening ceremony included Gao Zhengwen, Deputy Director-General of the Department and a member of its Party Leadership Group, along with officials from relevant divisions and affiliated institutions, leaders from the Kunming Museum, the Fine Arts School of Yunnan Arts University, the Yunnan Plateau Ecological Environmental Protection Foundation, the Yunnan Ethnic Culture and Arts Promotion Association, as well as representatives of students and teachers from Fengyuan Primary School in Kunming, artists, exhibitors, provincial green schools and communities, university student organizations, and members of the press. The ceremony was hosted by Chen Li, Director of the Department’s Publicity and Education Division.

Biodiversity is a key part of building an ecological civilization and forms the foundation for human survival and development. Yunnan, as one of the most biodiverse and ecologically important regions in China, is both a major biodiversity reservoir and a southwest ecological security barrier. In 2020, Yunnan’s capital Kunming will host the 15th Conference of the Parties (COP15) to the UN Convention on Biological Diversity (CBD), a landmark event in the history of ecological civilization for both Yunnan and China. The province aims to use this exhibition to promote ecological culture and showcase the beauty of Yunnan’s environment.

Deputy Director-General Gao emphasized that the purpose of hosting the exhibition is to thoroughly promote Xi Jinping’s thought on ecological civilization, highlight Yunnan’s achievements in environmental protection, spread biodiversity knowledge, raise public awareness, and create strong publicity momentum for the upcoming COP15 in Kunming.

7.

Earth Hour | Tsinghua Professor: Live in Harmony with Nature, Protect Urban Biodiversity

Starting with Crows

For many Beijing residents, each autumn and winter evening brings the spectacular sight of dense flocks of crows gathering in treetops. Behind this spectacle lies a combination of factors.

Crows are species that prefer tall trees, which give them vantage points to watch for danger. As urban expansion has led to the felling of surrounding forests, crows have been forced to find new habitats.

Beijing winters are bitterly cold. Due to the “urban heat island effect,” crows forage in suburban garbage dumps by day but fly back into the city at night to find warmth and rest. Old residential areas and university campuses with large trees and quiet surroundings have become their favored havens.

Crows are not the only species adapting to city life. Sparrows, swallows, magpies, bats, geckos, and a wide range of plants and insects also thrive in urban environments.

A Peking University survey recorded 178 bird species within its campus, including 21 under Class II or higher national protection. Similarly, Tsinghua University’s Urban Ecology Research Group documented 2,640 woody plant species across 257 Chinese cities, including 1,671 trees, 743 shrubs, and 226 woody vines.

Clearly, cities harbor a rich variety of life. These organisms are the “small surprises” that enliven our busy urban lives.

8.

Biodiversity Richness in the Eastern Sanjiangyuan Region

On October 19, the Shan Shui Conservation Center released interim findings from a biodiversity survey in the Mako River forest area in the eastern Sanjiangyuan region. Over nearly a year of monitoring in Banma County, Guoluo Tibetan Autonomous Prefecture, Qinghai Province, researchers using infrared cameras recorded multiple wildlife species including snow leopards, brown bears, musk deer, Tibetan macaques, and blood pheasants—demonstrating the area’s rich biodiversity.

Located at the junction of Qinghai, Gansu, and Sichuan provinces, the Mako River forest area hosted 60 infrared cameras operating for 4,500 camera-days, capturing images of 18 mammal species and 9 bird species.

The survey confirmed that the Mako River forest is Qinghai’s richest known area for ungulates, with abundant populations of tufted deer, musk deer, Chinese serows, and gorals, all maintaining healthy numbers.

The project was jointly conducted by Mako River Forest Station, the Nianbaoyuze Conservation Association, the Sanjiangyuan Conservation Foundation, and the Shan Shui Conservation Center.

9.

Chinese and Foreign Prosecutors Discuss Judicial Protection of Biodiversity in Kunming

Kunming, November 18 (China News Service, Reporter Hu Yuanhang) — The International Symposium on Judicial Protection of Biodiversity opened in Kunming, bringing together prosecutors and scholars from China, Brazil, the UK, Australia, the Netherlands, and other countries to discuss legal and practical issues in biodiversity-related judicial protection.

The event was co-hosted by the Eighth Procuratorial Office of China’s Supreme People’s Procuratorate and the European Environmental Bureau. Topics included judicial protection of nature reserves, judicial safeguards for biodiversity and public participation, and prevention and remediation of environmental damage.

Zhang Xueqiao, Deputy Procurator-General of the Supreme People’s Procuratorate, emphasized in his speech that as one of the earliest parties to the CBD, China attaches great importance to biodiversity conservation. In 2017, China formally established its public interest litigation system, creating a unique “China model” for judicial protection of the public interest. This has produced positive results in promoting law-based governance and supporting integrated management of environmental and resource issues.

Between July 2017 and September 2019, Chinese procuratorial bodies filed 118,012 public interest cases related to ecological and resource protection, accounting for 54.96% of all such filings. These cases led to the remediation of 2.14 million hectares of farmland, wetlands, forests, and grasslands; the removal of 31.04 million tons of solid waste and household garbage; and the recovery of 3.45 billion yuan in ecological restoration and environmental management costs.

“Nonetheless, China still faces severe biodiversity challenges. The overall decline has not been fundamentally curbed, and in some regions, the balance between biodiversity protection and economic development requires more scientific management,” Zhang said. He expressed hope that the symposium would foster collective wisdom and consensus, further develop China’s public interest litigation system, and promote international exchange and cooperation in biodiversity-related judicial protection.

10.

One Million Species Face Extinction, UN Study Warns Humanity to Protect Biodiversity

If no action is taken, the global rate of species extinction will accelerate to tens or hundreds of times higher than the average over the past 10 million years.

A new UN study warns that one million species are currently on the brink of extinction. The inability to maintain biodiversity will have serious consequences for Earth and human life, yet concrete solutions remain elusive.

On May 6, UNESCO Director-General Audrey Azoulay released the Global Assessment Report on Biodiversity and Ecosystem Services in Paris. This was the first comprehensive UN report on global biodiversity since 2005.

The report states that human activities now threaten more species than ever before. At least 25% of plant and animal species—about one million in total—are at risk of extinction, many within decades, unless humans act to mitigate drivers of biodiversity loss.

Over the past 50 years, five major drivers have altered biodiversity and ecosystems: changes in land and sea use, direct exploitation of organisms, climate change, pollution, and invasive alien species.

The report warns that without action, extinction rates will rise dramatically, reaching tens to hundreds of times above long-term averages. Azoulay stressed that protecting biodiversity—at the genetic, species, and ecosystem levels—should be treated with the same urgency as combating climate change. “With the adoption of this historic report, no one can now claim ignorance,” she said.

2020.1.1-2022.11.1

1.

Encouraging Progress in China’s Biodiversity Conservation: From “Smiling Angels” to “Tens of Thousands of Antelopes Running Together”

Recent biodiversity stories have become hot topics: the northward “tour” of Yunnan’s wild elephants, the giant panda’s threat status downgraded from “endangered” to “vulnerable,” the frequent appearance of the “smiling angel” Yangtze finless porpoise, repeated sightings of snow leopards in Sanjiangyuan National Park, and the recovery of Tibetan antelope populations on the Qinghai–Tibet Plateau from 70,000 to 300,000, recreating the spectacular scene of “tens of thousands of antelopes running together.”

At a press conference on August 18, Minister of Ecology and Environment Huang Runqiu stated that these heartening developments demonstrate real achievements in China’s biodiversity conservation. He attributed the progress to three main factors: significantly increased public awareness, the gradual improvement of the protected area system, and strengthened supervision and law enforcement.

Huang noted that since the 18th National Congress of the Communist Party of China, awareness of ecological and biodiversity protection has grown steadily, with responsibilities clearly assigned and the idea of harmony between humans and nature becoming a broad consensus. Local governments and departments have taken effective measures to guide and support conservation. “On the Qinghai–Tibet Plateau, ecological compensation programs have turned farmers and herders from exploiters of nature into guardians—working as wildlife, forest, and wetland rangers, becoming the ‘ecological guardians of the plateau.’ Since 2016, Tibet and Qinghai have provided over 900,000 ecological jobs, increasing incomes of farmers and herders by nearly 8 billion yuan.”

2.

“Protecting Biodiversity, Building a Shared Future for All Life on Earth” — China in Action

The 15th Conference of the Parties (COP15) to the Convention on Biological Diversity (CBD) was held in two phases in Kunming, China: October 11–15, 2021, and the first half of 2022. With the theme “Ecological Civilization: Building a Shared Future for All Life on Earth”, the conference aimed to establish the Post-2020 Global Biodiversity Framework and set new conservation targets.

On September 30, 2020, President Xi Jinping delivered a keynote speech at the UN Biodiversity Summit, stressing: “The accelerated loss of biodiversity and ecosystem degradation pose major risks to human survival and development. The COVID-19 pandemic has reminded us that humanity and nature are a community of shared destiny. We must unite and act swiftly: protect while developing, and develop while protecting, to build a beautiful homeland where all life coexists in harmony.”

China has attached great importance to biodiversity protection, adopting a series of measures that have produced remarkable results and promoted coexistence between humans and nature.

The northward journey of Yunnan’s elephants, the giant panda’s reclassification to “vulnerable,” and the frequent appearances of Yangtze finless porpoises are among the positive news stories that have attracted attention in recent years.

China is one of the most biodiverse countries in the world: it hosts about 35,000 higher plant species (10% of the global total, ranking third worldwide) and 686 mammal species, with the highest rate of endemism globally. It is also rich in crop and forest genetic resources. Yet due to habitat loss, fragmentation, overexploitation, pollution, and climate change, China is also among the countries most seriously threatened by biodiversity decline. For example, the China Red List of Biodiversity assessed 34,450 higher plant species, of which 3,767 (10.9%) are threatened.

3.

Global Biodiversity Faces Multiple Threats

According to a recent UPI report, a new study shows that the extinction risk facing Earth’s species may be far more severe than previously thought. A past biodiversity survey found that since 1500, about 30% of global species have been threatened or driven to extinction.

The new study, published this week in Frontiers in Ecology and the Environment, surveyed a “large and diverse” group of biodiversity experts worldwide, covering all major taxonomic groups and habitats across freshwater, terrestrial, and marine ecosystems.

A total of 3,331 biodiversity experts from 113 countries were asked to estimate past and future global biodiversity loss and to rank the factors driving species endangerment and extinction.

The study, led by Forrest Isbell, Associate Professor at the University of Minnesota, compared these expert assessments with other sources of information. The researchers stressed the importance of this work, noting: “Decision-makers often rely on expert judgment to fill critical knowledge gaps.”

The report stated: “Expert judgment provides estimates and forecasts for key uncertainties in areas such as nuclear safety, volcanic eruptions, climate change, and biodiversity loss. The most accurate assessments come from large and diverse groups of experts, partly because expertise declines sharply outside one’s specialization.”

4.

Global Biodiversity Conservation Looks Toward a New Chapter

The 7th World Conservation Congress concluded on September 10 in Marseille, France. During the congress, the International Union for Conservation of Nature (IUCN) updated its Red List of Threatened Species, highlighting the state of biodiversity loss and the close ties between biodiversity protection, human well-being, and socioeconomic development.

Observers noted that one of the congress’s key aims was to alert the international community to species under threat and the accelerating extinction rate. Looking ahead, the world anticipates that the upcoming COP15 in Kunming, China, will adopt the Post-2020 Global Biodiversity Framework and launch a new chapter in global biodiversity protection.

Biodiversity Crisis Deepens

The updated IUCN Red List assessed the extinction risk of 138,374 species worldwide, finding that 38,543 are threatened with extinction. Craig Hilton-Taylor, head of the Red List Unit, warned that species are disappearing at an accelerating pace: “The trend is 100 to 1,000 times higher than natural extinction rates. Humanity is approaching a sixth mass extinction, and if extinctions continue at this speed, we will soon face a major crisis.”

IUCN President Zhang Xinsheng added that humanity’s survival depends on Earth and “Mother Nature,” which now face three great crises: pollution, climate change, and biodiversity loss combined with ecosystem degradation. He emphasized that biodiversity loss and ecological decline are approaching critical red lines.

5.

The “China Moment” Opens a New Chapter in Global Biodiversity Protection

On the afternoon of October 11, the first phase of the 15th Conference of the Parties to the Convention on Biological Diversity (CBD COP15) opened in Kunming, Yunnan Province, with traditional ethnic song and dance performances at the ceremony.

The conference, themed “Ecological Civilization: Building a Shared Future for All Life on Earth,” was the first UN global meeting to adopt ecological civilization as its theme. Over 1,800 representatives attended, including delegates from CBD parties, international organizations, financial and research institutions, NGOs, businesses, media outlets, and Chinese local governments.

At the opening ceremony, Huang Runqiu, China’s Minister of Ecology and Environment, received the COP15 gavel from Egypt’s Environment Minister and COP14 President Yasmine Fouad. After three years, the “China Moment” in global biodiversity conservation had arrived.

In the coming COP15 sessions, a new global biodiversity framework for the next decade will be finalized. With around one million species worldwide at risk of extinction and biodiversity under unprecedented pressure, all parties look to COP15 to open a new chapter for global conservation.

“COP15 will be a historic milestone,” said Elizabeth Mrema, Executive Secretary of the CBD Secretariat. “We are at a critical moment. To realize the 2050 Vision of Living in Harmony with Nature, action must be taken in this decade to halt and reverse biodiversity loss, with biodiversity on a path to recovery no later than 2030.”

Entrusted with the mission of advancing global sustainable development, COP15 carries the responsibility of leading the future of biodiversity conservation. As host country, China is widely expected to play a vital role in driving consensus toward a balanced, ambitious, and practical Post-2020 Global Biodiversity Framework.

6.

Rare Wildlife is “Returning”: Tangible Achievements in China’s Biodiversity Conservation

Beijing, July 7 (Xinhua, Reporter Wang Libin) — From the south to the north, from inland to coastal regions, rare wildlife species that had disappeared for many years are now “returning,” reflecting solid achievements in China’s biodiversity conservation.

This was revealed at the press conference of the 2021 Eco Forum Global Guiyang International Forum. Cui Shuhong, Director of the Department of Nature and Ecology Conservation of the Ministry of Ecology and Environment, said that recently there has been much biodiversity-related news. For example, desert cats — one of the world’s most mysterious felines and a first-class protected species in China — were filmed in the Qilian Mountains Nature Reserve; three brown-necked hornbills were spotted in the forests of Yunnan at elevations above 2,000 meters; Siberian tigers entered villages in Heilongjiang; Asian elephants migrated northward in Yunnan, attracting nationwide attention; and whales were recently seen again in Shenzhen’s Dapeng Bay. Wild giant pandas, snow leopards, and other rare and endangered animals have been frequently sighted. These events, to some extent, highlight the achievements in biodiversity conservation and ecological restoration in China.

As part of ecological civilization construction, biodiversity protection has been progressively integrated into national plans such as the China Biodiversity Conservation Strategy and Action Plan (2011–2030), with comprehensive deployment for strengthening ecosystem and biodiversity conservation and restoration. By the end of 2019, China had established 11,800 nature reserves of various types, covering more than 170 million hectares, or 18% of its land area—achieving ahead of schedule the Aichi Target of 17% land protection by 2020 under the UN Convention on Biological Diversity. China has also established botanical gardens and wildlife breeding bases, successfully breeding large numbers of rare and endangered species. Significant progress has been made in ecosystem conservation and restoration.

7.

Ministry of Ecology and Environment Holds January Press Conference, Focuses on Biodiversity Conservation

China Environment News, Beijing, January 28 — The Ministry of Ecology and Environment (MEE) held its regular monthly press conference today. Cui Shuhong, Director of the Department of Nature and Ecology Conservation, attended and introduced biodiversity protection progress. Liu Youbin, spokesperson of the Ministry, presided over the conference, reported recent key environmental protection work, and answered questions from the media.

Liu Youbin noted that in 2020, China’s ecological protection system thoroughly implemented Xi Jinping’s Thought on Ecological Civilization and carried out the decisions of the CPC Central Committee and the State Council. The phased goals of the battle against pollution were successfully achieved, and the quality of the ecological environment continued to improve. During the 13th Five-Year Plan period, ecological and environmental protection reached historic achievements, with all nine binding ecological indicators in the plan being exceeded.

The MEE will continue to apply the new development philosophy, systematically plan ecological and environmental protection during the 14th Five-Year Plan period, and develop the action plan for peaking carbon emissions before 2030. It will strengthen pollution prevention and control, ecological conservation and restoration, nuclear and radiation safety, and law-based ecological inspection and enforcement, while preventing and resolving environmental risks. China has completed the reform of the solid waste import management system and banned all imports of solid waste as of January 1, 2021, having reduced imports by 100 million tonnes from 2017 to 2020.

The MEE also issued new guidelines for the content, format, and technical standards for Environmental Impact Report Forms for Construction Projects, to be implemented from April 1, 2021. The theme for China’s 2021 Environment Day was set as “Living in Harmony with Nature” to raise public awareness of biodiversity conservation and strengthen the philosophy of respecting, adapting to, and protecting nature, thereby building a beautiful homeland where humans and nature coexist harmoniously.

8.

News Weekly: Protecting Biodiversity — We Are Taking Action

May 22 is the International Day for Biological Diversity. The 2020 theme was “Our Biodiversity, Our Food, Our Health,” emphasizing the dependence of food, nutrition, and health on biodiversity and healthy ecosystems.

Earth’s rich variety of life is the product of billions of years of evolution. From animals and plants to microbes, every step of human history has been intertwined with these species, evolving together. Yet many species once accompanying humankind are now fading away, disappearing into history.

A recent UN report shows that around one million species of plants and animals worldwide are threatened with extinction, many of which could vanish within decades. Research indicates that human activities are the main driver: population growth has accelerated consumption and exploitation of natural resources, while deforestation, pesticide abuse, overfishing, smuggling, and pollution have severely disrupted ecosystems, leaving many species without habitats.

China was among the first to ratify the Convention on Biological Diversity. Through programs such as natural forest protection, returning farmland to forest and grassland, wildlife protection, and establishing nature reserves, as well as advancing a national park system, vegetation cover has been restored, providing living conditions for wild plants and animals.

However, ecological restoration requires time, and efforts must be further strengthened. This includes coordinated protection and restoration of forests, grasslands, wetlands, deserts, and wildlife, as well as stronger participation in international or regional joint law enforcement against wildlife crimes.

Fundamentally, biodiversity conservation requires transforming resource use methods and improving efficiency. Everyone must adopt the concept of conservation, intensive, and circular resource use, focusing on system efficiency and minimizing damage to ecosystems and biodiversity during development and resource utilization.

9.

“The Sixth Mass Extinction?” UN Calls for Action to Save Biodiversity

According to The Guardian, the destruction of coral reefs, tropical rainforests, and other critical ecosystems has placed human society at risk. Scientists warn that without fundamental action, dire consequences such as freshwater shortages and climate instability will follow.

As The Huffington Post noted, biodiversity loss is closely intertwined with climate change, and together they pose an existential threat to human civilization. International environmental law expert John Knox previously wrote that biodiversity loss has “serious and far-reaching impacts” on human well-being, including reduced fisheries and agricultural yields, depletion of medicinal resources, and increased infectious diseases.

In response to the crisis, the international community is taking action. A draft plan released on Monday stated that to protect remaining wildlife, by 2030 nearly one-third of Earth’s surface must be protected, and pollution must be reduced by at least half.

“Biodiversity and the benefits it provides are the foundation of human well-being and a healthy planet,” the draft stated. “Despite our efforts, global biodiversity continues to decline, and if current trends persist, this decline is expected to continue or worsen.”

The Post-2020 Global Biodiversity Framework aims to stabilize biodiversity loss by 2030 and restore ecosystems by 2050, with the ultimate vision of “living in harmony with nature.” But achieving these goals requires urgent action at both local and global levels.

The draft listed 20 action targets for the next decade, including reducing carbon emissions and ensuring food sustainability. Some goals emphasize human well-being, such as providing vulnerable communities with better food security and clean resources to reduce “human–wildlife conflicts.”

10.

Encouraging Progress in China’s Biodiversity Conservation: From “Smiling Angels” to “Tens of Thousands of Antelopes Running Together”

Recent biodiversity news has become widely discussed: the northward “tour” of Yunnan’s wild elephants, the downgrading of the giant panda’s threat status from “endangered” to “vulnerable,” the frequent appearance of the “smiling angel” Yangtze finless porpoise, repeated sightings of snow leopards in Sanjiangyuan National Park, and the recovery of Tibetan antelope populations on the Qinghai–Tibet Plateau from 70,000 to 300,000, recreating the spectacular scene of “tens of thousands of antelopes running together.”

At an August 18 press conference, Minister of Ecology and Environment Huang Runqiu said these heartwarming events prove that China’s biodiversity conservation has achieved real results. He attributed the success to three factors: rising public awareness, improvements in the protected area system, and strengthened law enforcement.

Huang noted that since the 18th National Congress of the CPC, awareness of ecological and biodiversity protection has steadily increased, responsibilities have been clarified, and the concept of harmony between humans and nature has become widespread. Governments at all levels have taken effective measures to encourage public participation.

“For example, through ecological compensation programs on the Qinghai–Tibet Plateau, farmers and herders are no longer exploiters of nature. Instead, they earn livelihoods as wildlife, forest, and wetland rangers — becoming ‘ecological guardians of the plateau.’ Since 2016, Tibet and Qinghai have created over 900,000 ecological jobs, increasing rural incomes by nearly 8 billion yuan.”

**Biodiversity search volume peak news**

1. 2018/04/16-2018/04/22

1.1

Strengthening Scientific and Technological Support to Safeguard Biodiversity

Recently, a video of a wild brown giant panda circulated widely online. It was captured by an infrared camera installed at a monitoring site in Changqing National Nature Reserve, Shaanxi Province, as part of the biodiversity observation network established by the Ministry of Ecology and Environment. To date, all scientifically documented sightings of brown pandas have been in the core area of the Qinling Mountains in Shaanxi. This rare glimpse is a reflection of the richness of China’s biodiversity.

Biodiversity is closely linked to everyone’s daily life—food, water, medicine, timber, energy, and other necessities all come from biodiversity, while ecological services such as carbon sequestration, oxygen release, water conservation, soil retention, and eco-tourism also depend on it.

Biodiversity is both a condition for human survival and a material foundation for sustainable economic and social development. It is also a vital component of building ecological civilization and a “Beautiful China.” On September 17, 2010, the State Council approved and issued the China Biodiversity Conservation Strategy and Action Plan (2011–2030), which, together with related national plans, forms the top-level design of national biodiversity conservation. In 2011, the “China National Committee for Biodiversity Conservation” was established, comprising 25 ministries and led by a Vice Premier, to coordinate biodiversity conservation nationwide.

Over the years, China has made remarkable progress in biodiversity conservation, though challenges remain. The overall decline in biodiversity has not been fundamentally curbed, manifested in the degradation of ecosystem functions, increased threats to species, and the loss of genetic resources. The second national survey of livestock and poultry genetic resources revealed that 15 local breeds are no longer found, and more than half of the local breeds are declining in population size.

1.2

Hainan Biodiversity Research Center Cooperation Base Established

Haikou, April 18 (Reporter Yao Shaolong, Nanhai Net) — Recently, Hainan Tropical Wildlife Park signed a memorandum of understanding with the Zoological Society of London (ZSL) to establish the Hainan Biodiversity Research Center Cooperation Base and invited ZSL technical experts to join.

The cooperation base will provide a valuable platform for wildlife protection, science education, and related efforts in Hainan. At the launch ceremony, the center appointed Dr. Samuel Turvey (postdoctoral researcher and senior fellow, ZSL Institute of Zoology), primatologist Dr. Carolyn Thompson, and Dr. Ma Tianjiao as technical advisors, presenting them with appointment letters. After completing relevant national procedures, they will begin work at the base.

Both sides also discussed cooperation in building conservation capacity, monitoring, research, habitat restoration and management, public science education, and conservation planning for endangered and vulnerable species in Hainan.

About ZSL

The Zoological Society of London (ZSL), founded in April 1826, is a scholarly institution that has developed into an international charity for research, conservation, and education focused on animals and their habitats. ZSL manages the London Zoo and Whipsnade Zoo, collaborates with many European zoos, and published Zoological Record, a major international zoological literature index, from 1864 to 1980.

1.3

What is the Highest-Flying Bird? What Native Insects Live in Shanghai? “Biodiversity Activities” at Shanghai Zoo Await You

Starting today, the Shanghai Zoo launches its 2018 biodiversity program as part of Earth Day activities. Events include the Save the Lesser White-fronted Goose campaign, a global marine mollusk exhibition, a primate diversity series, a showcase of native insects of Shanghai, themed lectures, nature classes, and more.

Where did the concept of biodiversity come from? Biodiversity refers to the variety of life on Earth—including animals, plants, and microorganisms—in all forms, levels, and assemblages. It encompasses ecosystem diversity, species diversity, and genetic diversity. On December 20, 2000, the UN General Assembly adopted a resolution designating May 22 each year as the International Day for Biological Diversity, to raise global understanding and awareness.

It is estimated that Earth has between 3 and 10 million species, of which only about 1.5 million have been formally described. However, human activities and unsustainable patterns of production and consumption are destroying biodiversity and threatening ecosystems. Overexploitation of biological resources, soil degradation, loss of forests, and disasters linked to climate change are causing species to disappear at an alarming rate. The rapid decline of biodiversity will bring severe consequences to humanity. Thus, protecting Earth’s biodiversity is, in essence, protecting ourselves.

1.4

Lijiang Forest Ecosystem Research Station: Safeguarding Northwestern Yunnan’s Biodiversity

Northwestern Yunnan is one of the most biodiverse regions in the world due to its unique geology, complex terrain, and diverse climate. The Lijiang Forest Ecosystem Research Station of the Kunming Institute of Botany, Chinese Academy of Sciences, is located at the core of this area.

Xu Kun, senior engineer and head of the station, explained to China Science Daily: “Our station focuses on collecting wild plant resources and building living collections, supporting sustainable use of wild plant resources through research and technology development, and conducting long-term biodiversity monitoring in cooperation with sample plot networks.”

Preserving Living Plant Germplasm

The station is part of the core zone of the Lijiang Alpine Botanical Garden, the world’s highest-elevation alpine–subalpine botanical garden, noted for its rich vegetation types and complete vertical vegetation belts.

Xu recounted the station’s milestones:

In 2012, it joined the biodiversity research network of the CAS Bureau of Life Sciences, becoming one of CAS’s ten forest stations.

In 2013, it joined the China Forest Ecosystem Research Network and CAS’s Forest Ecological Control Experiment Network.

In 2016, it became part of the China Forest Biodiversity Monitoring Network and the China Botanical Garden Union.

One of the key tasks is collecting and preserving living plant germplasm. About 50 new plant species are planted annually in the Lijiang Alpine Botanical Garden, sourced from regional expeditions to places like Yulong Snow Mountain and Dali.

The station is a key technical platform supporting CAS’s “Three Breakthroughs,” the iFlora Research Initiative, and the “Plant Core Germplasm Innovation Research” project. It also underpins Kunming Institute of Botany’s priority research on the geographic patterns of biodiversity.

1.5

[New Era · New Development · New Outlook] Establishing a Biodiversity Database: Lianyungang Coordinates Ecological Resources for High-Quality Growth

Yesterday, reporters learned that to implement the Three-Year Action Plan for High-Quality Ecological Development, Lianyungang City will fully launch the construction of an “eco-port city.” Within three years, the city aims to complete a comprehensive baseline survey and assessment of biodiversity, establish a biodiversity database, and provide systematic, scientific, and digital tools for the protection of mountains, rivers, forests, farmland, lakes, and grasslands.

Lianyungang will strengthen ecological red-line management by ensuring that red-line areas do not shrink and protection standards are not lowered. The city will enhance coastline protection and restoration, coordinate land–sea management, strictly enforce controls on sea use for construction projects, and maintain a natural coastline retention rate of no less than 31%. A municipal-level marine ecological red-line system will be implemented, ensuring the red-line ratio is no less than 30.8%. By 2020, the city planned to establish two provincial-level wetland parks, restore 10,000 mu (≈667 hectares) of wetlands, and achieve a natural wetland protection rate of 52%.

Lianyungang will also coordinate ecological resources to build a green port city with a “four horizontal and three vertical” framework. By 2020, it aimed to add 500,000 mu (≈33,333 hectares) of afforestation, raise forest cover to 31%, expand urban green space by 900 hectares, and provide 14 m² of park green space per capita. A pilot biodiversity survey had already begun in Donghai County, with plans to complete a citywide baseline survey and establish a biodiversity database. By then, protection rates for nationally key protected species and representative ecosystem types would reach 95%.

The city will promote ecological civilization demonstration projects, leveraging its mountains, rivers, coastline, wetlands, and island resources to enhance competitiveness. With “eco +特色 (local特色)” as the guiding principle, it will develop characteristic villages and towns, supported by the region’s natural endowments. By 2020, Lianyungang aimed to establish at least three national-level and over 50 provincial-level ecological civilization demonstration sites.

eDNA Technology Confirms the Existence of the World’s Fourth Yangtze Giant Softshell Turtle — Could a Fifth Be Found in Yunnan?

“Another Yangtze giant softshell turtle found in Vietnam!”

On April 12, the Turtle Survival Alliance (TSA), the world’s leading turtle conservation organization, announced on its website that Dr. Caren Goldberg, Assistant Professor at Washington State University, had confirmed the presence of this turtle through environmental DNA (eDNA) testing.

The news quickly spread across social media.

How endangered is this species?

Some may wonder: “What’s so exciting about finding just one turtle?” This question reflects a lack of understanding of how critically endangered the species is.

The Yangtze giant softshell turtle (Rafetus swinhoei), an oval-shaped turtle with yellow speckles, can grow to more than one meter in length and weigh over 100 kilograms. Among more than 300 turtle and tortoise species worldwide, it is the rarest and most endangered.

With the individual confirmed on April 12, there are now only four known living turtles of this species: two in Suzhou Zoo in China and two in Vietnam — one in Dong Mo Lake and the newly discovered one in Xuan Khanh Lake.

From common species to critically endangered in just half a century

In 1873, British taxonomist John Edward Gray studied a turtle specimen collected near Shanghai and sent to the British Museum by Robert Swinhoe. Using Linnaeus’s binomial system, Gray named the species Oscaria swinhoei, calling it the most beautiful turtle he had ever seen.

For over a century, however, due to limited research conditions and the similarity of juvenile R. swinhoei to the Chinese softshell turtle (Pelodiscus sinensis) and adults to the Asian giant softshell turtle (Pelochelys cantorii), the species was repeatedly misidentified.

It was not until 1988 that Meylan and Webb restored its taxonomic validity, placing it in the genus Rafetus with the Latin name Rafetus swinhoei. During this time, Chinese herpetologist Zhao Kentang tirelessly advocated for its recognition. It wasn’t until 1994 that Zhao Ermi formally acknowledged Rafetus swinhoei’s validity in Chinese academia and recommended the Chinese name “斑鳖” (Spotted Softshell Turtle).

1.7

Carefully Protecting the Ecological Environment: Making Hainan’s Mountains Greener and Waters Cleaner

In April 2013, during an inspection tour in Hainan, General Secretary Xi Jinping emphasized that lush mountains, clear waters, and blue skies are the province’s greatest assets for building an international tourism island. He urged Hainan to balance development and protection, enhance greenery and safeguard marine ecosystems, set an example in ecological civilization, and leave behind a sustainable “green bank” for future generations.

Thanks to years of prioritizing ecology and environment, Hainan has followed a low-carbon, green, and sustainable development path.

For example, forest ranger Wei Guang has guarded the Bawangling Reserve, home to the critically endangered Hainan gibbon, for 17 years. Since his arrival, the gibbon population has grown from 13 to more than 27 individuals. Similarly, in Datian National Nature Reserve, the Hainan Eld’s deer has increased from 26 to 428 individuals since the reserve was first established.

As ecosystems recover, biodiversity has flourished across Hainan’s reserves. Yinggeling Nature Reserve, a key genetic resource bank, records more than 4,000 species of flora and fauna, including 2,262 vascular plant species, with 9 species under Class I national protection and 74 species under Class II.

Since Hainan became a province, the number of forestry nature reserves has increased from 19 to 30, and protected area expanded from 100,000 hectares to 240,000 hectares. These include eight national forestry nature reserves (Tongguling, Diaoluoshan, Jianfengling, Dongzhaigang, Wuzhishan, etc.) as well as the Sanya Coral Reef National Nature Reserve and Dazhou Island National Marine Nature Reserve.

1.8

Charming Biodiversity: The Harvest Mouse — A Flower-Bed Fairy

In Bournemouth, UK, photographer Miles Herbert captured delightful images of harvest mice (Micromys minutus) in tulip fields near his home. The tiny creatures climbed flower stems, curled up in the blossoms, and played among the fragrant petals, creating enchanting scenes.

The harvest mouse, also known as the Eurasian harvest mouse or dwarf mouse, builds its nests on plant stems — hence its name. Apart from jerboas, it is the smallest rodent in the order Rodentia. With a tiny skull, short rounded ears, and a prehensile tail, it looks adorable while also being highly agile, often using its tail to assist in climbing. Occasionally, it even swims in shallow water.

Although mostly nocturnal, harvest mice are sometimes active during the day. Typically, mothers forage at night while their young stay in the nest. These omnivores feed on grains such as corn, millet, soybeans, and rice, which makes them agricultural pests. In summer, they build spherical nests in grass or shrubs, while in winter, they dig burrows underground.

While generally causing limited agricultural damage, harvest mice are vectors of certain zoonotic diseases such as hemorrhagic fever with renal syndrome, leptospirosis, and plague.

1.9

Diebu Earns a World-Class Recognition

On April 19, the Fifth International Forum on Globally Important Agricultural Heritage Systems (GIAHS), organized by the FAO, was held in Rome, Italy.

China had four new systems recognized:

The Diebu Zhagana Agroforestry System (Gansu)

The Huzhou Mulberry–Dyke & Fish–Pond System (Zhejiang)

The Ancient Mulberry Tree Group in the Yellow River Old Course, Xiajin (Shandong)

The Southern China Mountain Rice Terraces (including Congyi Hakka Terraces, Youxi United Terraces, Xinhuaziquejie Terraces, Longsheng Longji Terraces)

With these additions, China now has 15 GIAHS sites, the most of any country.

Diebu’s Zhagana is a glacial landform heritage site, a provincial geopark, and was listed among China’s Top Ten Lesser-Known Peaks by Chinese National Geography in 2009. It was recognized as one of the first Chinese Important Agricultural Heritage Systems in 2013, a “Most Beautiful Leisure Village” in 2014, a “Model Village for Rural Tourism” in 2015, and ranked sixth in “China’s 30 Most Beautiful Places” in 2017.

1.10

China’s First National Botanical Garden Hidden in Xi’an! The Qinling Botanical Garden Opens Today

On September 27, the Qinling National Botanical Garden held its grand opening ceremony, marking a new milestone for ecological civilization in Shaanxi.

Jointly established by the Shaanxi Provincial Government, the State Forestry Administration, the Chinese Academy of Sciences, and Xi’an Municipal Government, the garden covers 639 km², with an elevation difference of over 2,000 meters. It is China’s only national botanical garden, the largest in the world, with the clearest vegetation zonation and most natural landscape. It is also one of the five core botanical gardens under CAS.

The garden is divided into four zones:

Ex-situ Plant Conservation Zone (10 km²)

In-situ Conservation Zone (575.31 km²)

Ex-situ Rare Animal and Cultural Heritage Protection Zone (16 km²)

Composite Ecological Function Zone (37.69 km²)

Qinling hosts rich biodiversity, with distinct vegetation belts and over 40 nationally protected animal species, including giant pandas, crested ibises, golden snub-nosed monkeys, and takins.

Major projects include:

One River: Ecological protection of the Tianyu River Basin

Two Squares: Entrance Plaza and Parking Area

Three Lakes: Cui Lake, Taiji Lake, and Maple Leaf Lake

Four Halls: Science Education Hall, Herbarium, Greenhouse, and Paleontology Hall

Six Zones: Wetland restoration, aquatic plant area, flower introduction trial plots, visitor reception areas, alpine plant zones, and more

Eighteen Specialized Gardens: e.g., Crabapple Garden, Osmanthus Garden, Maple Garden

2. 2019/05/27-2019/06/02

2.1

China Green Development Foundation Joins First Batch of Domestic Biodiversity Partnerships

On May 23, during the “International Day for Biological Diversity 5.22” publicity event in Nanchang, Jiangxi Province, organized by the Ministry of Ecology and Environment, a signing ceremony was held for the Biodiversity Partnership.

At the signing, the China Green Development Foundation (China Green Foundation), whose mission is biodiversity conservation and green development, joined other social organizations and corporate representatives in formally signing the Biodiversity Partnership Declaration, becoming one of the first eight domestic institutions to join.

Since the third Conference of the Parties (COP3) to the Convention on Biological Diversity in 1996, the Global Partnership on Biodiversity has grown steadily, attracting more enterprises and social organizations worldwide to participate in biodiversity conservation.

“The Biodiversity Partnership will play an important role in building a Beautiful China. It will serve as a bridge and link between social organizations, enterprises, research institutions, and government departments; facilitate dialogue between organizations and government, providing suggestions for laws and regulations; assist in developing biodiversity strategies; and promote communication, share practices, and carry out extensive domestic and international exchanges to showcase and share our achievements in biodiversity conservation.”

2.2

Biodiversity Effectively Protected at Daya Bay Nuclear Power Base

What do you imagine a nuclear power plant looks like? Guard towers, concrete, and no greenery? Or a barren, lifeless zone? On June 1, International Children’s Day, China General Nuclear Power Group (CGN) and the “Dive into Dapeng” Coral Conservation Volunteer Association jointly announced that the Daya Bay Nuclear Power Base is home to over 200 species of plants and animals, with biodiversity well protected. Among these, eight nationally protected terrestrial species and seven nationally protected coral species were identified. Visitors to the site were astonished, saying the nuclear base had become a “paradise” for wildlife.

Located on the Dapeng Peninsula in Shenzhen, Guangdong Province, the Daya Bay Base houses six 1-million-kilowatt pressurized water reactor units — the world’s largest such nuclear facility. On its 10 km² of land, wild plants and animals thrive, while its 11 km winding coastline shelters precious corals.

Long-term monitoring by volunteers over 15 years, combined with recent surveys, revealed over 200 species of terrestrial and marine flora and fauna. Protected species observed on land include the changeable lizard, egret, cattle egret, crested goshawk, common buzzard, collared scops owl, python, and the Chinese wisteria (Wisteria sinensis).

2.3

Zhou Jinfeng Shares with Zhejiang University Students: Biodiversity and Ecological Civilization

“What can Chinese youth do for biodiversity conservation and ecological civilization?” On May 29, Dr. Zhou Jinfeng, Secretary-General of the China Green Development Foundation, delivered a keynote address at Zhejiang University on biodiversity protection, ecological civilization, and the role of young people. The session was hosted by Professor Zhu Zengrong from the College of Agriculture and Biotechnology.

Dr. Zhou introduced the background of the Convention on Biological Diversity (CBD), the UN Millennium Development Goals, the Sustainable Development Goals, and the Aichi Targets. Framing his talk in the context of the Anthropocene and the “sixth mass extinction,” he highlighted the unsustainability of industrial civilization and the necessity of moving into the era of ecological civilization.

He also discussed biodiversity conservation and ecological civilization from the perspective of digital and life sciences, sharing examples from the Foundation’s practical work. Emphasizing that youth are the future — and the backbone of biodiversity protection — he encouraged students to actively join the efforts to build ecological civilization and promote sustainable development.

2.4

CCTV Exclusive Interview: Zhou Jinfeng on Natural Mudflats and Biodiversity Protection

On May 24, in response to the sand-mining incident at Bagua Shoal in Tianjin, CCTV interviewed Zhou Jinfeng, Secretary-General of the China Green Development Foundation. Zhou stressed that natural mudflats are the “cradle of life,” vital for maintaining biodiversity. He warned that biodiversity loss will ultimately lead to the extinction of all life. The rapid decline of mudflats is an urgent challenge in ecological civilization building.

He called on everyone to study ecological civilization concepts seriously, for businesses to practice them, and for media to take responsibility by highlighting and intervening against environmental damage. Zhou urged all parties to take part in biodiversity conservation, starting with small, concrete actions to usher in a new era of ecological civilization.

The interview aired on CCTV News Channel’s Live News. Also interviewed was Wang Jianmin, Director of the “Relict Gull Conservation Area – Tianjin.”

Thanks to joint efforts by the Foundation, related organizations, local media, Tianjin Daily, city management authorities, and volunteers, the Bagua Shoal incident gained national attention, leading to significant reductions in illegal sand mining and habitat disturbance.

2.5

City Holds Science Popularization Event for “International Day for Biological Diversity”

May 22 marked the International Day for Biological Diversity. This year’s theme, “Our Biodiversity, Our Food, Our Health,” emphasized the dependence of food, nutrition, and health on biodiversity and healthy ecosystems.

To popularize biodiversity knowledge, raise public awareness of its importance, and advance ecological civilization, the Municipal Ecology and Environment Bureau, together with the Hunjiang District Branch, organized a science education event at Baishan Radio and TV University.

The program included lectures, distribution of educational materials, and interactive Q&A. Staff explained the significance of biodiversity conservation, its role, and local protection measures, while distributing over 200 informational leaflets. More than 200 community residents participated.

Through the activity, residents gained a deeper understanding of the close relationship between biodiversity and human survival, strengthened their awareness of protecting biodiversity and the environment, and helped foster a positive atmosphere for broad public participation in conservation.

2.6

Qianjiang Holds International Day for Biological Diversity Publicity Event

On May 22, led by the District Forestry Bureau with participation from the District Planning and Natural Resources Bureau, District Ecology and Environment Bureau, District Water Resources Bureau, and others, Qianjiang organized an International Day for Biological Diversity publicity campaign at Wuling Cultural Square under the theme “Protect Biodiversity, Build a Green Home Together.”

The event featured banners, display boards, and distribution of informational materials. More than 2,000 pieces of biodiversity-related leaflets and posters were distributed to raise public awareness of biodiversity conservation, promote harmony between people and nature, and advance ecological civilization in building a livable Qianjiang.

The district is home to 655 species of vascular plants (137 families, 408 genera), including 4 nationally protected Class I plants and 7 Class II plants. It also has 213 species of vertebrates (27 orders, 56 families, 153 genera), including 11 nationally protected Class II animals. By the end of 2018, Qianjiang had established multiple reserves and parks, including Xiaonanhai National Geopark, Apongjiang National Wetland Park, a National Forest Park, Yangtoushan City-level Forest Park, Wuling Mountain Nature Reserve, and Xiaonanhai City-level Nature Reserve.

The activity helped popularize biodiversity knowledge, strengthen public awareness, and advocate starting with individual actions — reducing demand and destruction of nature, avoiding waste, and practicing waste sorting — to protect local biodiversity.

2.7

International Day for Biological Diversity: UN Calls for Urgent Action

May 22 marked the International Day for Biological Diversity. On that day, UN Secretary-General António Guterres warned that the world’s ecosystems face unprecedented threats and that urgent action is required.

Guterres stressed that biodiversity is vital for human health and well-being — the quality of our water, food, and air all depend on healthy ecosystems. He emphasized that ecosystems also contribute 37% of the mitigation needed to limit global temperature rise, helping achieve the Sustainable Development Goals and fight climate change.

However, a report by the Intergovernmental Science-Policy Platform on Biodiversity and Ecosystem Services (IPBES) found that nature is degrading at an unprecedented rate in human history. Since 1990, more than 290 million hectares of forest have been lost to deforestation. One million species of plants and animals are at risk of extinction, and over 90% of marine fish populations are declining or overfished.

“This will have severe consequences for people everywhere,” Guterres said. “The current negative trend threatens the achievement of 80% of the Sustainable Development Goals. We cannot allow this to happen.”

2.8

Biodiversity Conservation and the Convention on Biological Diversity

When it comes to biodiversity conservation, one cannot overlook the Convention on Biological Diversity (CBD). This international treaty for the protection of biological resources was signed in Rio de Janeiro in June 1992 and came into force on December 29, 1993. It is legally binding and currently has 196 Parties.

China signed the CBD on June 11, 1992, and ratified it on January 5, 1993, becoming one of the earliest signatories and ratifiers. China later ratified two CBD protocols: the Cartagena Protocol on Biosafety (2005) and the Nagoya Protocol on Access and Benefit Sharing (2016).

The Birth of the Convention

As early as the 1970s, the international community had recognized both the importance of biological resources for human development and the threats they faced.

In 1972, the UN Conference on the Human Environment was held in Stockholm, which led to the creation of the UN Environment Programme (UNEP). Governments signed various regional and international agreements on issues such as wetland protection and trade in endangered species. Although these agreements did not reverse biodiversity loss, they played a positive role and laid the foundation for future efforts.

2.9

Diqing Tibetan Middle School Students Experience Biodiversity at Alpine Botanical Garden

On May 22, the Diqing Prefecture Ecology and Environment Bureau and Diqing Tibetan Middle School co-organized an International Day for Biological Diversity event at Shangri-La Alpine Botanical Garden, themed “Our Biodiversity, Our Food, Our Health.” The event promoted sustainable development and biodiversity conservation.

In May, the Alpine Botanical Garden was full of flowers and lush greenery. Experts guided teachers and students through native grasslands, traditional grazing areas, plant genetic resource conservation zones, scenic valleys and forest corridors, science exhibition halls, and public education and academic exchange areas, while explaining Diqing’s biodiversity.

“The rare and unique plants amazed me, and the experts’ explanations were vivid and interesting. I learned so much,” said Zhuoma Chunzong, a first-year student. “These plants are not only beautiful but also play vital roles in ecosystems. From now on, I will cherish plants more, care for the environment, and tell others that conserving biodiversity means protecting our living environment and our shared Earth.”

Teacher Yang from the school added: “I hope students broaden their horizons and learn about their environment. I also urge everyone to care about endangered species and protect biodiversity through awareness and action.”

2.10

Biodiversity Conservation: Ecological Protection Is Close to Us All

The 1992 adoption of the Convention on Biological Diversity recognized that biodiversity is the foundation of human survival and development. As global awareness of biodiversity has grown, significant progress has been made in conservation. Yet, the trend of biodiversity decline has not been fundamentally reversed: deforestation, population growth, and species loss continue to challenge human society.

China is one of the most biodiverse countries in the world, but also one of the most threatened. Many may ask: what does biodiversity mean for us, and how does it affect our daily lives? The truth is, biodiversity is a basic element of human health and food systems.

A 2019 FAO report on The State of the World’s Biodiversity for Food and Agriculture found that while China’s food systems remain rich, in some areas wild relatives of key crops are disappearing at an accelerating rate.

This is a serious challenge. As one commentator put it, while society focuses on capital markets, entrepreneurship, and investment, we must pause and look at our ecological environment. All human progress rests on the foundation of survival. Protecting biodiversity is not just for nature, but for ourselves and future generations.

3. 2020/05/18-2020/05/24

3.1

International Day for Biological Diversity丨China’s First White Paper on Biodiversity Released in Kunming: Biodiversity in Yunnan

On May 22, the Information Office of the Yunnan Provincial People’s Government held a press conference at Haigeng Hall for the 2020 “5·22” International Day for Biological Diversity, where China’s first biodiversity white paper — Biodiversity in Yunnan — was officially released in Kunming.

The white paper consists of five sections: Preface, Achievements in Conservation, Conservation Measures, Future Directions, and Conclusion, with a total of more than 20,000 words.

Yunnan’s Strategic Role in Biodiversity Conservation

Yunnan holds a crucial strategic position in China and globally for biodiversity conservation.

It plays a vital role in maintaining the ecological security barrier of Southwest China.

With the joint efforts of Party committees, governments, departments of forestry, agriculture, natural resources, development and reform, finance, water resources, science and technology, education, and society at large, Yunnan has achieved remarkable results, ranking among the top in the country.

Key Measures Taken in Yunnan

Improving the Biodiversity Conservation System: Enacted China’s first biodiversity protection regulation — Yunnan Province Biodiversity Protection Regulations. Major lakes such as Dianchi, Fuxian, Erhai, and Lugu each have specific ordinances (“one lake, one regulation”), while nine protected areas such as Lashihai (Lijiang) and Dashanbao (Zhaotong) have specific laws (“one area, one law”).

Diversified ecological compensation mechanisms: Established wildlife damage compensation schemes.

Strengthening biodiversity research: Established national and provincial-level platforms, including the State Key Laboratory of Genetic Resources and Evolution, the National Forestry and Grassland Administration’s Asian Elephant Research Center, and the Yunnan Snub-Nosed Monkey Research Center.

3.2

Vice Minister: China’s Biodiversity Protection Achieves Positive Results

May 22 is the International Day for Biological Diversity. Vice Minister of Ecology and Environment Zhuang Guotai recently stated that China’s nature reserves now cover over 18% of the country’s land area. Most nationally protected wild plants and animals are effectively conserved; nearly 10 endangered species, including the crested ibis and Amur tiger, have begun to recover; and more than 60 species of rare and endangered wildlife have been successfully bred in captivity.

The 15th Conference of the Parties (COP15) to the Convention on Biological Diversity is planned to be held in Kunming. Elizabeth Maruma Mrema, Acting Executive Secretary of the CBD Secretariat, emphasized that China’s vision of building an “ecological civilization” is not only China’s aspiration but also reflects the common desire of humanity worldwide.

Biodiversity is the foundation of human survival and development, closely tied to everyone’s daily life and production. All countries share the responsibility to protect biodiversity and must cooperate to use biological resources sustainably. Yet human activities are rapidly reducing species populations. What measures has China taken with modern biotechnology to safeguard biodiversity?

3.3

International Day for Biological Diversity: Kunming Uses “Cloud Power” to Support Biodiversity Conservation

On May 22, the 27th International Day for Biological Diversity, Kunming launched a series of events under the theme “2020·5·22 — Promoting Biodiversity Conservation and Building a Model City for Beautiful China.” Citizens were invited to participate in protecting biodiversity, with digital platforms providing “cloud power” to support conservation efforts.

The international theme this year was “Our Solutions Are in Nature,” while Kunming’s theme was “Promoting Biodiversity Conservation and Building a Model City for Beautiful China.” Both aimed to advocate for public participation and collective action in biodiversity protection.

At the event site, exhibition boards helped spread biodiversity knowledge. Chen Jun, Deputy Director of Kunming Ecology and Environment Bureau, urged the public to start with small actions, becoming advocates and practitioners of biodiversity conservation, and to cherish and care for green mountains and clear waters.

To broaden engagement, organizers launched an interactive online mini-program “Participation is Power: 2020 International Day for Biological Diversity — Our Solutions Are in Nature.” Within two days, it had already attracted over 20,000 participants.

3.4

Yunnan to Launch Major Biodiversity Conservation Projects to Save Endangered Species

On May 22, Yunnan Provincial People’s Procuratorate, Yunnan Ecology and Environment Department, and Yunnan Forestry and Grassland Bureau jointly held a press conference for the International Day for Biological Diversity. Gao Zhengwen, First Inspector of the Department of Ecology and Environment, announced that Yunnan will implement major biodiversity projects, focusing on rescuing rare and endangered species, conserving species with extremely small populations, and strengthening biosafety and ecological security management, while also tackling invasive alien species.

Key actions include:

Integrating biodiversity conservation into the province’s “five major” development frameworks (politics, economy, society, culture, and ecological civilization).

Promoting biodiversity education across government agencies, Party schools, schools, enterprises, and communities to foster a social atmosphere of protection.

Strengthening accountability mechanisms, enforcing natural resource asset audits and lifelong accountability, and improving ecological damage compensation and liability systems.

Enhancing unified law enforcement, combining biodiversity conservation inspections with special campaigns such as the “Green Shield” protection initiative.

Expanding funding channels through fiscal transfers, ecological compensation, donations, corporate investment, international cooperation, and market-based approaches.

Conducting ecosystem monitoring and assessments, building a biodiversity big data platform, and establishing monitoring, early warning, and forecasting systems.

3.5

International Day for Biological Diversity: Jiangsu Hosts 165 Rare and Endangered Species, Signs of Ecological Recovery

Today is the International Day for Biological Diversity, themed “Ecological Civilization: Building a Shared Future for All Life on Earth.”

Since 2017, Jiangsu Province has conducted a comprehensive biodiversity baseline survey. Results show 165 rare and endangered species in the province, with populations of some protected animals gradually increasing, reflecting a positive ecological trend.

Examples include:

Laoshan Mountain: habitat of the Chinese Apollo butterfly (Parnassius apollo), where conservation groups have set up 40+ survey lines. Surveys indicate population growth.

2017–2019 survey: recorded 4,588 species across 39 pilot counties — an increase of 460 species compared with previous records. Among these, 165 are rare and endangered.

Zhangjiagang, Suzhou: sightings of the reed parrotbill (dubbed the “panda among birds”).

Gaoyou region: the Oriental stork, a Class I nationally protected species, now has more than 20 nesting sites.

Coastal and Tongshan (Xuzhou) areas: scientists rediscovered the leopard cat.

Experts predict Jiangsu’s species count could reach 5,000–6,000 in the future, showing continued improvement in the province’s ecological environment.

International Day for Biological Diversity: Public Participation is Essential for Conservation

(CNR Beijing, May 20, Reporter Liu Fei) — Biodiversity is the material foundation for human survival and development and is closely tied to every aspect of our production and daily lives. May 22, 2020, marks the International Day for Biological Diversity.

This year, several major global events have sparked renewed reflection on the relationship between humans and nature: Australia’s wildfires burned for over 200 days, devastating many species’ habitats; East African countries were struck by severe locust plagues; and the COVID-19 pandemic spread across the globe.

So, as the International Day for Biological Diversity approaches, what can we do to protect it?

The Jiatang Grassland in Chengduo County, Yushu Prefecture, Sanjiangyuan, is an important water conservation area. It is home to desert cats, snow leopards, 72 bird species including the black-necked crane, and over 150 plateau-endemic plants such as gentians. For a white-collar worker in Beijing, is appreciating photos from afar the only way to “care,” with no real ability to help?

Not so, says Vice Minister of Ecology and Environment Zhuang Guotai: “Through the Jiatang Protected Area public-interest project, citizens only need to accumulate 350 grams of ‘green energy’ from low-carbon lifestyles to apply to protect 1 square meter of nature reserve. To date, 35.18 million square meters have been protected through donations.”

3.7

Biodiversity Column | Ecological Civilization and Biodiversity

May 22 is the International Day for Biological Diversity, designated by the UN General Assembly. This year, China’s publicity focuses on the COP15 theme: “Ecological Civilization — Building a Shared Future for All Life on Earth.”

A Look Back at International Biodiversity Day

In November 1994, the first COP of the CBD (COP1) proposed setting up an International Day for Biological Diversity. In December that year, the 49th UN General Assembly designated December 29 — the date the CBD entered into force — as International Biodiversity Day.

Later, following COP5’s proposal, the 55th UN General Assembly passed Resolution 201 in December 2000, moving the date to May 22, in honor of the day the CBD text was adopted in Nairobi in 1992.

Since 2001, annual commemorations have had unified themes.

2020 Theme: “Our Solutions Are in Nature” (以自然之道·养万物之生).

Biodiversity and Ecological Civilization

Biodiversity is fundamental to building an ecological civilization, ensuring sustainable development, and maintaining harmony between humans and nature.

3.8

Today丨International Day for Biological Diversity

The International Day for Biological Diversity (also known as World Biodiversity Day) highlights the importance of public education and awareness in implementing the Convention on Biological Diversity (CBD) at all levels. On December 20, 2000, the 55th UN General Assembly passed Resolution 201, designating May 22 — the date of the CBD’s adoption — as the International Day for Biological Diversity.

On May 20, 2020, China launched its biodiversity publicity campaign in Beijing. Premier Li Keqiang of the State Council delivered an important instruction.

Chinese name: 生物多样性国际日

English name: International Day for Biological Diversity

Date: May 22 each year

Type: International day

Background: The Significance of Biodiversity

Biodiversity refers to the diversity of life on Earth across all forms, levels, and combinations of living organisms.

It includes genetic diversity, species diversity, and ecosystem diversity.

Biological invasions — when non-native species enter a new area without natural enemies — can reproduce rapidly, disrupt local ecosystems, and threaten native species.

Biodiversity is the product of billions of years of evolution and the foundation of human survival and sustainable development.

3.9

Protecting Biodiversity: China in Action

May 22 is the UN-designated International Day for Biological Diversity. On this day, most countries worldwide hold publicity activities to raise awareness and encourage broad participation in biodiversity conservation, working together to build a beautiful planet. This year, China’s focus is aligned with COP15’s theme: “Ecological Civilization — Building a Shared Future for All Life on Earth.”

China’s Actions

The government has placed biodiversity conservation at the core of ecological civilization and high-quality development.

Established the National Committee for Biodiversity Conservation, chaired by a Vice Premier in charge of ecological environment affairs, to coordinate nationwide work.

Gradually improved the legal and regulatory framework for biodiversity conservation.

Issued and implemented the China Biodiversity Conservation Strategy and Action Plan (2011–2030).

Carried out the UN Decade on Biodiversity — China Action campaign.

Implemented major biodiversity projects, built an initial biodiversity monitoring network, and made significant progress in achieving the Aichi Targets.

3.10

Honoring International Day for Biological Diversity: Saving Endangered Species, We Are Taking Action!

Green development is the trend of the times. On May 22, at latitude 1°N, in the warm tropical breeze, we once again welcome International Day for Biological Diversity.

Biodiversity is the result of billions of years of evolution, the resource on which humanity depends, and a precious gift from nature.

In 1992, at the UN Conference on Environment and Development in Rio de Janeiro, 153 countries signed the Convention on Biological Diversity.

In 1994, the UN General Assembly designated December 29 as International Biodiversity Day.

In 2001, the date was changed to May 22.

The Harsh Reality

Nature has no substitutes. If we use it without awareness, once lost, it is gone forever. Modern human activities are increasingly damaging the environment. Biodiversity is under unprecedented threat:

Every hour, one species disappears.

About 34,000 plant species and over 5,200 animal species face extinction.

Species extinction is irreversible and can trigger cascading effects through the food chain, endangering many more species.

Urgent Call

Let us now get to know the rare species on the brink of disappearing — and take immediate action to save them.

4. 2021/05/17-2021/05/23

4.1

International Day for Biological Diversity | 90% of China’s Terrestrial Ecosystems and 85% of Key Wildlife Populations Effectively Protected

(CCTV News) Today (May 21) is the International Day for Biological Diversity. Since the 18th National Congress of the Communist Party of China, the country has established a protected-area system with national parks at its core. These protected areas now cover more than 18% of China’s land area, ensuring effective protection for 90% of terrestrial ecosystem types and 85% of key wildlife populations.

Biodiversity, comprising ecosystem diversity, species diversity, and genetic diversity, is a vital part of the ecological environment and is essential for human survival and sustainable socio-economic development.

According to Liu Yan, Director of the Center for Biodiversity Conservation and Biosecurity at the Nanjing Institute of the Ministry of Ecology and Environment, the five major direct drivers of global biodiversity loss are: land-use change, overexploitation of resources, pollution, invasive alien species, and climate change.

Liu also noted that in recent years China has introduced the ecological conservation redline system, with redline areas covering more than 25% of the country’s land area, effectively protecting ecosystems and species.

4.2

Beijing Releases Interim Results of Biodiversity Survey: 70 Newly Recorded Species Discovered

Yesterday, the Beijing Municipal Ecology and Environment Bureau released interim results from the city’s biodiversity survey. Reporters learned that since the launch of the survey in 2020, 5,086 species have been documented, including 70 newly recorded in Beijing and 12 species newly recorded for China. During the 14th Five-Year Plan period, the city aims to achieve full survey coverage across all grid units to dynamically map its biodiversity baseline.

Survey Across 212 Grid Units

Beijing is one of the world’s most biodiversity-rich megacities and the world’s capital with the greatest bird diversity. During the 13th Five-Year Plan, the city’s ecological index (EI) rose continuously, from 64.2 in 2015 to 70.2 in 2020, an increase of 9.3%. Although Beijing covers only 0.17% of China’s land area, it accounts for about 8% of the nation’s plant species. Birds recorded in Beijing account for more than one-third of China’s bird species, a unique status among northern cities.

According to Cao Zhiping, Director of the Bureau’s Ecology Division, the city was divided into 212 survey grids in 2020 in line with national standards. Comprehensive surveys of ecosystem diversity and species diversity—including mammals, birds, amphibians, fish, insects, and algae—were carried out in selected grids.

4.3

2021 International Day for Biological Diversity Press Conference

Today is the UN-designated International Day for Biological Diversity, and this year’s theme is “We’re Part of the Solution.” To mark the occasion, the provincial government press office held a news conference, inviting leaders from the Department of Science and Technology, Department of Ecology and Environment, Department of Agriculture and Rural Affairs, and the Forestry and Grassland Bureau to announce major achievements in biodiversity science and technology. These included the release of the Catalogue of New Species and New Records in Yunnan (1992–2020), updates on Yunnan’s seed industry development, and advances in breeding and protecting new forestry plant varieties.

May 22 commemorates the adoption of the Convention on Biological Diversity (CBD) at the Nairobi conference in 1992. In October this year, COP15 of the CBD will be held in Kunming—a global event in biodiversity conservation and a rare opportunity to showcase Yunnan’s rich biodiversity.

To prepare for COP15, the Yunnan Provincial Department of Ecology and Environment, together with the Xishuangbanna Tropical Botanical Garden of the Chinese Academy of Sciences (CAS), compiled the Catalogue of New Species and New Records in Yunnan (1992–2020), with support from CAS Kunming Institute of Botany, CAS Kunming Institute of Zoology, and Southwest Forestry University.

4.4

Strengthening Spatial Planning and Building Ecological Corridors: Comprehensive Biodiversity Protection in China

Today (May 22) marks the International Day for Biological Diversity. Biodiversity is the foundation of harmony between humans and nature. The Ministry of Natural Resources reported that China has been intensifying efforts to protect ecological space and biodiversity.

Mangroves are among the most diverse ecosystems on the planet. In the Zhangjiangkou Mangrove National Nature Reserve in Fujian Province, over 150 bird species and numerous benthic organisms thrive. However, invasive Spartina alterniflora has spread aggressively, threatening local ecosystems and biodiversity.

Professor Zhang Yihui from the College of Environment and Ecology at Xiamen University noted: “When we talk about biodiversity, we think of maximizing species numbers. But biodiversity includes species beneficial to humans and species harmful to ecosystems. For invasive species like Spartina alterniflora, control measures are necessary.”

In response, the reserve has made invasive species eradication a priority, investing more than 1.5 million yuan to remove Spartina and expand mangrove coverage by 50 hectares.

Huang Guanmin, Director of the Reserve’s Science and Education Center, said: “After the removal, foraging grounds for waterbirds increased. Space once occupied by Spartina has been cleared, benthic life has flourished, and native species like mudskippers and fiddler crabs have become more abundant.”

4.5

International Day for Biological Diversity: Rare and Endangered Species in Jiangsu Reaching 165, Ecosystem Improving

May 22 marks the International Day for Biological Diversity. Through proactive measures, China has achieved remarkable progress in protecting wildlife, plants, and nature reserves.

At Jiulongkou Ecological Wetland in Jianhu, Yancheng, Jiangsu, visitors can often see flocks of reed parrotbills—rare birds known as the “giant pandas of the avian world.”

Thanks to strong environmental protection, more than 400 bird species now inhabit the area, making it an internationally renowned migratory bird corridor. In 2019, the Yancheng Yellow Sea Wetlands were inscribed as a UNESCO World Natural Heritage Site, China’s 14th such site.

In recent years, biodiversity conservation has been comprehensively strengthened across China, with 11,800 protected areas of various types—including nature reserves, forest parks, wetland parks, marine parks, and geoparks—covering 18% of land area and 4.1% of marine area.

Populations of many endangered species are stabilizing or increasing. The wild giant panda population, for example, has grown from 1,114 in the 1970s–80s to 1,864 today. Meanwhile, China is accelerating the establishment of a protected-area system with national parks at its core. Pilot programs have been launched for 10 national parks, including the Giant Panda, Amur Tiger and Leopard, and Sanjiangyuan, significantly boosting biodiversity protection outcomes.

4.6

International Day for Biological Diversity | Protecting Biodiversity Through Parent–Child Planting and Hands-On Ecological Experience

Today is the International Day for Biological Diversity. According to Notice No. 2021-016 issued on March 11, 2021, by the Secretariat of the Convention on Biological Diversity, the 2021 theme is “We’re part of the solution.” This theme builds on last year’s momentum of “Our solutions are in nature,” emphasizing that biodiversity provides answers to many sustainable development challenges. From nature-based solutions to climate change, public health, food and water security, and sustainable development, biodiversity is the foundation for rebuilding our planet.

On May 22, the Xuhui District Ecology and Environment Bureau, together with Xujiahui Subdistrict, held a themed event at the Rainbow No. 2 Residential Community’s New Era Civilization Practice Station under the banner “Protect Nature, We’re Part of the Solution—2021 International Day for Biological Diversity.” Volunteers from China Unicom Shanghai South Branch, Shanghai Xuhui Software Development Co., community parent–child families, and “Bottle Garden” volunteers participated.

The Bureau gave a presentation on “What is biodiversity?” and “Why should we protect biodiversity?” The host then engaged participants with questions such as “How do children understand biodiversity?”, “What is a living organism?”, “How can we prevent invasive species?”, and “How do herbicides harm biodiversity?”—all designed to provoke reflection on protecting the ecological environment.

4.7

China Achieves Significant Results in Biodiversity Protection

(Beijing, May 22, People’s Daily Online) Today is the International Day for Biological Diversity, with this year’s theme “We’re part of the solution.” China is one of the world’s most biodiverse countries, with ecosystems of many types. More than 2,900 terrestrial vertebrate species have been recorded, accounting for over 10% of the global total, and over 36,000 higher plant species, ranking third globally.

According to the National Forestry and Grassland Administration, in recent years China has expanded biodiversity protection efforts, strengthening conservation of wildlife and habitats. As a result, 90% of vegetation types, 65% of higher plant communities, and 85% of key protected wildlife populations are effectively safeguarded.

Giant pandas increased from 1,114 in the 1970s–80s to 1,864 today.

The critically endangered Hainan gibbon rebounded from 7–9 individuals to 33, with a fifth family group now forming.

Crested ibises recovered from just 7 birds at discovery to more than 5,000 today.

Tibetan antelope rose from tens of thousands to over 300,000.

The white-headed langur population grew from about 300 in the 1980s to more than 1,300.

Siberian cranes increased from 210 to over 4,500.

China has also advanced wild plant conservation through propagation and ex situ protection, reintroducing 206 endangered plants into the wild, including 112 species endemic to China.

4.8

International Day for Biological Diversity | First Major Update to the Wildlife Protection List in 32 Years

May 22 is the International Day for Biological Diversity, and wildlife conservation is a crucial part of biodiversity protection.

In February 2021, the National Forestry and Grassland Administration and the Ministry of Agriculture and Rural Affairs jointly released a revised List of National Key Protected Wild Animals. This was the first major revision since its release in 1988. Previously, there were only two minor adjustments: musk deer (2003) and pangolins (2020) were upgraded to Class I.

43 Species Newly Elevated to Class I

The revised list made sweeping changes. Sixty-five species were upgraded from Class II to Class I, including the Yangtze finless porpoise—the only remaining mammal in the Yangtze River and often called the “giant panda of the water.”

Additionally, 43 species were newly added directly to Class I, such as the large-spotted civet, small-toothed civet, and banded civet. The China Felid Conservation Alliance (CFCA) had long advocated for higher protection status for civets. CFCA head Song Dazhao called the revision “inspiring,” noting that civet species face severe survival crises.

For example, all three civet species newly elevated to Class I have been recorded fewer than 10 times in the wild in China over the past 20–30 years. The small-toothed civet has never been recorded in the wild. Song explained that while its arboreal lifestyle makes it hard to capture on ground-based infrared cameras, the lack of sightings also suggests it may already be functionally extinct in China.

4.9

Biodiversity Day: What It Means for You and Me, and China’s Progress in Protection

Biodiversity Day, proposed by the United Nations Environment Programme, is an international observance dedicated to protecting and sustainably using biodiversity. This year, May 22 marks the 21st International Day for Biological Diversity.

In an interview with China Daily, Ma Keping—researcher at the Chinese Academy of Sciences Institute of Botany, Secretary-General of the CAS Biodiversity Committee, and Chair of the IUCN Asia Regional Committee—explained biodiversity’s value from three perspectives:

Direct value: Daily necessities—food, clothing, housing, medicine—are mostly derived from biodiversity. Staple grains, vegetables, and fruits all come from diverse species. A richer biodiversity means a greater range of choices and improved quality of life.

Indirect value: Plants are the only organisms that can fix solar energy into forms usable by other species. Biodiversity regulates climate and maintains oxygen levels. For instance, visiting Tibet in January versus July feels drastically different due to vegetation and biodiversity.

Future option value: Many aspects of biodiversity’s potential remain poorly understood. Protecting it preserves options for future human needs.

4.10

Beijing Hosts 2021 Main Event for International Day for Biological Diversity

On May 21, the main celebration of the 2021 International Day for Biological Diversity was held in Beijing under the theme “We’re part of the solution.” Minister of Ecology and Environment Huang Runqiu attended and delivered a speech.

Huang emphasized that biodiversity is the foundation of human survival and development—the lifeblood of Earth’s community of life. Today, global biodiversity is under unprecedented threat. As one of the most biodiverse countries, China also faces severe challenges. Only by uniting society, embracing the principle of harmony between humans and nature, and aligning ecological protection with economic and social development can sustainable growth be achieved.

He noted that the Chinese government regards biodiversity protection as a core part of ecological civilization and has systematically advanced related work. On September 30, 2020, President Xi Jinping addressed the UN Biodiversity Summit, calling on nations to uphold ecological civilization, multilateralism, green development, and shared responsibility to build a harmonious home for all life.

Guided by Xi Jinping’s vision of ecological civilization, China has taken strong measures: establishing policies and legal frameworks, improving air and water quality, intensifying ecological protection and restoration, advancing biodiversity surveys, monitoring, and assessment, and raising public awareness and participation in biodiversity conservation.

5. 2021/10/11-2021/10/17

5.1

COP15 Launches a “New Decade” of Global Biodiversity Restoration

The first phase of the 15th meeting of the Conference of the Parties to the Convention on Biological Diversity (COP15) concluded on the afternoon of October 15 in Kunming, China. The conference successfully held a high-level meeting, released the Kunming Declaration, and advanced the CBD process, laying an important foundation for the success of the next phase.

Elizabeth Maruma Mrema, Executive Secretary of the CBD Secretariat, described the meeting as “extraordinary” and said it has set biodiversity restoration on the “path to recovery.”

Providing High-Level Political Momentum for Global Biodiversity Governance

Due to the COVID-19 pandemic, the COP15 schedule was postponed twice before finally being confirmed for two phases in October this year and next year. The just-concluded first phase was held in a hybrid online-offline format, including a high-level meeting and an Ecological Civilization Forum, attracting over 5,000 representatives from more than 140 Parties and 30 international institutions and organizations.

During the two-day high-level meeting, nine heads of state, along with the UN Secretary-General, attended the Leaders’ Summit. A total of 125 ministerial representatives from 119 Parties and 26 international organizations, as well as 24 ambassadors to China, also participated. This was unprecedented in CBD history in terms of the level of participation and number of world leaders present.

Leaders and international organization heads called for solidarity, practical actions, innovation and benefit-sharing, and enhanced financial, technological, and capacity support to strengthen biodiversity conservation and global environmental governance.

5.2

Press Conference on the High-Level Segment of the UN Biodiversity Conference

At 7:00 p.m. on October 13, 2021, the COP15 Press Center hosted a hybrid (online and offline) press conference on the high-level segment. Speakers included Elizabeth Maruma Mrema, Executive Secretary of the CBD Secretariat; Zhao Yingmin, Vice Minister of the Ministry of Ecology and Environment, representing Huang Runqiu, COP15 President and Minister of Ecology and Environment; and Wang Xiangang, Vice Governor of Yunnan Province. The press conference was moderated by Liu Youbin, Director of the COP15 Press Center.

They reported that, with the joint efforts of all Parties, the two-day high-level meeting concluded successfully that afternoon. The panel introduced the main achievements of the meeting and responded to questions from journalists.

5.3

Ministry of Ecology and Environment: Three Major Outcomes of the High-Level Segment of COP15

(Kunming, October 13, Xinhua) Vice Minister of Ecology and Environment Zhao Yingmin announced that the high-level segment of the 2020 UN Biodiversity Conference (first phase) achieved three important outcomes.

High-Level Political Momentum: The meeting provided a powerful political push for global biodiversity governance. Leaders and heads of international organizations called for unity, practical action, and strengthened global biodiversity and environmental governance.

Adoption of the Kunming Declaration: After extensive consultation, the meeting adopted the Kunming Declaration, injecting new momentum into global environmental governance and advancing the 2050 Vision of “Living in Harmony with Nature.”

New Chinese Initiatives: China announced new measures, including:

establishing the Kunming Biodiversity Fund with an initial contribution of 1.5 billion RMB;

formally designating the first group of national parks;

releasing sectoral and industry-specific carbon peaking implementation plans, supported by measures to establish a “1+N” carbon peak and carbon neutrality policy framework.

Zhao noted that despite the complex global pandemic situation, the meeting fulfilled its mission, boosted global political will, and built consensus for advancing biodiversity governance.

5.4

Building a Global Ecological Civilization and Protecting Global Biodiversity

Yesterday, the first phase of COP15 concluded. At a press conference that evening, Minister of Ecology and Environment Huang Runqiu stated that the conference achieved three important results, which will strongly guide and encourage all Parties to engage in dialogue, build consensus, cooperate pragmatically, and work toward an ambitious yet balanced and practical “Post-2020 Global Biodiversity Framework.” This will help mainstream biodiversity restoration and realize the 2050 Vision of “Living in Harmony with Nature.”

Balancing Ambition and Pragmatism

Huang explained that in 2010, the 10th COP in Aichi, Japan, adopted the “Strategic Plan for Biodiversity 2011–2020” with 20 Aichi Targets. However, the 2020 UN review found that only six were partially achieved, while some sub-targets worsened.

Against this backdrop, COP15’s first phase provided a systematic summary of global biodiversity efforts, promoted the design of strategies for the next decade and beyond, and drew a new blueprint for biodiversity protection. This is crucial to halting and reversing biodiversity loss and promoting global sustainable development.

5.5

Building a Shared Future for All Life on Earth: A Blueprint for the Next Decade of Biodiversity Protection

On October 15, the first phase of COP15 concluded in Kunming. With the theme “Ecological Civilization: Building a Shared Future for All Life on Earth,” the conference is seen as a milestone in CBD history.

The high-level international meeting brought together representatives for political dialogue under this theme, charting a course for biodiversity protection over the next decade.

A key outcome was the Kunming Declaration, adopted at the October 13 ministerial plenary session. The declaration commits to formulating, adopting, and implementing an effective Post-2020 Global Biodiversity Framework to reverse biodiversity loss and put it on a path to recovery by 2030, ultimately achieving the 2050 Vision of “Living in Harmony with Nature.”

Mainstreaming Biodiversity

“Mainstreaming” is a central concept of the Kunming Declaration: integrating biodiversity protection into political, economic, and social planning and development at national and regional levels. This approach addresses gaps in past biodiversity implementation and serves as a future action plan.

The declaration made 17 commitments, including:

strengthening cooperation to integrate biodiversity conservation and sustainable use into decision-making;

accelerating updates to National Biodiversity Strategies and Action Plans (NBSAPs);

improving and expanding protected area systems;

enhancing the global legal framework for environmental protection.

5.6

Four Highlights of the 2020 UN Biodiversity Conference

The 2020 United Nations Biodiversity Conference actually consisted of three meetings: the 15th Conference of the Parties (COP15) to the Convention on Biological Diversity (CBD); the 10th Meeting of the Parties to the Cartagena Protocol on Biosafety; and the 4th Meeting of the Parties to the Nagoya Protocol on Access and Benefit-sharing. Among these, the “main event” was COP15.

The CBD is an international treaty for the protection of the Earth’s biological resources, currently with 196 Parties. The COP is the treaty’s highest decision-making body, convened every two years. Decisions are made by consensus, requiring agreement from all Parties. At COP13 in 2016, it was announced that China would host COP15.

Every 10 years, the COP sets new biodiversity conservation targets for the next decade. In 2010, COP10 adopted the Aichi Biodiversity Targets (2011–2020). Out of 20 targets, none were fully achieved by 2020, and only six were partially met.

COP15 arrived at a crucial juncture to set new goals for the coming decade. The international community has high expectations, hoping the outcomes will help reverse the accelerating trend of biodiversity loss.

5.7

Biodiversity: China’s Green Development Confidence

“China has officially designated its first batch of national parks — Sanjiangyuan, Giant Panda, Northeast Tiger and Leopard, Hainan Tropical Rainforest, and Wuyi Mountain — covering 230,000 square kilometers and nearly 30% of key national wildlife species.” On October 12, President Xi Jinping addressed the COP15 Leaders’ Summit via video, declaring phrases such as “Those who cherish green mountains will be rewarded by them” and “Lucid waters and lush mountains are invaluable assets.”

The summit theme, “Ecological Civilization: Building a Shared Future for All Life on Earth,” underscored biodiversity’s fundamental role in human well-being and survival. The white paper Biodiversity Conservation in China emphasized that China, as one of the earliest Parties to sign and ratify the CBD, has consistently prioritized biodiversity conservation, keeping pace with the times and making notable progress.

China’s achievements in biodiversity are not confined to academia but have entered public discourse. Milestones include:

The giant panda downlisted from Endangered to Vulnerable.

The Tibetan antelope upgraded from Endangered to Near Threatened.

1.18 billion mu of afforestation, making China the world leader in artificial forests, contributing 42% of global greening.

A 10-year fishing ban across key areas of the Yangtze River, showcasing China’s determination to protect its “Mother River.”

Leading the world in hydropower, wind, and solar power, establishing the largest clean energy system globally.

5.8

Protecting Biodiversity: China’s “Generation Z” in Action

(Kunming, Oct. 15, Xinhua) For most young Chinese born in the 1990s and 2000s — the so-called “Generation Z” — scrolling on a smartphone is as natural as breathing. But 27-year-old Li Ruxue has been trekking deep into the mountains for nearly a week, often without a mobile signal except on ridgelines.

Li has been devoted to fieldwork since college, later joining a grassroots organization to protect the Skywalker hoolock gibbon (also known as the Gaoligong white-browed gibbon), a critically endangered primate found only in narrow areas of the Gaoligong Mountains. His work involves collecting gibbon feces to extract DNA, revealing inbreeding among populations.

“It’s all about luck,” Li explained. One must “happen” to encounter gibbons, “happen” to be present when they defecate, and “happen” that the feces fall without being contaminated. His schedule mirrors that of the gibbons, often leaving little time to eat or rest.

For four and a half years, Li has pursued gibbons, collecting feces and even facilitating “matchmaking” for population management. Recently, he shifted to Lijiang’s Laojun Mountain to study Yunnan snub-nosed monkeys, joined by a growing number of young conservationists, including interns born after 2000.

5.9

Foreign Media Commentary: Biodiversity Conference Highlights China’s Leadership

According to Bloomberg News (Oct. 9), the first phase of COP15 was held in Kunming, China. The meeting, delayed by COVID-19, marked a global negotiation on halting ecological destruction over the next decade, with most sessions conducted online.

The report noted that COP15 was China’s best opportunity to strengthen its leadership in global climate and biodiversity governance. President Xi Jinping’s surprise 2020 pledge for China to achieve carbon neutrality by 2060 positioned the country at the forefront of climate commitments. COP15 was the most significant UN conference China has hosted since the 1995 World Conference on Women, with Chinese media comparing it to the 2015 Paris Climate Conference.

Experts highlighted China’s motivations: environmental protection is seen as both a critical domestic challenge and a way to project global responsibility. The meeting sought to set a framework for action in the coming decade, including habitat protection, reducing plastic waste and pesticides, and mobilizing funding for biodiversity in developing countries.

China’s own track record includes decades of reforestation, protection of endangered species, and the promotion of Ecological Civilization — Xi Jinping’s vision of harmony between humanity and nature.

5.10

Chinese Academy of Sciences: China Leads the World in Many Areas of Biodiversity Research

The first phase of COP15 opened in Kunming on October 11.

On October 10, the CBD Secretariat held a media briefing, announcing the agenda to 78 domestic and international news outlets.

Agenda Highlights

The opening ceremony was scheduled for 3 p.m. on October 11. The conference included a high-level segment and an Ecological Civilization Forum, with press conferences on October 13 and 15 to announce major outcomes.

David Cooper, CBD Deputy Executive Secretary, stressed: “Biodiversity loss poses unprecedented challenges with serious negative impacts on our societies. We must act immediately to confront these crises.”

He emphasized that the high-level segment, especially the Leaders’ Summit and ministerial talks, was critical for setting a clear biodiversity policy direction. The most important outcome, he said, would be advancing the formulation of the Post-2020 Global Biodiversity Framework — a roadmap to guide global biodiversity protection for the next decade and beyond.

6. 2022/05/16-2022/05/22

6.1

Zhejiang Discovers 105 New Species of Wild Flora and Fauna — Biodiversity Achievements Remarkable

At the launch of the 2022 International Day for Biological Diversity event “Biodiversity Conservation in Zhejiang” held in Kaihua County, Quzhou City, it was announced that Zhejiang Province had discovered 100 new wild plant species and 5 new wild animal species in recent years.

May 22 is the International Day for Biological Diversity, and the theme for 2022 was “Building a Shared Future for All Life on Earth.” Notable newly discovered species include the Baishanzu horned toad, Zhejiang daphne, Taishun flat planthopper, and Lishui hook-petal sawfly, adding to the province’s growing biodiversity “treasure chest.”

Biodiversity Achievements in Kaihua

Kaihua, located at the source of the Qiantang River (the “Mother River” of Zhejiang), is a key national ecological function zone with a forest coverage rate of 80.9%. It is also one of 17 globally significant areas for biodiversity conservation.

The county’s biodiversity inventory includes:

2,251 species of higher plants

59 species of mammals

291 species of birds

37 amphibians, 76 reptiles, and 72 fish species

2,601 insect species and 579 large fungi species

Rare and endangered species such as the tufted deer, Asiatic black bear, and cabot’s tragopan.

In recent years, Kaihua has followed the path of “conservation through utilization, and utilization through conservation,” producing replicable experiences that contribute to biodiversity protection and sustainable use nationwide.

6.2

Special Report on the 29th International Day for Biological Diversity | Procuratorial Power Safeguards Ecological Diversity

Recently, the People’s Procuratorate of Xiantao City, Hubei Province, supervised two individuals, Zhang and Zeng, who had engaged in illegal fishing, to release over 2,000 silver carp fingerlings into a tributary of the Han River (a branch of the Yangtze). This act aimed to restore damaged fishery resources and aquatic ecosystems and safeguard biodiversity, witnessed by public supervisors and local residents.

Ancient Tree Conservation with Compensation Funds

In Guangyuan City, Sichuan, the local procuratorate handled a civil public interest litigation case against a building materials company for damaging ecological resources. The company had exceeded its licensed mining area, severely damaging forest land and stripping surface soil. The court supported the procuratorate’s claims, ordering the company to pay more than 600,000 yuan in ecological restoration compensation.

The funds were used to establish an ecological restoration and ancient tree conservation base. At the inauguration ceremony, Guangyuan’s chief procurator Liu Ping remarked that this was “the best way to return compensation funds back to nature.”

6.3

Diverse Life Protects Our Planetary Home

“Biodiversity gives vitality to the Earth and is the foundation of human survival and development. Protecting biodiversity helps safeguard our common home and promote sustainable development.” — President Xi Jinping at the COP15 Leaders’ Summit, October 12, 2021.

Over 2,000 years ago, Chinese philosophers already expressed concepts of harmony between humanity and nature: “All living things flourish together without harming one another” and “Heaven, Earth, and I live together, and all things and I are one.”

China’s Conservation Achievements

90% of terrestrial ecosystem types and 71% of nationally protected species are now effectively conserved.

Populations of rare species have rebounded:

Giant pandas increased from 1,114 (1970s–80s) to 1,864.

Hainan gibbons grew from 7–9 at their lowest point to over 30.

Crested ibises rose from 7 at discovery to over 5,000.

Tibetan antelopes recovered from tens of thousands to over 300,000.

On average, China discovers about 200 new plant species annually — one-tenth of the global total.

Policy and Leadership

“Lucid waters and lush mountains are invaluable assets” has become a widely accepted principle in Chinese society.

Since the 18th Party Congress, China has enacted or revised 20+ biodiversity-related laws, including the Forest Law, Grassland Law, Fisheries Law, Wildlife Protection Law, Environmental Protection Law, Marine Environment Protection Law, Seed Law, Wetland Law, Yangtze River Protection Law, and Biosecurity Law.

Integrated management of mountains, rivers, forests, farmlands, lakes, grasslands, and deserts has been implemented.

Since 2019, China has been the largest contributor to the CBD’s core budget.

6.4

Biodiversity Conservation in Action: Stories of Friendship Between Animals and Humans

As humanity’s co-inhabitants of Earth, wild animals not only maintain ecological balance but also bring joy by connecting people with nature. This has led to the establishment of numerous protected areas and ex-situ conservation programs to safeguard wildlife and expand endangered populations.

At the Qinling Giant Panda Research Center (Shaanxi Rare Wildlife Rescue Base), moving stories unfold daily between animals and people.

A Giant Panda’s “Cute Contrast”

Giant pandas, China’s national treasure, are rare and beloved worldwide. At the Qinling Four Treasures Science Park, visitors flock to the panda pavilion, where the bears’ playful eating routines fascinate crowds. Sitting comfortably, pandas use their paws — sometimes even all four limbs — to strip, tear, and chew bamboo endlessly, delighting tourists and earning admiration as symbols of biodiversity conservation success.

6.5

Remarkable Biodiversity Conservation Results: Rare Wildlife Thriving in Shandong

On May 20, at a press conference for the upcoming International Day for Biological Diversity, the Shandong Provincial Government highlighted local biodiversity achievements.

Deputy Director Guan Yanming of the provincial Department of Ecology and Environment showcased images and videos of species such as:

Animals and crops: Taihang red-scaled fish, Luxi cattle, wild soybean, Yantai apple, Laiyang pear.

Key Results

Over 85% of national key protected wild plants and animals in Shandong have been effectively conserved.

Shandong’s geography — backed by the continent, facing the sea, with diverse landforms — fosters rich biodiversity.

A province-wide biodiversity survey is underway, focusing on nine priority ecological regions, including:

Terrestrial: Yellow River Delta, Mount Tai–Mount Culai, Laoshan.

Marine: Bohai Bay and six other coastal areas.

Additional surveys include: wildlife resources, bird populations, fishery resources in lakes and reservoirs (e.g., Nansi Lake, Dongping Lake), aquatic life in rivers, and marine and coastal ecosystems.

Agricultural Genetic Resources

A census of crop, livestock, and aquaculture genetic resources has been completed at the provincial level, clarifying species, numbers, and distributions.

Follow-up steps include registering, evaluating, and cataloguing these resources.

6.6

Biodiversity Conservation | Xinhua Praises Shandong: Over 85% of Nationally Protected Species Effectively Conserved

In August 2021, the critically endangered spoon-billed sandpiper was first recorded in the Yellow River Delta; in March 2022, the critically endangered yellow-breasted bunting was discovered for the first time at the Dawen River ecological wetland in Anqiu, Weifang.

At a May 20 press conference, Deputy Director Guan Yanming of the Shandong Provincial Department of Ecology and Environment highlighted newly recorded species in recent years and presented the province’s biodiversity conservation progress.

He noted:

In 2021, Shandong designated 30 priority areas for biodiversity conservation.

In 2022, biodiversity surveys were carried out widely across these priority regions.

The province optimized and integrated protected areas, covering key biodiversity-rich zones, achieving effective protection for more than 85% of nationally protected wild plants and animals.

Additionally, by 2021, Shandong had completely eliminated Class V surface water quality bodies (the lowest category) across all monitored sections, improving ecological conditions to their best on record and providing a strong ecological shield for biodiversity.

6.7

International Day for Biological Diversity | What Do You Know About Biodiversity?

May 22 is the UN-designated International Day for Biological Diversity, aimed at raising public understanding and awareness.

What is biodiversity?

It is the ecological complex formed by organisms and their environments, including animals, plants, microorganisms, and the genes they carry, as well as the ecosystems they form. It includes genetic diversity, species diversity, and ecosystem diversity.

Species diversity refers to the richness and distribution evenness of biological species within a region. Forests and grasslands, for instance, have higher species diversity than deserts.

Genetic diversity refers to the total genetic information carried by organisms on Earth. Each species functions as a gene pool — the richer the genetic variety, the stronger the adaptability. Genetic mutations occur under environmental pressures, maintaining long-term evolutionary potential.

Ecosystem diversity refers to the variety of ecosystems’ composition, function, and processes, such as those seen in documentaries like Animal World. Species interact with each other and their environment, following the evolutionary principles of natural selection and survival of the fittest.

6.8

Ministry of Public Security Announces 10 Typical Cases of Crimes Damaging Biodiversity

Ahead of International Day for Biological Diversity (May 22), China’s Ministry of Public Security released details of 10 major biodiversity crime cases.

In recent years, law enforcement has intensified crackdowns on crimes involving:

Endangered wild animals

Nationally protected plants

Natural reserve destruction

Illegal hunting and fishing

Environmental pollution

Key results (Jan 2021 – Apr 2022):

28,000+ wildlife-related cases solved; 35,000+ suspects arrested.

13,000+ cases involving wild plants and illegal logging prosecuted; 12,000+ suspects arrested.

3,600+ environmental pollution crimes solved; 7,200+ suspects arrested.

Highlighted cases:

Qingdao, Shandong: Fei XX and others harming endangered wildlife.

Chongqing: Xiang XX and others illegally dumping hazardous waste.

Zhoushan, Zhejiang: Gu XX and others involved in illegal fishing.

These actions cut off illegal profit chains and provided strong legal safeguards for biodiversity.

6.9

Shandong Ecology Department: Deep Biodiversity Surveys and Solid Progress in Germplasm Resource Protection

On May 20, at a press conference marking International Day for Biological Diversity, Shandong provincial authorities presented their biodiversity conservation work.

Deputy Director Guan Yanming explained that:

During COP15 (Oct 2021, Kunming), the UN Development Programme (UNDP) selected Shandong as China’s only pilot province for its Biodiversity Finance Initiative (BIOFIN).

The project supports countries in building financial systems that enhance biodiversity protection by improving funding conditions and execution efficiency of biodiversity strategies and action plans.

Current efforts in Shandong:

Reviewing past biodiversity-related support policies and financial inputs.

Assessing future biodiversity financing needs.

Formulating a biodiversity finance plan to improve funding security.

Goal: Provide replicable experience for other provinces in China and contribute models globally.

6.10

Pooling Efforts to Advance Biodiversity Conservation — Yunnan Holds 2022 International Biodiversity Day Press Event

On May 20, 2022, the Yunnan Provincial Department of Ecology and Environment held a press conference introducing the Yunnan Online Biodiversity Museum and screening the promotional film Biodiversity of Yunnan. The online museum was officially launched at the event.

Context:

From Oct 11–15, 2021, COP15 Phase I was successfully held in Kunming under the theme “Ecological Civilization: Building a Shared Future for All Life on Earth.”

President Xi Jinping delivered a keynote speech “Building a Shared Future for All Life on Earth”, which provided guidance for global ecological civilization and biodiversity governance.

Key outcomes included: the landmark Kunming Declaration and the establishment of the Kunming Biodiversity Fund.

The short film “Elephants in Yunnan” screened during COP15 resonated globally, highlighting Yunnan’s rich biodiversity and sparking worldwide attention.

For 2022, the International Biodiversity Day theme “Building a Shared Future for All Life” aligned seamlessly with COP15’s vision, injecting momentum into Yunnan’s continued biodiversity initiatives.

**Climate change temporal pattern news**

2011.1.1-2013.12.31

1.

Climate Change Leads to Extreme Weather Changes

On the 28th, the Intergovernmental Panel on Climate Change (IPCC) released a report stating that evidence shows climate change has led to changes in the frequency, intensity, duration, and spatial extent of extreme weather events over the past half-century, including heatwaves, record high temperatures, and heavy rainfall. In some regions, extreme weather and meteorological events have increased.

NASA images show that in 1995, the Earth’s gravity showed significant differences, which were closely related to the phenomenon of melting ice sheets.

Global warming has long been regarded by some people in the scientific community as a “scandal in climatology.” These people believe that some scientists have made biased “edits” to climate data in favor of supporting the global warming theory. A recent report by the UK Met Office shows that global temperatures have indeed risen by 0.75°C since 1900, with the average temperature in 2010 being the highest.

The World Meteorological Organization (WMO) stated that from 2001 to 2010, the pace of global climate change accelerated, and that decade was also the hottest on record globally. The WMO noted that since 1971, global warming has become increasingly evident, with the global average temperature rising 0.166°C every 10 years compared to the previous decade.

In recent years, droughts, floods, severe cold, heatwaves, and other extreme and abnormal weather phenomena have frequently occurred around the world. While bringing adverse impacts on people’s production and life, they have also caused fear and panic psychologically. So, how should we view these weather and climate phenomena objectively and correctly from the perspective of meteorological science? Can the accuracy and practicality of weather forecasting be further improved in the future? Our reporter interviewed Zheng Guoguang, Director of the China Meteorological Administration.

2.

“Top Ten News on China’s Response to Climate Change and Low-Carbon Development 2011” Released in Beijing

On the morning of the 24th, the National Development and Reform Commission (NDRC) held a press conference in Beijing to release the “Top Ten News on China’s Response to Climate Change and Low-Carbon Development 2011.” Su Wei, Director-General of the Department of Climate Change of the NDRC, said at the conference that on December 1, 2011, the State Council officially issued the “12th Five-Year Plan for Controlling Greenhouse Gas Emissions”. This was the first time the State Council released a major document concerning the control of greenhouse gas emissions and the promotion of low-carbon development. It proposed to accelerate the transformation of the economic development model as the main line, firmly establish the concept of green and low-carbon development, take into account both domestic and international situations, and treat actively responding to climate change as a major national strategy for economic and social development. It is also regarded as a major opportunity for accelerating the transformation of the economic development mode, adjusting the economic structure, and promoting a new industrial revolution.

It emphasized adhering to a new path of industrialization, reasonably controlling total energy consumption, comprehensively using multiple means such as optimizing industrial and energy structures, saving energy and improving efficiency, and increasing carbon sinks to ensure the completion of the “12th Five-Year Plan” carbon intensity reduction target.

He stated that this selection fully respected public opinion and online voting by netizens, making the “Top Ten News” more credible and influential. It is of great significance to enhance public awareness of low-carbon action and to establish the concept of low-carbon development.

The event was specifically guided by the NDRC’s Department of Climate Change, with the Ministry of Science and Technology, the Ministry of Industry and Information Technology, the Ministry of Housing and Urban-Rural Development, and other departments participating in the guidance. It was jointly organized by China Economic Herald and the Editorial Committee of China Low-Carbon Yearbook, and co-organized by Tianjin Emissions Exchange. More than 220,000 netizens participated in the selection, which was also supported by expert review. At the press conference, the China Low-Carbon Yearbook 2011 was also launched. It is reported that the selection of the “Top Ten News on China’s Response to Climate Change and Low-Carbon Development” has been held for two consecutive years.

3.

On September 19, 2012, as the sun’s direct rays moved southward, scientists could finally breathe a sigh of relief: the massive Arctic sea ice melt of that year had finally stopped.

However, according to the U.S. National Snow and Ice Data Center (NSIDC), on September 16, the sea ice coverage reached its lowest point in 2012—1.32 million square miles, covering 24% of the Arctic Ocean’s surface. This was also the lowest point in observation history.

The previous record low was in 2007, when the coverage was 29%. In the late 1970s, after the summer melt, the coverage was about 50%.

There is no doubt that the melting of Arctic glaciers is accelerating. Scientists believe the main cause is human greenhouse gas emissions. On the other hand, glacier melting has created a positive feedback effect: glaciers reflect 85% of sunlight, whereas deep seawater reflects only 5% and absorbs more heat. This means that the less ice remains, the faster it melts.

Some even suggest that perhaps within eight years, by 2020, there may be almost no ice left in the Arctic Ocean in summer—not even ice floes.

“The Arctic is the Earth’s air conditioner. We are losing this air conditioner. It’s not just that polar bears may go extinct or that local people must adapt to new environments, which we have already seen—it’s about greater climate impacts,” said Walter Meier, a researcher at the NSIDC.

In recent years, reports of polar bears drowning have increased, as they are forced to swim long distances in icy waters in search of food.

4.

Extreme Weather Continues Across Multiple Countries, Food Prices Rise, Climate Change Causes Concern

Beijing, Aug 21 (China News Service) — Recently, many countries around the world have experienced continued extreme weather. While floods ravaged North Korea, the Philippines, and other countries, drought in parts of Europe and North America has become increasingly severe. Extreme weather has sparked new concerns about rising food prices and climate change.

According to KCNA on the 20th, influenced by the rainy season front, from the 17th to the 19th, many areas of North Korea, including the capital Pyongyang, again experienced heavy rains and flooding. The floods struck several of the country’s “granary areas.” Reports indicate the heavy rains would continue until the 21st. North Korea is currently stepping up various relief measures.

Japan’s Kyodo News reported that due to fronts and low pressure, Hokkaido and the Tohoku region recently experienced convective weather and torrential rains.

South Asia and Southeast Asia have recently been threatened by monsoons and typhoons. Typhoon Tembin struck northern Luzon in the Philippines, bringing heavy rainfall that caused floods and landslides. Since August, the death toll from floods and landslides in the Philippines has risen to 170, with more than 3 million people affected.

Typhoon Kai-tak also “refused to be outdone.” On the evening of the 17th, it made landfall in northern Vietnam, bringing strong winds and heavy rainfall, killing at least 27 people, damaging nearly 12,000 houses, and affecting 23,000 hectares of farmland.

While many Asian countries were fighting heavy rains and floods, many European countries were suffering from persistent high temperatures and frequent forest fires. Since late August, southern and central Italy have repeatedly issued red heat alerts. In France, 33 provinces were hit by heatwaves, with temperatures breaking historical records for the same period. Germany experienced its hottest day of the year on the 20th. Switzerland, the UK, and others were also under the grip of extreme heat.

5.

Research Shows: Future Global Warming May Lead to Worldwide Blackout Risks

Beijing, China Radio International — According to Reuters on the 4th, the latest scientific research shows that as global warming leads to reduced river flows, nuclear and coal-fired power plants in the United States and Europe may face shortages of cooling water, which would in turn increase the risk of global blackouts. In this hotter world, scientists need to rethink how to better produce electricity.

Global warming makes cooling water resources scarce for power plants

Data show a clear positive correlation between rising global temperatures and lower summer river levels. Based on this, a team of scientists from Europe and the U.S. studied how global warming would affect power plants that rely on river water for cooling.

In a research report released on the 4th, scientists predicted that due to a lack of cooling water, Europe’s coal and nuclear power generation capacity would decline by 4%–16% between 2031 and 2060, while the U.S. would see a 6%–19% decline. Even more worrying, the risk of total or large-scale shutdowns of power plants could increase threefold.

The project’s lead researcher, Dennis Lettenmaier, a professor of civil and environmental engineering at the University of Washington, warned that “we must re-examine our reliance on cooling.”

As is well known, coal, nuclear, and natural gas power plants all need to turn large amounts of water into steam to drive turbines and generate electricity, and any temperature changes in these water sources can affect power plant operations. Therefore, the impact of global warming on them will be enormous.

6

Frequent Extreme Weather, Rare Heatwaves May Signal Climate Change

In the past week, many European countries have experienced rare and extreme heatwaves, catching Europeans on vacation and foreign tourists in Europe somewhat off guard. Climate experts believe that a single extreme heatwave cannot be used as evidence of global warming, but the frequent occurrence of extreme weather is indeed a clear signal of changes in the Earth’s climate.

From August 17 to 20 were the most unbearable days for Europe. From Western Europe’s France and Germany, to Southern Europe’s Italy and Spain, to Central Europe’s Austria and the Czech Republic, the sun was scorching and the heatwaves oppressive. Residents chose various ways to escape the heat in the late summer.

Normally, Europe’s summer begins in mid-July and basically ends by late August. Europeans, who like to enjoy the sunshine, often choose to go to sunny places such as the seaside in August. High temperatures above 30°C are not common in European summers, and even if they occur, they are usually between late July and early August. This time, however, a prolonged period of extreme heat appeared in mid-to-late August, which meteorological departments consider to be very rare.

Europeans suffering through this heatwave inevitably linked such extreme weather with global warming, but quite a few climate and meteorological experts believe that this sustained extreme heat across many countries should not be simply regarded as evidence of global warming.

Jean-Pascal van Ypersele, professor at the Catholic University of Louvain in Belgium and Vice-Chair of the IPCC, told Xinhua reporters in an interview that this extreme heatwave affecting almost all of Europe did not last longer than the heatwave of the summer of 2003, which nine years earlier had caused about 50,000 deaths across Europe.

7

The Tragedy of Climate Change

The Earth’s suitable climate and environment have been an important reason for its nurturing of life and human civilization. However, since its birth, the Earth’s climate has never ceased to change. Climate change affects the evolution of the natural environment, and it also affects human life.

A report released by the IPCC in March 2012 stated that evidence shows climate change has led to changes in the frequency, intensity, duration, and spatial extent of extreme weather events over the past half-century, including heatwaves, severe cold, droughts, and heavy rainfall. In some regions, extreme weather and meteorological events have increased. Extreme weather, along with a series of non-extreme weather events (including climate changes caused naturally and by humans), combined with deficiencies and shortcomings in disaster prevention capabilities, can together lead to risks of disasters.

However, as a member of the Earth, humanity can reduce the impacts of these risks by formulating appropriate policies for prevention, preparedness, response, and recovery, thereby enhancing people’s ability to cope with extreme weather and events.

This was on October 15, 2011, in a suburban area of Bangkok, Thailand, where a boy swam through a street covered by floodwaters. In order not to wet the banknotes, he tried hard to keep his arm out of the water. Since mid-July 2011, Thailand had experienced the worst floods in 50 years. The floods caused hundreds of deaths and affected 2.9 million people.

The Earth’s suitable climate and environment have been an important reason for its nurturing of life and human civilization. However, since its birth, the Earth’s climate has never ceased to change. Climate change affects the evolution of the natural environment, and it also affects human life. A report released by the IPCC in March 2012 stated that evidence shows climate change has led to changes in the frequency, intensity, duration, and spatial extent of extreme weather events over the past half-century.

8

Scientists from Many Countries Say Last Year Extreme Weather Prevailed Globally, Will Intensify in the Future

Beijing, July 12 (China News Service) — According to Singapore’s Lianhe Zaobao on the 12th, a report titled State of the Climate jointly issued by scientists from 48 countries pointed out that extreme weather prevailed in 2011, with severe droughts, floods, and heatwaves sweeping across the globe. Rising greenhouse gas emissions mean that the world will face more extreme weather in the future.

Nearly 400 scientists from 48 countries published the 22nd annual State of the Climate report in the Bulletin of the American Meteorological Society. They said 2011 was one of the 15 hottest years since the 19th century, when scientists began recording weather. Severe drought occurred in Texas, USA, and unusual high temperatures appeared in England in November.

Scientists from NOAA and the UK Met Office pointed out that overall, 2011 was full of extreme climate events: historic droughts in East Africa, northern Mexico, and the southern United States; a higher-than-average number of hurricanes in the North Atlantic; and Australia experienced its wettest two years on record.

The report also stated that Arctic warming is occurring at twice the average rate of the rest of the planet, and Arctic sea ice shrank to its second smallest extent on record.

Global greenhouse gas concentrations continued to rise last year, with the average concentration of CO₂ surpassing 390 parts per million for the first time, an increase of 2.10 compared with 2010—the first such increase on record.

When investigating the causes of extreme weather, scientists did not directly attribute it to global warming, but pointed to climate phenomena such as La Niña. The report also analyzed how human-induced climate change influenced six major climate events, including droughts in Africa and the United States, as well as warm weather in the UK. In November last year, the temperature in central England was the second highest since 1659.

9

China’s Top Ten News on Responding to Climate Change and Low-Carbon Development in 2011 Announced

Beijing, Feb 24 (China News Service) — On February 14, the press conference for the release of “China’s Top Ten News on Responding to Climate Change and Low-Carbon Development in 2011” and the launch of the China Low-Carbon Yearbook 2011 was held at the China Science and Technology Hall. Su Wei, Director-General of the Department of Climate Change of the NDRC, attended the event and announced the Top Ten News of 2011.

The Top Ten News of 2011 are as follows:

The “12th Five-Year Plan” Outline emphasizes responding to climate change and low-carbon development.

Comment: On March 17, the Fourth Session of the 11th National People’s Congress passed the Outline of the 12th Five-Year Plan for National Economic and Social Development of the PRC, which regarded actively responding to climate change and accelerating low-carbon development as one of the key policy directions for economic and social development during the 12th Five-Year Plan period. “Actively respond to global climate change” appeared in the first chapter of Part Six “Green Development: Building a Resource-Conserving and Environmentally Friendly Society,” highlighting the country’s emphasis on climate change.

The Outline pointed out: Faced with increasingly severe constraints on resources and the environment, we must strengthen crisis awareness and establish a concept of green and low-carbon development. We must adhere to giving equal emphasis to mitigation and adaptation to climate change, fully leverage technological progress, improve systems and policies, and enhance climate change response capabilities. The Outline also clearly put forward requirements for “controlling greenhouse gas emissions,” “enhancing adaptation to climate change,” and “extensively carrying out international cooperation.”

The State Council issued the “12th Five-Year Plan on Controlling Greenhouse Gas Emissions”, setting overall requirements and main targets for China’s control of greenhouse gas emissions by 2015.

10

People’s Daily: Extreme Weather Makes the World Uncomfortable

Meteorological departments of many countries forecast that on August 2, two typhoons would affect Asia. Super Typhoon Saola left the Philippines and approached northeast Taiwan, China. Typhoon Damrey was expected to sweep past northern Shanghai before heading straight to southeastern Japan. Southern China would experience strong monsoon rainfall, which would also extend to Southeast Asia and eastern India.

Recently, extreme weather such as high temperatures, heavy rains, and droughts have frequently occurred worldwide, and the resulting natural disasters have become increasingly frequent and severe. The WMO has repeatedly reported in recent years that the frequent occurrence of extreme weather and climate events is highly unusual. Countries should actively take countermeasures to minimize disaster losses.

Heavy casualties and property losses, direct impact on economic development

The Philippine National Disaster Risk Reduction and Management Council confirmed on August 1 that Saola had brought storms and rains for several consecutive days to Manila, the capital, and surrounding areas, killing 12 people and forcing the evacuation of more than 145,000 people.

Since late June, multiple floods and landslides caused by heavy monsoon rains occurred in many parts of India and Bangladesh, causing heavy casualties and property losses. Reports said that torrential rains triggered by the monsoon in northeastern India had caused 124 deaths, forced 6 million people to leave their homes, and left more than 2 million people homeless. The Indian government stated that this was the most serious flood since 1998. Bangladesh also said that the country had suffered the most severe rainfall in years, with at least 100 deaths and 400,000 people affected.

In mid-July, Japan’s Kyushu region was hit by torrential rains, leaving 30 dead and 2 missing. Later in the month, the entire country suffered persistent heat, and between July 23 and 29 alone, 16 people died from heatstroke, about 50% more than the previous week, marking a new high for the summer.

Since July 18, after entering the rainy season, North Korea’s rainfall increased in most parts of the country. According to the latest statistics released by North Korean media as of July 28, a total of 88 people had died nationwide, more than 5,000 houses were completely or partially destroyed, and more than 12,000 households were flooded.

2014.1.1-2016.12.31

1

China Achieves Major Progress in Addressing Climate Change

On the eve of the 22nd Conference of the Parties (COP22) to the United Nations Framework Convention on Climate Change, the China’s Policies and Actions on Climate Change (2016 Annual Report) was officially released on November 1. At a press conference held by the State Council Information Office, China’s Special Representative for Climate Change Affairs, Xie Zhenhua, stated that the Chinese government attaches great importance to addressing climate change. Since the “12th Five-Year Plan,” China has made major progress in advancing its climate change response work. The Marrakech conference is the first COP after the Paris Agreement came into effect, and China hopes it will be a meeting focused on implementation.

Data show that during the “12th Five-Year Plan” period, China’s carbon intensity fell cumulatively by 20%, exceeding the target of 17% set in the plan. The energy structure was further optimized, with non-fossil energy accounting for 12% of primary energy consumption in 2015, exceeding the target of 11.4%. Forest stock volume increased to 15.137 billion cubic meters, achieving ahead of schedule the 2020 target for increased forest stock.

Xie Zhenhua said that the Chinese government actively participates in negotiations under the UNFCCC, firmly upholds the principles and framework of the Convention, and continuously strengthens comprehensive, effective, and sustained implementation of the Convention. In addition, China actively supports developing countries in improving their capacity to address climate change. Through the establishment of the South-South Cooperation Fund on Climate Change, since the “12th Five-Year Plan” period the Chinese government has contributed a total of 580 million yuan, providing material and equipment assistance to small island states, least developed countries, and African countries.

2

Scientific Report Says Climate Warming Intensifies, More Rain and Snow Worldwide

Beijing, March 10 (China News Service) — According to foreign media, a scientific report states that as global warming intensifies, extreme rainfall and snowfall events will increase in most parts of the world, including many arid regions.

According to reports, a research team from the University of New South Wales in Australia recently published a report in Nature Climate Change stating that because warm air contains large amounts of water vapor, global warming will increase extreme precipitation events.

The team collected and analyzed data from about 11,000 meteorological observation stations worldwide between 1951 and 2010.

The results show that during this period, in arid regions such as western North America, Australia, and parts of Asia, both annual precipitation and extreme precipitation events increased by 1% to 2% every decade.

In wetter regions such as eastern North America and Southeast Asia, extreme precipitation events also showed similar growth trends, with annual precipitation slightly increasing.

One of the authors of the report, Donat, said that whether in wet or dry regions, an upward trend in precipitation can be seen. Therefore, governments have the necessity to increase investment in flood prevention and related infrastructure.

3

[Decoding G20] Climate Change — How the G20 Responds

UN Secretary-General Ban Ki-moon told CCTV reporters that the G20 Hangzhou Summit was the first time in history that climate change was included in the G20 Action Plan. The Chinese government’s measures in addressing climate change will play an important leading role in the global implementation of the Sustainable Development Agenda.

So, how was the climate change issue presented at the Hangzhou Summit? What contributions did China make? Let’s look at Decoding G20.

The recently concluded Rio Olympics in Brazil remains unforgettable to many people. In the opening ceremony performance, a young boy stood blankly on stage, while the large screen beside him displayed this message: our planet has experienced record-breaking high temperatures for 14 consecutive months. With greenhouse gas emissions continuing to rise, global average temperatures continue to climb, almost breaking the record for the hottest year every year. Global warming has caused glaciers at the North and South Poles to melt and sea levels to keep rising. Perhaps in the near future, Amsterdam in Europe, Lagos in Africa, Rio in the Americas, and Shanghai in China will all be flooded and eventually disappear… The Rio Olympics used this form of expression to call for global attention to climate change.

The WMO’s latest State of the Climate statement shows that 2015 was the hottest year since modern observations began. Temperatures in parts of central and western Europe even exceeded 40°C. Rare forest fires broke out in the northwestern United States and western Canada, with more than 2 million hectares of forest burned in Alaska alone during the summer. And in July, the WMO released another media briefing, pointing out that in June this year, the global land and sea surface temperature was 0.9°C higher than the 20th-century average, with frequent heatwaves and extreme weather events.

4

Climate Change Causes Persistent Warming in the Arctic Circle — Experts: Extremely Unusual

According to foreign media reports, scientists said on the 24th that the “vicious cycle” of climate change is intensifying ocean warming and strengthening southerly winds, leading to abnormally high temperatures in the Arctic Circle. Data from the Danish Meteorological Institute show that over the past four weeks, temperatures over the Arctic ice sheet have consistently been 9 to 12°C higher than average.

It is reported that the Denmark-based institute tracks weather changes in the Arctic Circle hourly. Climate researcher Stendel said that last week, for several days, the temperature above the Arctic ice sheet was a warm 0°C, a full 20°C higher than the typical mid-November temperature.

Stendel stated that this was “the highest temperature ever recorded since the satellite data era began in 1979.” He said: “What we are observing is extremely unusual.”

At this time of year, the open waters of the Arctic Ocean should freeze again after summer ice melt, with thousands of square kilometers of sea water freezing every day. But Stendel said this did not happen, or at least the freezing rate was different from previous years. He said: “Not only did the ice fail to increase as usual, but the influx of warm air also caused much sea ice to melt.”

5

WMO: Extreme Weather Closely Linked to Global Warming

On November 8, the World Meteorological Organization (WMO) released a report titled The Global Climate 2011–2015, analyzing the hottest five-year period on record and the human footprint in extreme weather events.

The report stated that 2011–2015 was the hottest five-year period on record, with the average temperature 0.57°C higher than the baseline period of 1961–1990. Among these, 2015 was the hottest year on record, and it was also the first year in which global temperatures exceeded pre-industrial levels by more than 1°C. The report also analyzed whether human-induced climate change is directly linked to extreme events. Citing 79 studies published in the Bulletin of the American Meteorological Society between 2011 and 2014, the report noted that more than half confirmed that human-induced climate change has driven the occurrence of extreme weather events. Some studies pointed out that the probability of extreme heat events increased by tenfold or more as a result.

The report pointed out that Arctic sea ice continues to decline. The 2011–2015 average extent was 28% lower than 1981–2010, and the minimum summer ice extent in 2012 was the lowest on record. In addition, the surface of the Greenland ice sheet continued to melt at above-average rates in summer, and mountain glaciers also continued to shrink. The report also emphasized that ocean warming causes water expansion and leads to global and regional sea level rise. A series of studies concluded that continental ice sheets, especially Greenland and West Antarctica, are accelerating sea level rise.

The UNEP released a report stating that in 2015, the UN system emitted 2 million tons of CO₂ equivalent greenhouse gases. The data came from 66 UN entities, covering more than 284,482 staff members worldwide. Among them, facilities and equipment emissions accounted for 46%, air travel emissions 40%, and other travel 14%. The United Nations has been striving to reduce greenhouse gas emissions.

6

Why Does China Continue to Promote South-South Cooperation and Foreign Aid on Climate Change Despite Economic Downturn?

First of all, scientific understanding of climate change can be traced back about 100 years. At the end of the 19th century, scientists had already realized and raised this issue. After a century of development, climate change truly developed into a mature discipline. Over this hundred years, disputes have always existed around the scientific basis of climate change, and many uncertainties still remain today.

But scientifically, skeptical voices are now becoming fewer. The United Nations specially established the Intergovernmental Panel on Climate Change (IPCC), which periodically evaluates scientific progress on climate change and provides the latest scientific support for the political process of responding to climate change.

The United Nations Framework Convention on Climate Change (UNFCCC), which came into effect in 1994, was the most important milestone in the process of addressing climate change, marking the point at which the international community began to truly take action. Later, the Kyoto Protocol of 1997 further specified quantified emission reduction targets for developed countries. Up until that time, the international community still held an extremely optimistic attitude about solving the problem of climate change.

However, with the rapid development of China and other emerging developing countries, some countries such as the United States realized that adopting emission reduction measures would affect their economic competitiveness. In 2001, the United States announced it would refuse to ratify the Kyoto Protocol. The entire process of addressing climate change suffered a severe setback and entered a downturn.

After the economic crisis of 2007–2009, some developed countries found that they had to look for new sources of economic growth. The shale gas revolution in the United States and the large-scale use of renewable energy in the European Union made people believe that the green economy could become a new feasible model for economic growth. Some newly emerging developing countries also began to face constraints in resources and energy, as well as worsening environmental degradation, and thus hoped to seek a path of low-carbon development.

7

Significant Climate Changes Have Already Occurred in China

On the occasion of World Meteorological Day on March 23, Zheng Guoguang, Director of the China Meteorological Administration, delivered a speech entitled Scientific Understanding of Climate and Attention to Climate Security. In his speech, he said that since the mid-20th century, China’s climate has undergone significant changes.

Zheng Guoguang believed that climate change has a huge impact on China. Yields of major crops such as wheat, corn, and soybeans are declining; the runoff of major rivers is decreasing or becoming unstable; soil erosion, ecological degradation, and species migration are worsening; atmospheric environmental capacity is decreasing; the development and utilization of wind and solar resources are constrained; and the safe production and operation of major strategic projects such as the Qinghai–Tibet Railway, the Three Gorges Reservoir, the South-to-North Water Diversion Project, the West-to-East Gas Transmission Project, and the Three-North Shelterbelt Project are under serious threat. Continued large emissions of greenhouse gases will lead to further global warming, and the risks of climate change and climate disasters that China faces may further intensify.

“Faced with the current and future challenges of climate change, we need to establish the concept of respecting nature, adapting to nature, and protecting nature, and enhance our awareness of climate security,” Zheng Guoguang said. He emphasized the need to strengthen energy conservation and emission reduction, follow the path of low-carbon development, leave behind blue skies, green lands, and clear waters for future generations, take more proactive actions to adapt to climate change, and comprehensively enhance disaster risk management capacity.

8

Climate Change Events Around the World in 2016

If one paid attention to the news, 2016 saw many topics on glacier melting, global climate anomalies, and air pollution. Foreign media outlet BuzzFeed selected a series of photos showing climate change in different parts of the world in 2016. Here are 15 of them:

Australia

Scientists investigating the Great Barrier Reef found that rising seawater temperatures, turbid water, and bacterial infections caused massive coral bleaching. Nearly 70% of the northern section was affected, and recovery could take 10–15 years.

Canada

Sea ice in the Arctic is extremely important for polar bears, but researchers noted that winter sea ice formation has slowed. In Hudson Bay, Canada, the number of polar bears declined by 20% over the past 30 years.

China

In December, Beijing issued a “red alert,” restricting traffic and suspending classes and work.

France

Paris experienced its worst air pollution in the past decade in 2016.

India

In November, New Delhi’s air pollution index reached 999, making it the most polluted city in the world that day. According to the standard, an index of 500 is already classified as “hazardous.”

Switzerland

The Aletsch Glacier, the country’s largest glacier, is about 23 kilometers long and 900 meters deep, but it is rapidly shrinking. Experts predict that within this century, it could lose 90% of its ice.

Israel

The water level of the Dead Sea is dropping by one meter every year. Researchers said that if this continues, it will completely dry up by 2050.

Nicaragua

A photo taken by Oswaldo Rivas in 2008 shows a lake in a nature reserve, but this year the lake has dried up.

Norway

In the past 30 years, summer temperatures in Ny-Ålesund have risen by 11 degrees, causing significant glacier loss.

Antarctica

NASA and researchers at the University of California recently detected that glaciers in West Antarctica are continuously melting, directly leading to sea-level rise and possibly becoming irreversible.

9

New Report Reveals Links Between Multiple Extreme Weather Events and Climate Change

A new report completed by international researchers on the 15th stated that from heatwaves in East Asia, to wildfires in Alaska, to unusually long winter sunshine in the UK, many extreme weather events in 2015 were linked to climate change caused by human greenhouse gas emissions.

A total of 116 scientists from 18 countries including the United States, the United Kingdom, and China published a report titled Explaining Extreme Events from a Climate Perspective in the Bulletin of the American Meteorological Society. The report analyzed more than 20 weather events around the world in 2015. The results found that climate change played an amplifying role in many temperature-related extreme weather events, but had little influence on most extreme rainfall events.

In general, climate change is believed to increase the frequency of extreme weather events, but scientists usually avoid linking it to any single event. In the latest research, scientists combined historical observational data with climate models to determine whether and how climate change affected specific weather events.

The report said that 10 extreme high-temperature events in 2015, including heatwaves in Europe, India, Pakistan, China, Indonesia, and Australia, were made more severe by climate change. The record low Arctic sea ice extent in March last year and the large-scale wildfires in Alaska were also influenced by climate change.

In addition, the probability of tidal flooding on sunny days in the Miami area of the United States has increased by more than 500% since 1994; the probability of long winter sunshine events in the UK, like in 2015, has also increased by 1.5 times due to climate change.

However, some other extreme weather events in 2015 had little to do with climate change. These included the delayed spring rainy season in Nigeria, the strongest rainfall in a century in Chennai, India, and the extremely cold winter weather in the northeastern United States and Canada.

10

Academician of the Chinese Academy of Sciences: Global Change Characterized by Warming Is a Fact

At 15:00 on March 7, during the Fourth Session of the 12th CPPCC National Committee, a press conference was held in the multi-function hall of the Media Center. Five CPPCC members were invited to answer questions on issues such as comprehensively conserving and efficiently utilizing resources, promoting low-carbon and circular development, strengthening environmental management, especially the prevention and control of air, water, and soil pollution, enhancing ecological protection and restoration, using natural resources in an orderly and measured manner, and improving the overall quality of the ecological environment.

China Meteorological News Agency: Question to Committee Member Qin Dahe — Now that addressing climate change has become a national action, some members of the public and even some experts in the scientific community remain skeptical, believing that climate change is a pseudo-proposition and that global warming has plateaued. How should we view this issue?

Qin Dahe: Global change characterized by warming is an indisputable scientific fact. This is both an ancient and advanced discipline, and there is no issue of it being a pseudo-proposition. I would like to explain further: because science popularization has not been sufficient, many people do not have a clear concept. We must distinguish between weather, climate, and climate change.

What is weather? After CCTV’s nightly news broadcast, there is a weather forecast — this is short-term atmospheric phenomena: wind, rain, temperature.

What is climate? Climate is the average state of weather over a relatively long period. The World Meteorological Organization explicitly stipulates that 30 years constitutes one cycle. The average state from 1961 to 1990 is called the “average climate.” Has the climate of a place changed? We compare current weather elements with the 30-year average. The deviation from the average is called an “anomaly.”

Contemporary climate change does not only mean rising or falling temperatures. So-called climate change refers to changes in the climate system. The five spheres — atmosphere, hydrosphere, lithosphere, biosphere, and cryosphere — are all undergoing changes. Thus, global warming does not merely mean rising temperatures; it also means glaciers in high mountains are retreating, the ice sheets at the poles are melting faster, sea ice coverage is shrinking, biodiversity is decreasing, sea levels are rising — all of these are quantitative indicators of global warming.

2017.1.1-2019.12.31

1

China’s Work on Addressing Climate Change Has Achieved Remarkable Results

At the press conference held by the State Council Information Office on November 27 to release the China’s Policies and Actions for Addressing Climate Change – 2019 Annual Report (hereinafter referred to as the “Annual Report”), it was learned that in 2018, China’s carbon emission intensity decreased by about 4% year-on-year, and dropped cumulatively by 45.8% compared with 2005, which is equivalent to reducing 5.26 billion tons of carbon dioxide emissions. The share of non-fossil fuels in total energy consumption reached 14.3%. This basically reversed the rapid growth trend of greenhouse gas emissions, and remarkable results were achieved in addressing climate change.

This year marks the 11th release of the Annual Report. The contents of the report cover eight aspects, including mitigation of climate change, adaptation to climate change, planning and institutional building, basic capacity, broad participation of the whole society, active participation in global climate governance, strengthening international cooperation and exchanges, and China’s basic positions and propositions at COP25 of the UNFCCC. It comprehensively reflects China’s policies, actions, and work in the field of climate change since last year.

It is understood that since last year, China has actively addressed climate change. First, climate change mitigation work has been comprehensively advanced. In 2018, national carbon emission intensity fell by 45.8% compared with 2005, maintaining a continuous decline. This figure has already achieved ahead of schedule the commitment that by 2020 carbon emission intensity would drop by 40–45% compared with 2005. The rapid growth trend of greenhouse gas emissions was basically reversed. Second, adaptation to climate change was carried out in an orderly manner. Substantial work has been done in agriculture, water resources, forests, oceans, human health, and disaster prevention and mitigation, achieving positive progress. Third, systems and mechanisms for addressing climate change have been continuously improved. A working mechanism has taken shape featuring unified leadership by the National Leading Group on Climate Change and Energy Conservation and Emission Reduction, overall management by competent departments, coordination among departments, and full participation of local governments. Fourth, construction of the carbon market has been continuously promoted.

2

Earth, Are You Okay? A Review of Global Climate Events in 2019

In 2019, climate change worsened globally. Amazon, California, and Australia wildfires, air pollution in India, Hurricane Dorian, Antarctic glacier melting—global environmental problems have become increasingly severe. Let us review the climate and environmental news around the world in 2019.

The massive fires that started in the Amazon this August destroyed large areas, burning the rainforest at the rate of three football fields per minute. This triggered strong protests worldwide and disputes between Brazilian President Bolsonaro and European leaders.

Delhi and other northern Indian cities were shrouded in toxic smog, with pollution levels 20 times higher than the World Health Organization’s safety standard. India’s largest landfill—the Ghazipur landfill near New Delhi—rises by 10 meters every year. By 2020, the height of the landfill will exceed that of the Taj Mahal.

Hurricane Dorian caused severe damage in the Bahamas, killing more than 60 people and leaving hundreds missing.

California experienced devastating wildfires, burning down homes and forcing tens of thousands to flee.

Australia, including Sydney and other regions, was ravaged by bushfires nationwide. The country’s national treasure—the koala—faced the risk of extinction. Scientists linked the severity of the fires to climate change.

Mozambique was hit by two deadly cyclones. The storms destroyed the homes and crops of millions of people, causing losses of about USD 3 billion, accounting for 20% of the country’s GDP.

According to a report by the World Meteorological Organization, the Antarctic Peninsula is one of the fastest-warming regions in the world. Antarctica has even become a luxury tourist destination, but some people question whether tourists should visit Antarctica.

3

Climate Change Drives Millions onto the Streets – How Serious Is the Truth?

At the Climate Action Summit opened on September 23, scientists from the Intergovernmental Panel on Climate Change (IPCC) added more alarming evidence about global warming: if high greenhouse gas emissions are maintained, global sea levels will rise by 1.1 meters by 2100 and 5.4 meters by 2300, and these changes are caused by human activity itself. The enormous impact of climate change on the Earth’s environment and human life will be immeasurable.

Global warming is a topic that cannot be avoided. As climate change intensifies, we will hear more and more bad news brought about by global warming: shocking reductions in fish resources, massive coral reef deaths, rising sea levels year by year, and hundreds of millions of people suffering from climate-related disasters…

Millions Take to the Streets to Protest Climate Change

Before the UN Climate Action Summit opened on September 23, climate strikes took place in more than 150 countries. The global mobilization on Friday, September 20, appeared on the front pages worldwide, and the escalating actions throughout the week reached a climax on Friday, September 27, the second Global Day of Action. From September 20 to 27, a record-breaking 7.6 million people took to the streets demanding urgent action on climate change.

From Jakarta to New York, from Karachi to Amman, from Berlin to Kampala, from Istanbul to Quebec, from Guadalajara to Asunción, in big cities and small villages, millions of people around the world joined hands and raised their voices to protect the climate. They demanded a phased-out use of fossil fuels, control of burning and deforestation in the Amazon rainforest and Indonesia, and an immediate transition to renewable energy.

4

Press Conference on the Release of the 2019 Annual Report of China’s Policies and Actions for Addressing Climate Change

China has always attached great importance to addressing climate change. President Xi Jinping has repeatedly emphasized that addressing climate change is not something others are asking us to do, but something we ourselves need to do. It is an inherent requirement for China’s sustainable development, and also a responsibility for promoting the building of a community with a shared future for mankind.

At the National Conference on Ecological and Environmental Protection, he clearly pointed out the need to implement the national strategy of actively addressing climate change, and to promote and guide the establishment of a fair, reasonable, cooperative, and win–win global climate governance system. In July this year, Premier Li Keqiang presided over a meeting of the National Leading Group on Climate Change and Energy Conservation and Emission Reduction, studying and deploying key tasks in the field of climate change. Recently, the Fourth Plenary Session of the 19th CPC Central Committee pointed out the need to improve legal systems and technical guidance for green production and consumption, advance market-oriented green technological innovation, and more consciously promote green, circular, and low-carbon development.

For many years, China has regarded addressing climate change as an important starting point for ecological civilization construction, promoting high-quality economic development, and building a beautiful China. It has taken proactive actions to address climate change. Since 2018, localities and departments have adhered to Xi Jinping’s thought on ecological civilization, implemented the deployment and requirements of the National Conference on Ecological and Environmental Protection, and jointly promoted new progress in addressing climate change.

On the basis of systematically summarizing the work of addressing climate change, we organized and compiled the China’s Policies and Actions for Addressing Climate Change – 2019 Annual Report. This work began in 2009 and has been published continuously for 10 years. This year marks the 11th release.

5

In 2019, Global Warming Is Quietly Changing the Face of the Earth

Glacier melting has altered the Arctic landscape, rising sea levels are slowly making cities disappear, and the destruction of habitats may drive clownfish to extinction…

Did you know? Global warming is quietly changing the “appearance” of the Earth.

Glacier Melting Alters Landscapes – Cities Are Disappearing?

In November 2019, Venice, Italy, experienced its most dangerous week since 1872. The entire “water city” was completely flooded, suffering “apocalyptic destruction.” Research has shown that with global warming, Venice may be completely submerged within the coming decades, disappearing altogether.

Although the floodwaters in Venice have receded, the waters in the coastal Alaskan town of Kivalina have not. Due to rising sea levels, the area of this town is shrinking. By 2025, it will be submerged by seawater.

Cities inhabited by humans may be inundated and disappear just like that. The culprit behind all this may well be the massive melting of glaciers in the Arctic and Antarctic caused by climate warming.

These glaciers, once quietly “asleep” at the poles, majestic and beautiful, are now slowly melting away due to rising temperatures. As the glaciers retreat, the islands hidden beneath them are revealed, and the Arctic landscape is quietly changing.

Scientists have warned that the melting speed of large ice sheets in Greenland within the Arctic Circle is much faster than expected. By the end of the 21st century, it may put hundreds of millions of people worldwide at risk of flooding.

Behind glacier melting lies accelerating warming. Since the 1990s, the Arctic has warmed at twice the speed of the rest of the planet. The past six years have been the hottest six years in the Arctic.

In Russia on the edge of the Arctic Circle, climate zone boundaries in textbooks may even need to be revised. The capital Moscow, known for its severe cold, broke its December temperature record of the past 133 years, and due to little snowfall, it missed out on a white Christmas.

6

[Commentary Line] Focusing on Global Climate Change to Demonstrate “China’s Responsibility”

Against the backdrop of profound changes in the global political and economic landscape, China has adhered to energy conservation and emission reduction, taken the path of green development, and insisted on advancing ecological civilization and building a sound ecological environment. This not only demonstrates the spirit of responsibility of a major country, but also provides important reference for other nations in exploring the path of sustainable development.

It is worth noting that recently, a new session of the United Nations Climate Change Conference opened in Madrid, the capital of Spain. Delegates will discuss issues such as the outstanding details of the implementation rules of the Paris Agreement and financial arrangements. We see that at this conference, the “Chinese solution” once again became the focus of attention of the participating countries.

For China, ecological and environmental protection has long been an enduring theme. As early as 1978, the concept of environmental protection was included in the Constitution for the first time, stating that “the state protects the environment and natural resources and prevents and controls pollution and other public hazards.” In 2012, “Beautiful China” was written into the report of the 18th CPC National Congress, incorporating ecological civilization construction into the “Five-in-One” overall layout. In 2017, the report of the 19th CPC National Congress listed “ecological civilization” as an important component of the new journey of socialist modernization. Without doubt, the construction of ecological civilization highlights the greatness of “China’s governance.”

In recent years, special laws and regulations such as the Environmental Protection Law and the Law on the Prevention and Control of Air Pollution have been successively revised, and relevant intra-Party regulations on environmental protection have been frequently issued, such as the Regulations on Central Ecological and Environmental Protection Inspections and the Measures for Holding Party and Government Leaders Accountable for Ecological and Environmental Damage. It can be said that the role of China’s system is both normative and long-lasting. The systematic construction of the ecological civilization system will surely provide a solid guarantee for the sustainable development of Chinese society and for the building of a community with a shared future for mankind.

7

Hot, Hot, Hot! The Hottest June in 140 Years – Experts: Related to Climate Change

A heatwave swept through Paris, France. On June 29, people cooled off by playing in the fountains near the Trocadéro by the Eiffel Tower. (Photo source: Phys.org)

If you thought last month was hot, with watermelon, popsicles, air conditioning, and electric fans all needed, and if that didn’t work, just staying indoors—then you are not alone in battling the heat! According to a recent report by the U.S. science website Live Science, data from the U.S. National Oceanic and Atmospheric Administration (NOAA) show that June 2019 was the hottest June on record, and Antarctic sea ice extent also hit a new low that month.

Experts said that such extreme temperatures are related to climate change, and people should find ways to reduce greenhouse gas emissions, otherwise extreme heat events may become more frequent.

The Hottest June in 140 Years

NOAA data indicate that in June 2019, the average temperature of land and ocean combined was 0.95°C higher than the global average temperature (15.5°C). This made June 2019 the hottest June in the 140 years since NOAA began recording temperatures in 1880. Moreover, 9 of the 10 hottest Junes have occurred after 2010.

According to USA Today, NASA’s Goddard Space Flight Center, the Japan Meteorological Agency, and the EU’s Copernicus Climate Change Service also believe that June this year was the hottest June on record.

In the U.S., since temperature records began in Alaska in 1925, this June was the state’s second hottest June. Although Hawaii is almost always hot, this year the islands also experienced their hottest June on record. The Gulf of Mexico also had its hottest June in 110 years.

Europe was no exception. Austria, Germany, and Hungary all experienced their hottest June on record. Switzerland and France recorded their 2nd and 5th hottest Junes, respectively.

8

Scorching Summer – Overseas Data News Let You Feel Global Warming Directly

This summer, did you also feel unbearably hot? On July 25, a Paris weather station recorded a maximum temperature of 42.6°C, breaking the city’s 72-year record. The U.S. National Oceanic and Atmospheric Administration also confirmed with data: we just experienced the hottest June since weather records began. The issue of global warming is urgent, and more and more data journalism outlets have made it a focus of their reporting.

Results show that the record “2018 was the hottest year in Europe since 1900” may soon be broken. From January to the end of May, temperatures in some cities were much higher than the average from 1970 to 2000. In Warsaw, Kraków, and Tallinn, average temperatures in 2019 were 2.5°C higher than the same period at the end of the 20th century.

Higher temperatures disrupted lifestyles across Europe, affecting humans, animals, and plants. However, in the face of the already visible impacts of climate warming, some European cities have been slow to respond. During the 2018 heatwave, dozens of Europeans died of dehydration and heatstroke. Notably, however, no central government kept exact records of how many people died from high temperatures.

Urban areas are home to three-quarters of Europeans, and they are on the frontlines of climate collapse. Concrete and asphalt absorb heat during the day and release it at night, making cities several degrees hotter than surrounding areas. For urban residents who, due to poverty, cannot afford cooling devices such as air conditioning, local governments’ adaptation policies are truly a matter of life and death.

9

Latest Research Shows: The Global Warming Crisis May Double! Tropical Rainforests May Turn into Savannas

Reports indicate that many parts of the Northern Hemisphere are currently experiencing an unusual global heatwave, with temperatures in many places breaking historical records.

This widespread and unusual heatwave has raised concerns—does this represent real changes brought about by global warming? Now, according to new research, scientists have proposed even more disturbing changes: the temperature surge caused by global warming could drastically alter the Earth’s climate. Even if the Paris Agreement goal of limiting the global average temperature increase to within 2°C above pre-industrial levels is met, the Earth will still undergo many dangerous changes.

According to the Daily Mail, even if the long-term goal of the Paris Agreement is achieved, global sea levels will still rise by 6 meters or more due to rising temperatures, and tropical rainforests near the equator may transform into tropical savannas due to climate change. Large polar ice caps may also collapse, and important ecosystems may undergo major changes.

Recently, the research team examined three major warm periods in the past 3.5 million years, during which global temperatures were 0.5°C–2°C warmer than pre-industrial 19th-century levels. These three warm periods were 5,000–9,000 years ago, 129,000–116,000 years ago, and 3.3–3.0 million years ago. The first two warm periods were due to predictable changes in Earth’s orbit. However, the warming event around 3.3–3.0 million years ago was not. Scientists found that at that time, atmospheric CO₂ concentrations were about 350–450 ppm, similar to current levels on Earth.

10

Climate Change: Twelve Years Left to Save the Earth? We Only Have 18 Months!

(Copyright belongs to the author. For any form of reproduction, please contact the author.)

Do you remember that earlier time when people said: “We still have 12 years to save the Earth”?

Now it seems that people are gradually reaching a consensus—the next 18 months will be critical in addressing the global warming crisis and other environmental challenges.

Last year, the Intergovernmental Panel on Climate Change (IPCC) released a report stressing that to ensure global warming is kept within 1.5°C, net anthropogenic CO₂ emissions must fall by at least 45% by 2030 compared with 2010 levels.

But now, observers have realized that decisive political measures to make emission reductions possible must be implemented by the end of next year (not 2030).

As early as 2017, one of the world’s top climatologists persuasively explained to us—that 2020 was the definite deadline.

“Climate math is very clear: although the world cannot heal in the coming years, by 2020 the Earth may suffer fatal damage from human negligence and inaction,” said Hans Joachim Schellnhuber, founder and director of the Potsdam Institute for Climate Impact Research.

The recognition that the end of next year represents the last chance to tackle climate change is becoming clearer.

At a recent reception for Commonwealth foreign ministers, Prince Charles said: “I firmly believe that the next 18 months will decide whether we can keep climate change at survivable levels and restore the balance of nature upon which our survival depends.”

2020.1.1-20222.11.1

1

Release of the China’s Policies and Actions to Address Climate Change 2022 Annual Report — Steadfast and Powerful Steps Toward Green and Low-Carbon Development

The Ministry of Ecology and Environment recently released the China’s Policies and Actions to Address Climate Change 2022 Annual Report (hereinafter referred to as the “Report”). The Report covers five aspects: China’s new deployments to address climate change, proactive mitigation of climate change, active adaptation to climate change, improvement of policy systems and support safeguards, and active participation in global climate change governance. It comprehensively summarizes new deployments and policy actions in various fields since 2021, presents new progress and new achievements in China’s climate response work, and showcases China’s contributions to promoting global climate governance.

China Achieves a Win-Win of Economic Development and Pollution/Carbon Reduction

The Report points out that in recent years, under the guidance of Xi Jinping Thought on Ecological Civilization, China has firmly implemented the national strategy of actively addressing climate change, established the “1+N” policy system for carbon peaking and carbon neutrality, made a series of new deployments and arrangements, and adopted a more powerful package of policies and measures to vigorously, orderly, and effectively advance key tasks.

Industrial structure has been adjusted, with vigorous development of green and low-carbon industries. In recent years, industrial clusters such as new energy, new energy vehicles, and green environmental protection have been steadily promoted, supporting green, low-carbon, and high-quality development in industry, and building a green manufacturing system. By 2021, China had cultivated 430 specialized and innovative “little giant” enterprises in the energy conservation and environmental protection sector. The output value of the energy conservation and environmental protection industry exceeded 8 trillion yuan, with an annual growth rate of over 10%. Green product certification has covered nearly 90 categories, including building materials, express packaging, electrical and electronic products, and plastic products.

Energy structure has been optimized, with vigorous development of non-fossil energy and improvement in the clean utilization of fossil energy. By the end of 2021, China’s installed renewable energy power generation capacity reached 1.063 billion kilowatts, accounting for 44.8% of total installed capacity, and offshore wind power installations ranked first in the world.

2

Tsinghua University Professor Leads International Research Team to Propose a New Perspective on Global Climate Change Emission Reduction

China Education Daily – China Education News Network (Reporter: Dong Luanlong) – On October 25, the press conference for the Emerging Economies CO₂ Emissions Report 2022 was held in Beijing. Professor Guan Dabo of the Department of Earth System Science at Tsinghua University led an international team to release the Emerging Economies CO₂ Emissions Report 2022 and new research findings, proposing a new perspective on global climate change mitigation: small- and medium-sized emerging emitting economies (hereinafter referred to as “emerging emitting economies”) will be the main drivers of future global CO₂ emissions, while developed countries should make room for their future development in terms of carbon emissions.

At the press conference, Guan Dabo interpreted the Emerging Economies CO₂ Emissions Report 2022. He noted that in the new global climate governance framework, carbon emission accounting has become the foundation for accurately grasping emission trends and strengthening international cooperation on climate change. Previously, emerging economies generally faced issues such as inconsistent CO₂ emission accounting methods and incomplete scales. Therefore, the team used a “data crowdsourcing” approach, adopting internationally authoritative accounting methods to build a unified, transparent, and scientific accounting system, compiling CO₂ emission inventories for 50 emerging economies worldwide. The inventories provided in-depth descriptions and analyses of emissions from perspectives such as energy and sectoral sources and regional distribution, revealing the emission characteristics of emerging economies.

In addition, Guan Dabo shared the team’s latest research findings based on the emerging economies CO₂ emission inventory database. The research found that between 2010 and 2018, more than 50 emerging emitting economies had annual emission growth rates higher than the global average. Although the emissions of most economies accounted for less than 1% of global total emissions, their combined emissions were 1.6 times that of India. The industrialization-driven infrastructure construction in these countries will lead to continued growth in CO₂ emissions, which undoubtedly poses a severe challenge to the goal of limiting global warming to within 1.5°C by the end of this century.

3

The Lancet: Climate Change Causes Nearly 100 Million People to Go Hungry, Heat-Related Deaths Up 70% in the Past Five Years

On October 26, the globally authoritative medical journal The Lancet released its annual Countdown Report on health and climate change, pointing out that extreme weather caused by climate change is worsening public health conditions. Nearly 100 million people worldwide are suffering from hunger as a result, and the number of deaths among the most vulnerable populations due to high temperatures has increased by 68% over the past five years.

At the same time, the changing climate is affecting the spread of infectious diseases, putting many people at greater risk of emerging diseases and epidemics.

“Our health is being held hostage by fossil fuels,” said Marina Romanello, Executive Director of the Lancet Countdown and a health and climate researcher at University College London, at the press conference. “We see continued dependence on fossil fuels, which not only amplifies the health impacts of climate change but also exacerbates impacts alongside other simultaneous global crises—including the COVID-19 pandemic, inflation, the energy crisis, and the food crisis triggered by the Russia-Ukraine conflict.”

This annual report on health and climate change, released by the Lancet Countdown group, was jointly authored by nearly 100 experts from 51 institutions worldwide, and was released ahead of the upcoming United Nations Climate Change Conference (COP27). In addition to analyzing 43 indicators of health problems caused by climate change, this year’s report also added new content on hunger.

The report’s analysis noted that compared with 1981–2010, there were 98 million more reported cases of hunger worldwide in 2020, caused by the increasing frequency and intensity of extreme heat due to climate change.

“We are not saying that all food insecurity is caused by climate change. But we believe that in this complex web of causes, it is a very important factor—and it is only going to get worse.”

4

China’s Extreme Weather and Climate Events Increasing, Ministry of Ecology and Environment to Strengthen Capacity for Climate Change Adaptation

In recent years, under the global background of climate change, China has shown a trend of increasing frequency and intensity of extreme weather and climate events. On October 27, the Ministry of Ecology and Environment stated that it would deepen pilot projects for building climate-adaptive cities and strengthen capacity building for climate change adaptation.

At a press conference held on October 27, Li Gao, Director-General of the Department of Climate Change at the Ministry of Ecology and Environment, said that China’s climate conditions are complex, its ecological environment overall is fragile, and it is one of the countries most vulnerable to the adverse impacts of climate change. This summer, multiple regions in China experienced frequent extreme weather events, which severely affected people’s production and daily lives. Overall, extreme rainstorms, floods, high temperatures, droughts, and low temperatures have exhibited characteristics of strong extremity, obvious regional and seasonal phases, and frequent abnormalities. The scope of impact and resulting losses have further expanded, posing higher requirements for enhancing our ability to adapt to climate change.

This year, the National Climate Change Adaptation Strategy 2035 was issued, aligning with the goal of building a Beautiful China, and systematically planning China’s climate change adaptation work through 2035. To implement the National Climate Change Adaptation Strategy 2035, the Ministry of Ecology and Environment will guide local governments in preparing provincial-level climate change adaptation action plans and promote local adaptation work. The Guidelines for Preparing Provincial Climate Change Adaptation Action Plans have already been issued, and specialized training will be organized for localities to strengthen guidance on local adaptation work.

The Ministry will also deepen pilot projects for climate-adaptive city construction. Building on previous pilots, it will further explore mechanisms and models for constructing climate-adaptive cities, summarize and promote replicable experiences and practices, and continuously enhance urban climate resilience.

5

Top Ten Scientific Events in Climate Change in 2021

The IPCC released the AR6 WGI Assessment Report Climate Change 2021: The Physical Science Basis, pointing out that many observed changes are unprecedented. Climate change is bringing multiple different changes to different regions, all of which will intensify with further global warming. If active measures are taken now, many of the most catastrophic impacts of climate change can still be avoided.

The Glasgow Climate Change Conference reached the Glasgow Climate Pact, emphasizing the importance of science in policymaking for climate change response. Achieving the temperature control target requires rapid, deep, and sustained reductions in global CO₂ emissions, as well as substantial reductions in methane and other non-CO₂ greenhouse gases.

Climate scientists Syukuro Manabe and Klaus Hasselmann were awarded the 2021 Nobel Prize in Physics, marking the first time that the field of atmospheric and ocean sciences received the Physics Prize. This will further raise international attention to the issue of global warming.

The World Health Organization released the Special Report on Climate Change and Health, recommending that climate policies prioritizing the greatest health benefits should be considered. The report emphasized that unprecedented extreme weather events and other climate change-related events are causing increasing harm to human life and health, and that countries must make strong commitments to climate change action and achieve health-beneficial green recovery after the COVID-19 pandemic.

The Chinese government and the scientific community jointly advanced carbon peaking and carbon neutrality, with successive releases of the climate change White Paper, Blue Paper, and Green Paper.

Climate change caused frequent extreme events, with the probability of record-breaking heatwaves increasing, and tropical cyclones potentially expanding toward mid-latitude regions.

Climate tipping factors may trigger high risks. The Atlantic Meridional Overturning Circulation may be approaching a climatic tipping point. Crossing this threshold could result in a substantial and irreversible shift to a weakened state. The AMOC also affects global weather systems, and its potential collapse could have severe consequences—significantly cooling Europe and strongly affecting tropical monsoon systems.

A Decade of Climate Change in China: Entering a Pattern of Extreme Warming and Wetness

(Exclusive report by China Weather Network) This year, extreme heatwaves once again swept across the Northern Hemisphere, making the Earth seem hotter each year than the last. At the same time, record-breaking extreme precipitation events occurred frequently. Unprecedented extreme weather and climate events are appearing with increasing frequency on this planet. We are living through the most dramatic decade of climate change since the Industrial Revolution. In China, extreme weather and climate events have shown the characteristics of frequent occurrence, widespread impact, repeated outbreaks, and concurrent events. The feared “hotter, drier, and wetter future” may indeed be gradually becoming reality.

Not long ago, we experienced the world’s third hottest June and second hottest July on record. In China, an extraordinary heatwave lasting 79 consecutive days struck—this was also the most intense heatwave event in terms of comprehensive strength since 1961.

It is not only this year. Looking back over the past decade, we have witnessed more and more “extreme heatwave events,” “hottest summers,” and “hottest years in history.” The feeling that “each year is hotter than the last” is truly not an illusion.

According to data from the National Climate Center, since 1951, the annual average temperature in China has shown a significant upward trend, with a warming rate of 0.26°C per decade—higher than the global average of 0.15°C per decade during the same period—making China a sensitive area to global climate change.

The period from 2012 to 2021 was the warmest decade in the past 70 years. Among the ten hottest years on record nationwide, seven occurred within these ten years, with 2021 being the hottest year since meteorological records began.

The Qinghai–Tibet region has shown the fastest rate of warming, with an increase of 0.37°C per decade. Northern China, Northeast China, and Northwest China followed, with rates of 0.33°C, 0.31°C, and 0.30°C per decade respectively. Overall, the Qinghai–Tibet Plateau and mid- to high-latitude regions are the areas where warming has been most pronounced in China.

7

Ministry of Foreign Affairs: Climate Change Requires Joint Efforts of the International Community

A reporter asked: Recently, UN Secretary-General António Guterres called on the international community to provide assistance to African countries in coping with climate change, saying that concrete actions must be taken to address loss and damage caused by climate change, which concerns the survival of many countries, especially in Africa. What is China’s response?

Mao Ning responded: We have noted the relevant remarks by Secretary-General Guterres. In supporting Africa’s response to climate change, China has always been consistent and never absent. The Eighth Ministerial Conference of the Forum on China–Africa Cooperation adopted the China–Africa Declaration on Climate Change Cooperation, proposing the establishment of a new-era strategic partnership on climate change between China and Africa, and incorporating green development projects into the first three-year plan of the China–Africa Cooperation Vision 2035. Last month, China hosted the Ministerial Meeting of the Group of Friends of the Global Development Initiative, and proposed advancing the Global Clean Energy Partnership. The first batch of projects includes cooperation on climate change and green development in 19 African countries.

Mao Ning further introduced that China not only sincerely helps African countries cope with climate change, but also remains faithful to its commitments and takes pragmatic actions in addressing global climate change. We are actively advancing low-carbon development and green transformation domestically, committed to achieving the “dual carbon” goals. We constructively participate in the multilateral climate change process, making historic contributions to the conclusion and implementation of the Paris Agreement. China’s efforts have injected strong momentum into global climate action.

Finally, Mao Ning emphasized: Climate change is a common challenge for all humankind, concerning the future and destiny of humanity. It requires joint efforts of the international community. Developed countries bear undeniable historical responsibility for climate change, and should genuinely follow the principle of common but differentiated responsibilities, take the lead in undertaking significant emission reduction obligations, and faithfully deliver on their commitments to provide financial, technological, and capacity-building support to developing countries.

8

“We are now not only in the year 2022 AD, but also in the third year of the climate crisis era. This is a brand-new age in human history, and also in Earth’s history.”

These words were spoken by Wei Ke, Associate Researcher at the Institute of Atmospheric Physics of the Chinese Academy of Sciences. In June this year, during a public lecture, the 42-year-old meteorologist gave the audience a playful look and slowly said: “Why the third year? Keep listening and you’ll find out.”

Will 2022 be the hottest year on record? At least for many places, the past June and July already were. In June this year, land areas across the globe recorded the highest temperatures since the late 1850s, when systematic meteorological records began. Record-breaking heat, drought, wildfires, rainstorms, and floods swept the Northern Hemisphere, particularly across the European continent, the UK, and parts of the United States.

In this unbearably hot summer, more and more records were continually broken. However, it must be emphasized that the increasingly frequent and intense climate crises did not begin this year, nor are they unique to any one country or region. They are a continuing global phenomenon.

What is happening to our world? According to a report released in 2020 by the United Nations Office for Disaster Risk Reduction, compared with the previous twenty years, the first twenty years of the 21st century saw a substantial increase in the frequency of various disasters. Heat events increased by 232%, rainstorms by 134%, and all types of storms by 97%. China was the country with the most reported climate-related disasters in the report.

Behind these disasters lies an issue we must face: global temperatures are continuing to accelerate upwards. For every ordinary person, climate change is no longer distant or irrelevant. Our generation is now experiencing, and will continue to experience, more climate crises.

9

Global Warming Leading to Frequent Extreme Weather Events

Since June this year, heatwaves have swept across multiple countries in the Northern Hemisphere. Experts attribute this summer’s “roasting” high-temperature pattern to climate change. Climate change has triggered unprecedented extreme weather events worldwide, and future extreme heat is expected to occur more frequently and intensely.

The Intergovernmental Panel on Climate Change (IPCC) Sixth Assessment Report states that global warming in the past 50 years has occurred at a rate unprecedented in the last 2000 years, with instability in the climate system intensifying. According to China’s National Climate Center, global warming is the climatic background for the frequent occurrence of heatwave events in the Northern Hemisphere.

The World Meteorological Organization (WMO) believes that under the influence of climate change, future extreme heat events will appear more frequently and intensely. If greenhouse gas emissions continue to rise, the extent of global warming will be greater, and what we are experiencing now is merely “a foreshadowing of the future.” UK Met Office scientist Nikos Christidis emphasized that the risks brought about by climate change are rapidly increasing, with extreme weather events “becoming more and more common, and by the end of the 21st century, they will no longer be extreme events.”

Heatwaves May Become the “Norm” for European Summers

Faced with the heatwave sweeping across Europe, WMO Secretary-General Petteri Taalas stated on the 19th local time that high temperatures may become the “norm” for European summers, and that the negative trends triggered by climate change will continue at least until 2060. Bob Ward, Policy and Communications Director of the Grantham Research Institute on Climate Change and the Environment at the London School of Economics, also said that heatwaves will become more intense and frequent for at least the next 30 years.

Since the late 19th century, due to the emission of carbon dioxide and other greenhouse gases, global average temperatures have been rising. This cumulative effect will continue until we effectively achieve net-zero greenhouse gas emissions. This may occur as early as 2050, but it does mean that in the next 30 years, average temperatures will still rise, and extreme weather will continue to increase.

10

New Study: Greenland Ice Melt “Inevitable,” Global Sea Level to Rise at Least 25 cm

The journal Nature Climate Change published a study on Monday pointing out that with climate change and global warming, Greenland’s ice sheet will inevitably undergo large-scale melting in the future, leading to a global sea level rise of at least 25 cm.

“Zombie Ice” Destined to Melt

Nature Climate Change reported on the 29th that the total melt of Greenland’s ice sheet in the future will reach 110 trillion tons, resulting in at least a 10-inch (about 25 cm) rise in sea level. This is equivalent to the combined contribution of Greenland and Antarctic glacier melt plus seawater thermal expansion to global sea level rise over the entire last century.

The Geological Survey of Denmark and Greenland conducted the study. Researchers observed changes in the volume of Greenland’s ice sheet and surrounding glaciers, finding that runoff from ice melt is the main driver of global sea level rise.

According to scientists’ calculations, regardless of how the climate changes this century, about 3.3% of Greenland’s ice sheet—equivalent to 110 trillion tons of ice—will “inevitably melt.” If all this ice melted into water and were poured into the United States, it would cover it to a depth of 11 meters.

“This study provides the minimum value for Greenland ice sheet melt. Given the almost certain trend of global warming, sea levels will only continue to rise,” said Jason Box, lead author of the study and an expert at the Geological Survey of Denmark and Greenland.

Another author of the study, glaciologist William Colgan, explained that glaciers have their own balance system. In a perfect balance, snowfall on Greenland’s mountains would replenish the melting ice surface. But in recent decades, this balance has been disrupted: less replenishment, more melting. Under such conditions, some ice becomes “destined to melt”—scientists call this “zombie ice.”

**Climate change search volume peak news**

2015.11.30-2015.12.6

1

The Truth About Global Warming: Carbon Dioxide Is Like a Quilt, Methane Is Like a Blanket but Even Stronger

At the ongoing United Nations Climate Change Conference in Paris, discussions are underway on how humanity can work together to tackle global warming in the future.

According to some scientists, the Earth is rapidly sliding into the “danger zone” of global warming, a global challenge that involves multiple interlinked factors, making it extremely complex to address. By taking a time-based perspective, we can examine nine aspects of global warming to understand nine key truths.

Carbon Dioxide: The Thicker the “Quilt,” the Hotter the Earth Becomes

Greenhouse gases in the atmosphere wrap around the Earth like a quilt, trapping part of the Sun’s heat and keeping the planet warm. Since the Industrial Revolution, human activities have increased the concentration of greenhouse gases in the atmosphere, effectively thickening this quilt. This has caused the heat stored at the Earth’s surface to exceed what is necessary, leading to global warming.

According to data from the United Nations Environment Programme and the World Resources Institute, global greenhouse gas emissions caused by human activity have been on an upward trend, reaching nearly 50 billion tons of carbon dioxide equivalent in 2012, and 52.7 billion tons in 2014.

Greenhouse gases include carbon dioxide, methane, nitrous oxide, and fluorinated gases. Among them, carbon dioxide emissions have increased most significantly, with the largest sources being the combustion of fossil fuels such as coal, oil, and natural gas. Other human activities, such as agriculture, cement production, livestock raising, and deforestation, also contribute to higher carbon dioxide emissions.

Methane: The “Blanket” That Traps Heat Even More Than the “Quilt”

If carbon dioxide is a “quilt,” then methane, another greenhouse gas, is like a “blanket,” with an even stronger heat-trapping effect.

In the natural environment, methane is produced from livestock digestion, wetlands, and thawing permafrost, but it can also result from human activities such as landfills and rice cultivation. Industrial activity releases large amounts of methane alongside carbon dioxide.

According to the U.S. National Oceanic and Atmospheric Administration (NOAA), in 2014 the concentration of methane in the atmosphere was more than 2.5 times its pre-industrial level.

Compared with carbon dioxide, methane makes up a smaller share of the atmosphere and has a shorter atmospheric lifetime. However, in terms of its efficiency at trapping heat, methane is far more “effective” than carbon dioxide, making it a more potent greenhouse gas.

2

What Commitments Has China Made in Response to Global Climate Change?

On November 30 (local time), President Xi Jinping attended the opening ceremony of the Paris Climate Conference and delivered an important speech titled “Working Together to Build a Cooperative, Win–Win, Fair and Reasonable Climate Change Governance Mechanism.”

According to People’s Daily Online (December 1), this year’s global climate conference was held in Paris from November 30 to December 11, with representatives from 195 countries and the European Union in attendance to discuss the shared challenges facing human society today.

In the face of climate change, China has always responded proactively. This is not only an inherent requirement for China to achieve sustainable development but also a responsibility it bears as the “largest developing country” to actively participate in global governance and contribute to building a community with a shared future for humankind.

From the China–U.S. Joint Statement on Climate Change at the end of 2014, to China’s submission this year of its Intended Nationally Determined Contribution (INDC) to the United Nations, and successive joint statements with India, Brazil, the EU, the U.S., France, and other countries and regions on climate change, China has made a series of solemn commitments. These have sent out strong political signals to the world, demonstrating its determination to follow a green, low-carbon, and sustainable development path—earning widespread international recognition.

China Among the First Parties to the UNFCCC

In 1992, at the United Nations Conference on Environment and Development in Rio de Janeiro, Brazil, the United Nations Framework Convention on Climate Change (UNFCCC) was officially opened for signature.

This was the world’s first international treaty drafted to control greenhouse gas emissions and address global warming. Representatives from 153 countries and the European Community, including then Chinese Premier Li Peng, signed the UNFCCC.

China was one of the first ten Parties to the Convention. The Convention explicitly stipulated that developed and developing countries should assume “common but differentiated responsibilities” in protecting the global climate. The Convention officially entered into force on March 21, 1994.

3

Addressing Climate Change by Building a Green and Low-Carbon Corridor

Discussing the Belt and Road Initiative (BRI) from the perspective of low-carbon sustainable development and climate change response offers a new lens. Along the Belt and Road, regions may adapt to climate change and find new development opportunities through strengthened infrastructure construction.

Since General Secretary Xi Jinping proposed the strategic vision of “jointly building the Silk Road Economic Belt” in September 2013, the Belt and Road has become an important strategic pivot in China’s new pattern of all-round opening-up. Climate change is a common risk and challenge facing all of humanity. Addressing climate change is both a crucial opportunity for global green, low-carbon, and sustainable development, and a strategic factor in shaping the development pathways of BRI countries. Therefore, incorporating climate change response into the Belt and Road’s strategic planning holds long-term and profound significance.

Severe Resource and Environmental Pressures

Easing resource and environmental pressures and following a sustainable development path is a huge challenge for economic growth in Belt and Road regions.

Over the past 20 years, the economies of Belt and Road countries and regions have grown rapidly, with annual GDP growth rates about twice the global average. However, per capita GDP remains less than half the global average. The shares of agriculture and industry in GDP are significantly higher than the global average, while the share of services is markedly lower. Energy consumption, raw wood use, material consumption, and carbon dioxide emissions per unit of GDP are all more than 50% higher than the world average. Consumption of steel, cement, non-ferrous metals, water, and ozone-depleting substances per unit of GDP is more than twice the global average.

Overall, these regions are still in a stage where economic growth is strongly tied to resource consumption and pollutant emissions, with both continuing to rise rapidly. This poses a severe challenge to the sustainable development of their economies and societies.

4

Foreign Media: China Shows Great-Nation Responsibility and Determination in Tackling Climate Change

According to China News Service (December 1), the climate conference, which carries global expectations and the future of humankind, was held in Paris on November 30. About 157 heads of state and government, including Chinese President Xi Jinping, attended the opening ceremony. Foreign media noted that it was unprecedented for China’s top leader to personally attend and speak, highlighting China’s seriousness about the conference and its expectations for meaningful outcomes. This reflects the responsibility and determination of a major country in tackling climate change.

Hong Kong’s Ta Kung Pao commented on December 1 that greenhouse gas concentrations in the atmosphere reached new highs in 2015. The world is looking to the Paris conference to deliver strong decisions and set a milestone in global climate action.

This conference was regarded as the most favorable negotiating opportunity since the signing of the Kyoto Protocol in 1997. Major powers had deepened their understanding of the consequences of climate change and showed stronger willingness to cooperate. Many issues had already seen consensus reached through bilateral agreements. The participation of over 100 leaders provided momentum, making the Paris conference closer to success than ever before.

Analysts observed that at this climate conference, China, as the world’s second-largest economy and largest developing country, was playing a highly significant role. China is one of the largest emitters of carbon, but also one of the most active in energy conservation and emission reduction. Its proposals and ideas are increasingly recognized, and its actions are winning global approval. In addressing climate change, China is becoming a leading example with demonstrative significance.

President Xi Jinping has repeatedly stressed that tackling global climate change is a “tough battle” that concerns the interests and well-being of all humanity, and that only by joining hands can countries win this fight. China has signed multiple bilateral statements with the U.S., Europe, India, and others, resolving many differences that might otherwise hinder negotiations. Among them, the China–U.S. Joint Statement demonstrated a spirit of cooperation between major powers, with both sides making significant emission reduction commitments.

5

Tackling Climate Change, Demonstrating China’s Responsibility

In the depth of winter, over 100 heads of state and representatives from nearly 200 countries gathered at the United Nations Paris Climate Conference to jointly discuss the post-2020 international mechanism for tackling climate change, and to explore sustainable development pathways and governance models for humanity. On November 30, Chinese President Xi Jinping delivered an important speech at the meeting, articulating China’s views and positions on global climate governance.

How important is this Paris Climate Conference? UN Secretary-General Ban Ki-moon’s appeal makes it clear: “Now is the time for us to use common sense, to make compromises, and to reach consensus. It is also the time to look beyond national interests and put the common good first.” One could say that humanity’s efforts to tackle climate change have reached a critical juncture: with a concerted push, we can overcome the “collective action dilemma” in one go, but with even a slight relaxation, we risk sliding back into a “Copenhagen-style blizzard.”

“A successful international agreement must not only resolve present conflicts but also guide the future,” Xi Jinping said in his speech, outlining four guiding principles for the forthcoming Paris Agreement: “It must help achieve the objectives of the Convention and guide green development; it must unite global strength and encourage broad participation; it must increase investment and strengthen action guarantees; it must take into account different national circumstances and pursue pragmatic and effective solutions.”

At the same time, China’s proposals uphold the principle of “common but differentiated responsibilities.” They not only spoke up strongly on behalf of developing countries but also called on developed countries to take action with reasoned arguments. As the Financial Times noted: “At a time when the international climate agenda lacks advocates willing to take political risks, China is demonstrating leadership. The Chinese government’s initiatives have injected new momentum into the Paris Climate Conference.”

6

2015: The Hottest Year on Record

Yesterday in Geneva, the World Meteorological Organization (WMO) released its provisional statement on the global climate in 2015 and analyzed climate conditions from 2011 to 2015. The statement indicated that this year may be the warmest year on record, and that 2011–2015 will also be the warmest five-year period in history.

The WMO noted that in 2015 the global average surface temperature may reach the highest in history, about 1°C higher than the pre-industrial level, marking a critical threshold. From 1961 to 1990, the global average surface temperature was 14.0°C, while in 2015, the global average surface temperature from January to August was approximately 0.73°C higher than that baseline, and about 1°C higher than during the 1880–1899 industrial revolution period. The rise in global temperature was mainly due to the combined effects of a strong El Niño and global warming.

According to WMO data analysis, influenced by climate change, 2011–2015 was the warmest five-year period on record, approximately 0.57°C higher than the 1961–1990 reference period average. During this time, extreme weather events worldwide—especially heatwaves—occurred frequently.

“All these record-breaking events will ensure that 2015 is remembered for its global climate conditions. The concentration of greenhouse gases in the atmosphere broke historical records; in the Northern Hemisphere spring, global average carbon dioxide concentration surpassed 400 ppm (parts per million) for the first time. 2015 may well be the warmest year in history, with ocean surface temperatures also reaching the highest values ever recorded. It is highly likely that 2015 will exceed the critical 1°C threshold,” said WMO Secretary-General Michel Jarraud.

7

Global Warming: Nine Truths the People of Earth Must Know

At the United Nations Climate Change Conference currently being held in Paris, discussions are focused on how humanity can work together to address global warming in the future.

According to some scientists, the Earth is rapidly sliding into the “danger zone” of global warming, a global challenge involving multiple mutually reinforcing factors and a vast array of complexities.

Taking a time-based approach, Xinhua International Client lays out nine key truths about global warming.

Greenhouse gases in the atmosphere wrap around the Earth like a quilt, trapping part of the Sun’s heat and keeping the planet warm. Since the Industrial Revolution, human activities have increased the concentration of greenhouse gases in the atmosphere, thickening this quilt and causing the Earth to warm as excess heat is retained at the surface.

According to data from the United Nations Environment Programme and the World Resources Institute, global greenhouse gas emissions from human activities have been on the rise, reaching nearly 50 billion tons of carbon dioxide equivalent in 2012 and 52.7 billion tons in 2014.

Greenhouse gases include carbon dioxide, methane, nitrous oxide, and fluorinated gases. Among them, carbon dioxide emissions have increased most significantly, mainly from burning fossil fuels such as coal, oil, and natural gas. Other human activities, including agriculture, cement production, livestock raising, and deforestation, also contribute to higher carbon dioxide emissions.

If carbon dioxide is the “quilt,” then methane, another greenhouse gas, is the “blanket,” with an even stronger heat-trapping effect.

In the natural environment, methane is produced from livestock digestion, wetlands, and thawing permafrost, but it can also come from human activities such as landfills and rice cultivation. Industrial activity releases large amounts of methane alongside carbon dioxide.

8

The Climate Change Risks Facing South China: Real, Urgent, and Closely Connected to Us

As the British Consul-General in Guangzhou, my work covers a wide range of areas; however, the single most important issue that will affect our way of life and sustained prosperity is undoubtedly climate change. Without question, climate change is the most pressing real challenge currently facing both the UK and South China.

For the Pearl River Delta in particular, these impacts may seem distant, but we must take concrete, targeted action now.

This week, world leaders gathered in Paris for the largest climate change forum in six years. China has already set out ambitious emission reduction plans, aiming for greenhouse gas emissions to peak no later than 2030. The governments of South China’s provinces (Guangdong, Guangxi, Hunan, Jiangxi, Fujian, and Hainan) are also making their own contributions toward China’s low-carbon transition during the 13th Five-Year Plan period.

To achieve the “2°C scenario”—that is, to keep the global average temperature increase to no more than 2°C above the long-term historical average—we must unite and reach consensus.

At the same time, we must recognize that the risks posed by climate change are still not fully understood. For example, even under the “2°C scenario,” by the year 2100 large parts of the Pearl River Delta could still be completely submerged.

The current consensus is that for every 1°C rise in global average temperature, sea levels will rise by about 2.3 meters. This means that even if we manage to achieve the “2°C scenario,” the Pearl River Delta would still face the threat of a 4.6-meter sea-level rise (and if we do not strengthen climate action, even the “2°C scenario” may be out of reach). For the Guangzhou region, for example, the dark blue areas on the map would be engulfed by the ocean if sea levels rise by 5 meters.

9

On Global Climate Change: Eight Charts You Need to See

Is your country vulnerable to the impacts of climate change? Which countries are making major efforts to cut carbon dioxide emissions? Which countries are best prepared to adapt to climate change?

As the 2015 UN Climate Conference in Paris gets underway, we considered what readers most want to know. Using the most searched keywords on Google, we identified the key topics people are curious about, to help readers understand the debates unfolding at the summit.

Looking at the keyword “global climate change,” we found that people are most interested in the basics, such as “What is climate?” Many are also concerned about the causes and impacts of climate change, as well as how to adapt to it.

So let’s take a look:

Causes of Climate Change

Climate change is caused by human-driven increases in greenhouse gas emissions. Today, atmospheric carbon dioxide levels have reached their highest point in history.

Greenhouse gases include carbon dioxide (CO₂), methane (CH₄), nitrous oxide (N₂O), hydrofluorocarbons (HFCs), and perfluorocarbons (PFCs). Carbon dioxide is the largest contributor. Although methane and nitrous oxide account for smaller proportions of emissions, they still have significant climate impacts: methane is 25 times more powerful than carbon dioxide, while nitrous oxide is about 300 times more damaging.

Overall, greenhouse gas emissions from burning major fossil fuels (coal, oil, natural gas) account for about 65% of total emissions. The remaining 35% comes mainly from methane released by livestock and waste, as well as emissions from deforestation and land clearing.

10

Paris COP21: “Constituting” Climate Action

From November 30 to December 11, the 21st Conference of the Parties to the United Nations Framework Convention on Climate Change (COP21) was held here. More than 150 heads of state, 196 Parties, and 43,000 participants all came “for the sole purpose of protecting the Earth,” to ensure that the world delivers a new agreement to tackle climate change. This agreement will send a critical signal to the world about the transition toward a low-carbon economy.

The political momentum for reaching this climate agreement has built to its highest point. “Such political momentum may not come again,” UN Secretary-General Ban Ki-moon said on November 30 at the Global Leaders’ Forum.

At 1:30 p.m. local time in Paris, Chinese President Xi Jinping addressed the summit on behalf of China’s 1.4 billion people, sharing China’s expectations for the Paris conference and its views on global governance.

“French writer Victor Hugo once said, ‘The greatest determination produces the highest wisdom.’ I believe that as long as all parties demonstrate sincerity, firm confidence, and unity, the Paris conference will surely achieve a satisfactory outcome,” Xi Jinping said.

“China has provided a tremendous political push to support the success of this conference,” said Xie Zhenhua, China’s Special Representative on Climate Change.

According to the schedule, after the opening ceremony, national leaders would authorize negotiators to begin substantive talks on the Paris Agreement, which would establish an international post-2020 climate mechanism applicable to all Parties.

At present, 196 Parties have submitted their Intended Nationally Determined Contributions (INDCs), but these are still insufficient to keep humanity within the 2°C “safety threshold.”

Most of the world’s scientists believe that failing to do so will bring irreversible impacts to human civilization, including coastal cities being submerged by rising sea levels.

“2°C is the political consensus, and this will become the Paris-oriented target,” said Su Wei, China’s chief negotiator and deputy head of the delegation, in an interview with 21st Century Business Herald regarding the long-term climate goal.

2017.12.4-2017.12.10

1

Study Says Climate Change May Increase Risk of Civil Wars in Certain Regions

MEXICO CITY, December 5 (Xinhua) — A research report released on the 5th by Mexico’s Ibero-American University shows that climate change will impact societies in many ways, such as potentially increasing the risk of civil wars in some regions.

The report, titled Climate Change, Natural Disasters and Their Macroeconomic Effects, points out that in the Sahara region of Africa, climate change may cause reduced harvests and food shortages in some countries, leading to disputes over arable land. As a result, the likelihood of civil war breaking out in those countries the following year could rise to 50%.

The report also notes that in recent decades, the number of natural disasters such as floods and tornadoes has increased at an alarming rate due to the effects of climate change. Regarding the economic impacts of natural disasters, researchers at Ibero-American University pointed out that disasters reduce production activities, affect trade balances, worsen fiscal conditions, and exacerbate poverty levels.

In addition, natural disasters have a particularly large impact on vulnerable groups, which further aggravates social inequality. Both wealthy and poor countries may suffer from natural disasters, but less-developed countries find it harder to recover from them.

The report calls on the international community to take measures in science and technology, public policy, and financial investment to address the various challenges brought by climate change.

2

Climate Change May Intensify Volcanic Eruptions in Iceland

BEIJING, December 9 (Xinhua) — A new British study has found that during periods when Iceland’s glaciers covered larger areas, local volcanic activity was relatively low; but now, with global warming causing glaciers to melt, volcanic activity in Iceland may intensify.

Researchers from the University of Leeds reported in the latest issue of the U.S. journal Geology that they studied Icelandic volcanic ash preserved in peat and lake sediments. They found that between 5,500 and 4,500 years ago, volcanic activity in Iceland was significantly reduced. Before that, global temperatures had dropped sharply, which increased the extent of Iceland’s glaciers.

The researchers speculate that the reduced volcanic activity at the time may have been related to the extensive glacier coverage. Further analysis showed that from the time when temperatures decreased, causing glaciers to expand, to the reduction in volcanic activity, there was a lag of several hundred years.

Since the end of the last “Little Ice Age” around 1850, Iceland’s glaciers have been melting under the combined influence of natural and human-driven global warming. Thus, the researchers conclude, volcanic activity in Iceland will also become more active. “Although the influence of human activities on global warming makes it difficult to predict the length of the time lag between warming and increased volcanic activity, historical trends tell us that Iceland will face more volcanic eruptions in the future.”

Iceland’s volcanic activity results from multiple factors, including continental rift activity, pressure from underground gases and magma, and the pressure glaciers exert on the volcanoes’ surface. The researchers analyzed that when glaciers decrease, the pressure they exert on the Earth’s surface lessens, which may cause mantle rock to melt, affect magma flow, and thereby trigger volcanic eruptions.

3

China-Canada Joint Statement on Climate Change and Clean Growth

China and Canada confirm that climate action, including a firm transition toward low-carbon, climate-resilient, and sustainable development, is of critical importance. As a global challenge, climate change and the need to transition to a clean-growth economy require governments, enterprises, and other actors to respond decisively, collaboratively, and cooperatively in the context of sustainable development and poverty eradication, thereby injecting momentum.

Premier Li Keqiang and Prime Minister Justin Trudeau agreed that strengthening China-Canada cooperation is of great significance for global efforts to mitigate climate change and adapt to its impacts. Both sides further emphasized advancing environmental protection and economic growth in a coordinated manner. They pledged to take pragmatic action and demonstrate their determination to encourage the transition toward competitive, low-carbon, and climate-resilient economies and societies, and to promote clean growth.

Cooperation in Multilateral Processes

3. China and Canada welcomed the entry into force of the Paris Agreement under the United Nations Framework Convention on Climate Change (UNFCCC), which aims to strengthen the Convention, including its goals, and to bolster the global response to the threat of climate change. Both sides reaffirmed that the Paris Agreement is irreversible and cannot be renegotiated. They again confirmed their strong commitment to the Agreement, reflecting fairness and the principle of common but differentiated responsibilities and respective capabilities, while taking into account different national circumstances. They committed to rapid, comprehensive, and effective implementation of the Agreement and to advancing their respective nationally determined contributions (NDCs). Both sides called on all Parties to uphold and advance the Agreement, implement their NDCs, and strengthen their efforts over time in accordance with relevant provisions.

4

The Enormous Impacts of Climate Change: Human Reproduction May Be Threatened

Everyone knows that the Earth’s climate is undergoing enormous changes: sea levels are continuously rising, air quality indices are worsening, some places are experiencing continuous floods, while others face long-term droughts and water shortages. These are some of the impacts of climate change on our planet and human life.

Beyond these, climate change may also bring about impacts that people are less familiar with. Some researchers have suggested that the enormous impacts of climate change may threaten human reproduction for future generations. What exactly does this mean?

According to global statistics, in many developed countries, birth rates have been continuously declining. In some developed cities, negative population growth has even appeared. The causes of these issues are closely related to environmental factors, though there are also more direct reasons such as the use of contraceptives and heavy economic pressures in daily life.

However, scientists have stated that the decline in birth rates associated with climate change is scientifically supported, even if many ordinary people remain skeptical. For example, in 2015, a U.S. study specifically investigated this issue and found that if the average daily temperature remains at 26.7°C for about 10 months, the average birth rate will continuously decline, dropping by about 0.5%. This may sound abstract, and many people may not have a clear idea of how serious this is.

5

Seven Surprising Impacts of Climate Change: Each More Unexpected Than the Last

Sea level rise, deteriorating air quality, and longer and more frequent droughts are usually considered the main ways in which climate change affects the human biosphere, seriously disrupting normal human life.

In a research article titled Climate Change Will Lead to War, John Wendell, a columnist for Nautilus Magazine, pointed out that in the vast Fergana Valley of Central Asia, complex borderlands, ethnic tensions, struggles over water resources, dwindling resources, and rising temperatures have combined to make this region a place where global warming and human conflict intersect — an area characterized by a unique convergence of geography, climate change, and political strife.

However, the impacts of global climate change go far beyond this. Many surprising changes are also occurring. Because of the interdependence between Earth’s ecosystems and living organisms, once the climate changes, all animals, including humans, will be forced to adapt to their natural environment in unexpected ways. These involve animal physiology, species boundaries, human behavior, and social organization. Below are seven surprising impacts of climate change:

Scientists point out that in recent years we have seen more and more sharks for two main reasons: rising sea temperatures are pushing sharks to migrate northward, and longer summers are encouraging more people to swim and recreate at beaches. Either of these factors alone could increase the number of shark attacks on humans, but when combined, their effect is even stronger. Statistics show that 2015 was the year with the highest number of unprovoked shark attacks on humans worldwide, with a total of 98 incidents.

6

Global Climate Change Is Absolutely Not What You Think! It’s Not Only China That Has Smog

If you still think that only China has smog, and always assume that many places in the world can serve as refuges, then you may need to wake up.

The following set of photos truly reflects the extent of global environmental fragmentation over the past year.

Researchers from NASA and the University of California recently detected the fastest rate of glacier melting in history in West Antarctica, which will directly lead to sea level rise and is very likely to be an irreversible process.

The basin of the Pilcomayo River, flowing through Argentina and Paraguay, is facing the most severe drought in nearly 20 years. Crocodiles are trapped in muddy riverbeds, their fate uncertain, and crows perched on withered branches are becoming increasingly common sights.

In the first half of the year, when scientists conducted field research on the Great Barrier Reef, they found that about 93% of the corals had experienced bleaching due to rising sea temperatures.

Measurements by the Austrian Institute for Mountain Research showed that the snowline of the Alps is rising at a rate of 1.5 meters per year.

Bolivia experienced the most severe drought in 25 years, entering a state of emergency. Glacier water, the main source of urban water supply, has dropped sharply, and the Ajuan Khota Dam has already dried up.

Sea ice is crucial to the survival of polar bears, but studies show that the formation of winter ice in the Arctic has already slowed. In Hudson Bay, the polar bear population has declined by 20% over the past 30 years.

In December, Beijing issued a “red alert for smog,” with traffic restrictions, school closures, and factory shutdowns. On that day, the air pollution index exceeded 260, more than five times the level safe for human breathing.

After Ethiopia experienced the most severe drought in 50 years, excessive rainfall and flooding once again damaged its already fragile farmland.

7

Nature-Based Solutions to Climate Change: Protecting and Restoring Land Is Key

When it comes to addressing climate change, land is key. Today, agriculture, forestry, and other land uses generate about one-quarter of global greenhouse gas emissions. Implementing sustainable land management strategies can contribute more than one-third of the short-term emission reductions needed to achieve the targets set by the Paris Climate Agreement.

For a long time, we have sought to establish a balance of interaction between people and nature, but only recently have we realized the crucial role of land-use management in solving climate change problems. With the development of remote sensing, artificial intelligence, and biochemical modeling, we are now better able to predict outcomes and design strategies to manage and minimize negative consequences.

Some of the most promising ways to curb climate change are called “natural climate solutions”: protecting, restoring, and enhancing land management to boost carbon sequestration capacity or avoid greenhouse gas emissions from land use worldwide. A new study conducted by The Nature Conservancy and 15 other leading institutions has calculated the total potential of these solutions in detail.

One of the most important natural climate solutions is protecting frontier forests — primary forests that act as natural carbon sinks. Intact tropical and boreal forests, as well as savannas and coastal ecosystems, have stored massive amounts of carbon over centuries. If these areas are destroyed, the carbon will be released. Protecting frontier habitats also helps manage water flows, reduce flood risks, and maintain biodiversity.

Afforestation is another important natural solution. Globally, it is estimated that two billion hectares of land have been deforested or degraded. Trees are the best carbon-capturing and storage mechanism the world possesses, so reversing this trend will significantly lower global carbon levels. We estimate that simply planting more trees could enable the world to capture three billion tons of carbon dioxide annually — equivalent to the emissions of 600 million cars.

8

El Niño Greatly Increases Global Forest Fires and Carbon Emissions

A paper published online on the 3rd in the British journal Nature Climate Change reports that El Niño has significantly increased the number of fires in pan-tropical forests and the resulting carbon emissions. The study found that El Niño reduces rainfall and water storage in pan-tropical forests, thereby fueling the occurrence and spread of fires. These fires follow the seasonal patterns of tropical continental regions, which makes them predictable and may help scientists forecast fire events.

The El Niño–Southern Oscillation (ENSO) is a periodic variation of the ocean–atmosphere system. In the tropical eastern Pacific, sea surface temperature changes occur — the warm phase (El Niño) and the cold phase (La Niña) — accompanied by changes in sea-level pressure over the equatorial western Pacific. La Niña is the exact opposite of El Niño, being a phenomenon in which sea surface temperatures remain abnormally cold. ENSO has now been proven to have a huge impact on annual climate variation.

A research team from the University of California analyzed satellite data from 1997 to 2016 (covering six El Niño and six La Niña events) to identify climate conditions associated with burned areas and combustion emissions. They found that compared with La Niña events, El Niño-induced reductions in rainfall and water storage led to an average 133% increase in combustion emissions from pan-tropical forests.

The study showed that fire occurrences in equatorial Asia peak from August to October, then shift to Southeast Asia and northern South America from January to April of the following year, move to Central America from March to May, and finally to southern Amazonia from July to October.

This fire trajectory reveals the Earth system’s lagged response to ENSO. Improving understanding of this could help build fire risk forecasts and explain the accelerated growth rate of atmospheric CO₂ concentrations during El Niño events.

9

China’s Climate and Ecological Environment Improves in the Past Five Years: Over 80% of Areas Show Increased Vegetation Coverage, Significant Improvement in Desertification

The latest research by the National Climate Center shows that in the past five years (2012–2016), China’s climate pattern has been characterized by warm and wet conditions, and overall air quality has improved. Vegetation coverage has increased in more than 80% of the country, and desertification has been effectively controlled.

Against the background of climate warming, from 2012 to 2016, China’s average temperature was 10.1°C, which is 1.2°C higher than the average from 1961 to 1990. Average precipitation was 7.1% higher. Meteorological disasters overall showed an increasing trend, with the number of heavy rain days increasing by 22.5%; drought days in North China and Northeast China increased by 20%–30%; and the average maximum wind speed of landfalling typhoons was 20% higher than normal. With continuous progress in disaster prevention and mitigation, the proportion of annual direct economic losses caused by meteorological disasters relative to GDP fell from an average of 3.3% in the 1990s to 0.6%; the number of deaths and missing persons fell 62% compared to the 1990–2011 average.

Air cleanliness is an important standard for measuring the climate and ecological environment. Data show that although climate conditions in the past five years were unfavorable for clearing air pollutants, smog processes and PM2.5 concentrations showed a downward trend. For example, the annual average PM2.5 concentration at the Shangdianzi atmospheric background station in Beijing was 37.2 micrograms per cubic meter, down 10% from the previous five years (2007–2011). In 2016, the average concentration was the lowest since records began in 2005.

Satellite monitoring shows that vegetation coverage nationwide has increased over the past five years, with over 80% of areas improving. Vegetation coverage in desertification areas also increased, and 92% of desertified land showed ecological improvement, with vegetation conditions significantly improving. In the Four Regions of the Three-North Shelterbelt (Northeast, North China, Northwest, and Xinjiang), average vegetation indices over the past five years increased by 2.0%, 7.8%, 7.5%, and 7.0% respectively compared to the previous five years.

10

2015 Was a Critical Year for China’s Climate Change Response and Low-Carbon Development

The selection event for the “Top Ten News of China’s Climate Change Response and Low-Carbon Development in 2015,” organized by China Economic Herald, Beijing Institute of Circular Economy, the School of Management of Capital Normal University, and the editorial board of China Low Carbon Yearbook, was recently announced.

Representatives from government departments, experts in the field of climate change, and reporters and editors from major central news media participated in the selection. After rigorous preliminary and final reviews, the “Top Ten News of China’s Climate Change Response and Low-Carbon Development in 2015” were selected as follows:

The Fifth Plenary Session of the 18th CPC Central Committee included “green development” as one of the “Five Development Concepts” and proposed promoting low-carbon circular development.

President Xi Jinping delivered an important speech at the Paris Conference, playing a significant political role in advancing climate change negotiations, and the Chinese delegation made outstanding contributions to the conclusion of the Paris Agreement.

China submitted its Nationally Determined Contribution (NDC) document on climate change to the United Nations, announcing its 2030 action goals and major measures.

During the “12th Five-Year Plan” period, China’s energy intensity and carbon intensity per unit of GDP dropped significantly, the proportion of non-fossil energy increased markedly, mitigation of climate change achieved remarkable results, and the capacity to cope with climate events was further strengthened.

The government vigorously promoted the development of new energy vehicles, which saw explosive growth.

The pilot carbon emissions trading programs accelerated, laying a solid foundation for establishing a national carbon trading market in 2017.

The energy-saving and emission-reduction effects of China’s high-speed rail improved significantly, with the third-generation rail transit traction technology system put into operation.

China became the third country in the world to master blue LED technology, opening up a new path for saving lighting energy and reducing carbon dioxide emissions.

A strong El Niño caused frequent extreme weather events nationwide, with widespread smog in northern China. Joint prevention and control efforts in the Beijing-Tianjin-Hebei region were fully mobilized to combat air pollution.

Publicity and education on climate change response were widely carried out, the concept of green and low-carbon development took deeper root in people’s hearts, and public participation continued to increase.

2018.2.5-2018.2.11

1

Millions of Years Ago, Climate Change Drove Citrus Trees From the Himalayas to the Rest of the World

According to a new study, sudden climate change that occurred 6 to 8 million years ago caused citrus trees to migrate from the Himalayas to other parts of the world. As citrus trees spread, they changed, and eventually brought sweet orange juice to our kitchen tables.

In order to better understand the origin of citrus trees, scientists have mapped the genomes of more than 50 varieties of citrus fruits, including clementines and bergamots.

They found that today’s citrus trees originated from at least ten ancestral species in the southeastern foothills of the Himalayas. According to a study published Wednesday in Nature, this region includes eastern Assam, northern Myanmar, and western Yunnan. Millions of years ago, the weakening of the Himalayan monsoon made the weather drier. This triggered the spread of citrus trees across Southeast Asia, where new species evolved to adapt to different environments.

Today, citrus trees are among the most widely cultivated fruit trees in the world. However, until now, the origin of citrus trees has remained unclear. Daniel Rokhsar, a geneticist at the University of California, Berkeley, and co-author of the study, said: “Fortunately, genetics can allow scientists to see a glimpse of citrus history.” But this new study not only provides a history of how citrus spread over time; it also offers growers a genetic roadmap to create high-quality, pest-resistant new citrus varieties.

2

The Peril of Climate Change! Polar Bears May Face an “Existential Crisis”

With global warming, the sea ice area in the Arctic is rapidly shrinking, posing a serious threat to the survival of polar bears. Experts say that due to the disappearance of sea ice and food shortages, polar bears may face an “existential crisis.”

Although polar bears are good swimmers, they are not aquatic animals and must rely on sea ice to survive. Scientists once discovered nine swimming polar bears off the northwest coast of Alaska, with the farthest one already 60 miles from shore. Satellite images showed that sea ice in this area had almost completely disappeared. Under normal conditions, polar bears can swim 10 to 50 miles, but swimming 50–100 nautical miles makes it difficult for them to reach land safely, and they may drown.

As global average temperatures rise, the rate of ice melt around the Arctic accelerates, and the polar bears’ territory is continually “eroded,” making food increasingly difficult to find. Many times, in order to hunt, they are forced to swim about 100 kilometers. Scientists state that only the strongest adult males can complete such long migrations, while the weaker and younger ones die along the way.

In addition, the reduction of sea ice greatly limits the platform polar bears use to hunt seals, making it impossible for them to obtain enough food. A recent research report published in Science by the U.S. Geological Survey and the University of California, Santa Cruz, showed that due to climate change altering their living environment, polar bears’ metabolism is faster than previously thought, and because they cannot find sufficient food, polar bears may face extinction earlier than expected.

3

How Much Do You Know About Climate Change?

Scientists always tell us that global warming and the Earth’s environment are already on the brink of danger, and that human factors are the culprits behind these malignant changes.

But how exactly does burning coal or cutting down forests lead to glaciers melting, sea levels rising, and seawater overheating thousands of miles away? Let’s summarize some key facts about climate change.

Greenhouse gases in the atmosphere act like a layer of insulation, making Earth’s climate more suitable for life. They can absorb the sun’s warmth directed at the Earth, retain part of the heat, and prevent all energy from escaping into space through back radiation. Since the Industrial Revolution, human activities have relied heavily on burning fossil fuels for production and daily life. This has disrupted the original balance of the atmosphere, dramatically increasing the amounts of methane, nitrous oxide, and fluorinated gases. But the real driver of global temperature rise is carbon dioxide.

China and the United States are the countries that emit the most greenhouse gases, followed by the European Union. The burning of coal, oil, and natural gas is the biggest cause of excessive carbon dioxide emissions. Agriculture, cement production, livestock, and deforestation also increase the amount of carbon dioxide in the air.

Human industry has caused too many greenhouse gases to enter Earth’s atmosphere, preventing heat from escaping. It is like trying to sleep comfortably with just a light summer blanket, but instead covering yourself with a five- or six-kilogram heavy quilt. Our planet, wrapped in this thick blanket, is sweating profusely and its “temperature” is rising rapidly.

With every breath we take, we release carbon dioxide — something unavoidable in our daily lives. The oceans can absorb carbon dioxide, and plants consume carbon dioxide during photosynthesis. This forms a carbon cycle with both production and consumption. However, the speed and volume of carbon dioxide generated by industrial production are too fast and too high, making it impossible for oceans and plants to absorb it at their natural rate.

4

Ten Major Events in the Climate and Energy Sector in 2017: Who Was the Real “Gray Rhino”?

Looking back on 2017, “monster” hurricanes, record-breaking high temperatures, and heat waves showed us the destructive power of climate change as a “gray rhino” crisis. We also witnessed governments providing political momentum for global climate governance through multilateral cooperation, as well as non-state actors across society taking action to explore solutions for responding to climate change.

Over the course of a year, people gradually realized that compared to the direct impacts of climate change and the global trends in addressing it, the Trump administration’s ability to intervene was very limited. Here, we review ten major events in climate change and the energy sector in 2017 from the perspectives of climate impacts, global climate governance, and new energy industry development. We can see that climate change, as a “gray rhino” event, is continuously urging human action, while a future supported by clean and sustainable energy is beginning to emerge.

Our review highlights major low-carbon and energy events that may influence the future direction of energy development. We found that significant events in the low-carbon, climate change, and energy fields occurred mostly in Asia, especially between China and EU countries. This shows the new changes in renewable energy development and climate policy after Trump withdrew from the Paris Agreement.

On June 1, 2017, Trump announced in the White House Rose Garden that the United States would withdraw from the Paris Agreement on climate change, claiming he would revive the coal industry and “make America great again.” This move left the U.S. unprecedentedly isolated at international meetings such as the UN, G7, and G20. In response, 125 U.S. cities, 9 states, 902 businesses, and 183 universities issued a joint statement, “We Are Still In,” forming an alliance representing 120 million Americans and $6.2 trillion of GDP. This alliance continues to push for America’s transition to clean energy and the implementation of climate goals.

5

British Media: China’s Determination to Reduce Pollution and Emissions Is Unprecedented, and the Trend Has Shifted

When it comes to difficult government work, few jobs are as tough as that of Xie Zhenhua, head of China’s climate negotiation delegation. On the day he agreed to an interview, Beijing’s air quality was “unhealthy.” At least, that’s what the air quality monitoring app on smartphones showed. Beijing residents check this app as frequently as Londoners check the weather forecast.

Most of the smog comes from cars and coal-fired power plants that helped boost China into becoming the world’s second-largest economy. In 2012, China’s carbon dioxide emissions exceeded the combined total of the United States and the European Union, accounting for nearly one-third of global emissions.

This puts Xie in a difficult position. For most of the past decade, the 64-year-old engineer has represented China in international climate negotiations aimed at reducing emissions.

It is well known that when faced with unrealistic demands from other countries, Xie would pound the table during climate talks. But now, he says, regardless of external pressure, the pollution seen daily outside the window forces China to make changes. “Sometimes the international community doubts whether China will truly take action. There should be no doubt on this issue. China will definitely take action — not only to protect people’s livelihoods and health, but also to contribute to global efforts to mitigate climate change,” he said.

Indeed, China has made remarkable progress in shifting from coal to cleaner power. Last year, China added 94 million kilowatts of new installed capacity, nearly 60% of which came from renewable energy. This included about 11 million kilowatts of solar power, enough to power a small Chinese city.

After 30 years of rapid economic growth, China still has nearly 100 million people living in poverty. Before reaching a standard of living comparable to the West, the idea of China reducing emissions as quickly as the West is extremely unpopular. “China is not Chad, but on the other hand, China is also not the United States, the EU, or Japan,” Xie said.

6

Wang Shi: “My Life Has Entered the ‘Third Stage,’ with Public Welfare as the Theme”

On the evening of January 23, Wang Shi, the founder of Vanke Group, who had long stayed away from the public eye, appeared at the Beijing Water Cube. He delivered a nearly four-hour-long public speech titled Return to the Future, in which he talked about his entrepreneurial journey, life reflections, and future plans. The venue was packed with an audience.

In his speech, Wang stated that for many entrepreneurs, it is not that the company cannot do without you, but rather that you cannot do without the company — because you have lost yourself, your sense of existence, and your sense of value. In fact, for every entrepreneur, letting go is only a matter of time and inevitability. Human life, including professional life, is limited. In the end, you must let go, but that is passive. Actively letting go is the true test.

After a long period of silence, Wang Shi has returned to the public eye, declaring in a public way that he has entered the second half of his career.

On January 19, 2018, he resigned as an independent director of BGI Genomics (300676). He has already made clear that the main theme of the “third stage” of his life will be public welfare.

That evening, in his speech, he said that charity and public welfare are not merely about the relationship between giving and receiving, not merely about giving money or goods, but rather about equal attention and mutual giving.

On June 30, 2017, Wang formally stepped down as chairman of the board of Vanke Group. The next day, he announced that he would serve as chairman of the Vanke Foundation. In addition, he holds multiple public welfare titles, such as chairman of the council of the One Planet Foundation in Shenzhen, president of the Mangrove Conservation Foundation, and the second president of the SEE Foundation.

His presence can be seen at various charitable and public welfare events: participating in low-carbon forums, attending UN climate conferences, conducting field research in nature reserves, discussing medical aid and clean energy projects with local governments, as well as attending gender equality conferences to explore social responsibilities in promoting the development of women.

7

A “Bomb” Buried Under the Arctic Seafloor May Accelerate Global Warming

You may have noticed that in swamps or ponds, bubbles sometimes rise from the mud. These bubbles may look insignificant, but they are closely related to global warming. Their true identity is methane. Methane is an important greenhouse gas, with a greenhouse effect more than 20 times stronger than carbon dioxide. Recently, a new study showed that methane eruption events between Norway and the Arctic may have occurred over the past 8,000 years.

Beneath the Arctic seafloor lies an important resource known as “combustible ice,” which is natural gas hydrate. At its core, one of its main components is still methane. Research from the Centre for Arctic Gas Hydrate, Environment and Climate (CAGE) shows that methane eruption events over millennia are the result of the melting and leakage of combustible ice under the seafloor. The methane, frozen in icy water, was released in large quantities, and such eruption events occurred at the end of the last Ice Age.

In the past, scientists believed that the reason for methane release in the Arctic was ocean warming, and they attributed large-scale methane leakage to human causes. However, this new study published in Nature Communications indicates that the actual situation is more complicated. Methane hydrates can form because of low temperatures and immense pressure under the seafloor, which is why they exist in the deep ocean. At the end of the last Ice Age, as the ice sheet shrank, the pressure on the seafloor changed.

This change destabilized the methane hydrates. The hydrates began to dissolve, releasing methane. Researchers used ice sheet models to develop their theory and concluded that methane hydrates have been leaking methane for at least 8,000 years. They explained that methane release on the seafloor of areas such as the Svalbard archipelago may have lasted for a long time, with the melting of hydrates causing slow and steady seepage.

8

Scientists Study: The Sun May Dim Within the Next 30 Years, Leading to an Ice Age

According to The Weather Network and a February 9 report by the Daily Mail, in recent years, natural disasters caused by global warming have frequently been the focus of public concern. Recently, a scientist’s new study suggests that the sun’s 11-year activity cycle goes through active and quiet phases. It is expected that within the next 30 years the sun may dim, leading the Earth into a “mini ice age.”

Currently, scientists at the University of California, San Diego, say that the timing of the next solar minimum has been accurately predicted: in 2050, the sun may become “abnormally cold.” This next cold period is called a “grand minimum,” during which the Earth may experience conditions similar to those in mid-17th century Europe. At that time, the phenomenon was known as the “Maunder Minimum,” when temperatures were so low that London’s Thames River froze. Meanwhile, the Baltic Sea also froze to the extent that Swedish troops were able to march across the ice to invade Denmark in 1658.

Scientists say the conditions during the “grand minimum” will resemble those of a “mini ice age.” Physicist Dan Lubin, who led the study, analyzed the “Maunder Minimum” and pointed out that humans may face an even worse situation than in the mid-17th century. Specifically, the sun may become dimmer than in previous solar minima.

The study noted that the likelihood of the sun entering a new minimum is very high, because the recent pattern of declining sunspots resembles that of earlier minima. When the sun is in a “maximum” phase, nuclear fusion at its core releases more magnetic loops to the surface due to extreme ultraviolet wavelengths. But when the sun is in a “minimum,” its magnetic power weakens, sunspot formation decreases, and ultraviolet radiation reaching the surface decreases accordingly. As a result, the sun’s surface appears dimmer.

9

Shaanxi Province Issues Action Plan for Meteorological Support of Ecological Civilization Construction

The plan will strengthen the foundation of meteorological services for ecological civilization construction by improving comprehensive ecological meteorological monitoring capacity, enhancing intensive business support capacity, and establishing a sound organizational system for ecological meteorological services.

In order to further improve meteorological services for ecosystem protection, the plan will actively carry out services for ecosystem protection and restoration, and strengthen services for climate change and extreme weather events. It will gradually improve the artificial weather modification system for ecological environment protection and restoration.

The plan also seeks to fully leverage the role of meteorological services in supporting green development: providing meteorological support for green urbanization, strengthening meteorological services for rural revitalization, carrying out meteorological support for the construction of national parks, and strengthening climate feasibility assessments for major plans and key projects.

Furthermore, the plan will strengthen meteorological forecasting services for atmospheric environment management, and improve the level of meteorological support for air pollution prevention and control. It will also strengthen the legal responsibilities of ecological civilization meteorological services and raise the level of legal governance in ecological civilization meteorological support.

It is expected that by 2020, Shaanxi’s ecological meteorological monitoring system will be improved, interdepartmental integration mechanisms will be further strengthened, and scientific and talent support for ecological civilization meteorological construction will be further enhanced. The capacity for meteorological support of ecological civilization construction will be significantly improved.

10

The Ten Least Environmentally Friendly Foods

The Natural Resources Defense Council (NRDC), an international environmental organization, recently released a report on CNN. By analyzing 197 kinds of food and calculating the carbon dioxide emissions per kilogram of food, it ranked the ten most environmentally harmful foods in the United States.

Beef. NRDC rated beef as the most environmentally destructive food. Consuming 1 kilogram of beef produces 26.5 kilograms of carbon dioxide emissions, five times higher than chicken and turkey. According to data from the Food and Agriculture Organization of the United Nations (FAO), livestock accounts for 14.5% of global greenhouse gas emissions, making it one of the main causes of climate change. Of this 14.5%, 65% comes from beef and dairy cattle. Sujatha Bergen, one of the study’s lead authors, explained that beef is the culprit mainly because of feed. Cattle feed requires large amounts of pesticides and fertilizers, whose main components are petroleum products. When fertilizer decomposes, it releases large amounts of greenhouse gases. In addition, the digestive systems of cows emit large quantities of methane, a greenhouse gas 25 times more potent than carbon dioxide.

Lamb. NRDC estimated that consuming 1 kilogram of lamb produces 22.9 kilograms of carbon dioxide. Feed is again the main reason. Lamb feed consists primarily of corn and soybeans, and growing these crops requires fertilizer. Fertilizer decomposition produces nitrous oxide, a greenhouse gas 298 times more potent than carbon dioxide.

Butter. Consuming 1 kilogram of butter produces 12 kilograms of carbon dioxide, mainly from the production chain. Bergen said that producing butter requires separating colostrum into low-fat milk and cream, then pasteurizing, freezing, fermenting, and churning it through a series of steps. This process consumes energy and increases carbon emissions.

2021.11.15-2021.11.21

1

Contributing China’s Wisdom to Global Climate Response

On the evening of November 13 local time, after being extended by one day, the 26th Conference of the Parties (COP26) to the United Nations Framework Convention on Climate Change (UNFCCC) closed in Glasgow, United Kingdom. The conference adopted a decision document and reached a consensus on the implementation rules of the Paris Agreement.

COP26 was the first session of the Conference of the Parties held since the Paris Agreement entered the implementation stage. Over nearly two weeks, parties worked together to bridge differences and expand consensus, ultimately reaching agreement on the implementation rules of the Paris Agreement, laying a solid foundation for its implementation. The conference also adopted several other documents, including the Glasgow Leaders’ Declaration on Forests and Land Use.

In addition, all parties agreed to extend the long-term finance agenda until 2027. Developed countries will continue to undertake existing obligations before 2025 and complete arrangements for new quantified finance targets beyond 2025 by 2024. The conference also decided to establish and immediately launch the two-year Glasgow–Sharm el-Sheikh Work Programme on the Global Goal on Adaptation, to implement the requirements of the Paris Agreement on adaptation and enhance parties’ understanding of the global adaptation goal.

Although the conference achieved certain progress on adaptation and financial support, some core concerns of developing countries were not well addressed. As early as the Copenhagen Climate Change Conference in 2009, developed countries collectively pledged to provide at least $100 billion annually by 2020 to help developing countries cope with climate change. However, twelve years have passed, and developed countries have never truly delivered on this commitment. Many developing countries expressed disappointment during the conference, and negotiations on funding implementation still have a long way to go. On adaptation, developed countries continue to show a negative attitude toward the global goal on adaptation and still oppose making it a formal negotiation agenda item.

2

The “Forgotten Victims” of Climate Change

The surging Meghna River swallowed up Rashida Begum’s home, forcing her to leave Noakhali in southeastern coastal Bangladesh and relocate to the country’s second-largest city, Chittagong.

Begum rented a shack of less than 10 square meters in an illegal slum set up for climate refugees, and since then she has squeezed into it with her four children and husband.

“We have nothing. Apart from this overcrowded slum, we simply cannot afford a better place to live,” Begum said helplessly to The Scotsman recently.

Begum is clearly not an isolated case.

In Bangladesh, tens of thousands of people are forced to migrate every year because of climate disasters. The vast majority flood into the slums of Dhaka, the capital, and Chittagong, the second-largest city.

This South Asian country, with a population of 170 million, has contributed very little to historical carbon emissions, yet it has become one of the countries most affected by climate change. Increasingly frequent cyclones and heat waves, severe floods, and rising sea levels — the impacts of global warming are destroying the lives of millions of Bangladeshis. According to a World Bank report, by 2050 about 13.3 million Bangladeshis will become climate migrants, accounting for 37% of all migrants in South Asia.

As the global climate crisis worsens, more and more people are being forced from their homes by related natural disasters. This group is often referred to as “climate migrants,” though some call them “climate refugees.” The UN Refugee Agency refers to them as “people displaced in the context of climate change and disasters.”

The 26th UN Climate Change Conference (COP26) has just concluded in Glasgow, UK. Several experts, in interviews with Beijing News, pointed out that the conference achieved some important results to continue advancing global climate governance. However, it still fell short in pushing developed countries to help less-developed countries cope with climate change and compensate them for the losses they suffer due to it.

3

Victims, Accomplices, and Answers to Climate Change and Nature’s Destruction

We are facing intertwined crises: climate change, biodiversity loss, land degradation, food security, and social inequality. Changing our food and agricultural production and consumption patterns, transitioning to sustainable land-use practices, and emphasizing nature-based solutions are important pathways to mitigating and adapting to global climate change and restoring biodiversity.

We live today in a world where food, agriculture, and land use are the main factors driving climate change, natural destruction, and biodiversity loss.

23% of net greenhouse gas emissions come from changes in agriculture, forestry, and other land use [1]. If all the cows in the world were counted as a country, its carbon emissions would almost equal those of the United States [2].

60% of tropical deforestation is caused by the expansion of farmland for commercial agriculture [3]. The Amazon rainforest, covering more than 2 million square miles, has long been an important and reliable carbon sink. However, according to a decade-long study recently published in Nature, due to deforestation, climate drying, and forest fires, southeastern Amazonia has, for the first time in history, released more carbon dioxide than it absorbed [4].

Food, land, and ocean systems also threaten 72% of the world’s endangered species [5]. For example, due to long-term land reclamation from lakes, sand mining, quarrying, and navigation, species such as the Yangtze River dolphin, Chinese paddlefish, and Reeves shad have become functionally extinct, while the Yangtze finless porpoise and Chinese sturgeon are critically endangered.

Despite this, we must further develop agriculture to ensure food security and provide employment.

The State of Food Security and Nutrition in the World 2019 report noted that hunger levels in Africa have risen by nearly 20%, with malnutrition being very serious. Worldwide, more than 820 million people still suffer from hunger, nearly one-ninth of the global population.

4

China’s Contribution Helps Launch a New Journey for Global Climate Response

After being extended by one day, the 26th Conference of the Parties to the United Nations Framework Convention on Climate Change (UNFCCC) concluded in Glasgow on the night of November 13. The conference adopted a package of decisions on the implementation rules of the Paris Agreement, marking the beginning of a new journey for the international community to fully implement the Paris Agreement. As a responsible major country, China played an active and important role in promoting global climate action and contributed to the success of the conference.

However, because developed countries failed to fully respond to the core concerns of developing countries on adaptation, finance, and technology support, the conference still had regrets and shortcomings. Global climate response still faces many challenges.

Significance: A New Journey Begins for Global Climate Response

This conference was the first session of the Conference of the Parties held since the Paris Agreement entered the implementation stage. It was a meeting that linked the past and the future. The international community had high expectations for this conference to complete negotiations on the implementation rules of the Paris Agreement and further promote its comprehensive, effective, and balanced implementation.

Over nearly two weeks, parties worked together to bridge differences and expand consensus, ultimately reaching agreement on the implementation rules of the Paris Agreement. These included negotiations on leftover issues such as market mechanisms, transparency, and a common timeframe for nationally determined contributions, laying a solid foundation for the implementation of the Paris Agreement.

Zhao Yingmin, head of the Chinese delegation and vice minister of the Ministry of Ecology and Environment, told Xinhua after the meeting that the decision documents adopted at this conference, especially the agreement on the implementation rules of the Paris Agreement, are of great significance for upholding multilateralism and focusing on its implementation. They will launch a new journey for the international community to fully implement the Paris Agreement.

5

Study: Climate Change Causes Birds to Shrink in Size and Grow Longer Wings to Cool Down

Data show that in October, the area of deforested trees in the Amazon rainforest reached a new high. New research has also found that even in the most pristine areas of the Amazon untouched by humans, birds are shrinking in size and growing longer wings due to the impact of climate change.

According to data released by Brazil’s National Institute for Space Research, 877 square kilometers of trees were cut down in the Amazon rainforest in October, more than half the area of Rio de Janeiro. This was a 5% increase compared to the same period in 2020 and the highest level since the agency began recording logging in 2016.

Reports said that in 2020 the deforestation rate in the Amazon surged, mainly due to mining and agricultural activities. The situation in 2021 may be even worse.

A study published in Science Advances pointed out that even the most pristine areas of the Amazon are affected by climate change. Over the past 40 years, the hotter and drier environment of the Amazon rainforest has affected birds’ diets and physiology, making their bodies smaller and their wings longer.

The report’s lead author, ecologist Vitek Jirinec from the Integral Ecology Research Center, said: “For me, the biggest key is that this is happening in the heart of the world’s largest rainforest, far away from direct human disturbances such as deforestation… This is worth deep reflection.”

Over the past 40 years, Jirinec and others analyzed data from more than 15,000 birds that were captured, weighed, and tagged. They found that since the 1980s, the body weight of almost all bird species has declined.

Most birds have lost an average of 2% of their body weight every decade. For example, birds that weighed 30 grams in the 1980s now weigh an average of 27.6 grams.

6

Implementing Action Is Key to Addressing Global Climate Change

The 26th Conference of the Parties (COP26) to the United Nations Framework Convention on Climate Change (UNFCCC) recently concluded. Through the joint efforts of all parties, a package of decisions on the implementation rules of the Paris Agreement was finally adopted. As a responsible major country, China has always engaged in active communication and consultations with relevant parties in a constructive manner, playing a positive role in ensuring the successful outcome of the conference. However, in terms of fully implementing the Paris Agreement, the international community, especially some developed countries, still needs to strengthen concrete actions.

This conference was the first session of the Conference of the Parties since the Paris Agreement entered the implementation stage in 2015, and the international community had placed high expectations on it achieving substantive results. Over nearly two weeks, parties worked hard to bridge differences and expand common ground, completing negotiations on the implementation rules of the Paris Agreement that had dragged on for six years. These included outstanding issues such as market mechanisms, transparency, and a common timeframe for nationally determined contributions. More than 50 relatively balanced political outcome documents were adopted, including the “Glasgow Climate Pact,” further building international consensus on promoting low-carbon energy transitions and laying the foundation for the comprehensive and effective implementation of the Paris Agreement.

Nevertheless, there remain regrets and shortcomings at this conference. The main issue is that developed countries still failed to adequately respond to the core concerns of developing countries. As early as COP15 in 2009, developed countries had pledged to jointly mobilize at least $100 billion annually by 2020 to help developing countries cope with climate challenges. However, this promise has never truly been fulfilled, and negotiations on financing implementation still have a long way to go. On adaptation, developed countries have also failed to confront the urgent reality that developing countries generally have weak capacity to adapt to climate change. They continue to hold a negative attitude toward the global adaptation goal and still oppose making it a formal negotiation agenda item.

7

British Media: Climate Change Affects Daily Life—Reduced Incomes, Job Losses, and Rising Prices

Climate change is not only a major political, environmental, and survival issue for humanity; it also concerns every aspect of our daily lives, directly impacting our expenses and incomes.

According to a report by the BBC, just look at whether the prices of daily necessities on your shopping list are rising, and whether your monthly bills for water, electricity, and gas are increasing. If you happen to live in an area frequently hit by extreme weather, you may even face the risk of unemployment or losing your home.

BBC journalists reviewed decades of climate data and found that over the past 40 years, extremely hot weather has become increasingly frequent. The number of days with maximum temperatures exceeding 50°C has doubled, and more regions are experiencing such extreme climates.

The Intergovernmental Panel on Climate Change (IPCC) in its assessment report on the state of climate change pointed out that if all possible emissions scenarios occur, the global temperature increase will reach 1.5°C by 2040. If emissions are not reduced in the coming years, this will happen even earlier.

In recent years, as extreme weather events have become more frequent, floods, droughts, wildfires, typhoons, and heat waves have occurred more often and affected a wider range of regions.

A report released on November 4 by the Food and Agriculture Organization of the United Nations showed that the world food price index surged to a new peak, reaching its highest level since July 2011.

The U.S. National Centers for Environmental Information recently published statistics showing that in the first nine months of 2021, the United States experienced 18 climate disasters, each causing over $1 billion in damage.

According to the center’s statistics, from 1980 to 2000, the U.S. experienced about 7 such extreme climate disasters annually. But in the five years from 2016 to 2020, the average rose to 16 per year. Since 2017, insured losses have exceeded $370 billion. As a result, the insurance industry predicts that premiums will inevitably rise significantly in the future.

8

Cold Air Arrives Again! Where Is the Global Warming Everyone Talks About?

Cold air has arrived once more. After experiencing the “flash freeze” at the beginning of the month and the “thick” gift of the first snow, are you still unfamiliar with La Niña? Have you gained any emergency experience in keeping warm? Will you still scramble to dig out thick clothes? Can you quickly find your “beloved car” in the middle of a blizzard?

According to reports, on the 20th, the China Meteorological Administration again issued a blue cold wave warning. Over the next three days, strong cold air will move from northwest to southeast, affecting central and eastern China with strong winds and significant temperature drops. Over the following two days, central and eastern Inner Mongolia, and northeastern regions will experience light to moderate snow or sleet. On the 22nd, some areas in central and eastern Heilongjiang and eastern Jilin will see heavy snow, while northeastern Heilongjiang may see extremely heavy snow.

Looking back at the cold wave at the beginning of the month and the first snowstorm of this winter, while many still shiver at the memory of the cold, some netizens have raised questions: Is extreme cold weather related to “double La Niña”? Will it help disperse smog? Does it mean that global warming is no longer happening?

Faced with the forceful arrival of the cold wave, netizens’ responses were vivid and helpless: “The cold wind is slapping my face randomly,” “It’s really so cold I could cry.”

According to the National Climate Center, this winter will see a weak-to-moderate La Niña event. Given that a La Niña event already occurred in autumn and winter of 2020–2021, 2021 will be a “double La Niña year.”

La Niña refers to a phenomenon in which sea surface temperatures in the equatorial central and eastern Pacific become abnormally cooler over a wide range, with the intensity and duration meeting certain thresholds.

In most winters when La Niña reaches its peak, cold air activity affecting China is more frequent and stronger than usual. The probability of temperatures in most parts of central and eastern China being lower than average is therefore higher. For tropical and subtropical regions, La Niña events affect water vapor conditions in southern China, making them significantly worse than usual, which is largely unfavorable for precipitation.

9

Inner Mongolia Advances Climate Change Response During the 14th Five-Year Plan with Multiple Measures

The General Office of the People’s Government of Inner Mongolia Autonomous Region recently issued the 14th Five-Year Plan for Climate Change Response in Inner Mongolia (hereinafter referred to as the “Plan”). The Plan comprehensively summarizes the positive progress and results achieved during the 13th Five-Year Plan in promoting actions across various fields to address climate change, analyzes the situation faced during the 14th Five-Year Plan, and further clarifies the goals and key tasks for climate change response during this period.

The Plan specifies that by 2025, Inner Mongolia will have initially formed a new framework for climate change response that is aligned with ecological civilization construction, coordinated with high-quality development, integrated with ecological and environmental protection, synergistic with energy conservation and emission reduction, and consistent with carbon peaking and carbon neutrality efforts. Carbon emissions in key industries, fields, and regions will peak first, the leading role of low-carbon pilot and demonstration projects will be significantly strengthened, adaptation capacity to climate change will be further improved, and governance systems and capacities for climate change will be effectively enhanced.

The Plan also refines and quantifies targets, identifying 12 main indicators, including nine binding and three expected ones, with clear target values for 2025.

To achieve these goals, Inner Mongolia has further clarified its key tasks. These include:

Mitigation: controlling greenhouse gas emissions through nine areas such as carbon peaking actions, building a green and low-carbon industrial system, building a clean and low-carbon modern energy system, strengthening source control of energy conservation, controlling CO₂ emissions in key industrial sectors, controlling CO₂ emissions in urban and rural construction, controlling CO₂ emissions in transportation, effectively controlling non-CO₂ greenhouse gas emissions, and increasing ecosystem carbon sinks.

Adaptation: improving climate change adaptation through six areas such as enhancing urban adaptation capacity, agricultural and pastoral adaptation capacity, forestry adaptation capacity, adaptation in ecologically fragile areas, public health adaptation capacity, and strengthening disaster prevention and mitigation systems.

Regional development: advancing regional low-carbon development pilot demonstrations by promoting integrated green development and deepening low-carbon pilot demonstration projects.

10

Contributing China’s Wisdom to the Global Climate Response

At the World Leaders Summit held after the opening of the conference, China put forward three proposals on how to address climate change and promote global economic recovery in this era: uphold multilateral consensus, focus on pragmatic action, and accelerate green transformation. These proposals won wide praise from the international community.

During the conference, China consistently engaged in active communication and consultations with relevant parties in a constructive manner. The joint document issued by China and the United States on bilateral climate cooperation laid a solid foundation for the success of the conference, effectively boosting confidence among all parties in jointly tackling climate change, and constructively advancing the conference process by injecting momentum into bridging differences and expanding common ground. The Chinese delegation actively consulted and coordinated with the United Nations, the UNFCCC Secretariat, the UK presidency of the conference, and all parties, safeguarding the principles established by the UNFCCC and the Paris Agreement, such as common but differentiated responsibilities, and protecting the rights and interests of developing countries. In doing so, it played a constructive role and contributed to the success of the conference.

Zhao Yingmin, head of the Chinese delegation and vice minister of the Ministry of Ecology and Environment, stated after the meeting that the decision documents adopted at this conference, especially the agreement on the implementation rules of the Paris Agreement, are of great significance for upholding multilateralism and focusing on its implementation. They will launch a new journey for the global climate response. As a participant, contributor, and leader in global ecological civilization, China will unswervingly follow a path of high-quality development that prioritizes ecology and pursues green and low-carbon growth, continuously contributing to the global response to climate change. China will, as always, uphold multilateralism, firmly abide by the principles established by the UNFCCC and the Paris Agreement, and do its utmost to help other developing countries vigorously develop renewable energy, achieve transitions to clean energy systems, and jointly enhance capacity to address climate change.

2022.2.7-2022.2.14

1

At the Beginning of 2022, Tonga’s Volcanic Eruption and Extreme Snowstorms in the U.S. Cast a Shadow—Is a Major Climate Shift Coming?

At the very start of 2022, the volcanic eruption in Tonga and extreme snowstorms in the United States once again cast a shadow over many countries. Some have even begun to question whether the climate in 2022 will undergo major changes—could large-scale cooling be coming to Earth?

According to a Xinhua News Agency report on February 7, in recent days, multiple regions in the U.S. Midwest, Northeast, and South were hit by snowstorms. The report emphasized that due to the impact of the snowstorm, as of February 5 local time, more than 100,000 users were still without power.

In fact, the United States already suffered a winter storm in 2021, and unexpectedly, at the very beginning of 2022, another winter storm occurred, greatly affecting the daily lives of many people. It is reported that the governor of New York State has urged residents to stay home as much as possible, avoid driving, and emphasized that the “danger period” of the winter storm has not yet passed and weather conditions remain highly uncertain. Meanwhile, on February 4, a U.S. mountaineering rescue association issued a warning that with the arrival of the winter storm, the risk of avalanches on mountains in states such as Vermont had increased, and people should remain highly alert.

NASA, based on relevant data analysis, stated that the winter storm currently hitting the U.S. is far stronger than the one in 2021. Moreover, this storm took an unusually rare route, stretching 3,000 kilometers across the country, bringing heavy snow, freezing temperatures, and other extreme climate phenomena to more than a dozen states, causing serious impacts in many areas. According to Global Times, as of February 4 local time, total snowfall in dozens of American cities had already reached double digits, and thousands of flights were canceled as a result.

2

Research on the Connection Between Addressing Climate Change and Arms Control

Climate change and the arms race are common challenges currently faced by humanity. At first glance, these appear to be two separate issues, but in fact there is an intrinsic link between them. Both responding to climate change and promoting arms control require adherence to the fundamental philosophy of building a community with a shared future for humankind. At the research level, the two disciplines are also directly connected. This article introduces studies on the relationship between climate change response and arms control, two key areas of global governance, through three examples.

The impact of U.S. partisan politics on its arms control and climate policies

As the world’s only superpower, the United States plays a vital role in global governance. Since the end of the Cold War, U.S. arms control policy has fluctuated under the influence of partisan politics. Broadly speaking, Democrats tend to rely more on arms control, while Republicans are more skeptical of it. U.S. climate policy has shown almost identical characteristics.

For example, the Clinton administration actively participated in negotiations on the Comprehensive Nuclear-Test-Ban Treaty and was the first to sign it. The Clinton administration also strongly supported the UNFCCC and its Kyoto Protocol.

By contrast, the George W. Bush administration was skeptical of the Comprehensive Nuclear-Test-Ban Treaty, withdrew from the Anti-Ballistic Missile Treaty, and refused to sign the Kyoto Protocol. The Obama administration actively engaged in negotiations and reached both the Joint Comprehensive Plan of Action (the Iran nuclear deal) and the Paris Agreement on climate change. The Trump administration, however, withdrew from both agreements.

Currently, the Biden administration has returned to the Paris Agreement and is seeking to rejoin the Iran nuclear deal. In the U.S. Congress, partisan tendencies are also very clear. Examining the influence of American partisan politics by combining the two issues of climate and arms control helps us systematically understand and predict the correlation in U.S. policy across these two domains.

3

Global Warming Uncertainties—How Do They Affect Policy-Making?

The Donors Trust Fund arose as a response to the Tides Foundation, and it quickly amassed four times the funding and a more strategically organized board of directors. Its board included senior officials from some of the most important institutions in the conservative movement, such as the American Enterprise Institute, the Heritage Foundation, and the Institute for Justice. With startup funds provided by Charles Koch, they acted as a central committee coordinating grants.

Researcher Robert Brulle noted that as criticism of fossil fuel interests obstructing reforms grew louder around 2007, tens of millions of dollars in donations from Koch, ExxonMobil, and other fossil fuel stakeholders appeared to vanish from the visible arena of public opposition.

Meanwhile, an increasing amount of comparable anonymous funding from Donors Trust began to flow into financing the climate denial movement. Brulle found that in 2003, of the 140 organizations whose financial records he studied, Donors Trust accounted for just 3% of their funding sources. By 2010, that proportion had risen to 24%. Indirect evidence suggests that fossil fuel stakeholders funding climate denial deliberately concealed their involvement, although Brulle could not prove it. “Where all this money really comes from is a huge unknown for us,” he said. The Koch brothers also had close ties to Donors Trust.

Public records show that Koch foundations donated considerable sums to Donors Trust, and large amounts of cash were then dispersed to nonprofits favored by them. For example, in 2010, the single largest grant was $7.4 million to Americans for Prosperity, whose chairman was David Koch himself. That funding accounted for about 40% of Americans for Prosperity’s contributions that year—demonstrating that the so-called “genuine grassroots organization” was, in fact, a myth.

4

The Cultural Value of Climate Change Fiction

Since the 1990s, extreme weather and natural disasters have occurred frequently, and climate change has affected human survival in all aspects. Global climate governance has now become one of the world’s most prominent issues. Responding to climate change, protecting the Earth, and building a community with a shared future for humankind are the responsibilities of every global citizen. Beginning in the early 21st century, climate change fiction has emerged, imagining the future of Earth’s climate while guiding readers to reflect on and discuss today’s social issues.

Focusing on environmental risk

Climate change fiction is a type of speculative work about “risk,” focusing mainly on human factors, such as the impact of fossil fuel burning on the climate. It imagines risks even when their precise nature and extent remain highly uncertain. With its rich imagination, climate change fiction is based on scientific predictions, while also going beyond them, exploring the complexity and diversity of personal and collective experiences of risk. Here, “risk” is not synonymous with “disaster,” but rather “the anticipation of disaster,” predicting how disasters might threaten humanity in an unknown future.

One example is Flight Behavior (2012) by American writer Barbara Kingsolver, which highlights the impact of climate change on Earth’s flora and fauna. In the story, millions of monarch butterflies suddenly appear in a valley, attracting countless tourists who believe it is a “natural miracle.” However, an ornithologist offers a different interpretation: the butterflies appear in a place where they should not be, showing an abnormal migration pattern completely different from known routes. The only explanation is climate change. Climatic anomalies disrupted the monarchs’ large-scale migration, causing them to lose their way and face the danger of extinction. This phenomenon reveals ecological risks that will ultimately affect human survival. What people saw as a “natural miracle” was actually a “natural disease.” The butterfly event on the mountain illustrates how humans and other species together constitute a complex, interdependent ecosystem—and how climate change is threatening the normal functioning of that system.

5

“21st Century Lecture Hall” Focuses on “Climate Change and Carbon Peaking and Carbon Neutrality”

According to the Ministry of Science and Technology website, on January 20, the 21st Century Center held its first “21st Century Lecture Hall” event of 2022. Li Gao, Director-General of the Department of Climate Change Response of the Ministry of Ecology and Environment, was invited to deliver a keynote report on the theme of “Climate Change and Carbon Peaking and Carbon Neutrality.” The lecture was hosted by Huang Jing, Director of the 21st Century Center, who welcomed Li Gao back to the Center as a guest speaker, introduced his career experience and important contributions to advancing China’s work on climate change, and expressed the hope that through this event participants would gain a more comprehensive and accurate understanding of China’s overall arrangements and progress on carbon peaking and carbon neutrality.

Drawing on his long-term experience in organizing and advancing climate change response work, Li Gao gave a detailed introduction to the scientific basis of climate change and the process of international climate governance. He conducted an in-depth analysis and interpretation of the historical context, from the formulation and signing of the UNFCCC, to the Kyoto Protocol and the Paris Agreement, and finally to the comprehensive outcomes of COP26, the “Glasgow Climate Pact.” He emphasized the positive and constructive role that China has continuously played in promoting the building of a fair, reasonable, and win-win system of global climate governance.

Li Gao also provided a comprehensive and systematic overview of China’s main progress in addressing climate change, as well as the policies, actions, and measures for achieving carbon peaking and carbon neutrality. He highly commended the 21st Century Center for its long-standing contributions in conducting climate strategy research, participating in international climate negotiations, advancing research and development of key technologies such as carbon capture, utilization, and storage (CCUS), and promoting bilateral and multilateral cooperation on climate change. He expressed the hope that the Center would continue to play its role as a think tank and provide strong support for China’s climate change response and carbon peaking and neutrality efforts.

6

Global Warming Underway: Greenland’s 4.7 Trillion Tons of Glacier Melt—What Will Happen to Earth?

Global warming has been discussed for many years, but in fact, it is already underway. Currently, the global average temperature is rising year by year.

Danish scientists, through analysis of climate satellite data, found that from April 2002 to August 2021—nearly 20 years—the glaciers on Greenland, located in the North Atlantic, have been melting at an astonishing rate. In that period, a cumulative total of 4.7 trillion tons of glaciers have melted. This research has been published on the Polar Portal website.

So much glacier melt is enough to raise global sea levels by 1.2 centimeters. To put it in perspective, China and the United States have roughly the same land area; if these 4.7 trillion tons of ice covered more than 9 million square kilometers of land, the thickness would reach 0.5 meters.

Satellite images show that the melting of Greenland’s ice sheet mainly begins from the edges. Over time, the ice sheet on the island will become thinner and smaller in area.

As temperatures rise, Greenland’s weather is also changing, with rainfall accounting for an increasing proportion of precipitation each year. In August 2021, an unprecedented heavy rain occurred on the Greenland ice sheet. According to scientists, this was the heaviest rainfall on the ice sheet since 1950.

Globally, glaciers are mainly distributed in high-latitude and high-altitude areas of mid- and low-latitude regions, with an area of more than 16 million square kilometers—almost the size of Russia. Among them, the glaciers of Antarctica and Greenland account for 96% of the world’s total glacier area and 99% of the total ice volume.

7

Foreign Media Marvel at Beijing’s “Olympic Blue,” Saying China’s Air Quality Has Seen a “Tremendous Transformation”

China Daily reported on February 10 that Beijing’s “Olympic Blue” has left a deep impression on the international community. According to the Associated Press, at this month’s Beijing Winter Olympics, the clarity of the air was such that athletes could see the mountains surrounding the city—a tremendous transformation compared with ten years ago. This demonstrates the efforts China has made in the past decade to improve air quality.

According to a recent report by the Ministry of Energy, after China’s air quality problems drew widespread concern in 2013, the country launched an ambitious plan to improve air quality and pledged to combat pollution with strong measures. In February this year, a report from the University of Chicago’s Energy Policy Institute noted that China had adopted large-scale measures to address air quality, including stricter emission standards for coal-fired power plants, limiting the number of cars on the road to reduce vehicle emissions, and replacing coal-fired boilers with gas or electric heaters.

China’s government reports on air quality data have also shown significant improvement. Pei Jia (transliteration), a 30-year-old Beijing resident who enjoys outdoor activities, said that the improvement in air quality had lifted his mood. Last year, Beijing had 288 days of good air quality, compared with only 176 days in 2013.

China has pledged that by 2060 it will have fully established an economic system of green, low-carbon, circular development, as well as a clean, low-carbon, safe, and efficient energy system. At present, China has made major progress in controlling emissions and is rapidly developing clean energy sources such as wind and solar power.

8

Beijing Winter Olympics Organizing Committee Closely Monitors Weather Changes, Continues to Innovate Broadcasting Methods

Closely monitoring weather changes, preparing contingency plans as needed

At the beginning of a press conference, Zhao Weidong mentioned several touching stories recently seen through broadcast footage. He said: “These touching stories happen every day. The cold of ice and snow collides daily with the warmth of humanity—that is the Olympics. I sincerely wish athletes can not only surpass themselves in competition but also enjoy the games and cheer wholeheartedly.”

According to weather forecasts, in the coming days a cold front may move south, possibly bringing snow to Beijing and Zhangjiakou. Would such potential changes affect the scheduling of the Winter Olympics events?

Zhao Weidong responded: “On the premise of respecting natural laws, we will do our best to ensure the normal progress of events, as well as the safety and health of athletes, staff, and volunteers.”

He introduced: “First, we are striving to strengthen precise weather forecasting. At present, three-dimensional weather monitoring networks have been established in Zhangjiakou and Yanqing, aiming for minute-level accuracy in time and hundred-meter-level accuracy in space, to meet meteorological demands for events. Second, venue staff will submit relevant data to experts from international sports federations, who will decide whether to adjust schedules. Generally, snowfall does not affect the schedule. But in extreme weather, the Beijing Winter Olympics Organizing Committee will work closely with the International Olympic Committee, international federations, and Olympic Broadcasting Services to promptly initiate competition rescheduling procedures, minimizing adverse impacts.”

9

Energy Blockchain and Dual-Carbon Strategy Research | Climate Change May Push U.S. Household Air Conditioning Demand Beyond Power Capacity

Household air conditioning demand exceeding power capacity

According to a new study on household demand, climate change will lead to increased air conditioning use during U.S. summers. If states do not expand power capacity or improve efficiency, prolonged blackouts could occur during summer heat waves.

The study projected air conditioning use in the U.S. under scenarios where global temperatures rise 1.5°C or 2.0°C above pre-industrial levels. It found that overall U.S. air conditioning demand could rise by 8% at the lower level and 13% at the higher level. The study was published in Earth’s Future, a journal of the American Geophysical Union (AGU), which focuses on interdisciplinary research on the past, present, and future of Earth and humanity.

The Intergovernmental Panel on Climate Change (IPCC), in its 2021 assessment report, pointed out that greenhouse gases emitted by humans would warm the global climate by more than 1.5°C as early as the early 2030s. Without significant measures, by the end of the 21st century, global warming may exceed the critical threshold of 2.0°C.

Earlier studies investigated how future temperature rises would affect annual electricity use or daily peak loads in specific cities or states. This new study is the first large-scale projection of household-level residential air conditioning demand. It drew on U.S. Energy Information Administration (EIA) data collected from 2005 to 2019 about household air conditioner use across the U.S., including observations and predictions of air temperature, heat, humidity, and discomfort indices.

10

Responding to Climate Change Does Not Dampen Winter Olympics Enthusiasm—Scenes from the Beijing Winter Olympics in the Snow

On the night of February 12, snow began to fall in the Beijing-Tianjin-Hebei region, affecting all three competition zones of the Beijing Winter Olympics. Forecasts were issued in advance, and the zones responded quickly to weather changes. On the field, athletes and coaches showed undiminished fighting spirit, also enjoying the unique joy of competing in the snow.

Meteorological services withstand the test of snowfall

At 4 a.m. on February 13, Shi Shaoying, deputy head of the meteorological service team in the Yanqing competition zone of the Beijing Winter Olympics, was already up. That whole day, she and her team provided continuous weather forecasts for alpine skiing events.

“The snowfall trend today was exactly as forecast—it turned lighter around 2 p.m.,” said Jenny Wiedeke, Director of Communications at the International Ski Federation, praising Shi’s team. “We were able to use this time to urgently clear the slopes, so although the competition was delayed for more than an hour, it did not need to be rescheduled.”

It is understood that in the core area of the mountain venues, the “Beijing RMAPS” numerical forecasting system—with 100-meter resolution and updates every 10 minutes—had already been put into practical use. On February 13, snow sports events most directly affected by weather conditions stood the test: all zones adjusted their schedules to varying degrees. The women’s downhill second official training in alpine skiing was canceled, but the men’s giant slalom, with a shorter course, was completed; in Zhangjiakou, cross-country skiing and Nordic combined events proceeded normally in the snow; the women’s slopestyle qualification in freestyle skiing was postponed due to poor visibility.

“Back on February 4, meteorological authorities had already forecast this round of snowfall, and two days ago had basically determined the timing and magnitude of snowfall at each venue, issuing early warning information,” said Yang Shuan, Vice President of the Beijing Winter Olympics Organizing Committee. He added that in transportation, catering, venues, and other areas, relevant departments, local governments, and mountain operations teams would work together to ensure athletes competed smoothly and stakeholders could carry out their work.
