# Supplementary material for: Public interest in biodiversity and climate change: A comparative culturomics study of China and the UK
Source: PLoS One. 2026 Jan 14;21(1):e0338006. doi: 10.1371/journal.pone.0338006 (PMC12803463; doi:10.1371/journal.pone.0338006)
Supplement: S3 Text — (DOCX) [file pone.0338006.s003.docx]

**News in the UK**

**Biodiversity temporal pattern news**

2011.1.1-2013.12.31

1

Ecuador's highland bugs key to biodiversity

Of all the things to worry about, it is not immediately obvious why anyone would choose some tiny bugs living in a stream some 4,500m (14,765ft) up in the Ecuadorean Andes.

But 36-year-old French river ecologist Olivier Dangles is passionate about them.

His hands shivering from the cold, he pokes at the writhing body of a tiny aquatic invertebrate he has just found in his sample. It will eventually become a fly.

"You could say some of these invertebrates are the polar bears of the high Andean streams," he says.

"There are at least five types here in danger of becoming extinct because of the disappearing glaciers which feed the streams where they are found."

Crowded out

It is hard to see how something you can only see properly under a microscope - and with complicated names such as Parochlus (Chironomidae, Diptera) - could rival the polar bear as a symbol of climate change.

But Professor Dangles and his fellow scientists at the French Development Research Institute and the Pontifical Catholic University of Ecuador are convinced that the rapidly retreating Andean glaciers offer an extra layer of stress not just to water supplies but also to the delicate mixture of fauna and flora in the region.

Since 2008, they have been collecting samples downstream from the majestic Antisana glacier, 40km (25 miles) south of the equator and an hour's drive from the capital, Quito.

Like many other glaciers in Ecuador, the part of the glacier feeding the streams has retreated around 200m since 1997.

Prof Dangles points to a nearby glacial stream which is milky white owing to small particles of silt carried down from the glacier.

2

Why bees & biodiversity benefit from indigenous wildflowers

Many of the wildflower areas that provide food for pollinating insects (such as honeybees and butterflies) have shrunk over the past few decades. So far, we have lost 97 per cent of lowland semi-natural grassland, 20 per cent of chalk grassland and thousands of miles of hedgerow. This is the effect of intensive agriculture and, in urban areas, an obsession with neatness. The result is that some wildflower species and insects have become extinct. ‘We need to fall in love with wild flowers, and those who have loved them in the past must rekindle their affections,’ says Sarah Raven in her beautiful new book Wild Flowers (£50, Bloomsbury). ‘It is not just about appreciating biodiversity and all that implies for a rich and healthy environment. It is also about us, and our connection to nature and the deepest possible delight that can be derived from feeling at home in a spectacularly flowery world.’

So what can you do to halt the decline in native fauna? For a start we can help by buying locally grown, organic food. This will attract insects and birds, and thus prevent the dreaded ‘Silent Spring' predicted by Rachel Carson in the 1960s. But we can also plant wild flowers in our own gardens: native blooms such as cornflowers will fill our outdoor spaces with the mellow hum of bees, the flutter of butterflies and bundles of colour.

3

Get a grip! Population growth impacts biodiversity

So for those who are still feeling a little let down, let me just add one additional reason for disappointment: how is it that the entire Rio+20 process came and went without any serious attention being given to one of the most important sustainability challenges today – namely, population?

I could of course have asked that question at any time over the last 40 years, pretty much since the time of the first UN Conference on Environment and Human Development in Stockholm in 1972 – when population was a big, controversial topic of debate.

So what exactly is going on? Why is it that the insights that we now have about the importance of population continue to go largely ignored – not least by the vast majority of environmental organisations whose ostensible purpose is to secure the foundations of the natural world to ensure good lives for people today and for people tomorrow? Forgive me if this is old hat, but let me just share with you a few of those insights.

Back in April this year, The Royal Society published its report People and the Planet. Over the preceding 18 months, I’d been lucky enough to be part of the working group that co-authored the report, which was based on the work of 22 brilliant scientists from all around the world, coordinated by a small group of Royal Society staff who had to process hundreds (and I do mean hundreds!) of detailed research reports. One of its key recommendations was this: “Population and the environment should not be considered as two separate issues. Demographic changes should be factored into international meetings such as the Rio+20 Conference.”

4

Oil deal 'threatens Ugandan biodiversity'

The discovery of oil in Uganda was a blessing to the impoverished East African country. But before the oil has even started pumping, disputes over tax, accusations of corruption and fears for the environment plague the sector. Alice Klein reports from Hoima

More than two billion barrels worth of crude oil are located under Lake Albert, which sits on the border of Uganda and Congo, and the surrounding forests and waterfalls. The World Bank has excitedly predicted 'black gold' could provide revenues of £1.3 billion per year during peak production thus lifting Uganda's population out of poverty, 40 percent of whom live on less than 80 pence per day.

Oil rights in western Uganda are owned by Anglo-Irish firm Tullow, which recently sold a third each to Total of France and the Chinese state oil company CNOOC in a deal worth £2.9 billion. Controversially, say critics, two rounds of exploration and extraction contracts between Tullow and the Ugandan Government - known as Production Sharing Agreements (PSAs) – have not been made public.

Campaign group Platform leaked the original PSAs in 2010 but the new agreements, hurriedly signed by President Museveni in February 2012, remain confidential. Platform has denounced the lack of environmental protection in the deals, saying the agreements fail to include penalties for pollution that are found in other countries such as fines per barrel of oil spilt, as seen in BP's £27 billion bill for the US Gulf Coast disaster.

But while lawyers wrangle over papers, it is the communities which fish Lake Albert and live along it's shoreline who are most concerned about potential oil spills, which they fear threaten their way of life. 'Tullow has a bad name here in Sebagoro,' says resident Harriet Namono, 28, who sells petroleum, tomatoes and fish on a rickety wooden table outside her hut.

5

Microplastics 'pose toxic threat to marine biodiversity'

Tiny particles of waste plastic that are ingested by shoreline "eco-engineer" worms may be negatively affecting biodiversity, a study says.

So-called microplastics may be able to transfer toxic pollutants and chemicals into the guts of lugworms, reducing the animals' functions.

An estimated 150 million tonnes vanishes from the global waste-stream each year.

The findings have been published in the academic journal Current Biology.

"We are losing a large volume of plastic and we know it is going into the environment and the assumption being made by policymakers is that this material is non-hazardous, it has got the same ranking as scraps of food," explained co-author Mark Browne, an ecologist from the US-based National Center for Ecological Analysis and Synthesis.

"The research we have done really challenges that," Dr Browne added, referring to the findings of lab work carried out by colleagues at Plymouth University, UK, led by co-author Prof Richard Thompson.

"Our findings show that the plastic itself can be a problem and can affect organisms.

"Also, when particles of plastic go into the environment what you find is that they accumulate large quantities of pollutants that are banned. So you have these particles themselves but also a load of nasty chemicals."

The team found that the tiny bits of plastic, which measure 1mm or smaller, transferred pollutants and additive chemicals - such as flame-retardants - into the guts of lugworms (Arenicola marina).

This process results in the chemical reaching the creatures' tissue, causing a range of biological effects such as thermal stress and the inability to consume as much sediment.

6

Reasons why India's biodiversity is at risk

Home to about a tenth of the world's known plant and animal species, India is a global biodiversity hot spot.

The country also has a bank of 50,000 varieties of rice, a grain that feeds about half of the world's population. Mangoes, tea, sorghum, millets and pulses grow freely on its lands.

But with 18% of the world's population crammed into just 2.4% of the global landmass, India's biodiversity is under pressure, something which the 12,000 delegates from 190 countries attending the UN meeting on biodiversity in the southern Indian city of Hyderabad will be aware about.

Here are some indicators of how India's biodiversity is under threat:

Four animals - the cheetah, Lesser Indian Rhinoceros, Pink-headed duck, and the Himalayan Mountain Quail - have become extinct in the last century.

A total of 929 animal species are threatened today, up from 648 in 2004, according to the International Union for Conservation of Nature (IUCN). India's rank in the global "shame list" of nations struggling to protect its species diversity has slid to seven, next to China.

Just 1% of India's 8,000km- (4,970-mile)-long coastline is protected from badly planned and illegal development. Coral reefs and fish are threatened by development projects like ports and power plants.

To be sure, India has explored and mapped the flora and fauna of only around 70% of its area, documenting some 150,000 plants and animals. Even the government admits there is a "wide gap" in mapping all species.

So the jury is still out on the total number of new and dying species. But in a country which spends about $2bn (£1.25bn) on biodiversity, there are some good tidings as well:

7

No substitute' for virgin forest

The crucial role that virgin forests play in conserving nature is confirmed in a study that spans the tropics.

An international team of researchers analysed more than 100 existing studies comparing wildlife in forests that had been modified and those that had not.

Nature, notably birds, does much better in virgin tracts, they report.

The researchers conclude in the journal Nature: "When it comes to maintaining tropical biodiversity, there is no substitute for primary forests".

The study feeds into one of the major debates going on in environmental circles: whether it is better to exploit lots of land relatively gently, or to develop intensively in some areas and leave others as wild as possible.

"Primary forests are truly unique and have exceptional value for biodiversity," said study co-leader Luke Gibson from the National University of Singapore.

"So if you can minimise the destruction of primary forests, then that might be the best strategy for tropical biodiversity.

"And if you have to use agricultural intensification of areas that are already used for agricultural production instead of focusing more on other forms of agriculture that attempt to maintain some levels of biodiversity, such as agrofrestry, that strategy might be more effective for maintaining the highest levels of biodiversity overall," he told BBC News.

'Marked' impact

The researchers reviewed 138 studies that included 2,230 examples where biodiversity had been compared between tracts of virgin forest and areas where something had changed.

Those changes ranged in severity from complete clearance for agriculture, through plantations and agroforestry, to selectively logged forests where only certain types of tree had been extracted.

In all but the selectively logged areas, the impact on biodiversity was marked.

8

New research highlights influence of intraspecific variability on biodiversity studies

A study of around 100 newly collected specimens of early ammonoids (marine invertebrates with distinctive coiled shells) suggests that the number of species they belong to might have been over-estimated due to the large variability in size and shape within each species.

Recognizing this intraspecific variability is important for biodiversity studies as failing to do so might artificially inflate the number of species known from a certain time or place.

Like dogs and various other organisms, ammonoids – a group of externally shelled cephalopods related to Nautilus, squids and octopods – show extremely large ranges in intraspecific variability in size and shape.

They have often been used to study changes in biodiversity, biogeography and extinction events in Earth history due to their high taxonomic and ecological diversity, wide geographic distribution, high evolutionary rates and preservable shell, as well as their successful evolutionary history on the Earth for over 300 million years. They survived multiple mass extinction events, only to go extinct together with the dinosaurs at the end of the Cretaceous.

Dr Kenneth De Baets of the University of Bristol, with Dr Christian Klug (University of Zürich) and Dr Claude Monnet (University of Lille), studied the intraspecific variability through ontogeny (development of an organism) in early ammonoids, which has rarely been attempted before. Ammonoids are ideal for this type of study as they hold a record of growth from embryo to adult in their accretionary shell.

Dr De Baets said: “It took more than three years to collect and prepare around 100 Devonian ammonoid specimens from the same geological layer in the same region of Morocco.”

9

Environmental damage and human rights abuses blight global tea sector

Human rights violations have been reported at plantations in virtually all major tea producing countries, while tea growing itself has a profound effect on the local environment. William McLennan reports

Tea is grown in more than 45 countries worldwide, and while India and China are the biggest producers, Sri Lanka and Kenya lead the way in exporting tea to meet the global demand. Two main issues are associated with tea production: the vast amount of land required to grow it, and the intensive labour needed to harvest it. Human rights violations have been reported at plantations in virtually all major tea producing countries, while the plantations themselves have a profound effect on the local environment.

Labour conditions

Tea production, in particular the labour required for harvesting the leaves by hand, has long been associated with the poor treatment of workers. In a labour-intensive industry such as tea production, reducing the cost of labour will increase profit margins and often leads to the inhumane treatment of workers.

Plantation labourers are poorly paid. In Sri Lanka poverty levels on plantations exceed the national average, with 30 per cent living below the poverty line despite being employed. In India, where workers are expected to pick more than 20 kilograms per day, wages are as little as $1-1.5 per day. Plantation work does not give labourers sufficient wages to pull themselves out of poverty, providing the multibillion dollar tea industry with a ready supply of cheap labour.

10

Biodiversity is the key to 1.5MW Knapp solar park

Higher Knapp Farm in the small Somerset village of Knapp, Somerset will soon be producing enough carbon-free electricity to run 442 local households, thanks to the approval of plans to develop a 1.5MW solar farm.

Solar developer, Lightsource Renewable Energy, entered into an agreement with the land-owner to operate a solar park for 25 years. In order to gain planning permission for the proposals, Lightsource had to consult closely with Taunton Deane Borough Council to address locals’ concerns.

First and foremost, local residents raised issues over the visibility of the proposed solar site. As the site slopes upwards at the eastern border, residents were concerned that the solar park would be eminently visible. Responding to the concerns, Lightsource went back to the drawing board and redesigned the site to a location in the lower areas of the land. In addition, the company proposed planting a screen of hazel coppice trees to shield sightlines.

Conor McGuigan, Business Development Director for Lightsource, explained: “When we develop a new solar farm, we are entering into an agreement with the landowner and council for a period of 25 years. Therefore we are required to demonstrate that the land we build on is protected and used correctly to its fullest potential in order to obtain planning permission”.

“We are strong advocates of optimising the opportunity that a solar farm presents to enhance site biodiversity. Higher Knapp Farm will not only benefit from a new hazel coppice, but a species rich wildflower mix will be planted beneath and between the solar panel rows, creating a more diverse habitat.”

2014.1.1-2016.12.31

1

Study shows urbanisation's impact on biodiversity

A dataset, described as the largest of it kind to date, has assessed the impact of urbanisation on biodiversity levels around the globe.

It found that cities supported far fewer species of birds and plants compared with similar areas of undeveloped land.

However, it showed the vast majority of flora and fauna in a city reflected an area's "unique biotic heritage".

"While we show that urbanisation has caused cities to lose large numbers of plants and animals, the good news is that cities still retain endemic native species, which opens the door for new policies on regional and global biodiversity conservation," said co-author Myla Aronson, a research scientist from Rutgers University, in the US state of New Jersey.

"In particular, the study highlights the value of green spaces in cities, which have become important refuges for native species and migrating wildlife."

The international team of researchers collected lists of plants in 110 cities and lists of birds found in 54 cities around the world.

"We were interested in plants and birds that were found in all parts of the city - from buildings, roads, vacant lots, brownfields, managed greenspaces and natural greenspaces - to understand how cities support biodiversity as a whole," Dr Aronson told BBC News.

Unique biotic heritage

The team analysed the data and found that cities retained about just 8% of bird species and 25% of plant species of comparable undeveloped land.

But Dr Aronson added: "Contrary to popular belief, we show that the plants and birds of cities are not all the same across the world.

2

Organic farming 'benefits biodiversity

Organic farms act as a refuge for wild plants, offsetting the loss of biodiversity on conventional farms, a study suggests.

Fields around organic farms have more types of wild plants, providing benefits for wildlife, say scientists.

The research is likely to fuel the debate over the environmental benefits of organic farming.

Studies suggest that organic farming produces lower yields than conventional methods but harbours more wildlife.

The new study, by researchers at the University of Swansea and institutes in France, looked at fields sowed with winter wheat in the region of Poitou-Charente.

They found that organic farming led to higher weed diversity on surrounding conventionally farmed fields.

"Wild plants are important for birds, bees and other farmland species," said Dr Luca Borger of the department of biosciences at Swansea University.

"Organic farming has advantages in maintaining these, but even a mixture of organic and non-organic farming in an area can help maintain this biodiversity.

Food security

Farmland provides essential habitat for many animals but intensification of agriculture has led to a loss of biodiversity.

However, in order to provide the extra food needed by the bigger human population of the future, without destroying forests and wetlands, farming needs to be made more intensive.

Supporters of organic farming say the method could be a potential compromise between meeting food security needs and providing habitat for bees, birds and other wildlife.

The researchers say land-sharing between organic farms and non-organic farms could have benefits for both crop production and biodiversity.

3

Scientists warn of 'unsafe' decline in biodiversity

An international team of scientists has issued a warning that biodiversity is dropping below safe levels for the support and wellbeing of human societies.

As a species we are inextricably connected with the processes of our local ecosystems, such as crop pollination, waste decomposition and regulation of the carbon cycle.

These ecosystems depend on the biological diversity within them to function.

The planetary boundaries framework updated in 2015 states that losing more than 10% of the biodiversity in an area places the local ecosystem at risk.

A report in Science this week states that 58% of the world's land coverage already falls below this safe level. They find that the global average of biodiversity has dropped to 85% of that of unaffected ecosystems.

Ascertaining the level of biodiversity loss that an ecosystem can endure is not straightforward and will be dependent upon individual ecosystems. Therefore a definitive level of 10% loss must be taken with caution.

Co-author Prof Andy Purvis, from Imperial College London and the Natural History Museum, explained: "Once we're the wrong side of the boundary it doesn't mean everything goes wrong immediately, but there is a markedly higher risk that things will go badly wrong."

Trying to further refine the "safe" value of 10% also produces risk, by increasing the value and allowing even further degradation of ecosystems irreversible damage could occur.

Adding credibility to the report, Owen Lewis, professor of ecology at Oxford University, who was independent of the research, told the BBC: "This is definitely a situation where the precautionary principle needs to be applied: we can't afford to wait to see the long-term consequences of degradation of natural ecosystems."

4

THE A-Z OF BIODIVERSITY

UNEP-WCMC would like to announce the launch of the updated Biodiversity A-Z website. The site provides concise and authoritative information about biodiversity in an accessible and simple manner.

Content is presented in the form of articles, factsheets and definitions. The new website structure divides the information up into easy to browse biodiversity-related themes, including two new themes - Marine and Acronyms - and has a new and powerful search.

As with any specialist field, conservation can contain complex concepts and confusing terminology. The Biodiversity A-Z is a reliable resource that helps to clarify biodiversity-related topics. It can be used by specialists and non-specialists alike, and provides both scientific information and details on the business and social relevance of terms. Information is presented in a concise manner, and is compiled by experts. This update includes a new website design with thematic groupings and user-friendly pages that make this tool quick and easy to use.

The new Biodiversity A-Z structure includes four themes. The ‘Areas’ theme contains updated factsheets on Areas of Biodiversity Importance and provides detailed explanations of the different types of area classifications, including their levels of protection and their significance for biodiversity. The ‘Terms’ theme makes accurate definitions of biodiversity-related terminology accessible under a unified platform. The newly-added ‘Marine’ theme provides useful factsheets on marine-specific features while the new ‘Acronyms’ theme includes over 250 commonly-used acronyms in the field of conservation.

5

Bat-sound library tracks biodiversity

Scientists have compiled the biggest known library of bat sounds in an effort to identify and conserve rare species.

Mexico is home to many of the world's bats, but it also has one of the highest rates of extinction.

International researchers recorded more than 4,500 calls from about half of Mexico's 130 bat species.

The audio library allows bat calls to be identified automatically, helping to monitor any changes in biodiversity.

Co-researcher, Prof Kate Jones, of UCL and Zoological Society London, said the ability to map bats was crucial for implementing effective conservation measures.

"We've shown it is possible to reliably and rapidly identify bats in mega-diverse areas, such as Mexico, and we hope this encourages uptake of this method to monitor biodiversity changes in other biodiversity hotspot areas such as South America," she said.

Bat detectives

For the study, scientists went into the desert, jungle and tropical forests of Mexico to record bat calls, using remote monitoring stations.

They built up an audio library that included all families of Mexican bats and about half the number of species.

Machine learning algorithms and voice-recognition software was developed, which was able to identify about 70% of bat species from their call.

Bats are usually hard to identify from their sounds because they have very similar calls, which are often too high in frequency to be detected by the human ear.

Lead researcher Dr Veronica Zamora-Gutierrez, from UCL and the University of Cambridge, said audio surveys were increasingly being used to monitor changes in biodiversity, and bats were especially useful for this.

6

Plant-killing fungi 'drive rainforest biodiversity'

Pathogenic fungi, normally associated with killing plant life, could play a key role in driving biodiversity in tropical rainforests, a study suggests.

Researchers found that the presence of fungal pathogens limited the growth of dominant species, allowing other plants to become established.

The scientists said the study offered an insight into why rainforests are biodiversity hotspots.

"The conventional wisdom in ecology suggests that if you have got more than one species competing for the same set of resources, one of those species should win and exclude the other," co-author Owen Lewis from the University of Oxford explained.

However, an idea developed by ecologists Daniel Janzen and Joseph Connell in the 1970s linked pests and diseases to high levels of biodiversity in tropical areas, which they described as negative density dependence.

"The way the hypothesis works is to give rare species an advantage - the idea is that pests and diseases tend to transmit more effectively if plants are growing close together," Dr Lewis told BBC News.

"This acts as a negative feedback mechanism, so if one species becomes too abundant locally then it tends to get hammered by the pests and diseases and this then gives rarer species a chance because they tend to be less affected.

"This acts as a balancing mechanism that prevents these rarer, and perhaps competitively inferior species, from being outcompeted."

"This is the first study to explicitly link a particular group of natural enemies to negative density dependence and the maintenance of species diversity in tropical forest plants," she wrote in a commentary for Nature journal.

7

Biodiversity loss breaching safe limits worldwide

The loss of species diversity has reached unsafe levels across 58% of the world's land surface, according to a new assessment led by Museum scientists.

The research, published in the journal Science, found that human land use has driven down the population of many species to a dangerous extent across vast swathes of the planet.

Worryingly, biodiversity 'hotspots' - areas that are the only home for large numbers of species, and with high levels of habitat loss - are among the regions badly affected. Twenty-two of the 34 hotspots suffer from unsafe levels of biodiversity loss. Other areas that have suffered big losses include grasslands and savannahs.

'Across most of the world, biodiversity loss is no longer within the safe limit suggested by ecologists,' says the paper's lead author, Dr Tim Newbold of University College London and the UN Environment Programme World Conservation Monitoring Centre.

As a result, he adds, 'we are approaching a situation where human intervention may be needed to sustain the function of these ecosystems.'

Ecological roulette

The assessment, conducted by Natural History Museum researchers and colleagues from across the UK, Europe and Australia, is one of the first to estimate biodiversity loss from ecological communities at a global scale.

To do this, rather than look at the numbers of species already lost to extinction, the team assessed the effect that habitat loss has had on populations of surviving species. This is an important measure of how well local ecosystems can continue to provide the goods and services on which people depend. It also tells researchers what scope remains for conservation.

8

Bullfighting is conserving Spain's biodiversity - ban at nature's peril

Bullfighting may cause suffering to animals, but that does not mean the EU should ban it or withdraw farm subsidies, writes Robin Irvine. Traditional bull-breeding estates are valuable reservoirs of biodiversity in intensively farmed landscapes, and without the bulls there would be nothing to sustain them.

The bulls' psychological and physical well-being is part of what determines whether they perform to their potential. This encourages breeders to raise them as 'naturally' as possible: in herds, with varied grazing, space, shade, dust baths, water and hidden spots to which they can retreat.

These formidable creatures are incredibly sensitive to change. To ensure proper care and minimise disruptions, the foreman works with a team of cowhands, working horses, the estate owner/manager, secretaries, grounds staff, vets, ethologists and even nutritionists.

As with any industry, standards can vary. I cannot speak for all bull breeders, but I certainly saw how seriously people took correct care and a modern approach in Andalusia. The world of the bulls is often labelled 'traditional', but breeders don't oppose modernity. These 'barbarians' have their own vision of the future, which actually complements the CAP in some respects.

Aside from food production - and let's not forget fighting bulls are high-quality beef animals - CAP subsidies are intended to support the sustainable management of natural resources and rural economies. Partido de Resina is an island of biodiversity: around 500 hectares of open woods and marshland surrounded by a sea of monotonous orange, olive and peach plantations.

9

World's poor most affected by biodiversity and ecosystem loss

Europe's biodiversity for life programme contributes to socioeconomic development and the eradication of poverty, writes Roberto Ridolfi

more than 70 per cent of the world's poor live in rural areas and depend directly on biodiversity and ecosystems for their subsistence.

Ecosystem services provide livelihoods, enhance food and nutrition security, enable access to water and to health and contribute significantly to climate change mitigation and adaptation.

For example, mangrove ecosystems, when sustainably managed, allow the maintenance of fish stocks at a high level, while at the same time protecting the coast from erosion, providing timber and fuel wood, and storing carbon in the roots and the soil.

An increasing population and global trade have put unsustainable pressure on renewable natural resources, such as bush meat, fuel wood or arable land, which is increasing long-term poverty and leads to biodiversity loss.

On the other hand, illegal wildlife trade of endangered species has a major impact on biodiversity, but also represents a real threat to national security and economic development for many African countries.

Unprecedented poaching levels and sophisticated smuggling capabilities are indicative of organised criminal activity.

Over €21bn of worldwide environmental crime is attributed to illegal wildlife trade, of which ivory is an important component.

It is believed that this money is partly financing illegal groups, such as the Lord's Resistance Army, Boko Haram and Al-Shabaab, playing a part in destabilising the security of large regions.

Since 1985, EU funding for biodiversity protection in sub-Saharan Africa went to direct support of protected areas management, with a special focus on Central and West Africa - for example, Virunga in Democratic Republic of Congo and Odzala in Republic of Congo.

10

PREDICTING THE IMPACT OF LAND-USE CHANGE ON BIODIVERSITY

Species that live within a narrow range and have specialist habitat requirements are disproportionally affected by the conversion of land for human-dominated use. Using the PREDICTS model, scientists at UNEP-WCMC and the Natural History Museum, London are able to predict the consequences of land-use change on biodiversity.

As the human population continues to increase, demand for more agricultural land is one of the main drivers of habitat loss and degradation. This change in land use presents the greatest immediate threat to biodiversity and could lead to changes in the way our ecosystems function as well as species extinctions.

PREDICTS – Projecting Responses of Ecological Diversity in Changing Terrestrial Systems – is a collaborative project that combines data from many different studies to identify how a range of human pressures impact on species and their habitats. In this latest study, biodiversity change under different scenarios of land-use change was predicted for nearly 4,000 topical and sub-tropical forest habitat dwelling taxa including invertebrates, reptiles, amphibians, mammals, and birds.

Forest-specialist species and species with narrow ranges are disproportionately lost from ecological communities when land is converted from natural to human-dominated habitats. In particular, birds are highly sensitive to urban land use and the number of bird species declines as human population density increases.

A number of species whose risk of extinction has not yet been assessed by The IUCN Red List of Threatened Species were identified by the model as being most sensitive to land-use change. This shows that the PREDICTS model could be used to make an early estimate of the impact of habitat loss on species for which there is little information. The model may also identify species that are not yet listed as threatened but could be with further land-use change.

2017.1.1-2019.12.31

1

The world is in trouble: one million animals and plants face extinction

A landmark report has confirmed that humanity is destroying its own life support system as the natural world faces unprecedented declines.

An international team of scientists, backed by the UN, has reported that communities around the world are likely to face dire consequences as ecosystems decline faster and faster.

Human impacts on the natural environment are now so great that we are eroding our own economies and food security, according to the world's leading climate scientists.

Every ecosystem around the world is affected by extinction, from coral reefs to tropical jungles, and the problem is accelerating with each passing day.

It is estimated that around one million animals and plants are threatened with extinction - more than ever before in human history. More than 40% of amphibian species, about 33% of reef-forming corals and more than a third of all marine mammals are threatened.

And it is humanity that is to blame, as about 75% of environments on land have been significantly altered by human actions, plus roughly 66% of the marine environment.

Compiled by 145 expert authors from 50 countries, with inputs from another 310 contributing authors, the report was released by the Intergovernmental Science-Policy Platform on Biodiversity and Ecosystem Services (IPBES).

It is the most comprehensive planetary health-check of its kind, having examined changes to the natural world over the past five decades.

Experts studied the relationship between economic development and human impact on nature. They also offered a range of possible scenarios for the coming decades and found that action is needed urgently if we are to protect both people and the planet from catastrophic damage.

2

A shocking new report reveals what we've done to the natural world

As a result of human activity, one million animal and plant species could disappear in the next few decades - the most that have ever been at risk in human history.

Only a large-scale reimagining of the world’s economic and financial systems can limit the damage done by humans, according to a new report released by the Intergovernmental Science-Policy Platform on Biodiversity and Ecosystem Services (IPBES).

Human activity has "significantly altered" 75% of our planet's land and 66% of the ocean. As the human population has increased, more than a third of the land surface and 75% of freshwater resources is now used to grow food. Urban areas have doubled in size since 1992.

Since 1980, plastic pollution has grown by tenfold and humans now pump 300-400 million tonnes of heavy metals, solvents, toxic sludge and other wastes from industrial facilities into our ocean and waterways every year.

The sheer amount of waste we dump in the water has created 400 dead zones in the ocean, areas with so little oxygen almost no life survives.

Intensive industrial agriculture and over-fishing are particular culprits in the natural world’s decline.

Dominic Waughray, Head of the Centre for Global Public Goods at the World Economic Forum, said the report was a wake-up call for governments and businesses.

"The science is clear that we are in the midst of a sixth mass extinction and we cannot continue with business as usual. The interconnections between the global food system, ecosystems and natural resources, climate change, and people’s health and livelihoods are deeply rooted."

The solution, Waughray said, is a wave of innovation across industries, especially within global supply chains.

3

The future of food under 'severe threat' as species diversity disappears

The future of food supplies is under "severe threat" because of the number of animal and plant species fast disappearing, a United Nations report said on Friday, as the world grapples with how to feed a growing population.

People are depending on fewer species for food, said the U.N.'s Food and Agriculture Organization (FAO), leaving production systems susceptible to shocks like pests or disease, droughts and other extreme weather events due to climate change.

Although about 6,000 plant species can be used for food, less than 200 varieties are widely eaten, and only nine make up most of the world's total crop production, the FAO said in the first report of its kind to assess biodiversity in food systems.

"The loss of biodiversity for food and agriculture is seriously undermining our ability to feed and nourish an ever-growing global population," said head of the FAO, Jose Graziano da Silva, in a statement.

"We need to use biodiversity in a sustainable way, so that we can better respond to rising climate change challenges and produce food in a way that doesn't harm our environment," he said.

By analysing data from 91 countries, the FAO said there was "mounting evidence" the world's biodiversity was under "severe threat" due to pollution, badly managed water and land use, poor policies, over harvesting and climate change.

Climate change will become a steadily bigger threat to biodiversity by 2050, adding to damage from pollution and forest clearance to make way for crops, according to more than 550 experts in reports approved by 129 governments last March.

4

Government sets out vision for a safe and biodiverse railway lineside

The Department for Transport (DfT) has set out a clear strategy for how it expects Network Rail to protect and enhance the UK’s lineside environment, while ensuring the safety of passengers and services.

The strategy follows recommendations from the independently chaired review led by John Varley, a non-executive director of the Environment Agency. First commissioned by the department in May 2018 to look at Network Rail’s lineside vegetation management, Valuing nature – a railway for people and wildlife, has outlined a number of proposals to protect and increase biodiversity.

The new policy has been drawn up in collaboration with the Department for Environment Food and Rural Affairs’ 25 Year Environment Plan. This ambitious document challenges Network Rail to achieve no net loss in biodiversity across the network by 2024, deliver a net gain in biodiversity by 2040 and produce a vision statement and biodiversity action plan by December 2019 detailing how it will meet these goals.

These targets build on the good practice Network Rail already deploy across the network to ensure vegetation does not delay journeys.

I’m pleased that we have agreed an ambitious strategy for enhancing the important vegetation and wildlife alongside our railways.

As we strive for a cleaner, greener railway, we must also work hard to enrich our unique biodiversity. The good work Network Rail is already doing ensures that we strike a balance between effective management, protecting passengers and reducing delays.

We must now go further and ensure we leave a legacy of a greener, vibrant and more diverse railway.

5

Supporting biodiversity in UK Overseas Territories

A 10 week Call for Evidence has been launched by the UK Government today (Friday, 10 May) to gather knowledge on safeguarding biodiversity in the UK Overseas Territories

Many of the Overseas Territories are globally significant in their ecological richness, containing over 90% of the UK’s marine and terrestrial nature. They are hotspots for biodiversity with an estimated 3,300 endemic species, but they are also highly vulnerable to environmental change.

To support on-going work in the UK Overseas Territories to tackle climate change and protect the natural environment, the Call for Evidence will help to develop and refine existing and potentially new funding streams from the UK Government to replace EU finance sources.

Today’s announcement builds on the 25 Year Environment Plan which contains a number of ambitions to improve biodiversity in the Overseas Territories, including the continued implementation of the Blue Belt programme.

The recent IPBES report drives home the message that we must increase our efforts to protect our environment as the effects of biodiversity decline and climate change are felt around the world.

“This is why it’s crucial to ensure that precious species and landscapes in our Overseas Territories continue to be supported. This Call for Evidence will provide us with the evidence to help determine the best way to do so.

“2020 is the year we must all agree on further action for climate, nature and ocean conservation, with appropriate funding to protect and enhance the planet’s most pristine ecosystems for flora and fauna.”

6

State of Nature: Britain’s biodiversity is under threat but it can yet be saved

The authoritative State of Nature 2019 report highlights a sharp decline in wildlife, plants and fungi caused by a variety of factors that range from climate change to urbanisation. But, with ambitious measures, the tide can yet be turned.

Britain’s biodiversity is dying out. Almost half of the birds, half of the fungi and a quarter of the country’s mammals are at risk of extinction and, looking specifically at wildlife, both population numbers and their distribution across the UK have been declining.

The stark warning comes from the latest State of Nature report, released on October 4 by a group of more than 70 conservation charities, research institutions and government bodies.

Biodiversity is declining — fast

Based on a rigorous statistical analysis of data collected by tens of thousands of volunteers, the study, which is published every three years, looks at changes in British wildlife over the medium and long term (10 and 50 years respectively).

The picture that emerges from the report is dire. Since 1970, which is the study’s baseline, about 41% of almost 700 bird, mammal, butterfly and moth species have seen a drop in numbers, against 26% that have seen a rise. And of more than 8,400 species monitored under the International Union for the Conservation of Nature Red List, one in seven is at risk of extinction, with 133 having already vanished.

But it’s not just animals that are disappearing fast. ‘We know one in five British wild flowers is under threat and continued declines must be urgently addressed if we are to better protect the wealth of wonderful wildlife plants underpin,’ points out Jenny Hawley of Plantlife. ‘Where wild flowers lead, wildlife follows: the marsh fritillary butterfly feeds almost exclusively on devil’s-bit scabious, so it lives or dies according to the prospects of its food plant.’

7

WWF report: Mass wildlife loss caused by human consumption

"Exploding human consumption" has caused a massive drop in global wildlife populations in recent decades, the WWF conservation group says.

In a report, the charity says losses in vertebrate species - mammals, fish, birds, amphibians and reptiles - averaged 60% between 1970 and 2014.

"Earth is losing biodiversity at a rate seen only during mass extinctions," the WWF's Living Planet Report adds.

It urges policy makers to set new targets for sustainable development.

The Living Planet Report, published every two years, aims to assess the state of the world's wildlife.

The 2018 edition says only a quarter of the world's land area is now free from the impact of human activity and the proportion will have fallen to just a 10th by 2050.

The change is being driven by ever-rising food production and increased demand for energy, land and water.

Although forest loss has been slowed by reforestation in some regions in recent decades, the loss has "accelerated in tropical forests that contain some of the highest levels of biodiversity on Earth", the report notes.

It says South and Central America suffered the most dramatic decline in vertebrate populations - an 89% loss in vertebrate populations compared with 1970.

Marine freshwater species are particularly at risk, the report says. Plastic pollution has been detected in the deepest parts of the word's oceans, including the bottom of the Mariana Trench in the Pacific.

Freshwater species - living in lakes, rivers and wetlands - have seen an 83% decline in numbers since since 1970, according to the report.

8

How to benefit species and habitats biodiversity in your woodland

Find out about priority habitats and species, the value of ancient and native woodland and when it's appropriate to remove trees to restore open habitats.

The conservation of biodiversity is an essential part of sustainable forest management. Forests cover nearly one-third of the world’s total land area and are vital in ensuring environmental functions such as climate regulation and soil conservation in addition to biodiversity. They provide habitats for a large array of plants and animals, some of which are rare or threatened. Through these important ecosystem services, biologically diverse forests and woodlands contribute to the sustainability of the wider landscape.

Support priority habitats and priority species

Many habitats that are important for biodiversity in the UK have been reduced in area and fragmented and, while they are generally protected, are in need of restoration and expansion. Priority habitats have the potential to provide the richest and most varied components of biological diversity within the UK. All types of native woodlands, as well as wood pasture and parkland, are woodland priority habitats.

Priority species are those that are declining, rare, at risk of extinction, and/or have special requirements. A high proportion of priority species are associated with semi-natural woodland.

Conserve ancient and native woodland

Woods that are both ancient and semi-natural in character have the greatest value for biodiversity. Known as ancient semi-natural woodland (ASNW) these are still widespread although fragmented. They serve as valuable refuges of woodland biodiversity, particularly for sedentary species that, once lost, do not readily recolonise. ASNWs also frequently retain characteristics of previous management such as coppice and other traces of cultural history.

9

NEW GLOBAL FOREST WATCH BIODIVERSITY MAPS HELP PRIORITISE AREAS FOR CONSERVATION

Two new global datasets, produced by UNEP-WCMC, will help identify areas of forest that are important for biodiversity.

Forests are incredibly biodiverse ecosystems, home to an estimated 80% of the world’s terrestrial biodiversity. But forests are rapidly being destroyed and degraded, posing a serious threat to biodiversity. As such, information about which areas of forest are most important for biodiversity, and how threatened they are, is crucial for prioritising conservation actions.

In collaboration with IUCN, Birdlife, the Natural History Museum and the World Resources Institute, UNEP-WCMC has produced two new data layers - of biodiversity importance and biodiversity intactness - for the Global Forest Watch website.

The biodiversity importance layer provides a visual representative of how important each pixel on the map is for forest species. Using species range data from the IUCN Red List, each pixel is weighted on the number of overlapping species ranges and the proportion of the global range the pixel represents. This means areas of forest that are either home to many common species, or a few globally rare species, would get a high rating.

The biodiversity intactness layer was produced using the PREDICTS database, and showcases how close the current ecological community is to its natural state, prior to any human disturbance. It shows how many individuals of a species that would be found in a pristine habitat are remaining.

By showing which areas of forest are important for biodiversity, and where they are under threat, these new data layers will allow scientists to identify and prioritise areas for conservation action.

10

BIODIVERSITY INDICATORS PARTNERSHIP LAUNCHES A NEW PLATFORM FOR VISUALISING BIODIVERSITY INDICATORS

The Biodiversity Indicators Partnership (BIP), together with NatureServe and UNEP-WCMC, have launched a visual, online platform to aid governments to make evidence-based decisions regarding biodiversity. The BIP Dashboard brings together the strength of the indicators brought together under the BIP, of which UNEP-WCMC is Secretariat, with NatureServe’s strengths in information technology and data visualisation to measure and visualise progress towards global, regional and national targets.

The new BIP Dashboard is an interactive, user-designed tool that brings together visual representations of such vital datasets as the Red List Index, the Biodiversity Habitat Index, and the Ecological Footprint. Indicators brought together under the BIP play a key role in measuring progress to achieving the Aichi Biodiversity Targets of the Strategic Plan for Biodiversity 2011-2020 as well as supporting other intergovernmental process such as the Intergovernmental Science-Policy Platform on Biodiversity and Ecosystem Services and the Sustainable Development Goals.

The tool is also structured to provide country-level dashboards, regional comparisons and exploration of indicators via an interactive map for any part of the world, allowing users to download visualisations of indicator trends at multiple spatial scales and use them for national reporting.

Anna Chenery, Coordinator of the BIP Secretariat, commented: “While the BIP has always been an authoritative collective of indicators and biodiversity data, we wanted to increase its functionality to make it as user-friendly as possible. The new interactive, visual representations such as scalable maps and downloadable graphs of time series data provide another avenue through which to interpret indicator trends.”

2020.1.1-2022.11.1

1

PM commits to protect 30% of UK land in boost for biodiversity

The Prime Minister is committing today (Monday 28 September) to protect 30% of the UK’s land by 2030.

Existing National Parks, Areas of Outstanding Natural Beauty and other protected areas already comprise approximately 26% of land in England. An additional 4% – over 400,000 hectares, the size of the Lake District and South Downs national parks combined – will be protected to support the recovery of nature.

The government will work with the Devolved Administrations to agree an approach across the UK, and with landowners and civil society to explore how best to increase the size and value of our protected land.

The announcement comes as the Prime Minister is set to sign the Leaders Pledge for Nature at a virtual United Nations event later today, committing to put nature and biodiversity on a road to recovery by 2030.

Boris Johnson will warn that countries must act now to reverse devastating biodiversity loss and prevent more species from being lost forever, with a 68% decline in global wildlife populations since 1970 alone.

We must turn these words into action and use them to build momentum, to agree ambitious goals and binding targets.

We must act now – right now. We cannot afford dither and delay because biodiversity loss is happening today and it is happening at a frightening rate. Left unchecked, the consequences will be catastrophic for us all.

Extinction is forever – so our action must be immediate.

The Leaders Pledge for Nature commits world leaders to take ten urgent actions, including on sustainable food production, ending the illegal wildlife trade and implementing nature-based solutions for climate change.

2

Britain faces biodiversity collapse

The UK has an average of only 53 percent of its biodiversity left, according to the Natural History Museum.

The UK may not have enough biodiversity to prevent an ecological meltdown and is already one of the world’s most nature-depleted countries – according to new data.

The UK has an average of only 53 percent of its biodiversity left, well below the global average of 75 percent, according to analysis by the Natural History Museum released on Sunday.

Both figures are lower than the 90 percent average, which experts consider the “safe limit” to prevent the world from tipping into an “ecological recession”.

Variety

This in turn would result in a future in which ecosystems do not have enough biodiversity to function well, leading to crop failures and infestations that could cause shortages in food, energy and materials.

Biodiversity represents the variety of plant and animal life on Earth, and scientists say it is dwindling fast.

“Much of the world has lost a large amount of its natural biodiversity,” said Dr Adriana De Palma from the Natural History Museum.

“Those systems have lost enough biodiversity to mean that we have to be careful about relying on them functioning in the way that we need them to.”

Researchers at the museum have developed the Biodiversity Intactness Index (BII), which measures the percentage of nature that remains in an area.

Species

The UK’s 53 percent BII places it in the bottom 10 percent of the world’s countries and last among the G7 group of nations.

The UK’s long-time low position in the league table is linked to the industrial revolution, said Professor Andy

3

UK takes lead to seek global action on nature at COP15 biodiversity conference

The UK will lead ambitious calls to protect nature at a UN meeting to agree global biodiversity targets in Nairobi this week - in line with UK’s domestic leadership through its Environment Act.

The UK-led statement, supported by 46 other high ambition countries, calls on the international community to halt and reverse biodiversity loss globally and adopt the ‘30by30’ target to protect at least 30 per cent of land and ocean by 2030. This will help restore ecosystems, drive species population recovery and halt extinctions by 2050.

The preliminary discussions in Nairobi, ahead of the rescheduled UN Convention on Biological Diversity COP15 conference, will help shape a Global Biodiversity Framework (GBF) to drive international action over this decade. This will build on momentum following the UK’s COP26 presidency, which led to the Glasgow Leaders’ Declaration on Forests and Land Use being agreed, a commitment by 140 leaders representing 90 per cent of the world’s forest to halt and reverse forest loss and land degradation by 2030.

Biodiversity loss cannot be ignored – deforestation, pollution and loss of nature is happening at an alarming rate. Following a two-year hiatus due to the pandemic, the UK also welcomes confirmation that - under China’s presidency - the COP15 conference will go ahead later this year in Canada.

Nature is in crisis. We need an ambitious set of targets to halt and reverse biodiversity loss globally by 2030, and I am delighted to hear the news COP15 will go ahead later this year. Nature recovery is one of the greatest challenges we face as a global community. We must reverse deforestation, protect our landscapes and seas and give them and the species they hold a chance to recover.

4

COP15 explained: What is the biodiversity conference and why is it important?

The 15th Conference of the Parties to the United Nations Convention on Biological Diversity, or COP15, is the latest meeting in which the nations of the world will come together to discuss the world's biodiversity.

These discussions cover all forms of life from microscopic viruses to enormous whales, as well as conservation, knowledge-sharing and financial policies associated with these organisms, their protection, and their use.

The first meeting took place in 1994 in the Bahamas, and COP15 is the latest. Due to delays associated with the COVID-19 pandemic, it has been split into two halves. The first part took place virtually in 2021, while the concluding part is set to take place later this year.

This was also originally due to take place in Kunming, China, but amid ongoing uncertainty due to COVID-19, this meeting has been moved to Montreal, Canada.

The 15th Conference of the Parties to the United Nations Convention on Biological Diversity, or COP15, is the latest meeting in which the nations of the world will come together to discuss the world's biodiversity.

These discussions cover all forms of life from microscopic viruses to enormous whales, as well as conservation, knowledge-sharing and financial policies associated with these organisms, their protection, and their use.

The first meeting took place in 1994 in the Bahamas, and COP15 is the latest. Due to delays associated with the COVID-19 pandemic, it has been split into two halves. The first part took place virtually in 2021, while the concluding part is set to take place later this year.

This was also originally due to take place in Kunming, China, but amid ongoing uncertainty due to COVID-19, this meeting has been moved to Montreal, Canada.

5

The world missed a critical deadline to safeguard biodiversity, UN report says

Back in 2010, before anybody expected the skies of California to glow an apocalyptic orange, or a pandemic to grind world economies to a near halt, representatives from 196 countries gathered in Nagoya, Japan, to tackle a different planetary crisis that is inextricably linked both to climate change and human health.

Human population growth, consumption, and transformation of natural habitats are rapidly unravelling the vibrant living fabric of the Earth in an extinction crisis that threatens to become humanity’s most enduring legacy.

The United Nations Convention on Biological Diversity (CBD)—ratified by all UN members except the United States—set 20 targets to stem the tide of biodiversity loss. With the publication of a major report this week, the final verdict is out on how well world governments have stepped up to the challenge.

The bad news is, we’ve failed. With none of the 20 targets fully met, and only six partially, the report is yet another reminder of the urgent need to redesign the way we produce, consume, and trade goods. But scattered throughout the 220-page document—a synthesis of scientific evidence, other UN assessments, and countries’ national reports—are glimmers of progress demonstrating that nature fares well when actions are taken. If we can scale them up immensely, there’s still hope for a future where humanity lives in harmony with nature.

“If actions had not even been taken on those few areas, the situation would have been even more dire than where we are today,” says Elizabeth Maruma Mrema, executive secretary of the CBD.

6

Wildlife populations have crashed by 69% within less than a lifetime

Wildlife populations have declined by a staggering 69% over the past 48 years.

This is the stark new finding from the latest World Wildlife Fund for Nature's Living Planet Report, with animals in Central and South America being particularly hard hit.

The planet's wildlife is being decimated. Within the span of a single lifetime, animal populations that scientists have monitored have declined by over two-thirds.

These shocking findings comes from the latest WWF Living Planet Report, which sets out to assess and quantify vertebrate animal populations around the world. Published every two years, the 2022 report is the most comprehensive to date, covering more species and more populations than ever before.

It shows that wildlife is facing a double threat: climate change and biodiversity loss. While these two issues are linked, they are also distinct.

Habitat loss caused by land use change, the insatiable desire of people to cut down more forests, farm more land, extract more minerals and build ever-expanding infrastructure are all taking their toll. But pollution, climate change, invasive species and disease are also of concern.

Marco Lambertini, the Director General of WWF International, says, 'We face the double emergencies of human-induced climate change and biodiversity loss, threatening the well-being of current and future generations.'

'WWF is extremely worried by this new data showing a devastating fall in wildlife populations, in particular in tropical regions that are home to some of the most biodiverse landscapes in the world.'

7

Destruction of forests and grasslands is biggest cause of biodiversity loss

The biggest direct driver of wildlife declines globally is the conversion of natural forests and grasslands to intensive agriculture and livestock.

This is the dramatic finding from a new paper that has looked into what is driving the unfolding biodiversity crisis. It found that land-use change caused the biggest effect over recent decades, followed by the exploitation of wildlife though fishing, logging and hunting, with pollution ranked third.

The planet is losing its wildlife at breakneck speed. Over the past four decades alone populations of mammals, birds, fish, reptiles and amphibians that scientists have monitored have fallen by an average of two-thirds.

But as we continue to put pressure on the natural world, through deforestation and over exploitation, we are threatening our own survival. This is not some existential threat that may come to pass in the distant future, but a reality we are seeing in the present.

Knowing what is causing this rapid decline in wildlife, known more broadly as biodiversity, is essential in helping to direct how we will stop and eventually reverse it.

A new study has assessed the potential different drivers of this decline and found that land use change is the number one cause, followed by the direct exploitation of wildlife and pollution. What is perhaps most surprising is that climate change has been only the fourth largest driver of recent biodiversity loss on land.

'Biodiversity is declining rapidly,' explains Andy. 'A million species of animal and plant are threatened with extinction, while ecosystems worldwide are changing away from their natural condition which means that they may be less likely to meet humanity's needs.'

8

We’ve overexploited the planet, now we need to change if we’re to survive

The relationship between humans and nature is under intense and increasing strain. The report released today by IPBES, the Intergovernmental Science-Policy Platform on Biodiversity and Ecosystem Services (akin to the IPCC reports on climate change), provides compelling evidence that humans are overexploiting wild species and habitats. Harmful activities, including habitat destruction, poor farming practices and pollution, have altered ecosystems significantly, driving many species past the point of recovery. In Great Britain alone, of the 8,431 species assessed in the 2019 State of Nature report, 1,188 are threatened with extinction. Globally, there are an estimated 1 million at risk, with biodiversity declining at a faster rate than at any time in human history.

We cannot ignore biodiversity loss. Biodiversity is the variability that exists among all living organisms, between different species, within species including genetic makeup, and in wider ecosystems. Billions of people rely on wild species for food, clean water, energy, income and health and wellbeing. Annually, crops worth up to £480 billion are pollinated by a variety of wild animals, and an estimated 4 billion people depend on natural medicines for their healthcare. These vital ecosystem services are fundamentally based on a healthy environment, and this requires biodiversity. Losing biodiversity leaves species and ecosystems less resilient to challenges such as invasive species or pests, meaning there is an increased risk of whole populations being wiped out and destabilising the entire ecological network. Nature is a finite resource, and human self-interest alone should determine that biodiversity must be protected.

9

How biodiversity loss could cause bankruptcy in some countries

Economists at the University of Cambridge have developed what they say are the world’s first biodiversity-adjusted sovereign credit ratings.

Their research finds that many countries are at risk of effectively going bankrupt if their credit ratings deteriorate due to ecological factors like biodiversity loss.

Sovereign credit ratings usually only weigh up geopolitical risk, but their findings suggest this will soon change because of the rapid deterioration of ecosystems.

The growing threat to the biodiversity of the planet, upon which life on earth depends, is well documented. It is declining faster than at any other time in recorded history, and this has serious implications for human health and prosperity.

Around a million animal and plant species are facing extinction according to the UN. It says human activity has altered almost two-thirds of the earth’s surface area. This is putting enormous pressure on nature and increasing the risk of zoonotic diseases such as COVID-19, as humans come into greater contact with wildlife.

Research by the Royal Society Scientific Journal also indicates that climate change is expected to become one of the largest drivers of biodiversity loss by the second half of this century. Rising temperatures and changing rainfall patterns could match or even surpass the effects of deforestation and agriculture.

However, biodiversity loss has the potential to impact economics, according to a new report from the University of Cambridge. A team of economists has drawn up what it says is the world’s first biodiversity-adjusted sovereign credit ratings. These show how ecological destruction can prompt credit rating downgrades and make borrowing costs spiral. The report says degradation of nature-provided “ecosystem services” - such as bees pollinating crops and plants that regenerate soil and prevent flooding - can create huge economic costs.

10

Time to align finance with biodiversity objectives

In celebration of the One Planet Summit for biodiversity held on 11 January, OMFIF is publishing this commentary from the latest edition of the Sustainable Development Institute Journal.

The planet is heading towards its sixth mass extinction, with 25% of plant and animal species at risk. Companies (and their financiers) are part of the problem and should become part of the solution.

The destruction of biodiversity has significant implications for the viability and profitability of companies. An estimated $44tn of global value added depends on the ecosystem services that biodiversity provides. As a result, businesses are exposed to poorly understood risks. At the same time, the negative impacts of many companies’ activities on biodiversity create significant societal risks, as well as liability risks for the companies.

Stopping biodiversity loss requires transforming production and consumption patterns. These changes can only be achieved through coherent and effective policies, and by aligning finance with biodiversity objectives. Global biodiversity spending is around $78bn-$91bn per year, mostly public expenditure. The challenge is not only to increase financing for biodiversity; it is also to reduce that which is harmful to biodiversity. Potentially harmful public support is more than five times total biodiversity spending. Total financing that is harmful to biodiversity is likely to be much higher.

Adopting an ambitious post-2020 global biodiversity framework with specific and measurable targets is vital for sending a clear policy signal to businesses and financial institutions. Governments should mainstream biodiversity into their own budgets and fiscal measures – reforming harmful subsidies, greening public procurement, and pricing biodiversity loss through economic instruments. The immediate priority, however, must be to factor biodiversity into Covid-19 recovery measures. With almost $12tn committed to date globally, spending decisions by governments could be decisive for biodiversity and therefore longer-term economic resilience and prosperity.

**Biodiversity search volume peak news**

2011.3

1.

Why invasive plants are the 'second biggest threat to biodiversity' after habitat loss

As experts gather in London for a major conference addressing the often overlooked threat of invasive species to biodiversity, Carrie Madren gets a briefing from those on the frontline in the battle against 'pest plants'

We can eat oranges from South Africa, buy electronics made in China and can fly from the US to Europe in a matter of hours. As trade and travel have skyrocketed around the world, so too has our ability to bring exotic and foreign things back with us.

For centuries, horticulturists, plant lovers, ecologists, landscapers and others have imported plants for exotic aesthetic appeal, or usefulness in righting a degraded landscape.' As trade increased and travellers had the capacity to bring more things with them, it [the trade in invasive species] has proliferated,' says Lori Williams, executive director of the U.S. National Invasive Species Council.

Enthusiastic flora introductions have brought a raft of unintended consequences with them: at first a new plant succeeds but then adapts too well to its surroundings and spreads out of control. Some of these plants flourish so much in new territories that they became threats to native species’ very survival.

As well as intentional introductions, pest plants also travel 'undercover' - hitchhiker seeds and plants arrive in crates or crevices and sneak past border controls. Seeds get stuck in tyre or shoe treads or creep in as soft packing material. Such unintentional introductions have brought in invasive grasses and other plants before stricter trade regulations were put into place.

2

How not to make your garden wildlife friendly

We're more than happy to bang on about what to do to make your garden wildlife friendly (here, here and here for example). But equally important is what not to do.

A report published this week from Plantlife has found that alien plant species are a big threat to our native wildlife. They cost more than £1.7 billion a year to clear up and affect some of our most beautiful landscapes, including the Lake District.

What's this got to do with our gardens? We've all heard of the problems caused by Japanese knotweed. No one's going to willingly plant that in their garden any more. (Scary thought: you can be refused a mortgage if your neighbour has it in their garden.)

But you can still buy some of the worst, most invasive offenders from your local garden centre. They have the potential - if they escape from gardens or ponds - to be so harmful to our biodiversity that Plantlife is calling for them to be banned.

Some of these danger plants are even sold under a 'Buy British' slogan. Don't be fooled - they've been cultivated in Britain but are as far from native as it's possible to be. And watch out for plants which are sold as 'oxygenating' plants for ponds and aquaria. Some of these bad boys can actually lead to great fluctuations in oxygen levels which are harmful to your fish and invertebrates.

All the good work you do for wildlife in your garden could be undone by planting these rascals in between your dahlias and pansies, or in your pond. So however pretty, useful or interesting they may look on those garden centre shelves, just walk on by.

3

Bees: a sting in the tale

The United Nations Environment Programme (Unep) buzzes into the ongoing discussion of bee decline this week, with a report examining the global nature of the issue and some of the reasons behind it.

Their top-line conclusions are that it's becoming a widespread, if not quite global, phenomenon, and that there's a multiplicity of causes.

Declines - and in some cases, sudden collapses - of colonies in Western Europe, North America and Japan have been widely reported

But it's perhaps not quite so commonly known that Chinese beekeepers "faced several inexplicable and complex symptoms of colony losses", as Unep puts it, or that collapses have also been seen in beehives along the banks of the Nile

As to the causes, the report highlights more than a dozen factors that could be responsible, in varying proportions, in different parts of the world, including:

diseases exacerbated by increased global movement of bees and of other things that may carry pathogens

agricultural chemicals

climatic factors

atmospheric pollution, which reduces insects' capacity to detect smells of, for example, important plants

loss of plant biodiversity, reducing the variety of bees' diet

A while back, writing about the stark and global crisis facing amphibians, I suggested that the only factor to hold responsible was "everything" - and that the same might be true for bees.

And this is basically the thesis that Unep is spelling out, through the scientific assessments of the Swiss, French and US experts enlisted to write its report.

4.

Irish hare under threat from 'European brown' hare

The Irish hare is under threat from the European 'brown' hare, according to a study from Queen's University.

The research has found increasing numbers of the brown hare have set up home in mid-Ulster and west Tyrone.

In March 2011, the Assembly voted to outlaw hare coursing in Northern Ireland to protect the future of the Irish hare.

But Dr Neil Reid from Quercus (Queen's University's Centre for Biodiversity and Conservation Science), said: "Our native hare remains vulnerable to another serious threat - that of the invading European hare."

European hares are found in Britain and continental Europe, but they have been highly successful in invading many countries beyond their native range in south-west Europe and parts of Asia.

There have been many studies on their impact on native species. Dr Reid reviewed these studies to get a clearer picture of how much of a threat the invading species might be to the Irish hare.

'Hybridising'

The study, published in the international journal Biological Invasions, suggested that European hares exhibit strong competition for habitat space and food resources with native species, most notably other hare species.

It also warns that disease and parasite transmission and climate change may give the invading European hare an edge over our native species.

Dr Reid added: "The Irish hare represents an evolutionary unique lineage, which is restricted to Ireland where it has been present since before the last glacial maximum, making it one of our few native mammal species.

"Hence, it has been isolated for 30,000-60,000 years. So the discovery that both species are hybridising in the wild is very worrying."

5

Ship container 'stepping stone' risk for alien invaders

A shipping container lost off the coast of California in 2004 is now teeming with underwater life, say scientists who returned to it with a robotic sub.

However, it is unclear whether the "artificial reef" that the container provides is a beneficial one.

Containers could provide a "stepping stone" route for invasive species to colonise new areas, the team says.

The unique study sheds light on the problem of the estimated 10,000 such containers that are lost each year.

It is estimated that some 200 million shipping containers are used globally each year, and that at any one time, between five and six million containers are in transit.

In February 2004, the container ship Med Taipei set off from San Francisco.

Caught in a storm on its coastal journey to Los Angeles, 15 containers broke free and were lost near Monterey Bay.

Four months later, on a routine remotely-operated vehicle (ROV) dive by scientists from the Monterey Bay Aquarium Research Institute (Mbari), one of the containers was spotted at a depth of 1,300m.

The container - listed as housing more than 1,000 tyres - appeared to be in pristine condition, but the Mbari team resolved to return and assess the container's effects on life on the seafloor.

Last week, Andrew DeVogelaere of the Monterey Bay National Marine Sanctuary and colleagues from Mbari took the Doc Ricketts ROV back to the site.

"What's normally down there is gently rolling, soft seabed covered with an amazing amount of life - there are these clear sea cucumbers every few feet, beautiful pink crabs, tubeworms," Dr DeVogelaere told BBC News.

6

Hen and Hammock: changing the world, one garden at a time

For many years we had a choice between buying what you really want or being a conscientious shopper and going for Fairtrade, eco-friendly products regardless of whether they actually looked good or worked. But times have changed and now, there’s a host of small companies proving you don’t have to sacrifice aesthetics to maintain your ethics. Among them are the Oxfordshire-based Hen and Hammock who sell a range of products to brighten up your garden, and believe that by choosing what we buy and sell, we all have the power to shape the future. ‘We believe that what we buy has a major impact on the environment. It is our duty as consumers to buy products which make a better world,’ says Hem and Hammock co-founder, Andrew Jones. And he’s right.

But excellent ethics aren’t the only thing the company has going for it. 10 per cent of profits are donated to non-profit organisations, including the Bumblebee Conservation Trust last year. Hen and Hammock also offsets all of its carbon emissions with Climate Care and sources its stock from suppliers who take environmental ethics as seriously as they do. Typical is garden furniture manufacturer, Arbor Vetum who use reclaimed timber. Recycling teak from early 20th century structures, Arbor Vetum became the first company in the world to be awarded the FSC Recycled label and the first in Europe to receive the Rediscovered Wood Certification by SmartWood.

7

Palm oil giants target Africa in 'land grab' following Indonesia deforestation ban

Indonesia's move to bring in a two-year moratorium on new palm oil plantations to protect its remaining rainforests has seen agribusiness giants like Sime Darby switch expansion plans to Cameroon, Ghana and Liberia

The sudden upsurge in land deals by palm oil companies in Africa could lead to large-scale deforestation and loss of farmland by local communities, NGOs and environmental groups in Africa have told the Ecologist.

Indonesia, the world's largest palm oil producer, is due to implement a two-year ban on granting new concessions of land to plantation companies in forest areas. There are also restrictions on the availability of land in Malaysia. This has led companies like Sime Darby, which has more than half a million hectares of palm oil in Indonesia and Malaysia, to look elsewhere.

Sime Darby - reported to be the largest palm oil producer in the world - has leased 220,000 hectares of land in Liberia and is considering buying a further 300,000 hectares for palm oil plantations in Cameroon. Despite the Indonesian ban, it still wants to reach 1 million hectares of plantation land worldwide by 2015. Other rival palm oil giants like Sinar Mar, Olam International and Wilmar International are also tying up land deals in Liberia, Gabon and Ghana.

For Sime Darby at least, the ambition is to target the lucrative European markets, particularly, say Friends of the Earth, for biofuels. It recently announced plans to build a processing plant in the South of France, bringing palm oil from its new plantations in Liberia.

However, Friends of the Earth say at least two of the areas in Liberia - Gbarpolu and Bong - where the company has leased land are currently heavily forested and include virgin rainforest.

8

Study of spiders shows species may be able to adapt to global warming

Species may be able to adapt to gradual increases in temperature preventing the collapse of biological communities in the face of global climate change

The predatory behaviour of spiders is unaffected by increased temperatures, according to research by Yale University, suggesting some species can adapt to global warming.

The Yale research examined a well-studied grassland food web, made up of a predatory spider, its grasshopper prey, and the plants grasshoppers fed on. The spider’s predatory behaviour is known to be temperature-sensitive, decreasing with increased temperatures. Researchers had expected higher temperatures to stop the spiders preying on grasshoppers, leading to more plants being eaten.

However, in the study, spider populations from warmer areas tolerated higher temperature ranges better than the populations from cooler areas and continued to control the grasshopper popualtion. This suggests they can adapt to local conditions and maintain their vital role in the community despite increased temperatures.

‘Species are almost certainly adapting to the climate change Earth has experienced during the past century,' study author Dr Brandon Barton told the Ecologist. 'My results show that species have the capacity to adapt to a range of temperatures, similar to those predicted by climate change models, and that a species’ role in the community can be conserved by this adaptation.’

Many similar experiments expose organisms to short-term, sudden increases in temperature, which does not allow for long-term gradual processes like climate change. Barton’s work overcomes these limitations by looking at populations along a natural temperature gradient to see if long-term changes in temperature would affect small-scale food webs. He sampled spiders at sites from a 500km, north-south axis, along the east coast of the United States, where temperature varied by 4.8C.

9

Super nature: creating a wildlife garden

From birds to dragonflies and butterflies to bees, Hazel Sillver explains how to turn your garden into a haven for wildlife

No garden is complete without wildlife. Who would be without the gentle hum of bees as they meander from one flower to another and the evening song of the robin perched on his apple tree. If you’ve had a rotten day, the sight of butterflies dancing in the late sun will cheer you up as you sip your G&T on the terrace, as do the daredevil antics of squirrels playing in the walnut tree. It’s a myth that a manicured garden doesn’t attract wildlife. You don’t need to live in a field or cultivate a jungle of nettles and rambling plants to have a garden that provides a habitat for a diverse range of birds, animals and insects. However, it’s true that if you pave over most of your garden, you will end up being the only living thing in it. Wildlife prefers vegetation to patios and decking. Likewise, chemicals and wildlife don’t mix, so it’s best to garden organically where possible. Butterflies, bees, birds and dragonflies all need specific plants and conditions to feel at home in your backyard. Here’s how to encourage them to take up residence in your garden.

10

Europe moves to ban imports of tar sands oil from Canada

An attempt to classify tar sands oil as more environmentally-damaging than conventional oil would effectively ban its sale within European Member States

The European Union is moving to prevent tar sands oil from entering the European market due to the greenhouse gas emissions (GHG) associated with its production.

Oil from the tar sands industry is set to be classified as having greater GHG emissions than conventional oil in a review of the European Union’s Fuel Quality Directive. Recognising the greater environmental impact of tar sands oil will effectively ban its use in EU states, where fuel providers are legally bound to aim for 6 per cent reductions in GHG emissions by 2020.

Tar sands are deposits of oil-rich bitumen mixed with clay and sand embedded in rocks often buried beneath the surface. Two tonnes of topsoil have to be removed to produce each barrel of bitumen, creating vast open mines. Extracting the deposits is estimated to be three times more carbon-intensive than conventional oil sources. It also causes the loss of natural habitats with vast areas of boreal forest cleared in order to mine the sandy bitumen – and pollution of local waterways with toxic chemicals.

The largest reserves of tar sands in the world are held in Canada, which plans to increase production from 1.5 million to 7 million barrels a day by 2020. Both Shell and BP are major investors in tar sands with BP having acquired a 50 per cent stake in the Sunrise Project, a tar sands extraction site near Alberta.

The EU had previously dropped the attempt to classify tar sands oil as more environmentally-damaging than conventional oil after strong lobbying by the Canadian government and the oil industry. However, following a year-long campaign by a coalition of environmental groups led by the Co-operative and including WWF, Greenpeace, Friends of the Earth and Transport & Environment, it is now being reconsidered.

2020.6

1.

View from India: Biodiversity, as seen from an Indian perspective

World Environment Day (WED) is celebrated annually on June 5. According to the United Nations, the theme for 2020 is biodiversity - a concern that is both urgent and existential.

The United Nations Environment Programme (UNEP) has announced that Colombia will host World Environment Day 2020 in partnership with Germany.

Biodiversity provides sustenance for communities living on the coastline or fringe areas. Biodiversity also plays a significant role in agriculture. Bio-pesticides, fodder and fuel are some of the benefits that the farming communities can enjoy when there’s an ecological balance of biodiversity. It’s crucial to protect biodiversity in order to prevent mass extinction of several species of animals.

Like biodiversity, wetlands also need to be safeguarded. Bird species rely on wetlands for breeding, nesting, as well as a place of shelter. Wetland-dependent species face habitat loss when wetlands go unprotected.

Here, View From India takes readers through a national programme conceptualised for the preservation of biodiversity and avian diversity in the country.

Several incidents relating to the bird community have recently made news in the media and gone viral. In February 2020, reports about an oriental darter struggling to free its beak from a piece of plastic touched many hearts. This echoed similar sentiments expressed when news broke of a black-necked stork’s beak being sealed with a plastic ring in June 2018. Both occurrences have happened in wetlands and resulted in an ecological imbalance. The wetlands require conservation. Being bird habitats, they need to be safeguarded from plastic trash.

All this and more has become a clarion call for the Government of India (GoI) to initiate a Visionary Perspective Plan (VPP). The Ministry of Environment, Forest and Climate Change has come up with a Draft of the VPP (2020-2030) for the conservation of avian diversity, their ecosystems, habitats and landscapes in the country.

2

City Council publishes Biodiversity Review for Oxford City Council Parks and Nature Areas 2020

On World Environment Day, Oxford City Council has published a Biodiversity Review for its parks and nature areas.

This document sets out what initiatives have already been put in place to further protect and increase biodiversity in the green spaces managed by the Council, and identifies what additional steps can be taken to ensure these vital urban habitats are achieving their full potential.

The document complements the 2015 Biodiversity Action Plan (2015-2020) which identifies the council’s wider sustainability policies and initiatives, and provides an overview of the national context for the biodiversity of Oxford.

ODS manages just over 600 hectares of accessible green space in the city and surrounding area on behalf of the City Council, including a country park, 33 nature areas and over 60 urban parks. Oxford also has 12 Sites of Special Scientific Interest, covering 278 hectares and including four managed by the city council; Port Meadow (which has been meadowland for at least 4000 years), Wolvercote Common, Shotover Country Park, and Lye Valley Nature Reserve.

Biodiversity Review for Oxford City Council Parks and Nature Areas 2020

The review explores the projects and initiatives which have been undertaken to support biodiversity and improve sustainability in the last five years, including planting 7000 new trees across the city, work with Treeconomics to prepare a tree ‘Canopy Cover Assessment’ around Oxford’s Urban Forest, 5,000 hours of conservation volunteering, the creation of biodiversity action plans, and improved green waste management.

3

Nature crisis: New global extinction target proposed

The world needs a single goal for fighting the loss of nature, much like the 1.5C target for climate change, according to conservation experts.

Extinctions of plants and animals should be kept well below 20 per year, they propose.

Last year, a UN report found that around one million species are now threatened with extinction.

Progress on biodiversity loss has been "far too slow, limited or ineffective," said Prof Georgina Mace of UCL, London.

Nature crisis: Humans 'threaten 1m species with extinction'

Achieving the target should ensure that natural systems "continue to function and meet the needs of people and the rest of life on Earth", she said.

The coronavirus pandemic has put the spotlight on the critical balance between nature and people. Scientists have long warned that close contact with wild animals through hunting, trade or habitat loss puts the world at increased risk of outbreaks of new diseases.

Yet, the timetable for setting new global biodiversity goals has been thrown into disarray by the pandemic. Meanwhile, we are running out of time to deal with the threat, conservation scientists warned last week.

They said human actions had pushed 500 mammals, birds, reptiles and amphibians to the brink of extinction, which is yet more evidence that the world is undergoing a sixth mass extinction.

In the new proposals, published in the journal Science, conservation experts in the UK and Germany call for a long-term goal to reduce species extinctions towards natural rates, with an easily measurable objective of fewer than 20 extinctions a year.

4

Biodiversity Net Gain

On 4 June 2020 our Planning & Environment team hosted a webinar to look at the Government’s proposals in the Environment Bill for mandating biodiversity net gain for new developments. Here we summarise the main issues that were discussed and give our thoughts on how the proposals might work in practice when (and if) they are implemented.

What is the current legal and policy position?

LPAs are currently subject to duties under the Natural Environment and Rural Communities Act 2006. They must have regard to the purpose of conserving biodiversity (which includes restoring or enhancing a species or habitat). This is difficult to enforce, as they only need to consider the duty.

The Environment Bill is going to strengthen the aspects of that duty that deal with enhancing biodiversity, which is part of a consistent theme running through the Bill of improving (rather than just conserving) the environment.

NPPF requires policy and decisions to contribute to and improve the natural local environment and minimise impacts. Requires LPAs to refuse applications if deemed harmful to biodiversity if the harm cannot be avoided through relocation, mitigation or compensation (but only as a last resort). Those developments that promote biodiversity are to be encouraged.

Planning Practice Guidance says net gains can be secured on site, off site or a combination of the two, through planning conditions, planning agreements or unilateral undertakings.

The effect of the Bill is to take the existing policy and guidance on biodiversity and turn them into statute.

5

DROUGHT, DESERTIFICATION AND DRYLANDS BIODIVERSITY

Desertification and drought are threatening lives and livelihoods. Harnessing dryland biodiversity in ecosystem restoration could help communities to respond to this threat as well as mitigate against and adapt to the related negative effects of climate change.

The 17th June is the World Day to Combat Desertification and Drought, which this year focuses on ‘Food. Feed. Fibre.’ and the links between consumption and land.

Drylands and Desertification

Drylands (arid, semi-arid and dry sub-humid areas) make up around 40% of the world’s land area and are areas of economic, social and environmental importance. They are home to more than two billion people in both rural and urban communities, including some of the world’s biggest cities, like New Delhi.

They can be biodiverse, productive landscapes; the first global assessment of dryland forests found that drylands include two thirds of the land area of 7 biodiversity hot spots and contain 1.1 billion hectares of forest, corresponding to over a quarter of the world’s forest area.

However, many drylands are rapidly degrading due to climatic variations and human activities such as deforestation and unsustainable land use. This degradation is also known as desertification.

Today, more than two billion hectares of previously productive land is degraded. Each year, 12 million hectares of land capable of producing 20 million tonnes of grain are lost due to drought and desertification.

Desertification is also exacerbated by drought, with an average of 70 countries affected by droughts each year.

6

COLOMBIA HIGHLIGHTS THE IMPORTANCE OF BIOCULTURAL DIVERSITY

Colombia is one of the most biodiverse countries on Earth and is the global host for World Environment Day 2020 on the 5th of June.

While events on the ground have been cancelled due to COVID-19, Colombia continues its crucial role as host by inspiring countries worldwide, demonstrating how the connection between people and nature can, and should be at the centre of decision-making.

Colombia is home to over 48 million people, mostly of Indigenous, European and African descent. The country has coastlines in both the Pacific Ocean and the Caribbean Sea. Its territory is crossed by the Andes, whilst the Amazon rainforest dominates its southern landscape. Colombia harbours over 51,000 species and 300 different ecosystem types. Sustainable development is, therefore, key to safeguarding Colombia’s rich biological and cultural diversity for people and for nature.

National Ecosystem Assessments are tools for sustainable development

The contributions of nature to people can be difficult to quantify and so are often overlooked and undervalued in national policy-settings and decision-making processes. National Ecosystem Assessments can support sustainable development at the policy level by providing governments with an up-to-date and robust evidence base about the true value and the benefits of biodiversity and ecosystem services and identify gaps in knowledge and understanding. Even more, by ensuring the engagement of key stakeholders in their development, findings from National Ecosystem Assessments are generally supported across sectors.

UNEP-WCMC is working with Colombia – along with 11 other countries - in building capacity for the development of National Ecosystem Assessments as part of its efforts to support the Intergovernmental Platform on Biodiversity and Ecosystem Services (IPBES).

Colombia’s National Ecosystem Assessment report, which will be launched later this year, is especially important as it recognises biodiversity as being critical for both the country’s economy and society.

7

How forest loss has changed biodiversity over the last 150 years

by Maria Dornelas, University of St Andrews; Gergana Daskalova, University of Edinburgh, and Isla Myers-Smith, University of Edinburgh

The Earth’s forests have been changing ever since the first tree took root. For 360 million years, trees have grown and been felled through a dynamic mix of hurricanes, fires and natural regeneration. But with the dawn of the 17th century, humans began replacing large swathes of forest with farms and cities.

The global pace of deforestation has slowed in the 21st century, but forests are still disappearing – albeit at different rates in different parts of the world. Boreal forests, which grow in the far north of the world and across vast areas of Canada and Russia, are expanding further north as the climate warms, turning tundra into new woodland. Many temperate forests, like those in Europe, saw their greatest destruction centuries ago. But in the tropics, forest loss is accelerating in previously pristine wilderness.

As forest cover has fluctuated over time, the biodiversity within forests has changed too. Forests support around 80% of all species living on land, but the species we see on our woodland walks today are likely to be different from those people saw in the past. Many species, such as the Alpine longhorn beetle, survive in intact old-growth forests, while species like the red fox have managed to thrive in areas with higher human impact.

We wanted to know how changes in biodiversity worldwide are linked to changes in the world’s forests, but this was always difficult, as the effects of forest loss vary from one place to the next. How biodiversity shifts over time following forest loss hadn’t been explored across the globe – until now.

8

Canon teaches young people wildlife photography to help preserve biodiversity

Canon's Young People program champions preserving biodiversity and supporting disadvantaged communities

As we celebrate World Environment Day, it's important to shine a light on companies using their power and reach for good. Canon and its Young People program in South Africa has been working with Mike Kendrick, the founder of the local non-profit organization Wild Shots Outreach. Together, they've been teaching wildlife photography to young people in local black communities to help them embrace their natural heritage and protect local biodiversity.

The Wild Shots Outreach program prioritizes high school students from government schools bordering Kruger National Park, one of Africa's largest game reserves. Despite their proximity, the majority of local young people living next to the reserve have never visited it or seen the wildlife it houses. This is due to the lingering impact of apartheid, expensive entry fees and the lack of transport.

Wild Shots Outreach addresses the need for more young people to experience the wildlife and wild places that they previously had little or not access to. By supporting young South Africans in this way, WSO and Canon's YPP are able to help them access their wildlife heritage and develop employment skills in wildlife conservation and tourism through photography.

However, as well as helping young people from disadvantaged communities, the project's additional hope is that "the battle for conservation can be won by sharing the beauty and value of South Africa's wildlife with all of its citizens."

9

Bird survey shows thriving ecosystem at Coal Authority sites

The survey, carried out in collaboration with the University of Hull and run by final-year geography student Rosie Jaques, showed our two sites support a total of 31 different bird species.

Our Woolley and Strafford mine water treatment schemes, in Barnsley, were chosen due to their diverse landscapes, which include reed beds that act like large filters in the final stage of our treatment process to remove iron from mine water before it is discharged into rivers.

Some of the species we found have populations that are declining or have been found in fewer areas nationally over recent years and appear on the red and amber lists of Birds of Conservation Concern.

The birds use all the different habitats that are present, such as the lagoons, and while it is smaller, the Strafford site has a significantly higher bird density.

This could be due to having more habitats in a smaller space, which are not only better-connected, but also form a mosaic rather than discrete blocks.

Rosie recorded 4 red-listed species of birds, including grey wagtails, linnets, house sparrows and yellow hammers.

In addition, there were 8 amber-listed species, including black headed gulls, house martins, kestrels, kingfishers, mallards, reed buntings, swifts and willow warblers.

Abby Moorhouse, Senior Geochemist with the Coal Authority, said:

It is so gratifying to see that our reed beds, which do an excellent job removing iron from mine waters, provide further benefits for the environment by creating habitats for other wildlife.

We value the work carried out by the university, which has increased our understanding of the biodiversity at our sites.

10

The Natural History Museum to host live high-profile interactive panel discussions on biodiversity and plastic pollution - chaired by adventurer and environmentalist David de Rothschild

The Natural History Museum is partnering with explorer and environmentalist David de Rothschild, to deliver two live panel discussions on its YouTube channel and Facebook page in celebration of the UN’s World Environment Day.

In his role as an ambassador for the Museum and Founder of Voice for Nature Foundation, David de Rothschild, will host the events on Friday 5 and Sunday 7 June, joined by a phenomenal line-up of experts, celebrities, and policy-makers to open-up debate and engage viewers with the challenges facing biodiversity and the catastrophic problem of plastic pollution. Viewers will be able to take part in a Q&A with the panel during the live discussion.

David de Rothschild said: “I am extremely excited to be hosting this series of discussions for the Natural History Museum with such a fantastic line up of speakers. The Museum has always been close to my heart and I hope that together we can inspire people that their own actions, both big and small, can help to reduce the impacts of biodiversity loss and plastic pollution. This World Environment Day, it is more important than ever to keep the conversation alive on what we can all do to protect our planet - It’s time for Nature.”

The first of the two events, “It's Time for Nature” will take place on World Environment Day, Friday 5 June at 6pm, on the Natural History Museum’s YouTube channel. David de Rothschild will be joined by Ben Fogle, UN Wilderness Patron; Sam Barratt, Chief of the Education and Youth Unit in UN Environment’s Ecosystems Division; Elise van Middelem, founder of SUGi; as well as the Natural History Museum’s Botanist, Dr Sandra Knapp.

2021.2

1

The banking industry’s role in the biodiversity crisis

By now, the leading causes of biodiversity loss, deforestation, pollution, urbanisation, and climate change are widely understood. So too, are the industries that are primarily responsible for this – agriculture, food production, mining, tourism, and fossil fuels. However, far less widely known is the banking sector’s role in secretively financing these industries. Banks are keen to publicise their involvement in environmentally sustainable initiatives and emphasise their green credentials. Despite this, banks provided more than £1.9 trillion to industries that are the primary drivers of biodiversity destruction in 2019 alone.

Portfolio Earth’s recent report “Bankrolling Extinction”, examined the investments of 50 major banks worldwide and found they have collectively invested approximately $1.25 trillion into industries that cause direct biodiversity loss, and $0.65 trillion into industries that contribute to indirect biodiversity loss. These investments ranged from $1.3 billion to over $210 billion between banks.

Biodiversity is defined as the variety of biological life on Earth and has immense value for the continuing function of Earth’s ecosystems. However, species loss is currently occurring at 100 to 10,000 times the background extinction rate, fuelling the biodiversity crisis. In fact, the UN warns that one million species are at risk of extinction due to human activities. Animal and plant species perform many vital functions, including regulating global temperature and moisture levels. Therefore, unchecked biodiversity loss could have disastrous implications for global climate change.

Banks have a symbiotic relationship with the capitalist system – neither one would survive without the other. Environmental activists such as George Monbiot are increasingly declaring the current capitalist system to be fundamentally incompatible with responsible planetary stewardship.

2

Delaying action on biodiversity by just 10 years will be twice as expensive as acting immediately, reveal Natural History Museum & Vivid Economics

The Urgency of Biodiversity Action compares the cost to the world’s governments of two strategies for achieving forest conservation goals by 2050: acting now or putting action off for a decade. There is no doubt that biodiversity is in rapid global decline; or that large-scale conservation and restoration of natural forests is needed to both safeguard wild species and slow dangerous climate change. But is restoration urgent as well as important – or could we get away with waiting for a decade before we act? A team of biodiversity researchers from the Natural History Museum joined forces with strategic economist consultants from Vivid Economics to answer this unresolved question.\

The answer is clear: we cannot afford to delay.

Citizens worldwide will have to pay twice as much if policymakers delay global action by as little as ten years rather than acting immediately.

If action is delayed, it may not be feasible to stabilise biodiversity globally – even at today’s depleted level – by 2050. The pace at which biodiversity and species are being lost is speeding up; the analysis finds any delay makes it even harder to restore nature and therefore less likely it will be economically and politically feasible. The global cost of food and materials production from 2021 to 2050 is lower under immediate action and higher if action is delayed, as a share of global-average household income

3

Post-2020 global biodiversity framework must focus on sustainable use, strong indicators and the links with human health

With governments meeting this week to discuss targets and indicators for the post-2020 global biodiversity framework, TRAFFIC urges Parties to strengthen global efforts to ensure trade and use of species is legal, at sustainable levels and safe, and effectively measure progress on the implementation of these efforts.

Parties to the Convention on Biological Diversity (CBD) will meet this week for an Informal Virtual Session of the CBD’s 24th meeting of the Subsidiary Body on Scientific, Technical and Technological Advice (SBSTTA 24), with a key agenda item being the post-2020 global biodiversity framework – including the targets that will replace the current Aichi Biodiversity targets set for the Strategic Plan for Biodiversity 2011-2020.

The updated zero draft of the post-2020 framework published in August 2020 currently features a target that directly addresses the issue of wildlife trade, and commits Parties to "ensure that the harvesting, trade and use of wild species of fauna and flora is legal, at sustainable levels and safe" by 2030.

This is the first time since the Convention’s entry into force in 1993 that a target specifically addressing wildlife trade is being considered. The current draft also includes a target that recognises the importance of considering the benefits to conservation and humans of sustainable use, with a target urging that "By 2030, ensure benefits, including nutrition, food security, livelihoods, health and well-being, for people, especially for the most vulnerable through sustainable management of wild species of fauna and flora".

4

Ossiam launches ‘Food for Biodiversity’ ETF focused on habitat preservation

Paris-based Ossiam has launched an ETF providing exposure to a portfolio of global food stocks optimized to minimize habitat and biodiversity destruction.

The Ossiam Food for Biodiversity UCITS ETF has listed on Deutsche Börse Xetra and is available to trade in euros (F4DE GY) or US dollars (F4DU GY).

Bruno Poulin, CEO of Ossiam, commented, “To address the increasing destruction of our natural environment, all sectors of the economy must contribute in numerous ways before it is too late.

“The Earth’s population is predicted to reach about 11 billion by the end of this century, but if current food production methods and diets are not drastically improved, there are likely to be calamitous environmental, social, and political outcomes.

“Mobilising capital and engaging with companies in the broad food and agriculture sectors is one way of making a positive contribution to the immense environmental challenges facing humanity now.”

Although technically actively managed, in the sense that it is not referenced to a specific index, the ETF follows a systematic rules-based model in its investment approach.

The initial investment universe comprises large- and mid-cap developed market equities of companies operating in the food sector. Eligible firms must have market capitalizations greater than $1bn and average daily trading values above $4 million.

All sub-sectors of the food supply chain are represented including agriculture, manufacturers, retailers, distributors, restaurants, and packaging food producers.

5

Scientists call for urgent efforts to accelerate in-depth studies of plant groups, especially in tropical, biodiversity-rich countries

A new paper, published today in the journal Trends in Plant Science, takes a critical look at the 300 year old tradition of botanical monography to determine its role and relevance in addressing some of the challenges arising from the current environmental crisis.

A botanical monograph is a publication that focuses on a plant group, bringing together all known information about those plants, compiled by an expert. The information comes from observing plants in habitat, specimens in botanical gardens and herbaria, and looking at the tree of life and various other attributes of the plant group.

Unprecedented changes in earth’s biodiversity/ biosphere are prompting urgent efforts to describe and conserve plant diversity and scientists argue monographs can help. Plant groups with an up-to-date monograph provide a robust baseline for deeper study, accurate conservation status assessment and conservation actions that can help to protect plants that are increasingly disappearing. However, unfortunately, many important plant groups don’t have a monograph.

Dr Sandra Knapp, Merit Researcher at the Natural History Museum, who co-wrote the paper says;

“Plant diversity is the fabric of ecosystems – understanding their identities and evolutionary relationships is more important now than ever and can play a significant part in conserving the diversity of our planet. Saving our planet begins with knowledge, understanding and appreciation of the other forms of life we share it with, including plants. Tragically these are disappearing at an alarming rate of knots, and unless we act now to accelerate our understanding, we risk losing many species, along with the irreplaceable knowledge they contain.”

6

THREE MORE COUNTRIES START NATIONAL ECOSYSTEM ASSESSMENTS

The Dominican Republic, Malawi, and Thailand are starting National Ecosystem Assessments (NEA), taking a further step towards accounting for nature and its contributions to people in policymaking on a national level.

The incorporation of these three countries into the National Ecosystem Assessment Initiative was celebrated at a recent workshop attended by the Dominican Republic’s Vice-Minister of Environment for Protected Areas & Biodiversity and Malawi’s Director of Environmental Affairs.

Working with UNDP and UNESCO within the framework of the Biodiversity and Ecosystem Services Network (BES-Net), UNEP-WCMC is engaging with and supporting 11 national governments in varying stages of undertaking NEAs. Three new countries beginning the process is an important milestone; NEAs summarise and evaluate critical knowledge on biodiversity and ecosystem services that can be used by decision-makers to inform policymaking across a range of sectors.

Nature and its contributions to people can be difficult to measure and are often overlooked and undervalued in national policies and decision-making processes worldwide. NEAs help to address this by establishing an up-to-date and robust evidence base which paves the way for greater consideration of the multiple values of biodiversity and ecosystem services in policymaking.

Policies that account for the values of biodiversity and ecosystem services can help to rebalance the relationship between people and nature and encourage sustainable development across sectors. Driven by stakeholders, NEAs can also help to mobilize different knowledge systems and actors for biodiversity conservation and sustainable use of nature. The NEA process is often country-wide and combines science and indigenous and local knowledge, across communities, sectors, and levels of government to build a relevant, broadly-owned, and credible evidence base.

7

We need to act now to save nature

Nature keeps all of us alive. If we don't look after it, millions of people all over the world will face sickness and starvation in the coming century.

But nature is struggling, and it needs our help. Animals and plants everywhere are disappearing. It is vital that we stop nature's decline - and we need to act now, together.

A new report commissioned by the Treasury has done the maths on how much it might cost the world to properly protect the natural environment, and whether we can afford to put it off for now.

It has found that it is twice as expensive to delay acting than it is for governments to act immediately. Urgent change is needed to stem losses before it becomes much more difficult.

Prof Andy Purvis, a biodiversity expert at the Museum, worked on the report jointly with Vivid Economics and fellow Museum researchers Dr Adriana de Palma and Ricardo Gonzalez.

It comes a week after a review by Prof Sir Partha Dasgupta, which Andy and colleagues contributed to, recommended that the world changes its economic systems to better protect nature.

Andy says, 'Action to conserve the natural world must be taken at some point. If it isn't, we know that hundreds of millions of people will die. We all know that we have to change the way we live in order to avoid a huge loss of life and wellbeing. To do otherwise would be criminally negligent.

'Tackling the biodiversity crisis is not free - but it's far cheaper to do it now than to kick the can down the road for future generations.'

8

UN calls for end to ‘war on nature’

The world is on track to be at least 3°C above pre-industrial levels by 2100 warns the United Nations Earth Programme

UNEP’s report, Making Peace with Nature, argues that climate change, biodiversity loss and pollution pose a ‘triple threat’ to human survival and says that they can only be transformed by how we manage our economies and feed ourselves.

In his foreword, UN secretary general António Guterres says, starkly: "Humanity is waging war on nature. This is senseless and suicidal. The consequences of our recklessness are already apparent in human suffering, towering economic losses and the accelerating erosion of life on earth, threatening our viability as a species."

Based on findings from assessments from expert international panels, the report provides a clear global analysis of current and projected human-induced environmental change. Advocating the widespread adoption of the UN’s Sustainable Development Goals, it calls for sustainable local and national economies, driven by renewable energy and nature-based solutions

The report reveals that the global economy has grown nearly five-fold in the last 50 years fuelled by a tripling in extraction of natural resources and energy. Although average prosperity has doubled, around 1.3 billion people are classed as poor and 700 million go to bed hungry each night. A million species of plants and animals currently threatened with extinction. Events like the COVID-19 are increasingly likely, as we continue to strip away species' natural habitats.

The report recommends that protected areas are expanded and calls for reducing fossil fuel subsidies as an "easy win" in the fight against climate change.

9

SUCCESSFUL PROTECTED AREAS ARE A MATTER OF QUALITY, NOT JUST QUANTITY

Protected areas offer vital solutions to some of the most pressing global challenges including climate change, species extinction, water scarcity, food insecurity and poverty. They provide safe havens for endangered species, store carbon, generate much of our clean air and water, harbour sites of cultural and spiritual importance, and support the livelihoods of millions of people.

Protected areas have a critical role to play in tackling the global nature crisis. However, their success in helping to halt and reverse biodiversity loss depends not just on how big they are or where they are placed, but also critically on their effective and equitable governance and management, as well as how they are integrated into wider landscapes and seascapes.

Current efforts to ensure the effectiveness of existing protected areas lag far behind efforts to designate new protected areas. Expansion is often prioritised over effectiveness of existing areas, despite effectiveness being essential for success. A recent global review of protected area effectiveness reinforced that to be more effective, area-based conservation efforts need to be better funded, climate smart, and equitably managed.

However, it’s challenging to determine globally how effective protected areas are in delivering their conservation objectives.

There is no fit-for-purpose global measure of whether protected areas are delivering the conservation outcomes they were designed to provide. While the global coverage of protected areas is comprehensively monitored in the World Database on Protected Areas (WDPA), many of the elements that indicate the effectiveness of protected areas, such as the adequacy of resources and management capacity, and biodiversity trends in protected areas are not sufficiently tracked locally or globally.

10

Explosive study shows the wildlife trade plays a major role in the rapid loss of the world’s species

The biodiversity crisis, aka the rapid disappearance of essential diverse life on earth, is – alongside the climate crisis – one of the most pressing issues on the planet right now. The rich tapestry of life on earth is responsible for the ecosystems that humans, and all living beings, depend on for survival and health. But the world’s species are disappearing at an astonishing rate.

The causes of biodiversity decline are largely human-led, such as the climate crisis, pollution and destruction of wild spaces. An explosive new paper also shows that the wildlife trade, in all its forms, bears a huge responsibility for the crisis.

The findings raise serious questions about the adequacy of the current legal international wildlife trading system.

Dramatic declines

The wildlife trade is a huge industry which involves the commodification of wild species for food, medicine, fashion, luxury goods, entertainment, laboratory testing, the pet trade, hunting trophies and more. It involves both legal and illegal trading, and it happens at a local, national and international level. The legal trade is reportedly worth around $300bn a year. The EU’s legal wildlife trade alone, for example, amounts to €100bn annually.

The paper, which is the work of a team of international scientists from the University of Sheffield, Norwegian University of Life Sciences and the University of Florida, looked into the relationship between species declines and the wildlife trade. Specifically, they investigated the declines of 133 land-based species that were subject to trade. Their analysis included legal and illegal trading, at all aforementioned levels (local, national and international). The ‘trade purposes’ they focused on were the bushmeat trade, the pet trade, ivory, traditional medicine and laboratory use.

2022.3

1

Biodiversity: Pressure grows for deal to save nature

A global agreement to reverse the loss of nature and halt extinctions is inching closer, as talks in Geneva enter their final day.

International negotiators are working on the text of a UN framework to safeguard nature ahead of a high-level summit in China later this year.

Observers have slammed the "snail's pace" of negotiations and are pressing for a strengthening of ambitions.

Divisions remain, including over financing the plans.

"The science is very clear, we do not have any more time to waste; we need to take action now," Bernadette Fischler Hooper, head of international advocacy at WWF-UK, told BBC News.

"Not only on biodiversity loss, but also on climate change which is a very inter-linked issue. So that is what's at stake here; it's actually the future of the planet and its people."

Biodiversity loss risks 'ecological meltdown'

Biodiversity: Why the nature crisis matters, in five graphics

Five key things about the extinction crisis

The final version of the draft UN Convention on Biological Diversity (CBD) will be negotiated in Kunming, China, at the Cop15 summit, which is expected to take place at the end of August.

What's needed to save animals and plants from extinction?

The outcome will decide for the coming decades how the world will address the challenges of reducing the extinction risk threatening more than one million species, protecting 30% of land and sea, eliminating billions of dollars of environmentally-damaging government subsidies and restoring degraded ecosystems.

2

Wildlife trade and the Post-2020 Global Biodiversity Framework

As governments and experts meet face-to-face in Geneva for discussions under the Convention on Biological Diversity (CBD), TRAFFIC has submitted a recommendations document concerning the Targets of the Post-2020 Global Biodiversity Framework (GBF), and a proposal for the development of a robust new headline indicator to monitor the implementation of the Target 5.

Following meetings in September 2021, TRAFFIC experts join Subsidiary Body on Implementation (SBI), Subsidiary Body on Scientific, Technical and Technological advice (SSBTA), and Open-Ended Working Group (OEWG) meetings, suggesting how to strengthen Post-2020 GBF Targets and how to ensure its implementation.

As we draw ever closer to the adoption of the post-2020 Global Biodiversity Framework, this is a critical time to ensure that these transformative Targets, to address the worrying levels of biodiversity decline, can be effectively implemented by the national and international community.

Safeguarding the sustainable use of wild species can directly combat the threats of unsustainable and/or illegal use, including commercial and international trade. TRAFFIC has proposed a process to develop an indicator for Target 5 to measure the sustainable use of wild species by 2030.

Unlike many other indicators that look at benchmarks on a global scale, this indicator will work from the grassroots up. The concept is to initially collect national data with the potential to aggregate up to a regional or global indicator – making sure all parties are successfully implementing the Target ‘on the ground.’

3

Five ways that high-tech maps can help protect biodiversity

Conservation technology is helping to track and protect species on the brink of extinction.

High-tech maps can predict where species will move in response to climate change, and provide a clear picture of which species are most at threat.

This technology can also monitor protected land, and analyse photographic evidence about species and how they are surviving.

With nearly 1 million species facing extinction, it has never been more important to know where threatened and endangered wildlife are located and how they are responding to threats, from habitat destruction to invasive species and climate change. Conservation technology is offering a solution. Using advanced tools such as remote sensing, artificial intelligence (AI) and statistical modelling, we are changing the way we protect species on the brink.

Here are five ways that high-tech maps are helping to protect biodiversity:

1. Predicting where species are moving in response to climate change

As the world warms, thousands of species are shifting their ranges to escape rising temperatures and precipitation patterns that have permanently altered their habitats. While these migrations are happening over many years and many generations, this is the beginning of a redistribution of the entire planet’s ecosystems – a process that could upend the delicate ecological relationships we all rely upon to survive.

It is essential to predict where roaming wildlife will end up. But doing so isn’t always easy, as each species is moving in a different direction and at a different pace to avoid a different climate impact. In response, scientists and policy experts from over 20 institutions created the Spatial Planning for Area Conservation in Response to Climate Change (SPARC) tool.

4

£2.3m secured to improve facilities, walking routes and biodiversity at West Reservoir

A more accessible, active and biodiverse West Reservoir could soon take shape after Hackney Council secured £2.3m in funding to deliver a project to improve it for local residents.

Made up of £700,000 from the Greater London Authority’s (GLA) Green and Resilient Spaces Fund, and £1.6m secured from developers, the project aims to: better connect West Reservoir with the local community and other green spaces, including the nearby Wetlands; increase biodiversity; and, improve facilities at West Reservoir.

Subject to engagement with local organisations and residents, the project could deliver a new accessible green space for local residents on the east side of the reservoir, allowing people to circulate around the perimeter of the reservoir, a new accessible bridge across New River, improved landscaping and wildlife habitats, and more accessible walking and cycling routes.

An improvement board composed of community and partner organisations will be set up to ensure the proposals best meet the needs of existing users and local people.

Design and engagement work on the project is set to begin in June 2022.

This funding is great news for West Reservoir and we’re going to use it - in consultation with local residents - to create new green space, make the area more accessible and active for local people, and improve it for wildlife.

5

Biodiversity loss has knock-on effects on global markets

Biodiversity losses in countries with smaller, less-developed economies, impact large, developed economies, according to a new study published in People and Nature.

Researchers from Trinity College Dublin and University of Reading have shown that pollinator decline and potential loss of pollination services can have widespread implications for global trade in food.

Bees and other pollinating animals are vitally important for the production of many leading food crops and commodities. From apples to strawberries, to chocolate and coffee – animal pollination is important to meet our demands.

However, changes in land use and climate, and the use of some chemical insecticides, threaten pollinators worldwide. Because we often import crops that we cannot grow ourselves, pollinator losses in our trading partners could have a significant impact on prices at home.

A new study by Trinity College Dublin and the University of Reading, funded by the Irish Environmental Protection Agency, has examined the effects of pollinator losses in small groups of countries on world food markets.

The team developed a simple economic model to examine what the effects of world crop prices would be if pollinators were lost in different countries. They applied this model by looking at pollinator losses in three groups of 25 countries: 1) Economically vulnerable countries with high debts and low incomes, 2) countries that were very vulnerable to natural disasters and 3) countries with very high use of pesticides.

“What we see in these three case studies is a fairly consistent pattern: the countries which suffer the biggest economic losses because of rising prices are large, well-developed economies that import a lot of pollinated crops like Germany, Japan, China, India and the UK.

6

Why wetlands are a versatile climate and biodiversity hack

The potential of wetlands to mitigate climate change and extreme weather events has been neglected.

More than a third of global wetlands have disappeared since the 1970s.

Quantified targets are needed for policy-makers to protect wetlands.

What’s the best and cheapest way to harness nature for fighting climate change? Answer: protect and restore the world’s wetlands – its bogs and lakes, mangroves and mires, peatlands and rivers, tidal mudflats, and floodplain marshes.

What’s the best way to counter extreme floods and droughts in a rapidly warming world? Protecting and restoring wetlands.

Which biodiverse ecosystems are disappearing faster than forests, but so far have received little attention from the Convention on Biological Diversity? You guessed it: wetlands again.

The many ecological benefits provided by the world’s wet places are too often neglected in discussions about both climate change and biodiversity protection – by scientists, conservationists and policy-makers alike. As a result, from the mangroves of South-East Asia to the marshlands of South America and the swamps of Central Africa, they continued to be drained and dammed with impunity.

At the recent Glasgow climate conference, nations lined up to pledge an end to deforestation and to start restoring forests. But there were few equivalent promises for ending wetland loss, or re-wetting drained land, even though wetlands are extremely important natural carbon stores.

Much the same has been happening at the latest negotiations in Geneva for the post-2020 global Biodiversity Framework under the Convention on Biological Diversity – even though wetlands contain more biodiversity than forests, holding 40% of the planet’s species on just 7% of the land.

7

Ecotricity explains: Boosting biodiversity at our green gas mills

We’re in a climate emergency. Today, only around 30% of our electricity is generated by truly renewable means – technically, it could be 100% by now if the political will was there.

Electricity is only half the story. Heating our homes and powering industry with fossil gas is one of the biggest sources of carbon emissions. How can we slash the climate impact of heating without scrapping tens of millions of gas boilers?

The answer, of course, is to make green gas from grass. Building green gas mills, as we’re doing right now near Reading, is the quickest and cheapest way to replace fossil gas use in Britain.

What’s more, our green gas mills are fed with species-rich herbal leys, which help boost biodiversity, lock in carbon and improve soil quality on the marginal grasslands or replaced monoculture grasslands that we plan to use.

What is a species-rich herbal ley?

Herbal leys are grasslands made up of legumes, herbs and grasses. The exact species mixtures will depend on local soil conditions but we’ll typically be planting various types of clovers, sainfoin, chicory, lucence, birdsfoot trefoil, along with grasses such as Timothy, ryegrass and cocksfoot.

How will green gas mills boost biodiversity?

Biodiversity has declined dramatically across much of lowland Britain because of the increase in both intensive arable systems and monoculture grasslands.

Our species-rich herbal leys will both feed our green gas mills and support a much higher density of insects, bees and other pollinators than any intensively managed grassland or arable field, thanks to the lack of artificial pesticides and agrochemicals.

8

Research demonstrates value ‘injurious weeds’ can bring to both pollinators and biodiversity

A new study demonstrates that weeds are far more valuable in supporting biodiversity than we give them credit for.

Dr Nicholas Balfour and Professor Francis Ratnieks at the University of Sussex compared the biodiversity value of plants classified as ‘injurious weeds’ with those stipulated by the Department for Environment, Food and Rural Affairs (DEFRA) for pollinator targeted agri-environmental options, such as red clover and wild marjoram.

Their findings, published in the Journal of Applied Ecology, show that the abundance and diversity of pollinators visiting weed species are far higher than DEFRA recommended plants.

In the UK, five species of native wildflowers are classified as “injurious” in the 1959 Weeds Act. Three of them are frequently visited by many species of bees and other insects – ragwort (Jacobaea vulgaris) and two thistles (Cirsium arvense, C. vulgare). The other two are docks (Rumex crispus and R. obtusifolius), whose flowers are mainly wind-pollinated.

Dr Balfour and Professor Ratnieks conducted a field study in East Sussex where they quantified and identified insects visiting three of these species – the flowers of ragwort, thistles, and other wildflowers, including those recommended by DEFRA – growing in six pasture or ex-pasture sites.

Their results, which found that pollinators were visiting weed species in higher numbers than DEFRA recommended plants, were mirrored by a subsequent analysis of scientific literature.

In the Database of Pollinator Interactions, four times as many pollinator species and five times more conservation-listed species have been recorded visiting the three insect-pollinated weeds. Of the 387 plant species analysed in the database, in terms of pollinator species recorded, the weeds were ranked 4th (C. arvense), 6th (J. vulgaris), and 13th (C. vulgare).

9

Biodiversity loss to become 'material risk' for global businesses by 2024

Businesses may soon struggle to access financial services as requirements to protect biodiversity raise significant liability and litigation risks over the next two years, Clyde & Co has warned.

In a new report, the global law firm highlights how a plethora of wide-ranging value chain due diligence standards are coming to the fore, and will be in place in many jurisdictions by 2024.

The Taskforce on Nature-related Financial Disclosures (TNFD) framework will be trialled this year, and investor pressure will likely lead to adoption, with mandatory reporting regimes to follow.

Further action will take place at the Convention on Biological Diversity’s COP15 in China later this year where countries are expected to agree to '30x30' targets to preserve and protect 30% of land and sea by 2030.

Concerted policy action is expected to increase law and regulation in this area, which is set to become the next big environmental, social and governance (ESG) challenge for global businesses.

Clyde & Co partner Nigel Brook said: “Businesses will see new physical, regulatory, market and reputational risks – all of which are compounded by the hyper-connectivity of global value chains.

The consequences of not managing these risks will play out in terms of inability to attract finance, do deals and ultimately in damage to market value.

“Insurers will face scrutiny of the biodiversity risks and impacts of their underwriting and investments. Biodiversity loss will lead to write-downs and write-offs in impacted business lines, increasing claims and rising premiums.”

10

Putting biodiversity at the heart of business agenda

Our sustainability journey began in 1993 with the realisation that responsible commercial forest management and deforestation required collective action. This led B&Q to work with WWF, the Rainforest Alliance and others to found the Forestry Stewardship Council (FSC) to improve forestry practices and provide greater confidence to our consumers.

In 2011, our British banner, B&Q, first achieved its goal of only using timber from responsible sources. Today, Kingfisher plc is an international home improvement company with 11 retail banners operating across eight countries. Each banner and the products we sell bring their own set of supply chain sustainability challenges which we’re working to solve.

At the end of 2020, we committed to becoming ‘Forest Positive’ and creating more forests than we use by the end of 2025.

Tackling climate change, eliminating deforestation and enhancing the livelihoods of communities in our supply chains are all key to our commitment to leading our industry in responsible business practices, which is at the heart of our strategic plan, “Powered by Kingfisher

Forest communities play a vital role in protecting critical forest landscapes. If we are to restore forests, halt deforestation and forest degradation, we need to foster partnerships that create long-lasting change.

We are a founding member of the Rainforest Alliance Forest Allies initiative, which is equally focused on protecting natural forests and partnering with communities to build strong local economies.

Forest Allies recognises indigenous peoples and local communities as effective forest stewards and future business partners. Working beyond our individual supply chains on projects in Indonesia, Cameroon, Peru, Guatemala and Colombia, the programme will restore ecosystems across over 300,000 hectares and benefit the livelihoods of over 7,500 local people.

2022.5

1

National Highways and The Wildlife Trusts announce biodiversity boost across England

National Highways and The Wildlife Trusts have joined forces to launch a new £6 million Network for Nature programme that will improve habitats across England, benefitting people, nature and wildlife.

The projects will help create, restore and connect places for wildflowers, trees and wildlife, where the environment has been impacted by activities from previous road building. Natural solutions such as wetlands and reedbeds will help filter polluted run-off from roads.

Wild areas which have been fragmented by highways will be improved and restored for nature, with one scheme piloting dormouse bridges alongside the M5 in Somerset, reconnecting isolated populations of critically rare hazel dormice and helping them spread into the wider landscape.

Another will see new wetland created in the Lugg Valley in Herefordshire, becoming a stepping stone for wildlife between two of the most important sites for wetland birds in the county, reducing pollution entering the River Lugg and creating sustainable drainage pools close to the A49.

Two areas of nationally important chalkland in Hampshire are also set for restoration, which could offer a boost to one of Britain’s rarest butterflies, the Duke of Burgundy butterfly. The pair of chalk downland sites near Winchester, which cover 65 hectares either side of the M3 motorway, will help address the fragmentation and loss of chalk grassland caused by the construction of the motorway over 20 years ago.

The projects will help create, restore and connect places for wildflowers, trees and wildlife, where the environment has been impacted by activities from previous road building. Natural solutions such as wetlands and reedbeds will help filter polluted run-off from roads.

2

50 projects receive up to £100,000 to boost investment in nature

Projects include rewetting lowland peat to grow plant fibre material to use as padding for clothes.

Innovative projects to restore and rewet peatlands, create green urban spaces and improve flood resilience are among 50 schemes to benefit from the final round of a pioneering fund to drive private investment in nature and tackle climate change.

The second round of the Natural Environment Investment Readiness Fund (NEIRF), announced today by Defra and the Environment Agency, will provide grants of up to £100,000 to environmental groups, local authorities, businesses and other organisations to help them develop nature projects to a point where they can attract private investment.

The funding will help to develop projects so they can demonstrate a return on that investment by capturing the value of carbon, water quality, biodiversity and other benefits provided by natural assets such as woodlands, peatlands and rivers – with revenue generated through the sale of carbon storage, improvements in biodiversity, natural flood management benefits and reduced water treatment costs.

Examples of projects receiving funding in round two include:

Rewetting lowland peat near Doncaster to grow plant fibre material to use as padding for clothes. This project will attract investors by showing how revenue can be generated from the sale of biodiversity credits and carbon units, as a means of compensating for biodiversity loss and carbon emissions, as well as through the creation of sustainable textiles.

Habitat enhancement, such as tree planting, brownfield land regeneration and nature recovery, to address ecological and environmental degradation in the Liverpool City Region with revenue generated through selling biodiversity units to housing developers that need to compensate and provide a net gain for any biodiversity loss resulting from the development.

3

Norfolk biodiversity audit lists coast's species

A "ground-breaking" biodiversity audit of more than 10,000 species in north Norfolk habitats has been created to help any decisions about their future.

The project by the University of East Anglia (UEA) looked at a 105-mile (169km) stretch of coastline.

It is hoped the information will help land managers and farmers protect and enhance habitats for wildlife.

Dr James Gilroy, ecology lecturer, said the key was to give the "huge database" to those who "really need it".

Among the variety of habitats are some of the largest areas of salt marsh in the country, sand dunes, freshwater grazing marshes and wetlands, and is home to 1,200 priority species.

Although much of nature conservation is evidence-based, typically this is restricted to large and charismatic species - particularly birds, the UEA said.

More than one million biological records of more than 10,000 species - including plants and invertebrates - were combined with other ecological datasets and knowledge from natural history experts and managers.

Prof Paul Dolman, part of the UEA team, said the work had been "ground-breaking".

"This is the first time anyone has fully quantified the important wildlife of this amazing landscape and identified what it needs," he added.

"Crucially, by working with land managers throughout the study we were able to develop a plan of how to expand and enhance nature along the coast.

"Relatively small changes such as changing the management of drainage ditches, or more dramatic ones, like transforming low-lying arable to new grazing marsh and areas at risk of saltwater flooding into new salt marsh, will provide more space for many species, even as coastal habitats are squeezed by sea level rises."

4

COP15: Time to tackle the nature emergency

You’ve probably heard all about COP26, but what about COP15?

COP15 is the long-awaited UN biodiversity summit. It’s the world’s attempt to tackle the ‘nature emergency’ by agreeing action to reverse the major loss of biodiversity caused by humans.

After 2 years of delays due to the coronavirus pandemic, the summit is anticipated to take place in the third quarter of 2022 in Kunming, China.

This article discusses the state of biodiversity and how COP15 could make a difference globally and in Wales.

You’ve probably heard all about COP26, but what about COP15?

COP15 is the long-awaited UN biodiversity summit. It’s the world’s attempt to tackle the ‘nature emergency’ by agreeing action to reverse the major loss of biodiversity caused by humans.

After 2 years of delays due to the coronavirus pandemic, the summit is anticipated to take place in the third quarter of 2022 in Kunming, China.

This article discusses the state of biodiversity and how COP15 could make a difference globally and in Wales.

We are entering the sixth mass extinction event

Global biodiversity levels have always undergone changes. Scientists differentiate six mass extinctions since life on Earth began, interspersed with periods of thriving biodiversity.

Each mass extinction is thought to have resulted in at least 75% of all species becoming extinct. Fossils found on Welsh coasts are evidence of species that became extinct hundreds of millions of years ago, replaced by others through evolution.

Scientists say we are entering the sixth mass extinction event, and the first linked to human activity. Research suggests the average rate of vertebrate species loss over the last century is up to 100 times higher than pre-human extinction rates.

5

We need decisive action on biodiversity loss, and the UK must lead the way

You can’t begin to understand the times we live in without first recognising the scale and pace of ecological destruction that has taken place over the past 50 years. Since 1970, the world has witnessed an average 68% collapse in mammal, bird, fish, reptile and amphibian populations. In that same period, 17% of the Amazon has been lost, meaning that the rainforest today emits more CO2 than it absorbs. Meanwhile, our oceans – the world’s largest carbon sink – are being pushed to the brink by overfishing and soaring temperatures.

This isn’t just a problem for the nature lovers amongst us or for the good people at the BBC’s natural history unit. It could spell catastrophe for all of us. From Covid to the wildfires devastating the Arctic, the great crises of our age are all directly linked to our degradation of the natural world. Just as the window to cut emissions is rapidly disappearing, so too is the time we have left to stop this wanton destruction.

It’s against this backdrop that world leaders will meet in Kunming, China, later this year for the COP15 conference on biological diversity. This is the international community’s best and perhaps last chance to agree binding and ambitious targets not just to halt, but to actively reverse ecological and biodiversity loss. And while COP15 may lack the brand awareness of last year’s COP26 conference on climate change, its outcomes are every bit as important for our future.

6

Biodiversity: opportunity, risk and the LGPS

Is biodiversity loss a bigger issue than climate change for local government pension funds and their pools? Room151 convened a roundtable of practitioners and investment managers to discuss what role biodiversity will play in investment decision-making over the next decade.

In many respects biodiversity loss is the unloved sibling of climate change. The need to combat global warming has been embraced by a growing number of countries and companies. The target set at COP 21 in Paris in 2015 – to pursue efforts to limit global temperature rises to 1.5°C above pre-industrial times – is well understood, and there are requirements under the Task Force on Climate-Related Financial Disclosures (TCFD) and an array of net-zero targets.

But the same attention has not been paid to biodiversity loss, and the impact here could be much greater. There is a popular meme that shows the major issues impacting the world as a series of waves. Covid 19, which has occupied our attention for the past two years, is the smallest wave, followed by larger waves for recession and then climate change. However, the biggest wave is for biodiversity collapse.

So are we waking up late to the biodiversity emergency? Some figures might put this into perspective:

More than half of global GDP depends significantly on nature

Biodiversity loss is costing the global economy 10% of its output each year

Humanity has caused the loss of 83% of wild mammals and half of all plants

60% of all medicines are based on natural organisms

7

Why Birds Eye is choosing now to mount a biodiversity campaign

It’s estimated the global population will reach almost 10 billion by 2050. Not only is this a staggering number in itself, but it also serves as a timely reminder of some of the challenges we are yet to face when it comes to sourcing our food sustainably. In order for the planet to feed this many people, it is crucial our industry finds processes and approaches to food production that don’t destroy biological diversity, but instead actively replenish it.

Biodiversity provides us with clean air, fresh water, good quality soil and crop pollination. It’s key in the fight against climate change, so there has never been a more vital time to focus on how we source and farm our food, and put processes in place to protect biodiversity for the future. Research suggests agriculture is currently responsible for 60% of global biodiversity loss, as a result of the emissions it produces. This may be a painful pill for the industry to swallow, but one that further reinforces why we must do more to defend our planet’s numerous ecosystems.

As highlighted in The Grocer several weeks ago, the health of soil across the globe is deteriorating at a steady rate of knots. According to the UN Food and Agriculture Organization (FAO), soil holds over a quarter of the planet’s biodiversity – yet biodiversity loss currently stands at an estimated global level of 100 to 1,000 times higher than the naturally occurring background extinction rate. Put simply: time is running out. We must take action now.

8

SECURING A SHARED FUTURE FOR ALL LIFE ON EARTH

Biodiversity represents the tremendous variety of life on Earth on which we all depend. But human influence, including from land use changes, overexploitation and climate change means the biodiversity on which we all depend is under threat. More than one million animal and plant species are now at risk of extinction.

Despite important progress that has been made to value, conserve and restore biodiversity, we have collectively failed to reverse a global decline.

Today, 22 May, is marked around the world as the International Day for Biological Diversity. Spearheaded by the Convention on Biological Diversity (CBD) – the major UN treaty that works for all aspects of biodiversity – this day is a chance to celebrate the kaleidoscope of nature that is all around us.

Later this year, the CBD will oversee the approval of new plan for nature: the global biodiversity framework. Its vital ambitions will include global targets to conserve, restore and sustainably use biodiversity. Today is an important opportunity to reflect on the crucial importance of these new targets. It is also a chance to explore the day’s theme – “building a shared future for all life” – which looks to highlight how biodiversity underpins sustainable human development.

International Biodiversity Day is a chance to celebrate. To celebrate the amazing diversity of life with which we share this planet. And to celebrate the many contributions that nature provides to people. But the day is also a chance to reflect. To reflect on the biodiversity crisis. The loss and degradation of life around us. And on the urgency and scale of the response needed to tackle this loss and ensure a nature-positive world.

9

National Tree Map helps improve biodiversity in London

A project targeting biodiversity gain and climate change resilience in Bloomsbury, London is turning to data captured by Bluesky International’s National Tree Map to ensure proposals are built on evidence-based solutions

The Green Infrastructure Consultancy has been commissioned to provide a strategy for Wild Bloomsbury, a project aimed at increasing both the quantity and quality of multifunctional green infrastructure in Bloomsbury for the benefit of people and wildlife.

The Green Infrastructure Consultancy is a UK-based company advising private, government, institutional and NGO clients on urban green infrastructure policy, strategy, planning, design, installation and maintenance.

National Tree Map is the most accurate tree map available

Data from Bluesky’s National Tree Map (NTM) was chosen to support the Green Infrastructure Consultancy report as it could provide evidence of existing green cover over the required area. The NTM is a unique dataset and is the most accurate tree map available covering all of England, Wales and the Republic of Ireland; Scotland will be coming online soon. It provides data of all tree canopy coverage and heights for trees 3m and taller and is updated and maintained on a rolling three-year update programme.

The NTM has been created using Bluesky’s high-quality, high-resolution aerial photography and height datasets coupled with innovative processing techniques. It can provide valuable data to numerous organisations for a variety of projects, but in recent months Bluesky has seen an increasing number of companies apply the NTM to climate change reduction initiatives.

10

New funding to protect biodiversity in UK overseas territories

Threatened species will benefit from funding to tackle invasive species and strengthen climate change mitigation.

Threatened species across the UK Overseas Territories (UKOTs) are set to benefit from £6.4 million government funding announced today (Monday 23 May).

Green turtles and the West Indian whistling duck are just some of the species that will be boosted by a share of the Darwin Plus initiative. A total of 20 environmental recovery projects will be supported to deliver marine conservation, research into threatened species, and improve resilience to climate change.

The UK Overseas Territories are unique and globally significant nature hotspots. Made up of thousands of small islands, vast oceans and dense rainforests, these areas are thriving with unique wildlife and contain 94% of the UK’s biodiversity.

Since 2012, the government has contributed more than £38 million towards 180 projects in the UK Overseas Territories through Darwin Plus, and has committed £30 million over the next three years to support even more environmental projects in these areas.

Funding through Darwin Plus and the Darwin initiative will help to deliver the commitments set out in the Environment Act to halt and reverse the decline of biodiversity and improve species abundance by 10% by 2042.

From rare sea birds to threatened coral reefs and plants, the Darwin Plus initiative is instrumental in protecting and restoring our precious natural environment across the Overseas Territories.

This investment will enhance our global capability to respond to climate change, address biodiversity decline and help safeguard the future of these unique habitats for the next generation.

**Climate change temporal pattern news**

2011.1.1-2013.12.31

1.

Climate change is on ice: UN scientists reveal the world's barely got any hotter in the last 15 years - but say they are now 95% certain man is to blame for global warming

UN scientists said today they are '95 per cent' certain that climate change is man made, but still could not explain why the world has barely got any hotter in the last 15 years.

The United Nations’ Intergovernmental Panel on Climate Change (IPCC) said that sea levels have risen by 19cm since 1901 and are expected to rise a further 26-82cm by the end of the century.

It added that concentrations of CO2 and other greenhouse gases in the atmosphere have increased to levels that are unprecedented in at least 800,000 years.

But the landmark report conceded that world temperatures have barely risen in the past 15 years, despite growing amounts of greenhouse gases being pumped into the atmosphere.

Temperature rises have dropped from 0.12°C per decade since 1951 to just 0.05°C per decade since 1998.

This slowdown has been seized upon by climate sceptics who claim carbon dioxide is not as damaging as has been suggested.

IPCC scientists, however, believe the pause is temporary and a return to 'substantial warming' is expected in coming decades.

The report only made a brief mention of the issue, stressing that short-term records are sensitive to natural variability.

In the briefing this morning, Thomas Stocker, co-chair of working group, said that the current warming hiatus could not be predicted because ‘there are not sufficient observations of the uptake of heat, particularly into the deep ocean.’

2.

Global warming pause 'central' to IPCC climate report

The Intergovernmental Panel on Climate Change (IPCC) is meeting in Sweden to thresh out a critical report on global warming.

Scientists will underline, with greater certainty than ever, the role of human activities in rising temperatures.

But many governments are demanding a clearer explanation of the slowdown in temperature increases since 1998.

One participant told BBC News that this pause will be a "central piece" of the summary.

Researchers from all over the world work with the IPCC to pore over thousands of peer-reviewed studies and produce a summary representing the current state of climate science.

Its previous report in 2007 was instrumental in helping the panel share the Nobel Peace Prize that year.

A new Summary for Policymakers on the physical sciences, the first of three parts that make up a report to be released over the next 12 months, will be published in Stockholm on Friday.

It will focus on the science underlying changes in temperature in the atmosphere, the oceans and at the poles.

New estimates will be given for the scale of global warming and its impact on sea levels, glaciers and ice sheets.

In its last report in 2007, the IPCC stated that "warming of the climate system is unequivocal" and that "most of the observed increase in global average temperatures since the mid-20th Century is very likely due to the observed increase in anthropogenic greenhouse gas concentrations" - in other words, humans burning fossil fuels.

In the latest draft summary, seen by the BBC, the level of scientific certainty has increased.

The panel states that it is 95% certain that the "human influence on climate caused more than half the observed increase in global average surface temperatures from 1951-2010."

3.

Climate change 'hiatus': Scientists seek to qualify evidence of apparent global warming slowdown

Authors of a landmark UN report on climate change remain adamant that humans are heating up the planet by burning fossil fuels and cutting down CO2-absorbing forests, despite data from the report suggesting a purported slowdown in global warming over the past 15 years.

The Intergovernmental Panel on Climate Change’s (IPCC) full 127-page document - due for release in Stockholm next week - is expected to suggest the link between human activity and global warming is clearer than ever, regardless of the recent lower rate of warming.

In a draft leaked in June, the IPCC said that while the rate of warming between 1998 and 2012 was about half the average rate since 1951, the globe is still heating up in the long term.

The leaked report qualified the apparent slowdown by citing natural variables in the climate system, the cooling effects from volcanic eruptions and the fact that an uncommonly hot year was picked as the starting point for the 15-year chart.

“Barring a major volcanic eruption, most 15-year global mean surface temperature trends in the near-term future will be larger than during 1998-2012,” a technical summary from the report said.

“Fifteen-year-long hiatus periods are common,” it added.

The Associated Press says it has obtained comments made to the IPCC by several governments concerned with how to tackle the apparently anomalous data.

Germany called for the 15 year slowdown to be dismissed as a blip, saying climate change needed to be measured in decades and centuries, rather than smaller intervals.

4.

Climate change 'driving spread of crop pests'

Climate change is helping pests and diseases that attack crops to spread around the world, a study suggests.

Researchers from the universities of Exeter and Oxford have found crop pests are moving at an average of two miles (3km) a year.

The team said they were heading towards the north and south poles, and were establishing in areas that were once too cold for them to live in.

The research is published in the journal Nature Climate Change.

Currently, it is estimated that between 10% and 16% of the world's crops are lost to disease outbreaks. The researchers warn that rising global temperatures could make the problem worse.

Dr Dan Bebber, the lead author of the study from the University of Exeter, said: "Global food security is one of the major challenges we are going to face over the next few decades.

"We really don't want to be losing any more of our crops than is absolutely necessary to pests and pathogens."

To investigate the problem, the researchers looked at the records of 612 crop pests and pathogens from around the world that had been collected over the past 50 years.

These included fungi, such as wheat rust, which is devastating harvests in Africa, the Middle East and Asia; insects like the mountain pine beetle that is destroying trees in the US; as well as bacteria, viruses and microscopic nematode worms.

Each organism's distribution was different - some butterflies and insects were shifting quickly, at about 12 miles (20km) a year; other bacterium species had hardly moved. On average, however, the pests had been spreading by two miles each year since 1960.

5.

What climate change? Fewer people than EVER believe the world is really warming up

A report from the UK Energy Research Centre also shows the number of those who resolutely do not believe in climate change has more than quadrupled since 2005.

The Government funded report shows 19 per cent of people are climate change disbelievers - up from just four per cent in 2005 - while nine per cent did not know.

The report comes as climate change scientists working on a landmark UN report on climate change are struggling to explain why global warming appears to have slowed down in the past 15 years even though greenhouse gas emissions keep rising.

Dr. Roy Spencer, a former NASA scientist and author of Climate Confusion, argues in his influential blog the UN report shows scientists are being forced to "recognise reality".

He said: "We are now at the point in the age of global warming hysteria where the IPCC global warming theory has crashed into the hard reality of observations."

Green Party leader Natalie Bennett blamed the Government for the increase in climate change doubters.

She said: "When the government is so clearly failing to act on climate change, or take seriously its obligations under the Climate Change Act, it's not surprising that the level of doubt about climate change has risen.

"Of course, however, the 72 per cent of the public who acknowledge the climate is changing are backed overwhelmingly by the scientific evidence.

"The US National Oceanic and Atmospheric Administration concluded that half of last year's extreme weather events around the world were in part caused by climate change.

6.

Climate change models may not have been accurate after all as study finds most widely overestimated global warming

Predictions on global warming trends may not be as accurate as at first their studies suggest.

A new study in the journal Nature Climate Change looked at 117 climate predictions made in the 1990's to the actual amount of warming.

Out of 117 predictions, only three were accurate. The other 114 overestimated the amount by which the Earth's temperature rose.

The predictions were roughly twice the amount of global warming than had actually occurred.

Some scientists have suggested that such results require a major overhaul in climate modelling and say the study shows that climate modellers need to go back to the drawing board.

‘It's a real problem ... it shows that there really is something that needs to be fixed in the climate models,’ climate scientist John Christy, a professor at the University of Alabama in Huntsville, told FoxNews.com.

‘I looked at 73 climate models going back to 1979 and every single one predicted more warming than happened in the real world.’

But other scientists are less concerned with the gross overestimation of the future of climate and global temperatures.

This is neither surprising nor particularly troubling to me as a climate scientist,’ Melanie Fitzpatrick, a climate scientist with the Union of Concerned Scientists, told FoxNews.com. ‘The work of our community is constantly to refine our understanding of the climate system and improve models based on that,’ she says.

She believed that over the long term, climate models will be accurate but there also too many variations in climate to expect models to be accurate over twenty years.

7

Changes will be coming soon' Climate change to devastate ecosystems by 2047

CLIMATE CHANGE could be destroying ecosystems and economies within a generation, scientists have warned.

Researches have revealed that current levels of greenhouse gas emissions could lead to a transformation of the world's climate as soon as 2047.

However in the world's most vulnerable regions the impact could come much sooner.

Areas within the Tropics could be hit by catastrophic changes in both temperature and rainfall in as little as TWO decades.

The changes could leave many species on the brink of extinction.

The Tropics house a large percentage of the Earth's population and provide significant quantities of the world's food supplies so the resulting damage could be devastating.

The results shocked us," said lead researcher Dr Camilo Mora, from the University of Hawaii.

"Regardless of the scenario, changes will be coming soon.

"Within my generation, whatever climate we are used to will be a thing of the past."

The scientists used 39 climate simulation models to look at the likely effects of global warming in different locations around the world.

Projections over the next 100 years showed when the climate in any given location was expected to shift outside these limits.

These projections have led to the prediction that Earth will experience a dramatically different climate by 2047.

The tropics will be worst affected and given that they are the poorest regions in the world they are also the least able to cope with the effects of global warming.

Dr Ryan Longmam emphasised this point, saying: "Our result suggest that countries first impacted by unprecedented climates are the ones with the least capacity to respond."

8.

Coastal wildlife species 'at risk due to climate change'

UK coastal species such as puffins and little terns could be "seriously affected" by erosion and climate change, the National Trust has said.

It has published a list of six species in a bid to highlight how wildlife might fare over the next few decades.

While triggerfish numbers are rising, species such as puffins are at risk due to factors like unpredictable weather.

The trust said climate change was set to cause more of a "bust than boom".

According to the organisation, the UK coastline is already being affected by rising sea levels, with projections suggesting seas could be half a metre higher than at present by 2100.

Matthew Oates, wildlife specialist for the National Trust, said: "Climate change could change the face of our coastal flora and fauna. With rising sea levels, our rich mud flats could simply disappear.

"Wildlife which relies on the gradual erosion of soft rock cliffs or lives on loose sand and shingle habitats could be caught out by an increasingly mobile landscape as a result of extreme weather.

"We are likely to see the boom and bust of more specialist plants and animals, as they suffer from increased flooding, salt deposition or drought stress. Unfortunately there may be more bust than boom."

The trust published its list of "six canaries in the mine" - a reference to the birds sent down mines to reveal the presence of dangerous gases - in a bid to show how plants, animals and humans would have to live alongside "an increasing rate of environmental change".

It said species such as little terns, which nest in colonies on sand and shingle beaches just above the high tide line, were vulnerable to exceptional high tides and summer storms.

9.

How Greenland may become greener: Climate change 'could accelerate the spread of forests and kill arctic animals'

Aarhus University research predicts forests similar to those found in Alaska and western Canada could thrive in parts of Greenland by the year 2100

The scientists said that if temperatures rise as predicted, 44 species of North American and European trees would be able to thrive in Greenland

The transformation would alter Greenland’s ecosystem, leading to the loss of Arctic animals and plants.

Climate change could bring about the greening of Greenland by the end of the century, scientists predict.

Today only four indigenous tree species grow on the island, confined to small areas in the south and three quarters of the world's most most sparsely populated country is covered by a barren ice sheet.

But by the year 2100 swathes of verdant forest could be covering much of its land surface, potentially changing the landscape and making life difficult for arctic animals, according to experts.

'Greenland has...the potential to become a lot greener,' said lead scientist Professor Jens-Christian Svenning, from Aarhus University in Denmark.

'Forest like the coastal coniferous forests in today’s Alaska and western Canada will be able to thrive in fairly large parts of Greenland, for example, with trees like sitka spruce and lodgepole pine.'

He said: 'It will provide new opportunities for the Greenlanders.'

The research showed that with expected levels of warming, a majority of 44 species of North American and European trees and bushes would be able to thrive in Greenland.

Many species could already grow in small numbers in Greenland today, according to the analysis published in the journal Philosophical Transactions of the Royal Society B.

10.

John Kerry says there is 'irrefutable and alarming evidence' that climate change is real

US Secretary of State John Kerry made the remarks Monday during an address to the Pacific Islands Forum

Mr Kerry argued that climate change is alarming and will only get worse if no action is taken to cut emissions

European and PIF leaders also spoke, calling on the rest of the world to act before it is too late

US Secretary of State John Kerry is concerned about the threat posed by global warming.

Speaking Monday via satellite to climate experts gathered for this week’s Pacific Islands Forum (PIF) in the Marshall Islands, US Secretary of State John Kerry asserted that climate change needs to be taken more seriously and that action can be taken to stem the tide of consequence.

The Marshall Islands, at no more than three feet above sea level, are especially at risk of rising seas, one of the many effects of global warming.

The science is clear. It is irrefutable and it is alarming,’ Mr Kerry said via satellite from Washington, DC. ‘If we continue down our current path, the impacts of climate change will only get worse.’

Saying ‘I stand with you in the fight against climate change,’ Mr Kerry advocated strong, immediate action to limit the threats posed to infrastructure, health, the world economy and even the viability of island nations.

Mr Kerry also said it will take more than one nation to make a difference.

‘If we act together, there is still time to prevent some of the worst impacts of climate change,’ said Mr Kerry, adding that ‘the people of the Pacific Islands know as well as anyone that we also need to prepare communities for the impacts that are already being felt.’

2014.1.1-2016.12.31

1.

COP21: What does the Paris climate agreement mean for me?

As the euphoria of delegates at the UN climate talks in Paris fades, it is time to get down to the business of saving the planet and ask what does it mean for me?

Over the past two weeks, almost every nation on the planet has sent a team of negotiators to Paris to pore over page after page of nuanced jargon peppered with what seemed like a world record attempt for the most square brackets in a document.

But these brackets did matter. In the tense talks at a conference centre in north Paris, semantics was king.

Negotiators inhabited a world were "shall" would result in something becoming legally binding and "should" actually meant voluntary, as BBC environment analyst Roger Harrabin explained here.

The fortnight kicked off with more than 150 world leaders, including Presidents Obama, Putin and Xi, descending on Paris to tell delegates that climate change was the most important issue facing us in the 21st Century.

Whether that was welcome support or unnecessary pressures it meant negotiators got down to business, often working through the night.

On Saturday evening - to claps, cheers and tears - a new landmark deal was born.

It was agreed by 195 nations. They will attempt to cut greenhouse gas emissions to a level that will limit the global average temperature to a rise "well below" 2C (3.6F) compared to pre-industrial levels - a level of warming deemed to be the point when dangerous climate change could threaten life on Earth.

2.

COP21: Rallies call for Paris climate change action

Hundreds of thousands of people have marched worldwide to demand action to stop climate change, the day before a UN summit starts in Paris.

One campaign group says more than 570,000 protesters took part in marches on all the main continents.

Activists want action at the Paris talks to limit the rise in the average global temperature to 2C (3.6F) above pre-industrial levels.

In Paris itself, more than 200 demonstrators were arrested after clashes.

The day's events

In pictures: How eco-activists are mocking brands and leaders

Why do two degrees matter?

Earlier, a human chain was formed by hundreds linking arms in the French capital along the route of a march that was called off after the 13 November attacks that killed 130 people.

A gap in the chain was left in front of the Bataclan concert hall, where 89 people were killed.

Hundreds of pairs of shoes were left on Place de la Republique to remember those left frustrated in their plans to march.

Among them were a pair donated by Pope Francis, who has called for urgent action on climate change.

Elsewhere across the world:

an estimated 50,000 people took part in a march in central London, where opposition leader Jeremy Corbyn addressed crowds

some of the earliest protests in the day took place in the Marshall Islands, a US territory in the Pacific Ocean threatened by rising seas

in Kenya, a march took place across the equator

a small group took part in a march across a glacier in the south of Chile

the mayor of Sydney in Australia tweeted to say that there were "at least 45,000" demonstrators, making it the biggest ever such march in the city

3.

Paris climate change talks: Lord Stern calls on rich countries to help poor nations cope with global warming

Lord Stern, the world’s most authoritative climate economist, is calling on rich countries to do everything they can to help poorer nations weather the storm of global warming – warning that a failure would escalate poverty, migration and conflict.

In an interview with The Independent ahead of the United Nations climate change summit in Paris, Lord Stern said the conference represented a crucial opportunity to reduce the huge inequality at the heart of global warming.

The huge resentment among poor countries over how they are suffering could potentially scupper a global agreement to tackle it, he explained.

“Equality is a big issue. The rich got rich on high-carbon growth and it’s the poor people of the world – whether they be poor people in rich countries or poor people in poor countries – who suffer earliest and most,” Lord Stein said.

The negotiations in Paris will largely concentrate on how much money the rich nations pay to the poorer ones to help them adapt to the effects of global warming – such as increased hurricanes and droughts – and to help finance the transition from fossil fuels to green energy.

The issue is fraught as developed nations with financial problems of their own baulk at channelling hundreds of billions of pounds into developing countries – which, in turn, worry that the amounts involved, though huge, are not nearly enough.

Lord Stern was speaking as new research underlined how extraordinarily expensive global warming will be for developing countries – and how much less it would cost if the world redoubled its efforts to curb it.

4.

Almost all climate scientists agree: climate change is real!

New analysis of the vast body of research shows 97% of climate scientists agree that human activities are fuelling climate change, writes Tim Radford. But thanks to aggressive attempts to convince us otherwise, only 12% of people in the US are aware of this high level of agreement.

In a rational world, it would have been an entirely unnecessary research project. But sadly not in Australia and the USA, hotbeds of climate change denialism.

US and Australian scientists have found an extraordinary degree of scientific concensus on the reality of climate change: 97% of climate scientists agree that climate change is happening - and that it is caused by humans.

Since the governments of 195 nations have de facto already accepted this, and collectively vowed at the UN climate conference in Paris last December to reduce greenhouse gas emissions from fossil fuel combustion and contain global warming if possible to a rise of 1.5C, it might be expected that citizens would need no further convincing. But surveys shows that they do - and particularly in the US.

So John Cook, climate communication fellow at the University of Queensland's Global Change Institute in Australia, and colleagues from the US, Canada and Europe report in Environmental Research Letters that they have examined all the research yet again.

And they have come up yet again with a conclusion that supports all previous research: that 96%-98% of climate scientists agree that climate change is caused by humans.

5.

Trump: The best thing ever for climate change?

Since the US election result, there has been consternation among climate campaigners and many environment ministers, especially those attending the annual Conference of the Parties (COP) in Marrakech.

The fear, and fury of the green response to Donald Trump is understandable.

Here's a man who has promised to "cancel" the Paris Climate Agreement, and who tweeted that climate change was a hoax invented by the Chinese - though he did seem to dispute this accusation during a presidential debate.

However his admiration for the coal industry and his determination to revive it are not in doubt. None of that sits well with those who believe that climate change is the greatest threat facing the planet.

But there is another school of thought that says that a Trump presidency could actually aid the fight against climate change.

The Paris agreement became part of international law in super-quick time, mainly because countries were aware of Mr Trump's threats to wreck the deal.

"His negative impact is as overblown as his haircut," said Joe Ware from Christian Aid.

"He's already generated the fastest coming into force of any treaty with the ratification of the Paris treaty in record time."

If Mr Trump decides to withdraw the US from the Paris Climate Agreement it would be a blow to the deal - but not the terminal one that many fear.

China, India and all the major emitters have reiterated their belief at this conference that the deal is irreversible and they will honour their commitments. Mr Trump appears to have forged a spirit of unity among all parties at the conference, even the World Coal Association (WCA).

6.

Is Japan playing hunger games with climate change?

The impact of rising temperatures on the world's food supplies is a key issue for climate experts meeting in Japan. But food security is not just about developing countries. As environment correspondent Matt McGrath reports, a changing climate is one of a number of issues pushing Japan towards a food crisis.

In the historic Ueno Park in the middle of Tokyo, seemingly normal people are earnestly staring at trees.

They are waiting for the first signs of cherry blossom, a spring event of special importance to the Japanese, who will turn out in their thousands to sit under the scented branches and drink themselves squiffy in a custom called Hanami.

But bear hugs from drunken businessmen are a minor threat to the cherries compared to a warming world, according to research.

"There are already reports that the cherry trees are not doing as well as they usually do because the climate is changing," said long time Tokyo resident Martin Frid, who works on food safety issues for the Consumer's Union of Japan.

"They are blossoming at different times, there is now more irregularity."

Because of their cultural significance, the appearance of the blossoms has been recorded in some parts of Japan for over a thousand years.

These records have enabled scientists to work out the impact of global warming on the trees: In recent years they've been blossoming about four days earlier than the long term average.

Experts fear that under some warming scenarios, it could be a fortnight earlier by the end of this century.

While Japan could cope with this change, other impacts of rising temperatures may impose more significant costs.

7.

Climate change: 'Monumental' deal to cut HFCs, fastest growing greenhouse gases

More than 150 countries have reached a deal described as "monumental" to phase out gases that are making global warming worse.

Hydrofluorocarbons (HFCs) are widely used in fridges, air conditioning and aerosol sprays.

Delegates meeting in Rwanda accepted a complex amendment to the Montreal Protocol that will see richer countries cut back their HFC use from 2019.

But some critics say the compromise may have less impact than expected.

US Secretary of State John Kerry, who helped forge the deal in a series of meetings in the Rwandan capital, said it was a major victory for the Earth.

"It's a monumental step forward, that addresses the needs of individual nations but it will give us the opportunity to reduce the warming of the planet by an entire half a degree centigrade," he told BBC News.

The new agreement will see three separate pathways for different countries.

Richer economies like the European Union, the US and others will start to limit their use of HFCs within a few years and make a cut of at least 10% from 2019.

Some developing countries like China, nations in Latin America and island states will freeze their use of HFCs from 2024.

International treaty designed to protect the environment against the impact of harmful substances

Created in 1987 following the discovery of a large hole in the Earth's ozone layer over Antarctica

Came into force in 1989 with the main aim of ending the use of chlorofluorocarbons (CFCs)

CFCs replaced by hydrofluorocarbons (HFCs)

Amendment proposed after scientists discovered that, while they pose no threat to the ozone layer, HFCs contribute to global warming by trapping heat radiating off the Earth

8.

Paris climate change agreement: China and US ratify deal as Barack Obama hails 'moment we decided to save our planet'

China and the US have formally ratified a historic climate change agreement drawn up in Paris to cut emissions and fight climate change. Presidents Barack Obama and Xi Jinping delivered documents to the UN Secretary-General entering their countries into the pact during a ceremony on Saturday.

The document certifies that the two countries – the world's worst polluters for carbon dioxide emissions – have taken all necessary domestic steps needed to join the agreement.

Mr Obama said the US was committed to being a global leader in the fight against climate change, hoping the Paris agreement would be remembered as the moment the world united to stop global warming.

“This is not a fight that any one country no matter how powerful can take alone,” he said. “Some day we may see this as the moment that we finally decided to save our planet.”

Mr Xi called the agreement a milestone that marks the “emergence of a global government system” for climate change. ​“Our response to climate change bears on the future of our people and the wellbeing of mankind.”

Erik Solheim, director of the United Nations environment programme, said the ratifications bring significant additional momentum to the drive against global warming.

“And by putting the wellbeing of our planet at the top of the agenda, the two largest economies in the world are also showing that our economic future is low-carbon and green," he said. “The fight against climate change remains difficult and urgent, but having heavy-hitters like China and the US on your side is extremely heartening.”

9.

Climate change: 2015 'shattered' global temperature record by wide margin

Global temperatures in 2015 were the warmest on record, according to data published by meteorologists in the UK and US.

The Met Office figures show that 2015 was 0.75C warmer than the long-term average between 1961-1990.

US data suggests that 2015 "shattered" the temperature record by the widest margin ever recorded.

Experts say the record temperatures were due to a combination of El Niño and human-induced warming.

Data produced by the US space agency (Nasa) and the National Oceanic and Atmospheric Administration (Noaa) show that 2015 "shattered" the previous record set in 2014 by 0.13C.

According to Noaa, the increase in temperature over land and ocean surfaces between 2014 and 2015 was the largest margin by which the record has been broken.

The fact that 2015 was going to break records had been heavily trailed by research agencies all over the world.

The Met Office predicted that 2015 would be between 0.52 and 0.76 above the long-term average. The actual temperature came in near the top end of that forecast.

Our warming world and El Nino

"Looking ahead, 2016 looks like it's also going to be another warm year and that's associated with the fact that human influence on the climate through greenhouse gas emissions has pushed us into new territory," said Dr Peter Stott from the British meteorological organisation's Hadley research centre.

We're much warmer than we were and then we've got the continuing effects of El Niño in the Pacific Ocean as well."

US experts agreed that the key reasons for 2015's record figures were a long-term trend of warming, mainly caused by the burning of fossil fuels, combined with the El Niño weather event.

10.

Climate change a serious security threat, warns Obama

President Obama has called climate change "a serious threat to global security" and heavily criticised climate change deniers.

In his strongest remarks on the issue yet, Mr Obama said climate change would "impact every country on the planet".

The US president made his comments in a speech to students at the Coast Guard Academy in Connecticut.

He has previously identified climate change as a central issue for the remainder of his time in office.

Faced with strong opposition on the issue from Republicans and industry, Mr Obama used his speech to frame the threat in terms of national security.

He said: "I'm here today to say that climate change constitutes a serious threat to global security, an immediate risk to our national security and make no mistake: it will impact how our military defends our country.

"Denying it or refusing to deal with it endangers our national security. It undermines the readiness of our forces."

He went on to list specific military facilities seen as under threat from adverse weather conditions.

"Around Norfolk, high tides and storms increasingly flood parts of our Navy base and an air base. In Alaska, thawing permafrost is damaging military facilities.

"Out West, deeper droughts and longer wildfires could threaten training areas our troops depend on."

He told the Coast Guard Academy students: "You are part of the first generation of officers to begin your service in a world where the effects of climate change are so clearly upon us.

"Climate change will shape how every one of our services plan, operate, train, equip, and protect their infrastructure, today and for the long term."

Mr Obama has used executive orders on climate change to circumvent opposition in Congress - issuing one in March ordering federal agencies to cut greenhouse gas emissions.

2017.1.1-2019.12.31

1.

Final call to save the world from 'climate catastrophe'

It's the final call, say scientists, in the most extensive warning yet on the risks of rising global temperatures.

Their dramatic report on keeping that rise under 1.5 degrees C says the world is now completely off track, heading instead towards 3C.

Keeping to the preferred target of 1.5C above pre-industrial levels will mean "rapid, far-reaching and unprecedented changes in all aspects of society".

It will be hugely expensive - but the window of opportunity remains open.

After three years of research and a week of haggling between scientists and government officials at a meeting in South Korea, the Intergovernmental Panel on Climate Change (IPCC) has issued a special report on the impact of global warming of 1.5C.

The critical 33-page Summary for Policymakers certainly bears the hallmarks of difficult negotiations between climate researchers determined to stick to what their studies have shown and political representatives more concerned with economies and living standards.

Despite the inevitable compromises, there are some key messages that come through loud and clear.

"The first is that limiting warming to 1.5C brings a lot of benefits compared with limiting it to two degrees. It really reduces the impacts of climate change in very important ways," said Prof Jim Skea, who co-chairs the IPCC.

"The second is the unprecedented nature of the changes that are required if we are to limit warming to 1.5C - changes to energy systems, changes to the way we manage land, changes to the way we move around with transportation."

2.

Changes needed by middle of century, say climate experts

Scientists describe the world after 1.5 degrees of global warming and ask people and governments to make some decisions.

A new report from the Intergovernmental Panel on Climate Change (IPCC) warns of the consequences of allowing global warming to reach 1.5°C above pre-industrial levels.

In their most authoritative assessment yet, the report by the United Nations scientist used data from 6000 independent studies to predict severe effects on sea level rise, increased heatwaves, lost coral reefs and depleted crop yields, all of which become more severe if global warming rises to 2°C.

Imperial College London experts were involved in the preparation of the report, with Dr Joeri Rogelj, Grantham Institute's Lecturer in Climate Change and the Environment coordinating and leading a chapter on climate change and sustainable development, and Professor Jim Skea from the Centre for Environmental Policy as co-chair of Working Group III, with responsibility for the report and its dissemination.

"Half a degree matters: half a degree of warming can make a world of difference for certain impacts," said Dr Rogelj. "Ways of keeping warming close to 1.5°C relative to preindustrial levels have been identified, and they all highlight the need for urgency."

The scientists recommended that in order to have a good chance of limiting the temperature rise to 1.5°C, human activities across the globe should be carbon-neutral by 2047. The urged measures to create smart cities with low-energy and few waste resources, alongside technological advances in agriculture, transport, manufacturing, homes and living.

"Deeper emissions reductions are required over the next decade to keep the window for limiting warming to 1.5°C open," said Dr Rogelj. Many scientists agree it will be necessary to call on unproven and expensive technologies to remove greenhouse gases, like carbon dioxide, from the atmosphere in order to reverse the warming effect.

3.

UN issues climate change warming

CLIMATE SCIENTISTS have warned that mankind has no more than a dozen years to rein in global warming, beyond which point it will significantly increase the risks of drought, floods, extreme heat and poverty for hundreds of millions of people.

The authors of the landmark report by the UN Intergovernmental Panel on Climate Change said that 'urgent and unprecedented changes' were needed to reach the target of limiting the man-made temperature increase to between 1.5C and 2C. They stressed that it achieving that was both practical and affordable, but acknowledged that it was at the 'ambitious' end of what was possible under the Paris Agreement.

Speaking for the Soil Association in the UK, head of farming Liz Bowles said: “The IPCC report shows that the world is not on track to avoiding devastating climate impacts to every aspect of human society, and underlines the fact that urgent and radical action is needed to cut emissions from agriculture.

“Climate change should be a top priority for Defra ministers, so it’s deeply worrying that just one line of the Agriculture Bill refers to the possibility of future support for cutting greenhouse gas emissions – particularly considering the recent Committee on Climate Change report which concluded there has been no progress in cutting agricultural emissions for the last decade.

“The government now urgently needs to prioritise support for widespread adoption of farming methods proven to reduce emissions and store more carbon in soils, such as organic farming and agroforestry. A shift towards less but better meat and dairy consumption and production is also needed, alongside dramatic cuts to food waste and a focus on shortening supply chains.

4.

Katowice: COP24 Climate change deal to bring pact to life

Negotiators in Poland have finally secured agreement on a range of measures that will make the Paris climate pact operational in 2020.

Last-minute rows over carbon markets threatened to derail the two-week summit - and delayed it by a day.

Delegates believe the new rules will ensure that countries keep their promises to cut carbon.

The Katowice agreement aims to deliver the Paris goals of limiting global temperature rises to well below 2C.

"Putting together the Paris agreement work programme is a big responsibility," said the chairman of the talks, known as COP24, Michal Kurtyka.

"It has been a long road. We did our best to leave no-one behind."

The summit accord, reached by 196 states, outlines plans for a common rulebook for all countries - regulations that will govern the nuts and bolts of how countries cut carbon, provide finance to poorer nations and ensure that everyone is doing what they say they are doing.

Sorting out the rulebook sounds easy but is very technical. Countries often have different definitions and timetables for their carbon cutting actions.

Poorer countries want some "flexibility" in the rules so that they are not overwhelmed with regulations that they don't have the capacity to put into practice.

The idea of being legally liable for causing climate change has long been rejected by richer nations, who fear huge bills well into the future.

A deadlock between Brazil and other countries over the rules for the monitoring of carbon credits threatened to derail the talks.

Brazil had been pushing for a weaker set of rules on carbon markets, despite strong opposition from many other countries. These discussions have now been deferred until next year.

5.

Trump: Climate change scientists have 'political agenda'

US President Donald Trump has accused climate change scientists of having a "political agenda" as he cast doubt on whether humans were responsible for the earth's rising temperatures.

But Mr Trump also said he no longer believed climate change was a hoax.

The comments, made during an interview with CBS's 60 Minutes, come less than a week after climate scientists issued a final call to halt rising temperatures.

The world's leading scientists agree that climate change is primarily human-induced.

Last week's report by the Intergovernmental Panel on Climate Change (IPCC) - the leading international body evaluating climate change - warned the world was heading towards a temperature rise of 3C.

Scientists say that natural fluctuations in temperature are being exacerbated by human activity - which has caused approximately 1C of global warming above pre-industrial levels.

The report said keeping to the preferred target of 1.5C above pre-industrial levels will mean "rapid, far-reaching and unprecedented changes in all aspects of society".

Climate change was just one issue touched on during the wide-ranging interview, during which Mr Trump also:

Said that "the day before" he took office the US had been on the verge of "going to war with North Korea"

Said Russian President Vladimir Putin was "probably" involved in assassinations but added, "I rely on them, it's not in our country"

Said Russia had meddled in the 2016 elections but added, "I think China meddled also"

Refused to say whether he would reinstate the migrant child separation policy but added "there have to be consequences" for entering the US illegally

Said he believed he had treated Christine Blasey Ford with "respect" after mocking her testimony in front of thousands at a rally, and that "had I not made that speech, we would not have won"

6.

Climate change policies need to improve for UK to reduce carbon emissions

Meeting the UK’s ‘net zero’ greenhouse gas emissions target will require revamping climate change policies and promoting ‘clean’ technologies.

This was the main conclusion of a new election analysis from the Centre for Economic Performance, by the London School of Economics, which involved input from Imperial researchers.

According to the findings of the report, UK greenhouse gas emissions are declining and have been declining for some time. The UK has a framework of long-run targets developed by the Committee on Climate Change, an independent body of experts advising government. Despite this, there are concerns about the country's ability to reach the 2050 target.

"The UK must do more to ensure it meets its climate targets", said Dr Ralf Martin, joint author of the report and Associate Professor of Economics at Imperial College Business School. "In doing so, UK policy-makers need to keep in mind that the policy objective must be to reduce global emissions."

"The UK can contribute to that most effectively by helping to develop, pilot and improve technology that will make a transition to clean technology the economically rational thing to do, even if households and firms ignore the potential damage from climate change"

The report says that a key factor of lower emissions growth over the last 10 years has been the drop in output and slow catch-up in the wake of the global financial crisis. But even without further recovery and continued slow growth due to Brexit, it is unlikely that future carbon targets will be met. The researchers highlighted how the UK can affect its own climate emissions and what happens in other countries, by pushing the knowledge frontier on clean technologies and practices.

7.

Climate change: UK 'can cut emissions to nearly zero' by 2050

The UK should lead the global fight against climate change by cutting greenhouse gases to nearly zero by 2050, a report says.

The Committee on Climate Change (CCC) maintains this can be done at no added cost from previous estimates.

Its report says that if other countries follow the UK, there’s a 50-50 chance of staying below the recommended 1.5C temperature rise by 2100.

A 1.5C rise is considered the threshold for dangerous climate change.

Some say the proposed 2050 target for near-zero emissions is too soft, but others will fear the goal could damage the UK's economy.

The CCC - the independent adviser to government on climate change - said it would not be able to hit “net zero“ emissions any sooner, but 2050 was still an extremely significant goal.

The main author Chris Stark told me: “This report would have been absolutely inconceivable just a few years ago. People would have laughed us out of court for suggesting that the target could be so high.”

The main change, he said, was the huge drop in the cost of renewable energy prompted by government policies to nurture solar and wind power.

He said the BBC's David Attenborough climate documentary, protests by Extinction Rebellion and speeches by the teenage campaigner Greta Thunberg had persuaded the public that the problem needed urgent action.

But Mr Stark said there was no way the 2050 target would be achieved unless the government backed it with policies and money.

He noted that the UK was already slipping away from a legal obligation to cut its emissions step-by-step between now and 2032.

8.

Climate change: CO2 emissions rising for first time in four years

Global efforts to tackle climate change are way off track says the UN, as it details the first rise in CO2 emissions in four years.

The emissions gap report says that economic growth is responsible for a rise in 2017 while national efforts to cut carbon have faltered.

To meet the goals of the Paris climate pact, the study says it's crucial that global emissions peak by 2020.

But the analysis says that this is now not likely even by 2030.

The report comes days before a major UN climate conference starting in Poland from 2-14 December.

For the last nine years, UN Environment have produced an assessment of the latest scientific studies on current and future emissions of greenhouse gases.

It highlights the difference between the level of greenhouse gas emissions that the world can sustain to keep temperatures within safe limits, with the levels that are likely based on the promises and actions taken by countries.

This year's report records the largest gap yet between where we are and where we need to be.

Between 2014 and 2016, global emissions of CO2 from industry and the production of energy were essentially stable while the global economy grew modestly - but in 2017 these emissions went up by 1.2% pushed along by higher GDP.

While the rise might seem small, it needs to be seen in context of efforts to keep global temperatures from rising by more than 1.5C, as recently outlined in a key IPCC report.

According to the UN, to keep the world below that target, global greenhouse gas emissions in 2030 would have to be 55% lower than today.

9.

Climate change: Data shows 2016 likely to be warmest year yet

Temperature data for 2016 shows it is likely to have edged ahead of 2015 as the world's warmest year.

Data from Nasa and the UK Met Office shows temperatures were about 0.07 degrees Celsius above the 2015 mark.

Although the Met Office increase was within the margin of error, Nasa says that 2016 was the third year in a row to break the record.

The El Niño weather phenomenon played a role, say scientists, but the main factor was human emissions of CO2.

The latest conclusions won't come as a much of a shock to observers, as the likely outcome was trailed heavily towards the end of last year.

Animation: Climate change explained in six graphics

What is climate change?

So warm was the early part of 2016 - influenced by a powerful El Niño - that some leading climate scientists were predicting as early as May that a new record was probable.

During an El Niño, a band of unusually warm ocean water develops in parts of the Pacific. The phenomenon affects the climate globally, disrupting weather patterns.

According to Nasa figures, 2016 is now the warmest year in a record that dates back to 1880.

"2015 has been the warmest year on record up until now, so 2016 has just beaten that and and it's beaten that by about 0.1-0.12 of a degree Celsius, which doesn't seem like a lot, but in terms of the year-to-year variations it's actually huge," Dr Gavin Schmidt from Nasa told BBC News.

"This is a very clear record that we're seeing. It is driven mainly by changes in the tropical Pacific where we had an El Niño event that produced a lot of warmth. But we've also seen long-term trends in warming mostly due to the increasing greenhouse gases."

10.

How kids organised one of the world's largest climate protests

In more than 112 countries, children are walking out to protest inaction on climate change – and many of the organisers have never met in person before. How are they doing it?

Last summer, on August 20, a 16-year-old-girl sat outside Swedish parliament, with a banner that said, “school strike for climate”. That girl was Greta Thunberg, whose goal – to get the Swedish government to act on climate change – created a social movement, led by young people around the world, most of whom can’t even vote yet. By November, committed groups of student activists had started to take up Greta's mantle too. On February 15, school children around the world walked out to protest political inaction on climate change, in one of the world’s largest ever protests.

Today, on March 15, there are scheduled walkouts in at least 112 countries. In the UK alone, there are more than 20 scheduled actions. For young people, particularly those who are still in school, climate change has become something of a flashpoint – evidence suggests that we only have 12 years to act decisively to keep our planet at a habitable temperature. But concrete action on climate change has stalled since the COP21 Paris Agreements (which many governments have yet to ratify or implement), leading many young people to believe that a protest is the only way to show how important the issue is to them.

“We should have started a while back, when there was still time to discuss it more deeply,” says David Wicker, a 14-year-old who lives in Italy and has been organising groups of students to protest via Fridays for Future, a youth organisation in Europe. “Now, we have no time for conferences that lead to nothing.”

2020.1.1-2022.11.1

1.

Temperatures of 40C in the UK ‘virtually impossible’ without climate change

Temperatures of 40C in the UK would be “virtually impossible” without climate change, scientists have said as temperatures climbed to record levels.

London Heathrow became the first place in the UK to ever record a temperature above 40C, reporting a provisional 40.2C at 12.50pm on Tuesday.

Met Office chief scientist Professor Stephen Belcher said: “I wasn’t expecting to see this in my career, but the UK has just exceeded 40C for the first time.”

He added: “For me it’s a real reminder that the climate has changed and will continue to change.”

He said Met Office research had showed that “it’s virtually impossible for the UK to experience 40C in an undisrupted climate, but climate change driven by greenhouse gases has made these extreme temperatures possible, and we’re actually seeing this possibility now.”

A recent Met Office study found that summers which see days above 40C are still very rare – although the risk of them is increasing – but they could occur as often as every three or four years by the end of the century if emission remain high.

Even with current pledges to cut emissions, 40C summers could occur every 15 years by 2100, the research found.

Prof Belcher said: “We are already committed to a level of warming and these extremes will get more extreme in the future.

“The only way we can stabilise the climate is by achieving net zero, and of course the UK has made some great strides in that direction.”

But he added: “We want to stabilise the climate at a safe level and that means reaching net zero soon.”

2.

Queen will not attend COP26 climate change summit

The Queen will not attend the COP26 climate change summit in Glasgow following medical advice to rest.

The 95-year-old monarch underwent preliminary medical checks in hospital last Wednesday after cancelling a visit to Northern Ireland.

She resumed public engagements on Tuesday by meeting ambassadors via video link from Windsor Castle.

Buckingham Palace said she "regretfully" decided not to attend a reception at the summit.

But the palace said she would deliver her address to delegates using a recorded video message instead.

The Queen was due to travel to Scotland as part of a string of COP26 engagements by senior members of the Royal Family including the Prince of Wales, the Duchess of Cornwall and the Duke and Duchess of Cambridge between 1-5 November.

It is understood that the monarch very much wants COP26 to result in meaningful action on climate change from participating nations, and hopes her absence will not be used by others as a reason not to attend.

The Queen was overheard at the opening of the Welsh Parliament earlier this month saying it was "really irritating" when people talk but don't act on climate issues.

Sir Peter Westmacott, a former UK ambassador to the US, said the cancellation was a "blow" to the summit, but argued the substance of the talks should not be affected.

Speaking on BBC Radio 4's Today programme, he said the Queen's attendance would have been the "icing on the cake" but it was still a "very important opportunity" for Prince Charles to speak alongside other senior royals.

3.

Climate change: UN emissions gap report a 'thundering wake-up call'

National plans to cut carbon fall far short of what's needed to avert dangerous climate change, according to the UN Environment Programme.

Their Emissions Gap report says country pledges will fail to keep the global temperature under 1.5C this century.

The Unep analysis suggests the world is on course to warm around 2.7C with hugely destructive impacts.

But there is hope that, if long term net-zero goals are met, temperatures can be significantly reined in.

Just a few days before COP26 opens in Glasgow, another scientific report on climate change is "another thundering wake-up call", according to the UN Secretary General, Antonio Guterres.

This week, we've already had a study from the WMO showing that warming gases were at a new high last year, despite the pandemic.

Now in its 12th year, this Emissions Gap report looks at the nationally-determined contributions (NDCs) or carbon-cutting plans that countries have submitted to the UN ahead of COP.

These pledges run up to 2030 and have been submitted by 120 countries. Unep has also taken account of other commitments to cut warming gases not yet formally submitted in an NDC.

The report finds that when added together, the plans cut greenhouse gas emissions in 2030 by around 7.5% compared to the previous pledges made five years ago.

This is nowhere near enough to keep the 1.5C temperature threshold within sight, say the scientists who compiled the study.

To keep 1.5C alive would require 55% cuts by the same 2030 date. That means the current plans would need to have seven times the level of ambition to remain under that limit.

4.

Climate change: Polls shows rising demand for government action

Popular support for governments to take tough action on climate change is growing around the world, according to a BBC World Service opinion poll.

The survey of over 30,000 people finds that 56% want their countries to play a leadership role at the critical COP26 meeting next week.

The desire to see ambitious goals set in Glasgow has grown substantially since 2015.

Concern about climate change is also at its highest point since 1998.

Presidents and prime ministers from around 120 countries will gather in Glasgow next week for the COP26 conference, dubbed the last, best chance of averting dangerous climate change.

Recent research shows that the plans so far on the table will not prevent global temperatures going far above 1.5C this century - a level that scientists say is the gateway to extreme impacts.

The UK, which is presiding over the talks, will hope that in negotiations with leaders they will be able to find a pathway that reduces emissions fast enough to stay below 1.5C.

This new poll suggests that people in rich and poor nations alike are supportive of the idea of greater ambition from their leaders.

Across the 31 countries polled, an average of 56% of people want their governments to set stronger targets that would address climate change as quickly as possible.

Another 36% want their government to take a more moderate approach and support gradual action.

Just 8% want their governments to oppose a deal.

In the 18 countries where a similar survey was carried out ahead of COP21 in Paris in 2015, the expectation for governments to play a leading role has grown substantially.

In 2015, 43% of those polled wanted strong action, but that has risen to 58% now.

5.

IPCC Sixth Assessment Report on Climate Change: Putting adaptation and resilience in focus

Today the IPCC has published the second part of its Sixth Assessment Report on Climate Change, which is focused on impacts, adaptation and vulnerability. It comes following the release of the first part of the report in August 2021, which established a global consensus on the role of humans in global warming and the rapid transformation required to address it, and is likely to be the last cycle of IPCC reports in this decade.

The conclusions reached by IPCC’s Working Group II, who put together the report, highlight the widespread adverse impacts of human-induced climate change and the risks that people and ecosystems will face if urgent action is not taken. The report reiterates disproportionate impact of climate change for some of the most vulnerable people in society, with current patterns continuing to increase their exposure to climate hazards.

The report notes that while there have been improvements in adaptation planning and implementation, that progress has been inconsistent and considerable gaps remain. Where many approaches prioritise reducing the most near-term challenges, the opportunity for significant, transformational adaptation is often missed. It calls for the mobilisation of financial resources and political commitments to create an environment in which more ambitious progress can be achieved.

In response, Stephanie Pfeifer, CEO, IIGCC commented: “Today’s IPCC report shows once again there is no time to wait; we must see urgent and accelerated action from the public and private sectors, including companies and investors, to drastically reduce greenhouse gas emissions while also facilitating the adaptation required to protect against the increasing physical risks posed by climate change to people and the planet.”

6.

Jeff Bezos Earth Fund: How can $10bn fight climate change?

Ten billion dollars - or £7.7bn - sounds like a serious amount of money.

It's how much the world's richest man Jeff Bezos has said he'll spend to fight climate change.

But what do you even spend $10bn on? And is it anywhere near enough?

According to Professor Elizabeth Robinson from the University of Reading, some of the solutions are known about but "are still not happening".

Elizabeth, an expert in environmental economics, suggests the money could be spent on persuading governments to stop funding fossil fuels - which produce carbon dioxide and warm the planet.

"We need to start investing in clean energy instead like renewable. If we do that, we're a lot of the way there."

The UK government announced a deal last year with the wind industry to ensure 30% of electricity comes from offshore wind by 2030.

Seamus Garvey, an energy storage expert at the University of Nottingham, agrees that renewable energy is key.

He says the technology is available but it needs to be "made cheaper" for people to use. "It's about flexibility in electricity."

Seamus suggests the need to mix wind and solar energy so it works all year long.

"Wind is very seasonal and blows strongly in the winter - as Storm Dennis has shown us. Solar is also seasonal and the sun shines mostly in the summer. So we need to balance it out."

There's lots of talk around the impact of cars and planes on the environment, so Elizabeth suggests money needs to be spent upgrading transport systems.

"We need to start investing in affordable low-cost public transport so people choose to move out of their cars and into trains."

7.

Climate change: World mustn't forget 'deeper emergency'

Despite the impacts of the coronavirus pandemic, the world mustn't forget the "deeper environmental emergency" facing the planet.

That's the view of the UN Secretary General Antonio Guterres, in remarks released to celebrate Earth Day.

The toll taken by the virus is both "immediate and dreadful", Mr Guterres says.

But the crisis is also a wake-up call, "to do things right for the future," said the Secretary General.

Mr Guterres re-iterated his view that the coronavirus is the biggest challenge the world has faced since the Second World War.

But as the world commemorates the 50th anniversary of Earth Day, the planet's "unfolding environmental crisis" is an "even deeper emergency", he says.

"Biodiversity is in steep decline," Mr Guterres stated.

"Climate disruption is approaching a point of no return.

"We must act decisively to protect our planet from both the coronavirus and the existential threat of climate disruption."

A long-term advocate of strong action to tackle global heating, Mr Guterres is now proposing six climate-related actions that should shape the recovery after the virus.

The world has to deliver new jobs and businesses through a "clean, green transition".

Taxpayers' money, when it is used, "needs to be tied to achieving green jobs and sustainable growth".

Money must be used to make people and societies more resilient to climate change, he says.

"Public funds should be used to invest in the future not the past."

Fossil fuel subsidies from governments is a theme that Mr Guterres has highlighted many times. These must end he says, and polluters must pay for their pollution.

8.

Climate change: Pakistan floods 'likely' made worse by warming

Global warming is likely to have played a role in the devastating floods that hit Pakistan, say scientists.

Researchers from the World Weather Attribution group say climate change may have increased the intensity of rainfall.

However there were many uncertainties in the results, so the team were unable to quantify the scale of the impact.

The scientists believe there's roughly a 1% chance of such an event happening in any coming year.

In the two months since flooding began in Pakistan, tens of millions of people have been affected, with around 1,500 dying because of the rising waters.

The intensity of the downpours saw the river Indus burst its banks, while landslides and urban flash floods swamped many areas.

Right from the start, politicians pointed to climate change as having made a significant contribution to the desperate scenes.

But this first scientific analysis says the picture is complex.

Certainly, the crippling heatwaves that gripped India and Pakistan earlier this year were easier to attribute, with researchers finding that climate change had made them up to 30 times more likely to happen.

But extreme rainfall events are hard to assess. Pakistan is located on the edge of the monsoon region where the rainfall pattern is extremely variable from year to year.

Further complications include the impact of large-scale weather events such as La Niña, which also played a role in the last major floods in Pakistan in 2010.

During the 60-day period of heaviest rainfall this summer scientists recorded an increase of about 75% over the Indus river basin, while the heaviest five-day period over the provinces of Sindh and Balochistan recorded a rise in rainfall of around 50%.

9.

Major UN report concludes world is off-track to fight climate change

The United in Science 2021 report shows climate change drivers and impacts are accelerating and not enough is being done to meet emissions targets.

The report – co-authored by Imperial College London researcher Dr Joeri Rogelj – presents the latest scientific data and findings related to climate change from multiple agencies

Its authors find that COVID-19 did not slow the relentless advance of climate change, and that there is no sign that countries are growing back greener, as carbon dioxide emissions have rebounded after a temporary blip due to the economic slowdown.

Data shows that greenhouse gas concentrations in the atmosphere continue to rise to record levels, committing the planet to dangerous future warming, and that rising global temperatures are fuelling devastating extreme weather throughout the world, with spiralling impacts on economies and societies. An estimated excess of 103 billion potential work hours were lost globally in 2019 compared with those lost in 2000.

The report also highlights that the past five years are among the warmest on record and there is an increasing likelihood that the average temperature will temporarily exceed 1.5°C above the pre-industrial era temperature in the next five years, which the Paris Agreement seeks to avoid.

The scale of recent changes across the climate system as a whole are unprecedented, and even with ambitious action to slow greenhouse gas emissions, sea levels will continue to rise and threaten low-lying islands and coastal populations throughout the world, according to the report.

10.

Why it's time to change the way we talk about climate change

Western Canada and the U.S. are currently enduring a climate-fuelled heat wave.

In British Columbia, temperatures hit 49.5 C breaking all-time records.

Scientists have been warning about the link between longer, more intense heat events and climate change for over 40 years.

Communication on these issues is vital, but we should focus more on solutions - particularly what individuals and communities can do.

New normal. Record-breaking. Unprecedented.

In recent days, as Western Canada and the United States have been broiling under a climate-fuelled heat crisis, all sorts of superlatives have been used to describe never-before-seen temperatures: the British Columbia community of Lytton hit a mind-boggling 49.5 C on June 29, breaking all-time temperature records three days in a row.

People are understandably shocked and scared by those numbers. But should this have come as a surprise? No. Scientists have been warning about the link between longer, more intense heat events and climate change for over 40 years. The language of “normals” and “new records” is rapidly becoming meaningless.

But the notion that humanity should have known, or should have done something about the crisis earlier — that we should be ashamed for our lack of inaction — is unhelpful for dealing with the climate crisis.

So, what’s a better, more helpful approach to communicating climate change?

The first thing to do is to spend more time talking about climate change. There is far too little discussion around this issue in the public sphere. Global heating is the biggest emergency the planet has ever faced, but one would not know it reading or listening to the news.

**Climate change search volume peak news**

2019.4

1.

Emma Thompson joins climate change protest - after hopping on 5,400-mile flight

Dame Emma Thompson arrived in London to join the Extinction Rebellion protests, even though she had to 5,400-mile flight to be there.

The 60-year-old Hollywood star flew in from Los Angeles and has stated she is willing to be arrested as part of the protest in central London.

Her first action was to urge others to "come and join" the demonstrations, in a video shared by the campaign group, despite running up a three-tonne carbon footprint to be in the thick of the protest.

Dame Emma admitted that she "wanted to be arrested" as part of her 60th birthday celebrations, which coincided with the first day of protests, but was 'away with her husband' at the time.

Speaking about joining the protest, Dame Emma said: "I absolutely wanted to be arrested on my 60th birthday but I didn't quite manage that.

"I'm so proud and thrilled to be part of Extinction Rebellion."

A representative of Dame Emma said she needed to take the 5,400-mile flight home to London after working in LA.

She joined the protests on the day organisers announced plans to target Heathrow Airport at the start of the Easter bank holiday weekend, which has been condemned by London mayor Sadiq Khan.

Dame Emma said: "We will occupy until you take action because that's the only way we are going to save our planet.

"We have to be here, we have to do this. It's inconvenient for people sometimes but its much more inconvenient to leave a planet that's so completely destroyed."

2.

Thousands of climate change protesters descend on London to ‘shut down capital’

Thousands of environmental protesters are hoping to ‘shut down’ London today in order to force the government to ‘declare a climate emergency’.

Activists led by the climate change group ‘Extinction Rebellion’, have come from across the UK, for what is described as an ‘international rebellion’.

They have pledged to block five central locations including Parliament Square, in a ‘non-violent act of resistance and rebellion’ that campaigners say could last for weeks.

The ‘festival of action’ is expected to converge on Marble Arch, Oxford Circus, Waterloo Bridge, Piccadilly Circus, as well as near Parliament.

It will involve people’s assemblies, performances, talks, workshops and food, campaign group Extinction Rebellion said.

Campaigners have also suggested that participants could attend ‘training sessions and briefings to make sure everyone is prepared for the mass civil disobedience to follow’, warning that while the movement is a peaceful one, there is always the possibility of arrest.

Demonstrators arrived at London’s Hyde Park on Sunday, with some having made the journey to the city on foot in recent weeks.

A spokesperson for Extinction Rebellion said: ‘Ordinary citizens, terrified by the threat we all face to our future, will risk arrest and imprisonment by blocking bridges, roads, harbours and transport systems.

‘Die-ins, cry-ins, funerals, banner drops, “blood” spilling, art and theatre will be part of a festival of global protest.

‘In London, people are willing to be arrested and to stay.’

Scotland Yard said they have ‘appropriate policing plans’ in place and that officers will be used from across the force ‘to support the public order operation during the coming weeks’.

They added: ‘We will always provide a proportionate policing plan to balance the right to a peaceful protest, while ensuring that disruption to communities is kept to a minimum.’

3.

Climate change protests: 122 arrests as Extinction Rebellion’s London shutdown continues

More than 120 protesters have been arrested during the ongoing anti-climate change demonstrations that have brought central London to a standstill.

The Met confirmed that 122 arrests have been made at the Extinction Rebellion protests since they began at 1pm yesterday, with the majority of people arrested for obstructing the Waterloo Bridge last night.

Read more: Climate change protest hits London for second day after 113 arrests

Five of the arrests were made for criminal damage at a commercial premises, after protesters vandalised Shell's London offices.

Climate change protesters vandalised the Shell Centre in central London yesterday (Source: Getty)

Extinction Rebellion said activists “intentionally” caused more than £6,000 worth of damage by smashing in windows and glass doors and spray painting the building.

The anti-climate change group has pledged not to disband its protests until the government takes “urgent action on the climate and ecological emergency”.

Protesters occupied have blockaded five tourist hotspots around London – Marble Arch, Oxford Circus, Waterloo Bridge, Parliament Square and Piccadilly Circus.

However, the Met yesterday issued an order for protesters to stay at Marble Arch after the demonstrations brought traffic in central London to a halt.

The Met imposed the order, which is expected to last until 6.55 pm today, after 55 bus routes were closed and 500,000 people were affected by the demonstrations.

“We are mindful of the impact the demonstrations are having on the local communities and are working hard with partners including TfL to keep London moving and open for businesses and visitors alike,” chief superintendent Colin Wingrove said this afternoon.

“At this time, ongoing demonstrations are causing serious disruptions to public transport, local businesses and Londoners who wish to go about their daily business.”

4.

Climate change activists climb Glasgow's Finnieston Crane for 'Rebellion Day' protest

A group of climate change protesters have took to the Finnieston Crane in Glasgow .

Four members of the Extinction Rebellion Scotland group began climbing the 175 feet tall structure by the banks of the River Clyde at around 5am on Monday.

The group revealed a massive banner which reads: "Science Not Silence".

Elsewhere in the city banners were pinned to the doors of the City Chambers, as well as Extinction Rebellion cones placed on top of statues in George Square.

The banner at the City Chambers building reads: "When the last tree has fallen humanity can't hide. Money can't solve it. What's your alibi?"

The protest was part of the group's 'Rebellion Day' - which saw attempts to raise awareness for climate change across the country and demand the declaration of a Climate Emergency by the world’s governments. Protests, including civil disobedience, are planned in 27 countries.

A number of banners were also put up in Aberdeen, including ones which read: "Climate crisis act now" and "Clean energy, not big oil".

A local member of Extinction Rebellion said: " We are just ordinary citizens like you and we want the council to act responsibly and save us from this accelerating disaster which may lead to serious flooding and food shortages in Scotland and across the world.

"27 other UK councils have declared a climate emergency. Why hasn’t Glasgow?"

Police arrived at the scene in Finnieston at around 7am.

A spokesperson confirmed that four people were reported to the procurator fiscal in connection with the protest.

Earlier this month, a group of 10 protesters from Extinction Rebellion got naked in parliament in an attempt to disrupt a Commons debate on Brexit.

5.

Semi-naked climate change protesters interrupt Commons debate on Brexit as they storm public gallery and GLUE themselves to the glass protecting MPs (and Ed Miliband can't believe his eyes)

Semi-naked climate change protesters interrupted a Commons debate on Brexit tonight as they stripped off in the public gallery.

Several of the 'Extinction Rebellion' group glued themselves to the window dividing MPs from the watching public in the biggest Commons security breach since 2014.

Labour MP Peter Kyle joked about the 'naked truth' as MPs' attention was distracted from his speech by the demonstration.

Tory MP James Heappey defied Commons rules to photograph the dozen people, who had slogans including 'climate justice act now' and 'eco collapse' daubed on their backs.

Speaker John Bercow told MPs to ignore the demonstration and continue with the debate as they stood in a line with their backsides pressed against the security glass.

Most were wearing only thong-style underwear which left little to the imagination.

Shocked MPs including former Labour leader Ed Miliband glanced up at the group as they stripped off behind the glass screen that separate the chamber from the public gallery.

Some of the protesters were singing Nelly the Elephant while under the gaze of Parliamentarians.

The protesters were later named as including Oliver Baines, 68, former charity chief executive, Bell Selkie, 48, a counsellor, Isla Macleod, 34, a celebrant, Iggy Fox, 24, a wildlife biologist and William Cooke, 36, former teacher and Mark Øvland, 35

Savannah, an English literature student from Ladbroke Grove in west London, was one of the naked protesters.

She said: 'A bunch of people glued themselves to the window in the public gallery.

'Everyone stripped and two people were elephants and had climate crisis written on them.

6.

Youth climate change strike London: Scores of students descend on Parliament Square for huge protest

Hundreds of students descended on Parliament Square today as part of the huge international youth campaign demanding action on climate change.

Eager young people handed out leaflets and newspapers for the Youth Strike 4 Climate movement as up to 800 people gathered before 11.30am on Friday to take part in a march.

Throngs of children held up colourful signs reading "Expect resistance!" and "Save our planet" at what was one of several UK-wide events in major cities including Sheffield, Leeds, Manchester and Brighton.

Organisers led spirited chants, including "We want, we want, justice" to the tune of the Queen song We Will Rock You.

The students then made their way past Downing Street chanting: "Theresa May, hear us say, climate change is here to stay.

It is the third mass student climate walkout in as many months.

Protesters are demanding that the Government declare a state of climate emergency as well as reforming the schools curriculum "to address climate change as an educational priority".

Families were among those joining the demonstration. Beth McLaughlin, 38, a psychologist from London, brought her children to the event to learn about how individuals can contribute to change.

Her son had been on all the Fridays for the Future school strikes so far.

Speaking in Parliament Square, she said: "I'd rather that they didn't have to miss school, but it makes more of a point and is more likely to effect change.

"Climate change is the most important issue there is, it makes all the other issues irrelevant really."

7.

OUR GOVERNMENT NEEDS TO ACT ON THE CLIMATE EMERGENCY

Gareth Redmond-King our Head of Climate Change explains what a climate emergency means, and what UK government needs to do to tackle our climate crisis.

Greta Thunberg, the school strikers, Extinction Rebellion and Sir David Attenborough have managed over the last few weeks to do what climate campaigners have been working hard to do for the last three decades – to get the message across to the British public that we are facing a climate emergency. Whatever you think about their various tactics, the message is absolutely right: climate change threatens our future and we are running out of time to tackle it.

Luckily, this is a crisis with a solution – rapid and deep emissions cuts. We have already promised our international partners that we will do our bit to prevent climate disaster by signing up to the Paris Agreement in 2015. By doing so we committed to reducing the UK’s emissions to net-zero in the first half of this century.

This week, the Committee on Climate Change publishes advice on how we get there and their advice must become the government’s blueprint for tackling the climate emergency. But while the Scottish government has already acknowledged that there is a climate emergency and committed to delivering the Committee’s advice, it remains to be seen whether there is enough political will in Westminster to follow suit. In the past the UK has been a climate leader, but right now Ministers are actively doing things which will make it much harder to get to net zero rapidly enough.

8.

Greta Thunberg Is In London And She Has Some Powerful Things To Say About Climate Change

In 2018, Greta Thunberg – a 16-year-old activist and Nobel Peace Prize nominee from Sweden – kick started a global youth movement in a bid to tackle climate change. Now, she’s on a European tour to meet with world leaders to address the issue. Having arrived in London, Thunberg is urging politicians to “listen to scientists”.

Read more: Climate Change Activist Greta Thunberg Is Nominated For The Nobel Peace Prize Aged 16

While appearing on BBC Radio 4’s Today programme on April 23, Thunberg stressed that everyone must work together to encourage politicians to act now. She made her message to clear: “Listen to the science, listen to the scientists. Invite them to talk,” she said. “I am just speaking on behalf of them, I’m trying to say what they’ve been saying for decades.”

Thunberg also praised the great efforts of Extinction Rebellion as climate change protests in the UK tip over into a second week (over 1,000 people have now been arrested). The activist group took over part of the Natural History Museum on April 22, which she believes is an important way to demonstrate: “As long as it’s non-violent, I think that could definitely make a difference.”

She’s been on a mission to curb climate change since she was a child. “When I was eight or nine years old my teacher showed our class photographs of plastic in the ocean and deforestation,” the told i-D. “I couldn’t stop thinking about those pictures. They were stuck in my head. I thought it was very sad that no one seemed to care about what was going on.”

9.

Extinction Rebellion has shifted politics on the climate

A new movement to demand action over climate change has changed British politics, argues Sarah Bates

Direct Action called by the Extinction Rebellion (XR) ­organisation has mobilised tens of thousands of people to demand climate justice.

The devastating impact of climate chaos has been highlighted by an “International Rebellion” ­coordinated by XR.

Last week saw climate chaos shoot to the top of the news agenda—extensive coverage has featured in every mainstream newspaper and TV broadcast. It’s being discussed in workplaces, universities, in ­supermarkets and on buses.

In central London activists occupied Marble Arch, Oxford Circus, Parliament Square and Waterloo Bridge from last Monday morning.

Each occupation was held by a dedicated group of activists, but many more people dropped in during the day or volunteered for night shifts.

Activist Lora signed up a month ago to help with admin, but soon found herself on an overnight shift in Marble Arch.

“It’s really uplifting to be here” she told Socialist Worker. “I was part of the wellbeing team until 1.30am handing out tea and snacks to people. I’ve never done anything like this before.

“I was nervous before I got here because it was out of my comfort zone. But as soon as I arrived I felt better, the atmosphere is great” she said.

Alongside existing XR supporters the week of action has brought together students, experienced environmental activists and those in the wider labour movement.

A trade union delegation organised by Campaign against Climate Change visited the Marble Arch and Oxford Circus occupations on Friday.

Campaign chair Suzanne Jeffery celebrated the “resilience and organisation” of rebels and said it was “hugely inspiring to see the movement make such an impact”.

10.

Naked body-painted climate change protester faces off with police outside Parliament – as football manager Joey Barton pledges his ‘solidarity’ to the cause too

A half-naked woman with her torso covered in body paint and a former England footballer were among the climate change protestors calling for dramatic cuts in carbon emissions in central London this afternoon.

The woman, whose back depicted an idyllic mountain lakeside scene, stood arms folded surrounded by police officers on the ninth day of the protest as the Extinction Rebellion group who have occupied Waterloo Bridge and Oxford Circus in the last week today marched on Parliament in their second week of demos.

Former Manchester City and England footballer Joey Barton, now manager of League One club Fleetwood Town, also appeared in 'solidarity' with the protestors today, as they looked resolved to make good their original promise to protest for two straight weeks.

More than 1,000 people have been arrested during climate change protests which started on Monday April 15. The action has seen Waterloo Bridge and Oxford Circus blocked and a 'die in' at the Natural History Museum.

Earlier today a skip lorry driver won fans online today after he was filmed berating one of the climate protesters bringing London to a standstill.

But a driver caught in tailbacks when police closed roads for the march said they were hitting ordinary working people and wasting police resources.

In footage shot by an LBC reporter, he said: 'We are sitting idle with their engines on. How is that helping? You're making vehicles, trucks, stop in traffic. I'm trying to earn my money like everyone else is. All it's doing is obstructing normal people.

2019.9

1.

Climate change report card: These countries are reaching targets

Following the UN Climate Change Conference, we take a look at which nations are on track to meet climate goals – and which are tanking.

It has been a little under four years since 196 countries negotiated the Paris Agreement, under which they committed to taking steps to limit the increase in global average temperature this century to well below 2 degrees Celsius (3.6 degrees Fahrenheit) over pre-industrial levels, and ultimately to limit that increase to 1.5 degrees C (2.7 degrees F). Under the agreement, each signatory submits its own national plan, setting targets for emissions reductions and specifying pathways by which it aims to meet those targets.

Despite the 2015 agreement, global carbon emissions increased 1.7 percent in 2017 and a further 2.7 percent in 2018; it has been estimated that the rate of increase in 2019 will be among the highest on record. The last four years have been the hottest on record, with 2019 on track to make it five. But analyses suggest that fast action now can reduce carbon emissions within 12 years and hold global increases below 2 degrees C and perhaps 1.5.

Are countries making progress? What kind? We got together with the Climate Action Tracker to see who’s dragging their heels and who is making the best efforts. The CAT covers all the biggest emitters and a representative sample of smaller emitters. Their data covers about 80 percent of global emissions and approximately 70 percent of the global population, and grades countries based on how likely their Paris commitments and actions, if replicated by other nations, would be to achieve a world of 1.5 degrees C of warming.

2.

Climate change summit: Global action falls ‘woefully short’ of what is needed, expert warns

The leaders of the world’s major economies fell “woefully short” of promising the concrete, dramatic action required to protect the planet from global warming at a United Nations summit yesterday, according to a leading climate change expert.

More than 60 countries promised to become carbon neutral by 2050, while over 65 pledged to step up action to tackle climate change – in most cases without giving details.

Meanwhile Russia, the world’s fourth biggest producer of greenhouse gas emissions, finally ratified the 2015 Paris Agreement to limit global warming to below 2C.

But despite these encouraging signs, the vast majority of meaningful new pledges came from the developing countries, cities and businesses, rather than the major economies and, taken together, are wholly inadequate, campaigners said.

Neither China nor the US, the world’s two biggest polluters, proposed any new meaningful action to tackle global warming and neither did the European Union.

“While countries were expected to come to the summit to announce that they would enhance their climate ambition, most of the major economies fell woefully short. Their lack of ambition stands in sharp contrast with the growing demand for action around the world,” said Andrew Steer, president of the World Resources Institute think tank.

“Many businesses and investors are ramping up their efforts, which should increase momentum. Similarly, smaller nations, especially the most vulnerable countries, are pushing ahead. But we need far greater national leadership on climate action– and we need it now,” added Mr Steer, a former World Bank climate change envoy.

3.

Greta Thunberg Can’t Do This Alone: How To Join The Fight Against Climate Change

Greta Thunberg condemned world leaders for their inaction on climate change at the U.N. Climate Action Summit on Monday, calling their failure to plan for drastic cuts in carbon emissions, which scientists say we need to avoid catastrophe, a "betrayal" of young people.

"You have stolen my dreams and my childhood with your empty words," Thunberg said. "And yet I’m one of the lucky ones. People are suffering. People are dying. Entire ecosystems are collapsing. We are in the beginning of a mass extinction. And all you can talk about is money and fairy tales of eternal economic growth. How dare you!"

While there were a few signs of progress at the summit, most of the biggest polluters failed to propose meaningful change.

“There’s a big dissonance between every leader saying to Greta, ‘We hear you’ and the commitments they are putting on to the table,” Isabel Cavelier, senior advisor at the Mission 2020 climate group, told The Guardian. “China said absolutely nothing new, India mentioned commitments made in the past, the U.S., Canada, and Australia aren’t here. We are seeing governments showing up empty-handed. There’s a feeling that the big emitters are holding things back.”

Though Thunberg's work has dominated coverage, there are also scores of young people around the world who have joined the movement against climate change, many of whom are participating in the Global Climate Strike this week. Several of them, along with Thunberg — including Alexandria Villaseñor, Chiara Sacchi, Catarina Lorenzo, Iris Duquesne, Raina Ivanova, and Deborah Adegbile — recently filed a lawsuit against five of the world’s major carbon polluters, stating that the countries are violating their rights as children. If the lawsuit succeeds, the U.N. would classify the climate crisis as a children’s rights crisis.

4.

Climate change: UN panel signals red alert on 'Blue Planet'

Climate change is devastating our seas and frozen regions as never before, a major new United Nations report warns.

According to a UN panel of scientists, waters are rising, the ice is melting, and species are moving habitat due to human activities.

And the loss of permanently frozen lands threatens to unleash even more carbon, hastening the decline.

There is some guarded hope that the worst impacts can be avoided, with deep and immediate cuts to carbon emissions.

This is the third in a series of special reports that have been produced by the Intergovernmental Panel on Climate Change (IPCC) over the past 12 months.

The scientists previously looked at how the world would cope if temperatures rose by 1.5C by the end of this century. They also reported on how the lands of the Earth would be affected by climate change.

However, this new study, looking at the impact of rising temperatures on our oceans and frozen regions, is perhaps the most worrying and depressing of the three.

In a nutshell, the waters are getting warmer, the world's ice is melting rapidly, and these have implications for almost every living thing on the planet.

"The blue planet is in serious danger right now, suffering many insults from many different directions and it's our fault," said Dr Jean-Pierre Gattuso, a co-ordinating lead author of the report.

The scientists are "virtually certain" that the global ocean has now warmed without pause since 1970.

The waters have soaked up more than 90% of the extra heat generated by humans over the past decades, and the rate at which it has taken up this heat has doubled since 1993.

5.

COMMUNITIES COPING WITH CLIMATE CHANGE

In September 2019, the Intergovernmental Panel on Climate Change (IPCC) released a special report on the Oceans and Cryosphere in a Changing Climate, which WWF made a significant scientific contribution to.

This report lays out in stark detail the impact climate change will have on the world’s frozen places, on our oceans and coastal areas, as well as the many millions of people that will be affected, and in some cases forced to leave their homes.

Around the world today, people are already living with the effects of vanishing ice, sea level rise and coastal erosion, which are destroying communities, impacting livelihoods and severely impacting traditional lifestyles.

Port Heiden is a native village of just over 100 people located 400 miles west of Anchorage on the Alaska Peninsula. It is only accessible by boat and plane. The entire village relocated - including buildings - in the late 1980s, as the coastline eroded and houses washed in to the sea. The new village moved about 5km away from the shore, severely impacting the community’s traditional subsistence lifestyle. The area still loses coastline every year.

Gerda Kosbruk is the Village Administrator and lifelong resident of Port Heiden. Gerda is passionate about her community’s struggle and the need for them to adapt and figure things out for themselves. Still, she expresses a hope that the world won’t forget about them.

"We watched our homes go into the ocean" said Gerda. "Where I played as a child is not there. How many people can say that where they grew up no longer exists? Not many people can tell that story. And we’re worth saving. We’re worth being here.”

6.

‘The Simpsons’ predicted Greta Thunberg’s climate change speech in 2007

The Simpsons has yet again predicted the future after a clip from the show’s 2007 movie saw Lisa Simpson give almost the exact same speech on climate change as Greta Thunberg.

On Monday (September 23), Thunberg delivered an emotive speech at the United Nations where she called out world leaders for their inaction on climate change. You can read the full speech here.

Now, a clip has been unearthed of Lisa from The Simpsons movie attempting to save Springfield from the dangers of environmental catastrophe.

In one key sequence, Lisa gives a speech to her classmates about what could happen to Springfield in 50 years unless something is done to avert the inevitable damage of climate change. The clip, which you can view below, is remarkably similar to the speech given by Thunberg this week.

It is not the first time the animated series has predicted the future. The creators predicted Daenerys Targaryen’s ‘mad queen’ turn in episode 5 of the final series of Game of Thrones.

A Game of Thrones parody episode of The Simpsons, called The Serfsons, was broadcast in 2017 and shows a dragon destroying Springfield and its inhabitants while The Simpsons family watched on.

In 2000, the episode ‘Bart To The Future’ envisioned a future in which Lisa Simpson is US president and inherits “quite a budget crunch” from President Trump. Elsewhere, in 1994 the show foreshadowed the 2013 horse meat scandal during the episode ‘Sweet Seymour Skinner’s Baadasssss Song’.

7.

Read climate change activist Greta Thunberg’s incredible speech to the UN – in full

Everyone is talking about the impassioned speech that 16-year-old Swedish climate change activist Greta Thunberg’s made at the UN Climate Change summit yesterday, as she made a heartfelt plea for world leaders to take more action towards cutting down emissions. Here is her incredible speech in full.

“My message is that we’ll be watching you. This is all wrong. I shouldn’t be up here. I should be back in school on the other side of the ocean. Yet, you all come to us young people for hope. How dare you! You have stolen my dreams and my childhood with your empty words. Yet I’m one of the lucky ones. People are suffering. People are dying. Entire ecosystems are collapsing. We are in the beginning of a mass extinction, and all you can talk about is money and fairy tales of eternal economic growth? How dare you?

“For more than 30 years, the science has been crystal clear. How dare you continue to look away, and come here and say you are doing enough when the politics and solutions needed are nowhere in sight? You say that you hear us and understand the urgency, but no matter how sad and angry I am, I don’t want to believe that. Because if you really understood the situation and still kept on failing to act, then you would be evil, and that I refuse to believe.

“The popular idea of cutting our emissions in half in 10 years only gives us a 50% chance of staying below 1.5°C and risks setting off irreversible chain reactions beyond human control.

8.

Top 3 things you need to know about the climate strike on 20 September

Inspired by school strikers, millions of people will be out on the streets around the world on Friday 20 September to demand action on climate change from their leaders. Here’s why you should get involved – and how to join in.

Young people have been going on strike from school on Fridays since Swedish teenager Greta Thunberg began her “School Strike for Climate” in August 2018. School children across the world then followed her lead, with monthly protests in the UK beginning in February 2019.

They argue that they’d rather be at school – but that there’s no point studying for a future under threat from climate change. They’ve done their homework, and the science is clear: this a climate emergency.

The idea of the Global Climate Strike is for as many people as possible to take the day off work on Friday 20 September to protest for climate action in solidarity with the school strikers. Young people will be out in force, and everyone else should be too.

Young people are really leading the charge on this one, and with good reason. The planet is set to warm by between 3C and 4C by 2100, with massive ramifications for everyone’s quality of life. As the next generation, their futures hang in the balance.

Whether you have children yourself, plan to, or neither, you probably agree that it would be ideal if the planet doesn’t go up in a ball of flames. Everyone should care.

Last year, one estimate suggested humans only had 12 years left to make the changes required. Then another estimate said it was more like 18 months.

9.

Climate change damaging purple heather: National Trust

Climate change is turning hillside heather from purple to brown, the National Trust says.

Acres of heather on the Long Mynd, in Shropshire, and Holnicote, on Exmoor, have suffered due to last year's hot, dry weather and an increase in pests caused by the following mild winter.

This has a knock-on effect on wildlife such as red grouse and emperor moths, whose caterpillars feed on the plants.

It is hoped "careful management" will allow the heather to recover.

The National Trust said up to 75% of the plant on both sites, which are in its care, was in poor health this year.

A prolonged hot summer in 2018 restricted water to the heather, while a lack of rain through the winter and first half of this year also took its toll.

The milder winter also boosted beetle numbers. The insects damage the heather's outer leaves and make it more susceptible to drought stress, the trust said.

Peter Carty, from the trust in Shropshire, said: "In places where heather was sheltered... or where damp conditions were present, the heather has survived.

"However, there will be no mass flowering this year."

Keith Jones, climate change specialist at the trust, said: "We are seeing first-hand the impacts of climate change on at least two of these special landscapes within our care."

At Holnicote, the trust is planting trees to slow the flow of water in the valley and restoring wet habitats such as blanket bogs to counter dry conditions.

Prolonged warmer weather could also boost heather shield bugs, a natural predator to heather beetles.

10.

Greta Thunberg marches in front of White House with American students over climate change crisis

Climate activist Greta Thunberg has taken her fight for the planet’s future to Donald Trump’s front door, marching alongside American students outside the White House.

Ms Thunberg, a 16-year-old who arrived in the US last month on a carbon emission-free sailboat, joined her US peers in a demonstration that at one point included an 11-minute “mass extinction” die-in to highlight the dire consequences of the changing climate.

“Hey, hey, ho, ho, climate change has got to go,” Ms Thunberg could be heard chanting along with the other students. The Swedish activist has gained a considerable following over the past year after she started a school strike outside her country’s parliament, sparking a global movement among students, known as “Fridays for Future”, who have followed her example.

“This is very overwhelming,” she said softly into a megaphone after the march in front of the White House, during which she walked amid the other activists. “See you next week,” she said towards the end of the event, referring to a planned 20 September global “Climate Strike” in which youth and adults are encouraged to walk out of school or work to urge more action on climate change.

Donald Trump is among a small minority of global leaders who has openly questioned the science of climate change. He has announced his intention to withdraw the United States from the Paris Climate Agreement, a global pact to stem the rise in global temperatures, and has a policy of maximising American production of fossil fuels.

Ms Thunberg has said she does not believe she can convince Mr Trump or other climate change doubters that global warming is real, but hopes they will take briefings from actual scientists and experts in this area.

2021.11

1.

Climate change: What did the scientists make of COP26?

Scientists and leading climate experts have voiced concerns about the outcomes of the COP26 climate conference, in Glasgow.

Those who spoke to the BBC praised the conference for getting countries to agree to meet again next year to pledge deeper emissions cuts. And they welcomed agreements on forests, innovation and especially methane - from fossil fuel extraction and livestock.

But the scientists fear politicians won’t deliver. And they say the hope of holding temperature rises to 1.5C above pre-industrial levels is far too unambitious anyway.

The experts say that with a temperature rise so far of just 1.1C, the world is already in a state of dangerous heating, with record temperatures, wildfires, floods and droughts.

Prof Sir David King, former UK chief scientist,told me: “Of course heating is already at a dangerous level. Greenland is sitting in blue sea for three months losing ice. Temperatures in the polar summer were 32C … the forests were on fire.

“Even if we cut emissions completely we’d still be in a difficult place because of the amount of greenhouse gases in the atmosphere already.”

There's a similar message from Prof Piers Forster, coordinating lead author for the Intergovernmental Panel on Climate Change. “People are already dying and species are becoming extinct with current temperatures," he said. "We have locked in centuries of sea level rise.

“1.5C has become the talisman for the safest we can hope for this century. But the more we learn the more we realise there is no safe limit [for emissions].”

The scientists we contacted appreciated that COP offered practical solutions.

2.

The tech to fight climate change is already here - now it needs urgent investment and support

After two weeks of negotiations, COP26 issued its final declaration, keeping alive the ambition of curbing global temperature increases to 1.5°C. A BCS team went to the summit to find out about the tech stories, and Computing at School did a presentation on Barefoot Climate Champion resources. BCS Senior Media Officer Claire Penketh reports.

A major aim of the 2021 United Nations Climate Change conference was to ensure global warming does not go above 1.5°C by 2100, to limit the worst impacts of climate change. Countries agreed at Glasgow to meet next year to pledge further major carbon cuts, with the aim of reaching the 1.5°C goal. Funding was also promised to help countries adapt to climate change.

This summit also marked the first time that coal was directly referenced in a COP agreement. However, the biggest disappointment for the smaller nations most affected by climate change and environmental activists, was the last-minute intervention by China and India which led to a commitment to ‘phase down’, instead of ‘phase out’ coal.

COP President Alok Sharma said China and India will have to justify themselves ‘to some of the most vulnerable countries’ over their stand on coal.

I spoke to Salem Avan, Director, Strategy, Policy & Governance Division, Office of Information and Communications Technology at the United Nations before the COP26 declaration was announced. His job, he said, includes ‘taking care of all technology that isn’t operational for member states... such as their technology needs.’

3.

COP26: Climate change will see 320 million people worldwide facing starvation this decade, report warns

The report, from Christian humanitarian organisation World Vision, also exposes the devastating impacts of long-term poor diet and food shortages on populations.

Key findings show 928 million people faced ‘severe’ food insecurity in 2020, with more than a quarter of the world’s children affected by malnutrition.

Nearly half of deaths in children under five years old – more than 2.5 million globally – are related to malnutrition.

Meanwhile, around one in eight people worldwide is at increasing risk of famine in coming years.

Increasingly frequent extreme weather events, brought on by climate change, are leading to cycles of drought and flooding which hamper farming.

This is especially disastrous for communities which are too poor to provide alternative food after crop failures.

The report highlights how a lack of healthy food can seriously impact the development of young children, leading to conditions which affect not only their own bodies but also the future of whole communities.

The aid organisation is calling for the threat to food and agriculture to be prioritised in actions to tackle the climate crisis.

Mark Sheard, chief executive of World Vision UK, said: “Weaker bodies are less able to perform necessary work needed for abundant agriculture.

“This means that food insecurity is a compounding issue which worsens year-on-year and generation-by-generation.

“World leaders must put food and nutrition at the top of the priority list when discussing climate change or risk condemning the world’s most vulnerable children to hunger, malnutrition and death.”

Speaking at a World Vision panel event at COP26 in Glasgow, Nick Dyer, the UK’s special envoy for famine prevention, confirmed the problem.

4.

Impact of climate change on workers’ health and safety

With the UN Climate Change Conference (COP26) in full swing, SHP looks at the importance of the event on a global scale, while also focusing on how the effects of climate change are seeping into the workplace, exploring what businesses can do to safeguard staff and contribute to the global effort.

The main goals of COP26, drafted to accelerate action towards the goals of the Paris Agreement and the UN Framework Convention on Climate Change, are as follows:

Secure global net zero by 2050 and keep 1.5 degrees within reach

Adapt to protect communities and natural habitats

Mobilise finance – to deliver the first two goals, developed countries must mobilise at least $100bn in climate finance per year

Work together to deliver – countries must accelerate action to tackle the climate crisis through collaboration between governments, businesses, and civil society.

According to the BBC, the Queen has urged world leaders at the COP26 climate summit to “achieve true statesmanship” and create a “safer, stabler future” for the planet.

A review carried out by Environmental Health, argues that, in the UK, climate change will directly influence health outcomes through changing exposure to heat and cold, air pollution, pollen, food safety risks, disruptions to access to and functioning of health services and facilities, emerging infections, flooding and other reasons such as water-borne diseases and increased exposure to UV radiation, as well as indirectly via for example changing prices of and access to food and energy.

Not only are businesses being encouraged to think about how they will contribute in the race to hit climate change targets, but employers are also being faced with a growing need to deploy strategies to manage the risk climate change poses to the health and safety of their employees.

5.

COP26: World headed for 2.4C warming despite climate summit – report

Despite pledges made at the climate summit COP26, the world is still nowhere near its goals on limiting global temperature rise, a new analysis shows.

It calculates that the world is heading for 2.4C of warming, far more than the 1.5C limit nations committed to.

COP26 "has a massive credibility, action and commitment gap", according to the Climate Action Tracker (CAT).

The Glasgow summit is seen as crucial for curbing climate change.

But the prediction contrasts with optimism at the UN meeting last week, following a series of big announcements that included a vow to stop deforestation.

COP26 is expected to finish this week.

The projection comes as the UK's Met Office warns that a billion people could be affected by fatal heat and humidity if the global average temperature rises by 2C above pre-industrial levels.

The report by Climate Action Tracker looks at promises made by governments before and during COP26.

It concludes that, in 2030, the greenhouse gas emissions that warm the planet will still be twice as high as necessary for keeping temperature rise below 1.5C degree.

Scientists say that limiting warming to 1.5C will prevent the most dangerous impacts of climate change from happening.

The COP summit held in Paris in 2015 laid out a plan for avoiding dangerous climate change which included "pursuing efforts" to keep warming under 1.5C.

But when governments' actual policies - rather than pledges - are analysed, the world's projected warming is 2.7C by 2100, suggests Climate Action Tracker. The Tracker is backed by a number of organisations including the prestigious Potsdam Institute for Climate Impact Research in Germany.

6.

Climate change: the worst offenders

Joe Biden launched a broadside at China and Russia for their failure to attend Cop26 as he signed off from the summit in Glasgow yesterday.

The US president claimed his counterparts Xi Jinping and Vladimir Putin had lost influence on the world stage by shunning the climate change conference. “China, Russia, and Saudi Arabia not showing up, was a problem,” Biden said in his final press briefing at the opening two-day World Leaders Summit. “We showed up. And by showing up, I think we’ve had a bit impact on how the rest of the world looks at the US.”

China has committed to achieving net zero emissions by 2060, while Putin has said that Russia “will strive” for the same target. But both countries are high on the list of the world’s biggest carbon dioxide emitters, along with the US.

China’s “huge” population and “explosive economic growth” have “pushed it way ahead of any other country in its overall emissions”, said the BBC.

Analysis by the Financial Times of data from Climate Watch and the International Energy Agency found that China’s CO2 emissions (CO2e) have increased significantly since the 1990s. Latest figures show that country emitted 11.7bn tonnes of CO2 in 2018 – almost a quarter of the global total.

China remains heavy reliant on fossil fuels. According to the newspaper’s analysis, 70% of the Asian nation’s electricity was produced from non-renewable or nuclear fuels, with renewables at 26% and nuclear at 4%.

Beijing has pledged to begin cutting greenhouse gas emissions by 2030, and to increase its capacity of wind and solar power to more than 1.2bn kilowatts by the same deadline. But as the BBC noted, President Xi “has not said exactly how these goals will be achieved”.

The total proportion of global carbon emissions coming from the US is less than half that of China, at 11.84%. But the Western superpower tops the list of the five biggest emitters in terms of tonnes of CO2 per capita, at 15 tonnes per person, compared with China’s seven.

7.

What is the 1.5 degrees climate change pledge? What happens if we pass 1.5 degrees of global warming?

The Paris Agreement is an international treaty which saw 196 countries commit to limiting global warming to well below 2, preferably 1.5 degrees Celsius.

Since, countries have pledged an aim to cut greenhouse gas emissions so the planet can become climate neutral by 2050 – with net zero greenhouse gas emissions.

But what does the 1.5 °C target actually mean? Why is it so important? And would would happen if world temperatures rise beyond it?

Since the Industrial Revolution, the world’s average surface temperature has risen by around 1 degree Celsius.

It might sound like a tiny number, but it has had an enormous impact on nature and human life. Glaciers and ice sheets have melted, sea levels have risen, and extreme weather events are on the rise.

The vast majority of scientists agree greenhouse gases, released into the atmosphere by human activity, are the cause of this warming.

And scientists project limiting warming to 1.5 degrees would reduce the worst impacts of climate change.

The Paris Agreement in 2015 adopted at COP21 had a goal of limiting global warming to well below 2 degrees – preferably 1.5 degrees. Countries also agreed to limit their CO2 emissions to ‘net zero’ by 2050.

However, despite this landmark agreement, we are currently on track to a temperature rise of a shocking 2.7 degrees above pre-industrial levels before the end of the century.

The 1.5°C pledge is crucial to secure a liveable future on Earth, according to the Intergovernmental Panel on Climate Change (IPCC).

8.

COP26: 17 key moments from the United Nations climate change conference in Glasgow

COP26, a United Nations conference on climate change in Glasgow is coming to an end, after world leaders including Boris Johnson and Joe Biden met to recommit to action towards slowing down global warming.

The 26th UN Climate Change Conference of the Parties (COP26) is coming to a close in Glasgow, two weeks after bringing together world leaders and delegates to accelerate action towards the goals of the 2015 Paris Agreement and the UN Framework Convention on Climate Change.

Following the G20 summit in Rome on 31 October, where leaders of the world’s 20 major economies reportedly struggled to bridge differences over how to combat global warming, the conference and surrounding events were attended by around 25,000 people, including senior officials, environment ministers and activists like Greta Thunberg.

The summit in Glasgow had been due to conclude at 6pm on Friday, but was extended as negotiators from 197 countries talked into the night in the hope of securing agreement, with an announcement now expected over the weekend.

Both China and Russia have failed to send physical representatives to the conference, which was attended by US President Joe Biden, German Chancellor Angela Merkel and French President Emmanuel Macron.

Analysis already that the pledges put forward at the summit could put the world on track for under 2°C of global heating for the first time, if they are fully committed to.

In the landmark Paris agreement, signed in 2015, nations committed to holding global temperature rises to “well below” 2°C above pre-industrial levels, while “pursuing efforts” to limit heating to 1.5°C. Those goals are legally binding and enshrined in the treaty.

9.

COP26: UK firms forced to show how they will hit net zero

Most big UK firms and financial institutions will be forced to show how they intend to hit climate change targets, under proposed Treasury rules.

By 2023, they will have to set out detailed public plans for how they will move to a low-carbon future - in line with the UK's 2050 net-zero target.

An expert panel will set the standards the plans need to meet to ensure they are not just spin.

Any commitments will not be mandatory. Green groups say this is not enough.

Net zero is when a business or a country achieves an overall balance between the amount of carbon it is emitting and the carbon that it's removing from the atmosphere.

Firms and their shareholders will be left to decide how their businesses adapt to this transition, including how they intend to decarbonise.

And although the plans will need to be published, the government said "the aim is to increase transparency and accountability" and the UK was not "making firm-level net-zero commitments mandatory".

The market will decide whether firms' plans are credible, the Treasury said.

Speaking at the COP26 climate summit, Chancellor Rishi Sunak claimed the UK was leading the world in becoming the "first-ever net zero aligned global financial centre".

He said the changes would mean: "Better and more consistent climate data; sovereign green bonds; mandatory sustainability disclosures; proper climate risk surveillance; and proper global reporting standards."

In total, 450 firms controlling 40% of global financial assets - equivalent to $130tn (£95tn) - have agreed to commit to limit global warming to 1.5C above pre-industrial levels.

10.

COP26: Boris Johnson compares climate change to James Bond - 'the Doomsday device is real'

[Boris Johnson](https://uk.news.yahoo.com/tagged/boris-johnson) used his opening speech to compare the climate crisis to a James Bond movie.

In his speech at the opening ceremony for [COP26](https://www.yahoo.com/tagged/cop26) on Monday, the prime minister compared the global climate emergency as similar to a 007 film.

He used the metaphor of the spy wearing a bomb which is about to explode, but lamented that unfortunately [climate change](https://uk.news.yahoo.com/tagged/climate-change) was a reality and not a movie.

"Welcome to Glasgow, and to Scotland, whose most globally famous fictional son is almost certainly a man called James Bond – who generally comes to the climax of his highly lucrative films strapped to a Doomsday device desperately trying to work out which coloured wire to pull to turn it off," he told world leaders from more than 100 countries.

"A red digital clock ticks down remorselessly to a detonation that will end human life as we know it, and we are roughly the same position, my fellow global leaders, as James Bond today.

"Except that the tragedy is this is not a movie, and the Doomsday device is real.

"And the clock is ticking to the furious rhythm of hundreds of billions of pistons and turbines and engines with which we are pumping carbon into the air faster and faster, record output and quilting the earth in a suffocating blanket of CO2."

Johnson, known for his unusual metaphors – such as using Kermit the Frog in a comparison about "going green" – added: "Let us think about those beady eyes, watching us around the world."

2022.4

1.

Climate change: IPCC scientists say it's 'now or never' to limit warming

UN scientists have unveiled a plan that they believe can limit the root causes of dangerous climate change.

A key UN body says in a report that there must be "rapid, deep and immediate" cuts in carbon dioxide (CO2) emissions.

Global emissions of CO2 would need to peak within three years to stave off the worst impacts.

Even then, the world would also need technology to suck CO2 from the skies by mid-century.

After a contentious approval session where scientists and government officials went through the report line by line, the UN's Intergovernmental Panel on Climate Change (IPCC) has now published its guidance on what the world can do to avoid an extremely dangerous future.

First, the bad news - even if all the policies to cut carbon that governments had put in place by the end of 2020 were fully implemented, the world will still warm by 3.2C this century.

This finding has drawn the ire of the UN Secretary General Antonio Guterres.

"Some government and business leaders are saying one thing - but doing another. Simply put, they are lying. And the results will be catastrophic."

That sort of temperature rise would see our planet hit by "unprecedented heatwaves, terrifying storms, and widespread water shortages".

To avoid that fate, the world must keep the rise in temperatures at or under 1.5C this century, say researchers.

The good news is that this latest IPCC summary shows that it can be done, in what Mr Guterres calls a "viable and financially sound manner".

But keeping temperatures down will require massive changes to energy production, industry, transport, our consumption patterns and the way we treat nature.

2.

Avoiding the worst of climate change is possible if we act now

We have just three years to keep the dream of 1.5⁰C alive.

Scientists from around the world have warned that carbon dioxide emissions must peak within three years, with steps to slash the use of fossil fuels and switching to cleaner energy needed to prevent even more damaging climate change.

We can adapt to rising global temperatures, but we need to start now.

This is the message of a crucial report on mitigating climate change from the Intergovernmental Panel on Climate Change (IPCC), comprising decades' worth of evidence assembled by hundreds of scientists.

The report is the third of a trilogy released over the past year examining the science and impacts of climate change, and how to avoid the worst of its impacts.

It advocates for a 'substantial reduction in overall fossil fuel use', with minimal use in the future if it cannot be avoided. Instead, support for renewable energy, and new carbon removal technology, is crucial if the world is to avoid overshooting the climate buffers.

The United Nations Secretary-General, António Guterres, was blunt in his assessment of the current state of climate policies, describing the report as 'a litany of broken promises' and 'a file of shame.'

He said, 'Some government and business leaders are saying one thing - but doing another. Simply put, they are lying. And the results will be catastrophic.'

He added that nations and businesses investing in fossil fuels were 'dangerous radicals' that were 'adding fuel to the fire' and putting the world on 'a fast track to climate disaster.'

The IPCC chair, Dr Hoesung Lee, said that despite the bleak assessment, change is possible.

3.

Earth Day 2022: Climate Change and Mountains - The Latest Science

Earlier this year, the UN's climate change panel, the IPCC, released the second part of its Sixth Assessment report, titled Climate Change 2022: Impacts, Adaptation & Vulnerability. This Earth Day, we're sharing the key points highlighted in the chapter focused on mountain environments.

As we struggle to meet the long-term Paris Agreement temperature goal to limit global warming to below 2°C and towards 1.5°C, mountains face elevation-dependent warming, shrinking glaciers, reduced snow cover and changing precipitation patterns, causing knock-on effects for water supply, energy production, ecosystem integrity, agricultural and forestry production and disaster preparedness. These crises come as additional strains to mountain communities already disadvantaged by geographical and political isolation.

Scientists estimate that climate change is affecting major mountain ranges two to three times faster than the rest of the world. One-third of Himalayan glaciers — at the very least — are predicted to disappear by 2100, as temperatures rise at a rate estimated to be up to three times the global average.

In the European Alps, the mercury has risen by 2°C over the course of the 20th century – a rise greater than the French average of 1.4°C, and double that of the northern hemisphere. According to one study, if present climatic conditions are maintained, the vanishing Argentière Glacier and the Mer de Glace could both disappear by the end of this century.

Alongside the health and safety risks posed by climate-change related disasters to local populations, climbing and trekking routes are shifting with changing conditions: access to the base of glacial routes is proving more difficult or riskier, while rockfall is increasing in some areas. These specific changes impacting outdoor activities are noted in the new paper.

4.

Climate change: Meaning behind Earth Day 2022 and how the Google Doodle is marking today’s climate event

Earth Day is recognised across the planet as a chance to raise awareness of the environmental crisis we all face

Google is marking this Earth Day with a doodle showing time-lapse satellite images of melting glaciers, retreating snow cover, coral bleaching and deforestation to highlight the threat of climate change.

Inaugurated in the United States in 1970, Earth Day is now recognised across the planet as an occasion on which to raise awareness of environmental issues.

Earth Day has been held on 22 April ever since it inception in 1970.

The concept was proposed by peace activist John McConnell at a 1969 Unesco conference in San Francisco, and was originally planned to be on 21 March, the first day of spring.

A month later, US senator Gaylord Nelson proposed a nationwide environmental teach-in on 22 April, an event which consumed McConnell’s plan.

It was named Earth Day, and its purpose grew, culminating in it becoming the largest single-day protest in human history.

On Earth Day 2016, the Paris Agreement, which focuses on climate change, was signed by more than 120 countries, including the UK, United States and China.

The theme of Earth Day 2022 is “Invest in Our Planet”.

“This is the moment to change it all – the business climate, the political climate, and how we take action on climate,” the Earth Day website states.

“Now is the time for the unstoppable courage to preserve and protect our health, our families, our livelihoods.”

5.

Google Doodle Earth Day: Time Lapses Show Impact of Climate Change

The time lapses show how climate changes is affecting the whole world, from Greenland to the Great Barrier Reef.

Google Doodle is observing Earth Day with Google Earth time lapses that reveal climate change’s impact on our planet.

The time lapses show how places across the world, from Mount Kilimanjaro to the Great Barrier Reef, have been impacted in recent years.

Google Doodle said: “Today’s annual Earth Day Doodle addresses one of the most pressing topics of our time: climate change.

“Using real time-lapse imagery from Google Earth Timelapse and other sources, the Doodle shows the impact of climate change across four different locales around our planet.”

Google encourages users to “Stay tuned throughout the day to view these scenes, each remaining on the homepage for several hours at a time.”

The four scenes featured in today’s Google Doodle are Mount Kilimanjaro in Tanzania, Sermersooq in Greenland, Australia’s Great Barrier Reef, and the Harz Forests in Germany.

Google Earth images from Mount Kilimanjaro, taken every December from 1986 until 2020, show glacier retreat at the summit, while glacier retreat in Greenland has been documented annually from 2000 to 2020.

The images from the Great Barrier Reef show coral bleaching on Lizard Island from March until May 2016, while footage from Germany, taken every December from 1995 to 2020, shows how the Harz Forests are being destroyed by bark beetle infestation, caused by rising temperatures and severe drought.

Google says: “Acting now and together to live more sustainably is necessary to avoid the worst effects of climate change,” and encourages users to use the search engine to “learn more about climate change and how you can take action.”

6.

Climate change could cause diseases to gain new hosts

Viruses could acquire thousands of new hosts as climate change causes increasing contact between different mammal species.

Diseases such as Ebola virus are likely to spread further than ever before, while new pathogens could emerge with the potential to affect both animal and human populations.

More than 15,000 instances of diseases crossing into new species could take place in the next 50 years as a result of climate change.

As mammals shift their ranges to adjust to increasing temperatures, thousands of new species will come into contact for the first time.

Even if climate change is limited to less than 2⁰C, more than 300,000 pairs of mammals which have never met before will meet, leaving open the possibility that the viruses they carry will jump from animal to animal or even into humans.

Dr Gregory Albery, who led the paper alongside Dr Colin Carlson, says, 'As the world changes, the face of disease will change too. Because climate change is shaking our ecosystems to their core, the way that we understand viruses and their ecologies will need to evolve.

'We need to start preparing for things not just as they are, but how they will be. By demonstrating that moving mammals will meet each other for the first time and form entirely new communities, we have demonstrated a novel and potentially devastating mechanism for disease emergence that could threaten the health of animal populations in the future.

'This will most likely have ramifications for our health too. This work provides incontrovertible evidence that the coming decade will not only be hotter, but sicker.'

The findings of the study were published in Nature

7.

IPCC Sixth Assessment Report on Climate Change: Mitigation of climate change

The IPCC has published the third part of its Sixth Assessment Report on Climate Change, focusing on mitigation. The report follows the second part of the report published in February this year which focused on impacts, adaptation and vulnerability.

The conclusions reached by IPCC’s Working Group III, who put together the report, outline the need to significantly reduce emissions by 2030 and the broad measures to do so. In particular, the report highlights the requirement for major transitions in the energy sector, which involves a substantial reduction in fossil fuel use, widespread electrification, improved energy efficiency and the use of alternative fuels.

The report does find that climate action is having a positive effect, but that the pace of change needs to accelerate, with the next few years critical. Specifically, the report states that limiting warming to around 1.5°C requires global greenhouse gas emissions to peak before 2025 at the latest, and be reduced by 43% by 2030.

In response, Stephanie Pfeifer, CEO, IIGCC, said: “Today’s IPCC report is a powerful reminder not just to investors, but governments, companies and civil society of the role they all must play in facilitating emissions reductions at scale if global warming is to be limited to 1.5°C. While the rallying cry is as loud and necessary as ever, the IPCC’s report does highlight that climate action is already delivering some positive results, noting that in many countries that there are policies, regulations and market instruments that are proving effective.”

“For investors, by highlighting the need to reduce emissions in industry, particularly those on the demand side in energy intensive sectors such as steel, cement and aviation, the IPCC report makes clear the fundamental and valuable role investors must play in helping companies in such sectors towards decarbonisation.

8.

Climate change and farming driving insect decline

Insect numbers have plunged by half in some parts of the world due to climate change and intensive agriculture, a study has found.

The combined pressures of global heating and farming are driving a "substantial decline" of insects across the globe, according to UK researchers.

They say we must acknowledge the threats we pose to insects, before some species are lost forever.

But preserving habitat for nature could help ensure vital insects thrive.

Lead researcher, Dr Charlie Outhwaite of UCL, said losing insect populations could be harmful not only to the natural environment, but to "human health and food security, particularly with losses of pollinators".

"Our findings highlight the urgency of actions to preserve natural habitats, slow the expansion of high-intensity agriculture, and cut emissions to mitigate climate change," she added.

Plummeting populations of insects around the world - a so-called "insect apocalypse" - have caused widespread concern.

However, scientific data gives a mixed picture, with some types of insects showing drastic declines, while others are staying steady.

In the latest study, the researchers pulled together data on the range and number of nearly 20,000 insect species, including bees, ants, butterflies, grasshoppers and dragonflies, at about 6,000 different locations.

In areas with high-intensity agriculture and substantial warming, insect numbers have plunged by 49% and the number of different species by 27%, compared with relatively untouched places that have so far avoided the most severe impacts of climate change, according to the research, published in Nature.

But the researchers said there was some cause for hope in that setting aside areas of land for nature created a refuge for insects, which need shade to survive in hot weather.

9.

Europe experienced its warmest summer on record in 2021, accompanied by severe floods in western Europe and dry conditions in the Mediterranean

The Copernicus Climate Change Service today releases its annual European State of the Climate report, examining climate variability of 2021 in Europe and globally. The report also provides the longer-term context and trends in key climate change indicators.

Globally:

The last seven years were the warmest on record, with 2021 ranking between 5th and 7th warmest[1]

Greenhouse gas concentrations of carbon dioxide (CO2), and particularly methane (CH4), continued to rise during 2021, by around 2.3 ppm and 16.5 ppb, respectively

In Europe:

Europe experienced its warmest summer on record, at 1.0°C above the 1991-2020 average[2]

Record rainfall contributed to severe flooding in western Europe

Annual sea surface temperatures (SST) in large areas of the Baltic and eastern Mediterranean Seas were the highest since at least 1993. In June and July, SSTs in parts of the Baltic were more than 5°C above average.

Summer extremes in the Mediterranean region:

The Mediterranean region experienced an intense and prolonged heatwave in July-August

Temperature records were broken in Italy and Spain, the former being a provisional maximum daily temperature record for Europe

Several countries, including Turkey, Greece and Italy saw intense wildfires

Low wind speeds in western Europe:

Some countries saw some of the lowest annual wind speeds since at least 1979

Lower than average wind speeds led to reduced potential for wind energy production in some western European countries

Flooding in western Europe:

Record rainfall on 14th July 2021 over Belgium and western Germany

Record river discharge in parts of the Meuse and Rhine catchments

Record-breaking rainfall and saturated soils contributed to the exceptional flooding event

10.

EXPLAINER: Can climate change be solved by pricing carbon?

As climate change bakes the planet, dozens of nations including the U.S. and many local governments are putting a price tag on greenhouse gas emissions that are causing more floods, droughts and other destructive events

As climate change bakes the planet, dozens of nations and many local governments are putting a price tag on greenhouse gas emissions that are increasing flooding, droughts and other costly catastrophes.

Pennsylvania on Saturday becomes the first major fossil fuel-producing state in the U.S. to adopt a carbon pricing policy to address climate change. It joins 11 states where coal, oil and natural gas power plants must buy credits for every ton of carbon dioxide they emit.

President Joe Biden is attempting a less direct approach — known as the social cost of carbon — that calculates future climate damages to justify tougher restrictions on polluting industries. Republicans say that could crush many businesses. They want the U.S. Supreme Court to stop the administration after lower courts in Louisiana and Missouri split on the issue.

Governments elsewhere have moved more aggressively. Canada, for example, imposes fuel charges on individuals and also makes big polluters pay for emissions. It's one of 27 nations with some kind of carbon tax, according to The World Bank.

The varied strategies come as scientists warn climate change is accelerating — and all can help reduce emissions. But experts say U.S. efforts have been hobbled by its fractured approach.

“Part of the reason you need all of these things to work in tandem is we do not have a federal climate policy," said Seth Blumsack, director of the Center for Energy Law and Policy at Penn State University. “We have social cost of carbon used in regulatory decisions but not (a carbon price) that is faced by the market.”
