# Supplementary material for: Public interest in biodiversity and climate change: A comparative culturomics study of China and the UK
Source: PLoS One. 2026 Jan 14;21(1):e0338006. doi: 10.1371/journal.pone.0338006 (PMC12803463; doi:10.1371/journal.pone.0338006)
Supplement: Attachment — Submitted filename: PONE-D-25-31967_reviwer.pdf [file pone.0338006.s005.pdf]

PLOS ONE

Public interest in biodiversity and climate change: A comparative culturomics study of China and the UK

--Manuscript Draft--

**Manuscript Number:**	PONE-D-25-31967
**Article Type:**	Research Article
**Full Title:**	Public interest in biodiversity and climate change: A comparative culturomics study of China and the UK
**Short Title:**	Public interest in biodiversity and climate change
**Corresponding Author:**	Li Li, Dr. rer. nat.<br>Xi'an Jiaotong-Liverpool University<br>Suzhou , JIANGSU CHINA
**Keywords:**	Conservation culturomics; environmental governance; Baidu index; Google Trends; Public interest
**Abstract:**	Understanding how the public engages with biodiversity loss and climate change is critical for designing effective environmental policies and conservation strategies. This study applies a conservation culturomics approach to compare public interest in biodiversity and climate change across China and the United Kingdom, two major environmental actors with distinct governance models and cultural contexts. Using search volume data from the Baidu Index and Google Trends between 2011 and 2022, we identify the peak periods of search interest in both countries. We then analyse the associated news content during peak and non-peak periods using grounded theory and thematic coding to uncover the dominant drivers of public attention. Our findings reveal a stark contrast between the sources of public engagement. In China, the public interest is predominantly state-driven, with peaks aligned with government-led campaigns and international events. Themes, such as domestic governance and ecological civilisation, are the most significant. In the UK, civil society, scientific discourse, and environmental activism act as the key catalysts in shaping public engagement. These differences reflect greater variations in political structures, media ecosystems, and cultural values. The results highlight the need for context-sensitive communication strategies. By linking digital behaviour with media discourse, this study offers new insights into public environmental engagement, providing actionable guidance for shaping conservation policies in diverse governance settings.
**Order of Authors:**	Ting Tong
	Magdalena Lenda, PhD
	Uri Roll, PhD
	Li Li, PhD
**Additional Information:**	
**Question**	**Response**
**Financial Disclosure**<br><br>Enter a financial disclosure statement that describes the sources of funding for the work included in this submission. Review the submission guidelines for detailed requirements. View published research articles from *PLOS ONE* for specific examples.<br><br>This statement is required for submission	The author(s) received no specific funding for this work.

Powered by Editorial Manager® and ProduXion Manager® from Aries Systems Corporation

and **will appear in the published article** if the submission is accepted. Please make sure it is accurate.<br><br>**Funded studies**<br>Enter a statement with the following details:<br>• Initials of the authors who received each award<br>• Grant numbers awarded to each author<br>• The full name of each funder<br>• URL of each funder website<br>• Did the sponsors or funders play any role in the study design, data collection and analysis, decision to publish, or preparation of the manuscript?<br><br>Did you receive funding for this work?	
**Competing Interests**<br><br>Use the instructions below to enter a competing interest statement for this submission. On behalf of all authors, disclose any competing interests that could be perceived to bias this work—acknowledging all financial support and any other relevant financial or non-financial competing interests.<br><br>This statement is required for submission and **will appear in the published article** if the submission is accepted. Please make sure it is accurate and that any funding sources listed in your Funding Information later in the submission form are also declared in your Financial Disclosure statement.<br><br>View published research articles from *PLOS ONE* for specific examples.	The authors have declared that no competing interests exist.

**NO authors have competing interests**<br><br>Enter: *The authors have declared that no competing interests exist.*<br><br>**Authors with competing interests**<br><br>Enter competing interest details beginning with this statement:<br><br>*I have read the journal's policy and the authors of this manuscript have the following competing interests: [insert competing interests here]*<br><br>* typeset	
**Ethics Statement**<br><br>Enter an ethics statement for this submission. This statement is required if the study involved:<br><br>• Human participants<br>• Human specimens or tissue<br>• Vertebrate animals or cephalopods<br>• Vertebrate embryos or tissues<br>• Field research<br><br>Write "N/A" if the submission does not require an ethics statement.<br><br>General guidance is provided below. Consult the submission guidelines for detailed instructions. **Make sure that all information entered here is included in the Methods section of the manuscript.**	N/A

**Format for specific study types**<br><br>**Human Subject Research (involving human participants and/or tissue)**<br>• Give the name of the institutional review board or ethics committee that approved the study<br>• Include the approval number and/or a statement indicating approval of this research<br>• Indicate the form of consent obtained (written/oral) or the reason that consent was not obtained (e.g. the data were analyzed anonymously)<br><br>**Animal Research (involving vertebrate animals, embryos or tissues)**<br>• Provide the name of the Institutional Animal Care and Use Committee (IACUC) or other relevant ethics board that reviewed the study protocol, and indicate whether they approved this research or granted a formal waiver of ethical approval<br>• Include an approval number if one was obtained<br>• If the study involved *non-human primates*, add *additional details* about animal welfare and steps taken to ameliorate suffering<br>• If anesthesia, euthanasia, or any kind of animal sacrifice is part of the study, include briefly which substances and/or methods were applied<br><br>**Field Research**<br><br>Include the following details if this study involves the collection of plant, animal, or other materials from a natural setting:<br>• Field permit number<br>• Name of the institution or relevant body that granted permission	
**Data Availability**<br><br>Authors are required to make all data underlying the findings described fully available, without restriction, and from the time of publication. PLOS allows rare exceptions to address legal and ethical concerns. See the PLOS Data Policy and FAQ for detailed information.	Yes - all data are fully available without restriction

A Data Availability Statement describing where the data can be found is required at submission. Your answers to this question constitute the Data Availability Statement and **will be published in the article**, if accepted.<br><br>**Important:** Stating 'data available on request from the author' is not sufficient. If your data are only available upon request, select 'No' for the first question and explain your exceptional situation in the text box.<br><br>Do the authors confirm that all data underlying the findings described in their manuscript are fully available without restriction?	
**Describe where the data may be found in full sentences. If you are copying our sample text, replace any instances of XXX with the appropriate details.**<br><br>• If the data are **held or will be held in a public repository**, include URLs, accession numbers or DOIs. If this information will only be available after acceptance, indicate this by ticking the box below. For example: *All XXX files are available from the XXX database (accession number(s) XXX, XXX.).*<br>• If the data are all contained **within the manuscript and/or Supporting Information files**, enter the following: *All relevant data are within the manuscript and its Supporting Information files.*<br>• If neither of these applies but you are able to provide **details of access elsewhere**, with or without limitations, please do so. For example:<br><br>*Data cannot be shared publicly because of [XXX]. Data are available from the XXX Institutional Data Access / Ethics Committee (contact via XXX) for researchers who meet the criteria for access to confidential data.*<br><br>*The data underlying the results presented in the study are available from (include the name of the third party*	All relevant data are within the manuscript and its Supporting Information files.

and contact information or URL).<br>• This text is appropriate if the data are owned by a third party and authors do not have permission to share the data.<br><br>* typeset	
Additional data availability information:	

manuscript Click here to access/download;Manuscript;MS.docx

Title page

Public interest in biodiversity and climate change: A comparative culturomics study of China and the UK

Author: Ting Tong, Magdalena Lenda, Uri Roll, Li Li

Ting Tong:

Department of Health and Environmental Sciences, Xi'an Jiaotong-Liverpool University, Chongwen Road 8, Suzhou, 215123, China

Department of Biological Sciences, National University of Singapore, 14 Science Drive 4 117557, Singapore

tingtong4396@gmail.com

Magdalena Lenda:

Institute of Nature Conservation, Polish Academy of Sciences, 31-120 Kraków, Poland.

magdalena.lenda1@gmail.com

Uri Roll

Sede-Boqer Campus, Ben-Gurion University of the Negev, Midreshet Ben-Gurion 8499000, Israel

uriroll@bgu.ac.il

Li Li:

Department of Health and Environmental Sciences, School of Science, Xi’an Jiaotong-Liverpool University, Chongwen Road 8, Suzhou, 215123, China

Li.Li01@xjtlu.edu.cn

Corresponding Author: Li Li, Ting Tong

Abstract

Understanding how the public engages with biodiversity loss and climate change is critical for designing effective environmental policies and conservation strategies. This study applies a conservation culturomics approach to compare public interest in biodiversity and climate change across China and the United Kingdom, two major environmental actors with distinct governance models and cultural contexts. Using search volume data from the Baidu Index and Google Trends between 2011 and 2022, we identify the peak periods of search interest in both countries. We then analyse the associated news content during peak and non-peak periods using grounded theory and thematic coding to uncover the dominant drivers of public attention. Our findings reveal a stark contrast between the sources of public engagement. In China, the public interest is predominantly state-driven, with peaks aligned with government-led campaigns and international events. Themes, such as domestic governance and ecological civilisation, are the most significant. In the UK, civil society, scientific discourse, and environmental activism act as the key

catalysts in shaping public engagement. These differences reflect greater variations in political structures, media ecosystems, and cultural values. The results highlight the need for context-sensitive communication strategies. By linking digital behaviour with media discourse, this study offers new insights into public environmental engagement, providing actionable guidance for shaping conservation policies in diverse governance settings.

Key words: Conservation culturomics, Environmental governance, Baidu index, Google Trends, Public interest

Introduction

Loss of biodiversity and climate change are among the most urgent environmental challenges confronting human societies. While policies and strategies to address these issues are often developed through top-down approaches, their long-term success depends heavily on public awareness, support, and behavioural change (Lu et al., 2018; Stroik et al., 2019). Therefore, understanding the drivers of public interest in environmental issues is critical for effective conservation and governance. Digital platforms, particularly Internet search engines and social media, offer novel insights into how the public engages with environmental topics. The emerging field of conservation culturomics leverages digital data to examine public sentiment, interest, and cultural trends related to conservation (Ladle et al., 2016; Troumbis, 2019;

Correia et al., 2021). Compared to traditional survey methods, culturomics provides broader and less biased access to public behaviour, avoiding issues like experimenter influence and social desirability (Jarić et al., 2020). Despite its potential, cross-national applications of this method remain limited, particularly in non-English-speaking contexts, such as China.

China plays a critical role in the global environmental governance and biodiversity conservation (Liu and Diamond, 2005; Zhang et al., 2017; Dong, 2020). As one of the world's largest economies, China's environmental policies have significantly impacted nature conservation and the success of global environmental actions (Liu and Diamond, 2005). In recent years, the Chinese government has demonstrated an increasing commitment to environmental protection, characterised by ambitious policies aimed at reducing pollution, enhancing energy efficiency, and mitigating the impacts of climate change (Dong, 2020; Zhang et al., 2017). China’s vision of “ecological civilization”, incorporated into its 2018 constitutional amendment, indicates the government’s approach to managing environmental challenges associated with rapid economic growth (Lu et al., 2020). This also indicates China’s intention to assume the role of a global leader in environmental governance (Percival, 2010). For example, China has enforced several stringent regulations to curb industrial pollution and invested heavily in renewable energy, aiming to shift away from its dependence on coal (Liu et al., 2021). Furthermore, China's ban on foreign waste imports, restrictions on plastic use, and prohibition of the ivory

trade underline its resolution to address environmental issues on multiple fronts (Qu et al., 2019; Wang and Li, 2021; Zhou et al., 2018). While challenging to implement, these policies signal China's evolving approach to environmental stewardship in the 21st century.

However, the international understanding of environmental engagement within Chinese society remains limited, partly due to language barriers and limited access to Chinese-language discourse (Amano et al., 2021; Seng and Lai, 2010). Despite their importance in shaping the effectiveness of government policies, public attitudes and behavioural drivers in China are understudied (Zheng and Shi, 2017; Zhang et al., 2022).

In contrast, the United Kingdom provides a different model of environmental governance, characterised by stronger civil society participation, open media discourse, and individual-driven activism. A comparison of these two countries—both key actors in the global environmental agenda—offers a unique opportunity to examine how differences in governance, media systems, and cultural values influence public environmental concern (Wurzel et al., 2020; Adler and Aycan, 2018; Tynan et al., 2010).

Our study explores public interest in biodiversity and climate change in China and the UK through the lens of conservation culturomics. We use search volume data from the Baidu Index and Google Trends between 2011 and 2022 to identify periods of heightened public attention and analyse the news content associated with these peaks. Our approach captures the intersection of societal

interest, digital behaviour, and media framing in two contrasting governance systems. We address the following three research questions:

1) What are the periods of peak public search interest in biodiversity and climate change in China and the UK from 2011 to 2022?

2) What topics in digital news coincide with these peaks? How do these patterns reflect broader socio-political and cultural drivers of environmental concern?

By linking public attention to digital media content, we provide new insights into how environmental narratives are constructed and received in different socio-political contexts. Our findings contribute to the growing body of literature on digital conservation methods, highlighting the importance of cross-cultural perspectives in shaping environmental policies and public engagement.

Methods

Study Design

Search engine data are an important source of information in conservation culturomics. Given the vast number of users of web search engines, such as Google and Baidu, these platforms can provide ample data to analyse public interest in environmental issues (Troumbis, 2021; Vaughan and Chen, 2015). English and Chinese are the two most widely used languages on the internet, with 952 million and 763 million users, respectively, as of 2017, and this number continues to grow (Petrosyan, 2023). For English-speaking users, Google is the

primary search engine, holding a dominant market share in the United Kingdom and the United States (Vaughan and Chen, 2015). Google Trends, a tool developed to estimate search volumes, has gained popularity in conservation studies in Europe and North America (Lenda et al., 2020; Troumbis, 2017). However, Google Trends does not include data from Mainland China because Google transferred its Chinese search service to Hong Kong in March 2010 (Vaughan and Chen, 2015). Mainland China has more than one billion Internet users (Zhou et al., 2022) and Baidu has a search engine market share of over 80%. Therefore, the Baidu Index is the best substitute for Google Trends when studying public interest in China (Lu et al., 2018).

Search Volume Peak and Temporal Patterns Detection

Search query data reflecting Chinese and British public interest was conducted using, respectively, the Baidu Index at http://index.baidu.com/ and Google Trends (limiting the location to the UK) at www.google.com/trends/. The Baidu Index was first introduced in 2011, implying that the earliest available Baidu search volume data came from 2011 onwards (Li et al., 2021). Furthermore, utilising a Chinese search engine eliminated the potential influence of spelling differences across languages (Janc, 2015). For instance, in English, the terms *biodiversity*, *bio-diversity*, and *biological diversity* refer to the same concept; however, in Chinese, there is only one word and no synonym for this concept. Therefore, in this study, we selected the keywords "生物多样性" (meaning “biodiversity” in Chinese) and "气候变化" (meaning “climate change” in Chinese) in Baidu Index and set the study period from 1 January 2011 to 1 November 2022. Google Trends offers access to an unfiltered sample of actual search queries submitted to Google. In contrast to the normalised representation of the absolute search volume in the Baidu Index, Google Trends provides relative search volume for a given term. This implies that the search results are adjusted and standardised, based on the specific time and location of a query, thereby providing normalised results of the search interest (Vaughan and Chen, 2015). Meanwhile, differences in the total search volumes can be observed among diverse regions that exhibit equivalent levels of search interest for a given term. Given that our focus was solely on the public interest in the UK, we restricted

the search region to the United Kingdom. To avoid any semantic bias like synonyms, singular or plural versions, misspellings and spelling variations, we used the Google Trends function of "Topics" search (Höpken et al., 2019; Valdez, 2021). For example, in this study, we adopted the term *biodiversity*, encompassing synonymous terms, such as *bio-diversity* and *biological diversity*. Furthermore, we explored the search volume of the term *climate change*, which incorporated related concepts like "climate variability" and other synonymous expressions. Finally, similar to the Baidu Index data in China, we set the search time from 1 January 2011 to 1 November 2022.

After data collection from Baidu Index and Google Trends, we procured four distinct trendlines reflecting the search volume dynamics for both, *Biodiversity* and *Climate change* within China and the UK. For each trendline, we focused solely on the highest and most conspicuous points. Following this approach, using Baidu Index, we identified six search volume peaks for *Biodiversity* and five search volume peaks for *Climate change* (Fig. 1). Using Google Trends, we explored five search volume peaks for *Biodiversity* and four for *Climate change* (Fig. 1). Notably, each peak point identified in Baidu Index corresponded to a weekly timescale, whereas each peak point in Google Trends corresponded to a monthly timescale. This discrepancy is attributed to inherent differences in the smallest timescales available in Baidu Index and Google Trends when the study period is extended to cover a span of 12 years. Additionally, to examine shifts in public interest over time, we analysed the

temporal patterns within each trendline, excluding weeks with search peaks. We divided the non-peak period spanning nearly 12 years into four distinct intervals: 1 January 2011–31 December 2013; 1 January 2014–31 December 2016; 1 January 2017–31 December 2019; and 1 January 2020–1 November 2022. We then performed individual analyses for both, the peak and non-peak search periods to identify and evaluate their specific patterns and trends.

Fig.1. Search volume trendline in Baidu Index: "Biodiversity" trendline (a). "Climate change" trendline (b). Search volume trendline in Google Trends: "Biodiversity" trendline (c). "Climate change" trendline (d).

Qualitative Data Analysis

Digital News Articles Detection

Prior conservation studies suggest that public interest in environmental issues is largely driven by the extensive dissemination of news articles through digital media and online social networks (Papworth et al., 2015). To evaluate the main topics and driving factors of public interest in biodiversity and climate change in China and the UK, we selected digital news as the cut-in point. To retrieve news articles on biodiversity and climate change in the UK, we used Google search engine and selected biodiversity and climate change as keywords (case-insensitive). Using Google's advanced settings, we set the region to the UK and constrained the search to the study period. Following this, we chose the top ten news articles as representative for all time periods, including the search for both,

peak and non-peak periods. The ranking of news articles for each period was based on Google's algorithmically determined "relevance" factor. Similarly, to retrieve news articles related to biodiversity and climate change in China, we utilised the Baidu search engine as the search platform. Due to the discontinuation of Baidu's historical news search function, we resorted to conducting searches on the web page searching using the keywords “生物多样性+新闻” (meaning “biodiversity + news”) and “气候变化+新闻” (meaning “climate change + news”).

News Thematic Analysis in MAXQDA

We conducted a thematic analysis of representative news based on grounded theory (Chapman et al., 2015). First, we standardised the lengths of the news texts. For Chinese news articles, only the first 450-500 words were selected, whereas for English news articles, only the first 250-300 words were used in the analysis. This variation in the number of words stems from inherent differences in language structure and information density between Chinese and English. These selected lengths were deemed sufficient to encompass all relevant information contained in the respective news articles. This step ensured the uniform contribution of news articles in theme analysis results.

An essential step before coding the text data, was to identify the characteristics of the texts and research topics, which facilitated subsequent code development and subcode categorisation (Consoli, 2021). This study

specifically focused on relevant topics within the overarching themes of biodiversity and climate change. To establish the coding framework, we primarily referenced the IPBES 2019 Global Assessment Report and the IPCC 2023 Sixth Assessment Report, using their highlighted keywords and topics to aid in the subsequent code development process. We adopted a grounded theory approach (Khan, 2014) to code the news texts. First, the two most relevant topics (codes) were assigned to each sentence in the news excerpt. After completing the first round of coding, we developed a comprehensive code system containing two levels of code. The primary code reflected the broad theme of news content (e.g., celebrities and opinion leaders), and the subcode depicted a specific topic under this theme (e.g., Greta Thunberg). It is worth mentioning that, in the following text, we will use the term "topic" to refer to the topic of subcodes and "theme" to refer to the topic of primary codes. Using this coding system, we reviewed the first-round coding results and modified them using the new code system.

MAXQDA (1989 – 2024, VERBI Software. Consult. Sozialforschung GmbH, Berlin, Germany) is a software program designed for qualitative and mixed-method data analysis (Belotto, 2018). We used this software for the qualitative content analysis of news texts. We used the Code Matrix Browser function in MAXQDA Visual Tools to illustrate the distribution of codes (topics) in specific documents (study periods). Additionally, we used the Code Relations Browser to visualise the relationships between different themes and determine the

frequency of their co-occurrence within the study period. We further conducted a Similarity Analysis to assess the level of topic occurrence among the different study periods.

Results

Comparison of Public Interest in Biodiversity between China and the UK

Baidu Index and Google Trends, respectively, revealed six peaks and five peaks in the search volume for *Biodiversity* between 1 January 2011 and 1 November 2022 (Fig. 1). During the peak period In China, the theme, *Domestic environmental governance,* occupied a significant portion of the total themes identified during the peak search periods; the abundance of related codes appeared 330 times, far exceeding the count of 73 times in the UK (Table 1a). It is notable that content related to International Day for Biological Diversity was categorised under the *Domestic Environmental Governance* theme in China due to strong government initiatives and extensive media coverage aimed at promoting biodiversity conservation on this occasion. In contrast, this topic was not mentioned in UK news coverage. In the UK, the theme *Nature's contributions to people* (141 counts), *Biodiversity threat* (144 counts), *Stakeholder* (95 counts), *Scientific research and technology* (101 counts), and *Climate change* (72 counts) ranked highest during the search-peak periods. In both, China and the UK, the theme, *International environmental governance,* was prominent during the search-peak periods, appearing 126 and 155 times,

respectively (see Table 1a). Table 2a displays all the topics that appeared under the theme, *International environmental governance,* for biodiversity in China and the UK. *The Convention on Biological Diversity* was the most frequently appearing news topic under the *International environmental governance* theme in both, China and the UK.

Table 1. Comparison of all themes for biodiversity between China and the UK during search-peak periods (a) and non-peak periods (b).

Table 1a

Theme for biodiversity (search-peak)	Abundance of codes in the UK news	Abundance of codes in Chinese news
Domestic environmental governance	73	330
International environmental governance	155	126
Nature’s contributions to people	141	67
Biodiversity threat	144	31
Transformative change	5	3
Stakeholder	95	37
Sustainable Development Goals	59	19
Climate change	72	5
Nexus thinking	18	15
Scientific research and technology	101	45
Urban biodiversity	4	6
Biodiversity hotspot	8	11
Conservation effectiveness	17	37
Biodiversity value	12	34
Biodiversity	2	14
Worldview and values	2	7
Charismatic species	0	10
Science popularization	4	14
Protect urgency	24	0

Table 1b

Theme for biodiversity (non-peak)	Abundance of codes in the UK news	Abundance of codes in Chinese news
Domestic environmental governance	38	169
International environmental governance	145	113
Nature's contributions to people	163	90
Biodiversity threat	123	56
Transformative change	6	12
Stakeholder	25	7
Sustainable Development Goals	23	6
Climate change	23	30
Nexus thinking	44	27
Scientific research and technology	93	32
Urban biodiversity	8	7
Biodiversity hotspot	14	2
Conservation effectiveness	10	23
Biodiversity value	26	48
Biodiversity	7	25
Worldview and values	7	24

Table 2. Topic comparison within the theme of International environmental governance in China and the UK during search-peak period for (a) biodiversity and (b) climate change. (IUCN: International Union for Conservation of Nature, WWF: World Wide Fund for Nature, TNFD: Nature-related Financial Disclosures, UNFCCC: United Nations Framework Convention on Climate Change.)

Table 2a

Topic for International environmental governance for biodiversity	Abundance of codes in the UK news	Abundance of codes in Chinese news
Convention on Biological Diversity	41	94
Conservation measures	33	0
International environmental policy	1	0
International cooperation	10	21
United Nations	14	11
WWF	1	0
IPBES	1	0

European Union	11	0
World Day to Combat Desertification and Drought	1	0
Visionary Perspective Plan	2	0
World Environment Day	10	0
World Database on Protected Areas	1	0
National Ecosystem Assessments	8	0
TNFD	1	0
International Biodiversity Day	3	0
Financial support	16	0

Table 2b

**Topic for International environmental governance for Climate change**	**Abundance of codes in the UK news**	**Abundance of codes in Chinese news**
UNFCCC	76	92
UN	4	4
IPCC	22	2
International cooperation	3	20
Donor-advised fund	0	13
WWF	1	0
Climate action tracker	7	0
Financial support	4	0
Earth day	18	0
Carbon market	12	0

In non-peak periods, the theme arousing the most public interest in China and the UK were, respectively, *Domestic environmental governance* and *Biodiversity threat*, *Science research and technology*. In both, China and the UK, the themes, *International environmental governance* and *Nature's contributions to people,* were common (Table 1b). Although the peaks for the UK-related data demonstrated greater dispersion and were divided into three distinct clusters, the six peaks of interest in biodiversity from China were significantly similar and could be classified into a single cluster. (Fig. 2a). For China, the top five topics with the highest distribution within *Domestic environmental governance* were *International Day for Biological Diversity* (21.8%), *Species conservation* (17.3%), *Ecological civilization* (10.9%), *China environmental leadership* (9.1%), and *Habitat conservation* (9.1%) (Fig. 3a). Furthermore, within the *Stakeholder* theme, during the search-peak period in

the UK, the *Private sector* and *Civil society* appeared 50 and 45 times, respectively, whereas in China, only *Civil society* appeared 37 times.

Fig.2. Illustration of multidimensional cluster analysis of news theme of (a) public interest in biodiversity similarity in China (CN) and the UK during search-peak periods, and (b) co-occurrences of themes for the climate change search-peak periods in the UK, showing a strong association between the topic, Climate change protest and Opinion leaders. (Opinion leaders include Joey Barton (4), Greta Thunberg (62), Emma Thompson (12), The Simpsons (10) and Trump (3); Climate change protest includes Climate Strike (20) and Extinction Rebellion (111).)

Fig. 3. Topics in Chinese biodiversity domestic environmental governance during peak period (a). Topics in China's climate change domestic environmental governance (b). Conspiracy theory theme of climate change in non-peak periods in the UK (c).

Comparison of Public Interest in Climate Change between China and UK

Between 1 January 2011 and 1 November 2022, we identified five peaks in the search volume for *Climate change* using Baidu Index in China and four peaks using Google Trends in the UK (Fig. 1). When we explored the interest in climate change search volume, both China and the UK exhibited a substantial occurrence of *International environmental governance* and *Climate change impact*, with China recording counts of 131 and 220 times, respectively, and the UK recording counts of 147 and 133, respectively. In both countries, *United Nations Framework Convention on Climate Change* (UNFCCC) was the most frequently coded news topic during peak search periods (Table 2b). Additionally, in China, the theme, *Domestic environmental governance,* was the most popular (123 counts), followed by *Climate change threat and causes* (106 counts) and a high diversity of topics (17 topics). In the UK, *Celebrities and*

opinion leaders (91 counts) and *Climate change protest* (131 counts) were the most popular, with relatively low topic diversity (14 topics) (Table 3a). During non-peak periods, *Climate change impacts* (333 counts) constituted the most important news topic in China, followed by *Domestic environmental governance* (89 counts). In the UK, the themes with the highest frequencies were *International environmental governance* (167 counts) and *Climate change impacts* (160 counts) (Table 3b).

Table 3. Comparison of all themes for climate change between China and UK during search-peak periods (a) and non-peak periods (b).

Table 3a

Theme for climate change (search-peak)	Abundance of codes in the UK news	Abundance of codes in Chinese news
Domestic environmental governance	11	123
International environmental governance	147	131
Climate change impacts	133	210
Biodiversity	45	47
Climate threat and reason	23	106
Mitigation and Adaptation	77	78
Approach effectiveness	48	13
Sustainable Development Goals	0	14
Stakeholder	54	11
Environmental injustice	0	19
Nexus thinking	0	26
Conspiracy theory	1	9
Transformative change	5	6
Scientific research and technology	29	32
Worldview and values	0	13
Celebrities and opinion leaders	91	4
Political agenda	0	12

Climate change protest	131	0
Brexit	9	0

Table 3b

Theme for climate change (search-peak)	Abundance of codes in the UK news	Abundance of codes in Chinese news
Domestic environmental governance	23	89
International environmental governance	167	49
Climate change impacts	160	333
Biodiversity	34	16
Climate threat and reason	39	23
Mitigation and Adaptation	85	62
Approach effectiveness	27	3
Sustainable Development Goals	1	1
Stakeholder	20	9
Environmental injustice	16	5
Nexus thinking	2	4
Conspiracy theory	43	6
Transformative change	11	2
Scientific research and technology	75	31
Worldview and values	1	4
Celebrities and opinion leaders	43	0
Political agenda	6	0
Concept of climate change	0	8
Climate change protest	24	6
Financial crisis	2	0
COVID	4	0

During the search peak periods of climate change in China, the topic, *China environmental leadership* and *Beijing Winter Olympics* had the highest counts, accounting for 30.9% and 28.5% of the total, respectively (Fig. 4). In the UK, there was a high co-occurrence between the theme, *Celebrities and opinion leaders* and *Climate change protest* (Fig. 2b), and the topics with the highest

occurrence under these two themes were *Greta Thunberg* (62 counts) and *Extinction Rebellion* (111 counts). Notably, the theme, *Conspiracy theory,* in the UK's climate change news rapidly declined from 36 counts during the period 2011–2013 to only one count during the period 2020–2022 (Fig. 3c).

Discussion

Our findings demonstrate distinct patterns of how public interest in biodiversity and climate change is shaped in China and the UK, reflecting broader differences in governance structures, cultural values, and media dynamics. In China, the public environmental interest is closely aligned with state-driven narratives and top-down communication strategies. By contrast, public concern in the UK is more strongly associated with civil society, scientific discourse, and opinion leaders. These contrasts offer valuable insights into how conservation communication and policies can be tailored across socio-political contexts.

Chinese public interest aligns with state-led environmental narratives

In China, both, peak and non-peak search periods for biodiversity and climate change show a consistent dominance of the domestic environmental governance theme. This pattern reflects the central role of government-led initiatives in shaping public discourse and awareness (Dong, 2020; Lu et al., 2020). News topics, such as the International Day for Biological Diversity, Ecological Civilisation, and China's Environmental Leadership, were especially

significant during the search peak periods (Fig. 3a), suggesting that the state's communication efforts have been effective in amplifying interest at strategic moments. This alignment is particularly evident during peaks that coincide with official events, such as the annual International Day for Biological Diversity and the 15th Conference of the Parties (COP15) to the Convention on Biological Diversity (CBD), held in Kunming. These findings support earlier arguments that the Chinese government plays a key role in framing narratives around biodiversity (Liu et al., 2003; Zou et al., 2017). The consistent reinforcement of biodiversity-related values across administrative levels, as observed in both national and local news sources, indicates a coordinated strategy for cultivating public awareness. Similarly, for climate change, Chinese public interest has been driven by themes, such as green development, the Belt and Road Initiative, and China's environmental leadership, further reinforcing the state's role in shaping public perceptions (Zou et al., 2017). Notably, climate change impacts emerged as the most frequently coded theme during non-peak periods, indicating that Chinese citizens were becoming increasingly aware of the tangible effects of climate change (Yu et al., 2013). However, the limited diversity of non-governmental topics, such as civil society, the private sector, and activism, suggests a relatively narrow discursive space for environmental engagement. While collectivist cultural values rooted in Confucianism may encourage deference to authority and national goals (Winfield et al., 2000; Wang, 2023), effective environmental governance requires broader public

participation and dialogue (IPBES, 2019). Enhancing bottom-up engagement and diversifying environmental narratives could increase the public ownership of conservation efforts in China.

Public interest in the UK driven by civil society and scientific discourse

By contrast, public environmental interest in the UK displayed greater thematic diversity and was more strongly influenced by civil society, scientific discourse, and activism. For biodiversity, themes, such as biodiversity threats, nature's contribution to people, and scientific research and technology, were dominant during both the peak and non-peak periods. The presence of private sector and stakeholder-related news also indicates a more distributed model of environmental governance (Rugman and Verbeke, 1998; Kelemen, 2010). Unlike China, where biodiversity and climate change interests are triggered by official events, the interest in climate change in the UK often coincides with activism, media coverage, and opinion leaders. The high frequency of terms, such as Greta Thunberg and Extinction Rebellion, during search-peak periods reflects the power of grassroots movements and individuals in shaping public discourse (Salerno, 2023). These findings align with broader cultural differences; individualistic societies, such as the UK, tend to emphasise personal agency and civic activism (Adler and Aycan, 2018; Veenhoven, 1999). Another key observation is the sharp decline in conspiracy theories in the UK over time, dropping from 36 mentions during 2011–2013 to just one during

2020–2022 (Fig. 3c). This trend may indicate a decline in climate scepticism and an increase in public trust in scientific consensus, which is an encouraging sign of climate policy (Lewandowsky, 2014). Interestingly, the UK news coverage also displayed greater thematic integration, with biodiversity often discussed in the context of climate change. This may suggest that environmental awareness in the UK is more systemic and interconnected, rather than compartmentalised —potentially mitigating the so-called "bandwidth limit effect" (Burivalova et al., 2018; Casola et al., 2022; Barbier et al., 2020).

Governance, culture, and culturomics in nature conservation

Although China's top-down communication approach effectively mobilises attention around strategic policy moments, it may limit the space for diverse societal voices. Conversely, the UK’s pluralistic media and activist-driven discourse foster broader engagement but may result in fragmented attention or politicization. The value of conservation culturomics lies in its ability to detect these differences across contexts, using large-scale real-time data. By combining search engine trends with media content analysis, we offer a perspective complementary to traditional survey-based approaches.

Study limitations

Our study and method are not without limitations. Differences in platform algorithms, censorship, and language use must be considered when

interpreting cross-country patterns (Vaughan and Chen, 2015; Amano et al., 2021). Baidu Index and Google Trends provide relative rather than absolute search volumes and their proprietary algorithms may affect data comparability between platforms (Vaughan and Chen, 2015). Second, while search behaviour is a useful proxy for public interest, it does not necessarily reflect deeper concern or action and may be influenced by external factors, such as media hype or algorithmic suggestions (Burivalova et al., 2018). Third, our analysis of digital news was limited to top-ranked articles during the selected time periods, which may not fully capture the diversity of media narratives, particularly in China, where access to historical news content is restricted. Additionally, demographic and regional differences within each country could not be assessed because of the aggregate nature of the data. Finally, broader political and cultural dynamics, including media censorship or polarisation, may influence both search behaviour and news framing, and should be considered when interpreting cross-national patterns. Future studies should integrate additional data sources to capture a more comprehensive perspective on public environmental engagement.

Conclusion

This study highlights the importance of contextualising conservation communication strategies. In China, state-driven messaging provides a strong foundation for mobilising public interest. However, expanding civil society

engagement and fostering bottom-up participation may enhance the sustainability of environmental governance. In the UK, leveraging public trust in scientific authority and activist voices can continue to strengthen civic engagement, although attention must be paid to maintaining coherence and inclusivity in environmental narratives. Our study contributes to the growing field of conservation culturomics by demonstrating its applicability to cross-cultural research and its potential to inform policy design. It also highlights the value of integrating digital behavioural data with media content analysis to better understand how public interest in environmental issues evolves over time. Tailoring environmental policies and communication to specific cultural and political contexts is essential to foster meaningful public engagement and, ultimately, build more effective and inclusive responses to global environmental challenges.

References

Adler, N.J., Aycan, Z., 2018. Cross-cultural interaction: What we know and what we need to know. Annu. Rev. Organ. Psychol. Organ. Behav. 5, 307–333.

Amano, T., Berdejo-Espinola, V., Christie, A.P., Willott, K., Akasaka, M., Báldi, A., Berthinussen, A., Bertolino, S., Bladon, A.J., Chen, M., Choi, C.-Y., Bou Dagher Kharrat, M., De Oliveira, L.G., Farhat, P., Golivets, M., Hidalgo Aranzamendi, N., Jantke, K., Kajzer-Bonk, J., Kemahlı Aytekin, M.Ç., Khorozyan, I., Kito, K., Konno, K., Lin, D.-L., Littlewood, N., Liu, Yang, Liu, Yifan, Loretto, M.-C., Marconi, V., Martin, P.A., Morgan, W.H., Narváez-Gómez, J.P., Negret, P.J., Nourani, E., Ochoa Quintero, J.M., Ockendon, N., Oh, R.R.Y., Petrovan, S.O., Piovezan-Borges, A.C., Pollet, I.L., Ramos, D.L., Reboredo Segovia, A.L., Rivera-Villanueva, A.N., Rocha, R., Rouyer, M.-M., Sainsbury, K.A., Schuster, R., Schwab, D., Şekercioğlu, Ç.H., Seo, H.-M., Shackelford, G., Shinoda, Y., Smith, R.K., Tao, S., Tsai, M., Tyler, E.H.M., Vajna, F., Valdebenito, J.O., Vozykova, S., Waryszak, P., Zamora-Gutierrez, V., Zenni, R.D., Zhou, W., Sutherland, W.J., 2021. Tapping into non-English-language science for the conservation of global biodiversity. PLOS Biol. 19, e3001296. https://doi.org/10.1371/journal.pbio.3001296

Barbier, E.B., Lozano, R., Rodríguez, C.M., Troëng, S., 2020. Adopt a carbon tax to protect tropical forests. Nature 578, 213–216. https://doi.org/10.1038/d41586-020-00324-w

Belotto, M.J., 2018. Data analysis methods for qualitative research: Managing the challenges of coding, interrater reliability, and thematic analysis, in: The Qualitative Report. pp. 2622–2633.

Burivalova, Z., Butler, R.A., Wilcove, D.S., 2018. Analyzing Google search data to debunk myths about the public's interest in conservation. Front. Ecol. Environ. 16, 509–514. https://doi.org/10.1002/fee.1962

Casola, W.R., Beall, J.M., Nils Peterson, M., Larson, L.R., Brent Jackson, S., Stevenson, K.T., 2022. Political polarization of conservation issues in the era of COVID-19: An examination of partisan perspectives and priorities in the United States. J. Nat. Conserv. 67, 126176. https://doi.org/10.1016/j.jnc.2022.126176

Chapman, A., Hadfield, M., Chapman, C., 2015. Qualitative research in healthcare: An introduction to grounded theory using thematic analysis. Journal of the Royal College of Physicians of Edinburgh 45, 201–205. https://doi.org/10.4997/jrcpe.2015.305

Consoli, S., 2021. Uncovering the hidden face of narrative analysis: A reflexive perspective through MAXQDA. System 102, 102611. https://doi.org/10.1016/j.system.2021.102611

Correia, R.A., Ladle, R., Jarić, I., Malhado, A.C.M., Mittermeier, J.C., Roll, U., Soriano-Redondo, A., Veríssimo, D., Fink, C., Hausmann, A., Guedes-Santos, J., Vardi, R., Di Minin, E., 2021. Digital data sources and methods for conservation culturomics. Conserv. Biol. 35, 398–411. https://doi.org/10.1111/cobi.13706

Dong, L., 2020. China's renewed perception of global environmental governance. Chin. J. Popul. Resour. Environ. 18, 319–323. https://doi.org/10.1016/j.cjpre.2021.04.003

Höpken, W., Eberle, T., Fuchs, M., Lexhagen, M., 2019. Google Trends data for analysing tourists' online search behaviour and improving demand forecasting: the case of Åre, Sweden. Inf. Technol. Tour. 21, 45–62. https://doi.org/10.1007/s40558-018-0129-4

IPBES, 2019. Summary for policymakers of the global assessment report on biodiversity and ecosystem services of the Intergovernmental Science-Policy Platform on Biodiversity and Ecosystem Services. In: Díaz, S., Settele, J., Brondízio, E.S., Ngo, H.T., Guèze, M., Agard, J., Arneth, A., Balvanera, P., Brauman, K.A., Butchart, S.H.M., Chan, K.M.A., Garibaldi, L.A., Ichii, K., Liu, J., Subramanian, S.M., Midgley, G.F., Miloslavich, P., Molnár, Z., Obura, D., Pfaff, A., Polasky, S., Purvis, A., Razzaque, J., Reyers, B., Roy Chowdhury, R., Shin, Y.J., Visseren-Hamakers, I.J., Willis, K.J. and Zayas, C.N. (Eds.), IPBES secretariat, Bonn, Germany, pp. 1–56.

Janc, K., 2015. Visibility and Connections among Cities in Digital Space. J. Urban Technol. 22, 3–21. https://doi.org/10.1080/10630732.2015.1073899

Jarić, I., Roll, U., Arlinghaus, R., Belmaker, J., Chen, Y., China, V., Douda, K., Essl, F., Jähnig, S.C., Jeschke, J.M., Kalinkat, G., Kalous, L., Ladle, R., Lennox, R.J., Rosa, R., Sbragaglia, V., Sherren, K., Šmejkal, M., Soriano-Redondo, A., Souza, A.T., Wolter, C., Correia, R.A., 2020. Expanding conservation culturomics and iEcology from terrestrial to aquatic realms. PLOS Biol. 18, e3000935. https://doi.org/10.1371/journal.pbio.3000935

Kelemen, R.D., 2010. Globalizing European Union environmental policy. J. Eur. Public Policy 17, 335–349. https://doi.org/10.1080/13501761003662065

Khan, S.N., 2014. Qualitative research method: Grounded theory. Int. J. Bus. Manag. 9, 224–233.

Ladle, R.J., Correia, R.A., Do, Y., Joo, G.-J., Malhado, A.C., Proulx, R., Roberge, J.-M., Jepson, P., 2016. Conservation culturomics. Front Ecol Environ 14, 269–275. https://doi.org/10.1002/fee.1260

Lenda, M., Skórka, P., Mazur, B., Sutherland, W., Tryjanowski, P., Moroń, D., Meijaard, E., Possingham, H.P., Wilson, K.A., 2020. Effects of amusing memes on concern for unappealing species. Conserv. Biol. 34, 1200–1209. https://doi.org/10.1111/cobi.13523

Lewandowsky, S., 2014. Conspiratory fascination versus public interest: the case of 'climategate.' Environ. Res. Lett. 9, 111004. https://doi.org/10.1088/1748-9326/9/11/111004

Li, W., Yang, G., Li, X., 2021. Correlation between PM2.5 pollution and its public concern in China: Evidence from Baidu Index. J. Clean. Prod. 293, 126091. https://doi.org/10.1016/j.jclepro.2021.126091

Liu, J., Diamond, J., 2005. China's environment in a globalizing world. Nature 435, 1179–1186. https://doi.org/10.1038/4351179a

Liu, J., Ouyang, Z., Pimm, S.L., Raven, P.H., Wang, X., Miao, H., Han, N., 2003.

Protecting China’s Biodiversity. Science 300, 1240–1241. https://doi.org/10.1126/science.1078868

Liu, Z., Deng, Z., He, G., Wang, H., Zhang, X., Lin, J., Qi, Y., Liang, X., 2021. Challenges and opportunities for carbon neutrality in China. Nat. Rev. Earth Environ. 3, 141–155. https://doi.org/10.1038/s43017-021-00244-x

Lu, X., Zhang, S., Xing, J., Wang, Y., Chen, W., Ding, D., Wu, Y., Wang, S., Duan, L., Hao, J., 2020. Progress of Air Pollution Control in China and Its Challenges and Opportunities in the Ecological Civilization Era. Engineering 6, 1423–1431. https://doi.org/10.1016/j.eng.2020.03.014

Lu, Y., Wang, Y., Zuo, J., Jiang, H., Huang, D., Rameezdeen, R., 2018. Characteristics of public concern on haze in China and its relationship with air quality in urban areas. Sci. Total Environ. 637–638, 1597–1606. https://doi.org/10.1016/j.scitotenv.2018.04.382

Papworth, S.K., Nghiem, T.P.L., Chimalakonda, D., Posa, M.R.C., Wijedasa, L.S., Bickford, D., Carrasco, L.R., 2015. Quantifying the role of online news in linking conservation research to Facebook and Twitter: Online News and Conservation. Conserv. Biol. 29, 825–833. https://doi.org/10.1111/cobi.12455

Percival, R.V., 2010. China’s Green Leap Forward toward Global Environmental Leadership. Vt J Envtl L 12, 633.

Petrosyan, A., 2023. Most common languages used on the internet 2017, by users. Statista. URL https://www.statista.com/statistics/262946/most-common-languages-on-the-internet/ (accessed 3.22.25).

Qu, S., Guo, Y., Ma, Z., Chen, W.-Q., Liu, J., Liu, G., Wang, Y., Xu, M., 2019. Implications of China’s foreign waste ban on the global circular economy. Resour. Conserv. Recycl. 144, 252–255. https://doi.org/10.1016/j.resconrec.2019.01.004

Rugman, A.M., Verbeke, A., 1998. Corporate Strategy and International Environmental Policy. J. Int. Bus. Stud. 29, 819–833. https://doi.org/10.1057/palgrave.jibs.8490053

Salerno, F., 2023. The Greta Thunberg Effect on Climate Equity: A Worldwide Google Trend Analysis. Sustainability 15, 6233. https://doi.org/10.3390/su15076233

Seng, G.Y., Lai, L.S., 2010. Global mandarin. Glob. Lang. Cult. Asia Impact Glob. Process. Lang. 14–33.

Stroik, P., Chakraborty, D., Ge, W., Boulter, J., Jamelske, E., 2019. Effect of reciprocity on public opinion of international climate treaties: experimental evidence from the US and China. Clim. Policy 19, 959–973. https://doi.org/10.1080/14693062.2019.1617666

Troumbis, A.Y., 2021. Imbalances in attitudes of European citizens towards biodiversity: Did the communication of the European Biodiversity Strategy work? J. Nat. Conserv. 63, 126041. https://doi.org/10.1016/j.jnc.2021.126041

Troumbis, A.Y., 2019. The time and timing components of conservation culturomics cycles and scenarios of public interest in the Google era. Biodivers. Conserv. 28, 1717–1727. https://doi.org/10.1007/s10531-019-01750-7

Troumbis, A.Y., 2017. Google Trends and cycles of public interest in biodiversity: the

animal spirits effect. Biodivers. Conserv. 26, 3421–3443. https://doi.org/10.1007/s10531-017-1413-x
Tynan, C., Teresa Pereira Heath, M., Ennew, C., Wang, F., Sun, L., 2010. Self-gift giving in China and the UK: Collectivist versus individualist orientations. J. Mark. Manag. 26, 1112–1128. https://doi.org/10.1080/0267257X.2010.508981
Valdez, J.W., 2021. Using Google Trends to Determine Current, Past, and Future Trends in the Reptile Pet Trade. Animals 11, 676. https://doi.org/10.3390/ani11030676
Vaughan, L., Chen, Y., 2015. Data mining from web search queries: A comparison of google trends and baidu index: Data Mining From Web Search Queries: A Comparison of Google Trends and Baidu Index. J. Assoc. Inf. Sci. Technol. 66, 13–22. https://doi.org/10.1002/asi.23201
Veenhoven, R., 1999. Quality-of-life in individualistic society. Soc. Indic. Res. 48, 159–188.
Wang, B., Li, Y., 2021. Plastic bag usage and the policies: A case study of China. Waste Manag. 126, 163–169. https://doi.org/10.1016/j.wasman.2021.03.010
Wang, C., 2023. Resurgence of Confucian education in contemporary China: Parental involvement, moral anxiety, and the pedagogy of memorisation. J. Moral Educ. 52, 325–342.
Winfield, B.H., Mizuno, T., Beaudoin, C.E., 2000. Confucianism, collectivism and constitutions: Press systems in China and Japan. Commun. Law Policy 5, 323–347.
Wurzel, R.K., Liefferink, D., Di Lullo, M., 2020. The European Council, the Council and the Member States: changing environmental leadership dynamics in the European Union, in: The Future of European Union Environmental Politics and Policy. Routledge, pp. 62–84.
Yu, H., Wang, B., Zhang, Y.-J., Wang, S., Wei, Y.-M., 2013. Public perception of climate change in China: results from the questionnaire survey. Nat. Hazards 69, 459–472. https://doi.org/10.1007/s11069-013-0711-1
Zhang, J.Y., Ben Ouagrahm-Gormley, S., Vogel, K., 2022. Creating Common Ground With Chinese Researchers. Issues Sci. Technol.
Zhang, Y.-J., Peng, Y.-L., Ma, C.-Q., Shen, B., 2017. Can environmental innovation facilitate carbon emissions reduction? Evidence from China. Energy Policy 100, 18–28. https://doi.org/10.1016/j.enpol.2016.10.005
Zheng, D., Shi, M., 2017. Multiple environmental policies and pollution haven hypothesis: Evidence from China's polluting industries. Journal of Cleaner Production 141, 295–304. https://doi.org/10.1016/j.jclepro.2016.09.091
Zhou, D., Zhu, J., Guo, Y., 2022. Does internet connect to social justice perception in China? Front. Psychol. 13, 917039.
Zhou, X., Wang, Q., Zhang, W., Jin, Y., Wang, Z., Chai, Z., Zhou, Z., Cui, X., MacMillan, D.C., 2018. Elephant poaching and the ivory trade: The impact of demand reduction and enforcement efforts by China from 2005 – 2017. Glob. Ecol. Conserv. 16, e00486. https://doi.org/10.1016/j.gecco.2018.e00486
Zou, Y., Fu, Y., Yang, L., Wan, X., Wang, Y., Liu, J., Foreign Economic Cooperation Office, Ministry of Environment Protection, Beijing 100035, 2017. China and COP

15: a path for responsible environmental power. Biodivers. Sci. 25, 1169–1175. https://doi.org/10.17520/biods.2017246

Figure 1

Click here to access/download;Figure;Figure1.tif

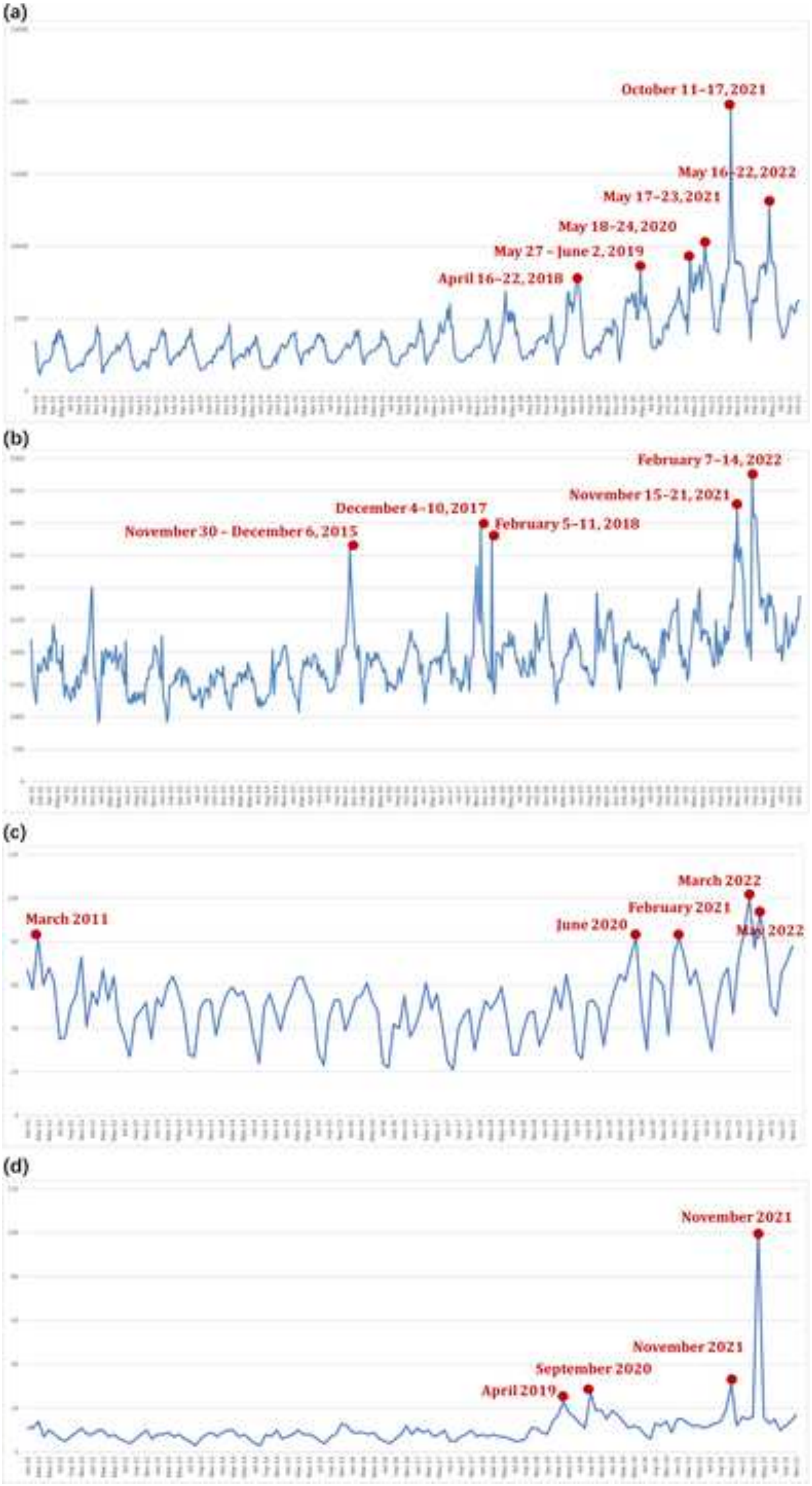

Figure 2

Click here to access/download;Figure;Figure2.tif

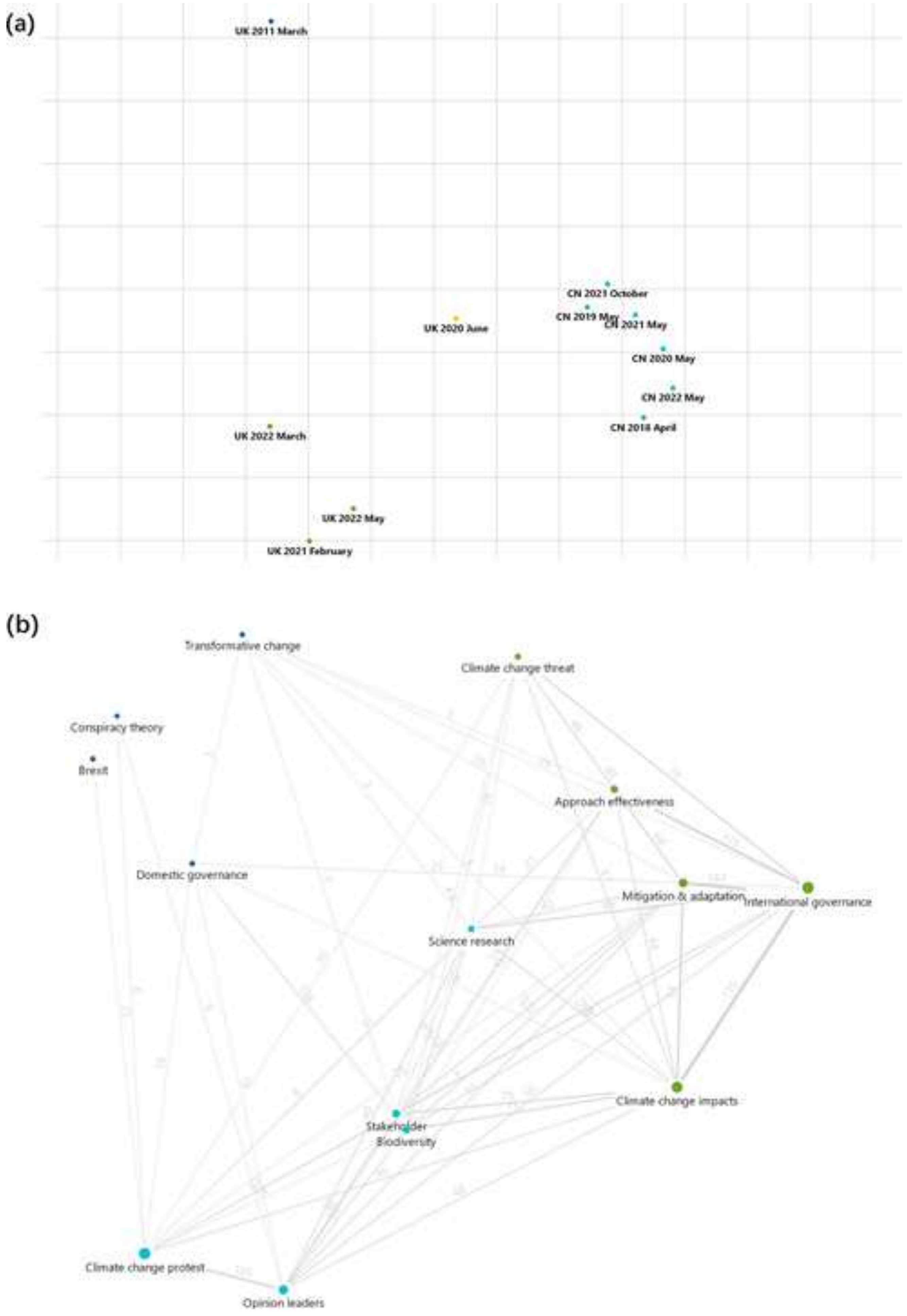

Figure 3

Click here to access/download;Figure;Figure3.tif

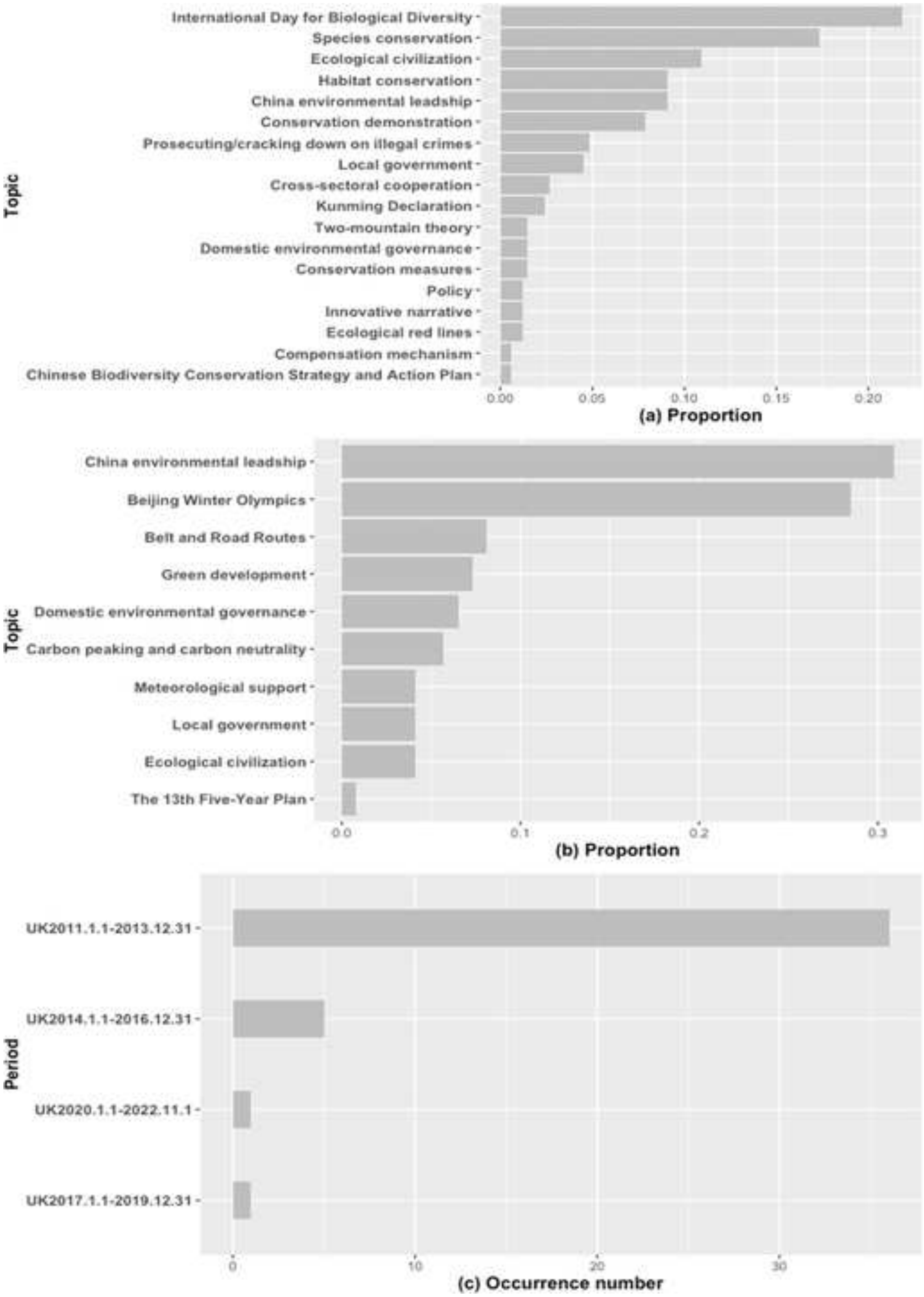

Click here to access/download
Supporting Information
supporting information.docx
