# Supplementary material for: Public interest in biodiversity and climate change: A comparative culturomics study of China and the UK
Source: PLoS One. 2026 Jan 14;21(1):e0338006. doi: 10.1371/journal.pone.0338006 (PMC12803463; doi:10.1371/journal.pone.0338006)
Supplement: Attachment — Submitted filename: Response to Reviewers.docx [file pone.0338006.s007.docx]

Response Letter to Academic Editor, Plos One

Dear Dr. Luisa Maria Diele-Viegas,

Thank you for allowing us to revise our manuscript “Public interest in biodiversity and climate change: A comparative culturomics study of China and the UK”.

We sincerely appreciate the constructive and insightful feedback provided by you and the reviewers. Following the detailed comments, we have carefully revised our manuscript to address every point raised. Specifically, we have made the potential policy impact of our findings and the methodological limitations explicit in the abstract. We ensured consistency in the stated research questions, expanded our discussion on the limitations of traditional methods and clarified how the culturomics approach addresses them. Following suggestions, we defined key concepts such as “public engagement” and “digital behaviour” for consistent use throughout the manuscript, explained the implications of temporal granularity differences between Baidu Index and Google Trends, justified the selection of the top ten news articles and discussed possible algorithmic biases. In the revised text we also clarified the rationale for different excerpt lengths in Chinese and English, with linguistic considerations, and expanded the limitations section to include algorithmic bias, potential censorship/self-censorship, and lack of demographic granularity.

Please find detailed responses below, in section for reviewers.

We hope our revised manuscript meets high scientific standards of the PloS One journal.

Best regards,

On behalf of all authors,

Ting TONG
Department of Health and Environmental Sciences, Xi’an Jiaotong-Liverpool University, Suzhou, China

**Response to Reviewers**

**Review Comments to the Author**: “We recommend making the potential impact of your findings and any methodological limitations more explicit in the final section of the abstract, ensuring consistency in the number of research questions stated, further elaborating on the limitations of traditional methods, and briefly defining key terms such as "public engagement" and "digital behaviour." It would also be beneficial to clarify how certain methodological choices (such as the temporal granularity of data and criteria for news selection) may affect the comparative results.”

**Response**: We would like to sincerely thank the Academic Editor and the two reviewers for their constructive and insightful comments, which have greatly helped us to improve the quality and clarity of our manuscript. In particular, we appreciate the reviewers’ suggestions regarding the need to make the potential impact of our findings and any methodological limitations more explicit in the final section of the abstract, to ensure consistency in the number of research questions stated, to further elaborate on the limitations of traditional methods, and to briefly define key terms such as public engagement and digital behaviour. We also acknowledge the importance of clarifying how certain methodological choices (such as the temporal granularity of data and the criteria for news selection) may affect the comparative results. We have revised the manuscript accordingly to address all of these points in detail. The specific revisions made in response to each comment are listed below. We believe that the revised version satisfactorily responds to all the comments and suggestions raised, and we hope that it now meets the requirements for publication in PLoS ONE.

**Original version Line 49**: “By linking digital behaviour with media discourse, this study offers new insights into public environmental engagement, providing actionable guidance for shaping conservation policies in diverse governance settings.”

**Review comments**: It is recommended to make explicit, in this final part of the abstract, the potential policy impact of the findings and any methodological limitations (e.g., search platform bias), in order to strengthen the manuscript's perceived applicability and robustness.

**Response**: We have specifically added, at the end of the abstract, the potential policy impact for China as well as the limitation regarding platform algorithms.

“Our findings further suggest that enhancing bottom-up participation and diversifying environmental narratives in China could foster greater public ownership of conservation efforts, whereas in the UK maintaining inclusive and coherent narratives is essential. However, limitations such as platform algorithms should be considered when interpreting these cross-country comparisons, as they may affect the comparability of search data between Baidu Index and Google Trends.” (see the new manuscript L46-52).

**Original version Line 71**: “Despite its potential, cross-national applications of this method remain limited, particularly in non-English-speaking contexts, such as China”

**Review comments**: Consider expanding the discussion on limitations of traditional methods and clearly delimiting what the culturomics approach proposes to solve.

**Response:** We have added the limitations of traditional survey methods and explained how the culturomics approach addresses these limitations.

“Compared to traditional survey methods (surveys, interviews, focus groups), which often suffer from limited sample sizes, high costs, temporal and spatial constraints, and biases such as experimenter influence and social desirability [6,7], culturomics uses passively generated digital traces (e.g., search queries, news, social media) to track public attention at broad spatiotemporal scales and in near-real time, reducing experimenter demand and lowering costs [3,6].” (see the new manuscript L71-76).

**Original version Line 113**: “digital behaviour”

**Review comments**: Include succinct definitions for these concepts in the introduction, ensuring that they will be used consistently throughout the text.

**Response:** We have added definitions for the terms ‘digital behaviour’ and ‘public engagement’ in the manuscript.

“Digital behaviour in our study refers to patterns of online activity that reflect public engagement with environmental topics, including actions such as submitting search queries to web search engines, accessing digital news content, and interacting with other online information sources. These activities leave passively generated data traces that can be analysed to infer levels of public interest and the drivers of attention across different temporal and cultural contexts [24].” (see the new manuscript L115-121).

“Therefore, understanding drivers of public interest in environmental issues is critical to foster effective public engagement– which we broadly define here as the active participation of individuals and communities in environmental awareness, decision-making, and action.” (see the new manuscript L62-66).

**Original version Line 114**: “We address the following three research questions:”

**Review comments**: Please review the number of questions listed for clarity (three are referenced, but only two are listed; adjust for consistency).

**Response:** Thank you for pointing out this inconsistency. The third research question was not listed on a separate line and has been corrected (see the new manuscript L126).

**Original version Line 184**: “Notably, each peak point identified in Baidu Index corresponded to a weekly timescale, whereas each peak point in Google Trends corresponded to a monthly timescale.”

**Review comments**: Please detail how this difference in temporal resolution (Baidu weekly, Google monthly) may impact result interpretation and comparability.

**Response:** We have added an explanation of how the difference in temporal granularity between Google Trends (monthly) and Baidu Index (weekly) could affect the search peak curves, and clarified that this has little impact on our results.

“Although the temporal resolution of peak points differed between the two countries, compared with the higher-resolution Baidu Index, the monthly scale of Google Trends may smooth short-term fluctuations but does not alter long-term trends or patterns driven by major events [35]. Subsequent news searches can still accurately identify the corresponding events, and as this study focused on comparing relative changes in public attention, the comparison of cross-platform trends and event response patterns should remain valid despite these difference in temporal resolution.” (see the new manuscript L193-200).

**Original version Line 210**: “Following this, we chose the top ten news articles as representative for all time periods,”

**Review comments**: Please provide a critical justification of the 'top 10' news article selection, highlighting possible algorithmic biases or limitations, especially for Chinese news coverage.

**Response:** We have added a discussion of the potential limitations of search engine algorithms arising from selecting the top 10 news articles, as well as the issue of lacking direct access to historical news searches in Baidu, and have provided reasonable explanations and solutions to address these issues.

“The selection of the top ten news articles was based on a balance between manageable data volume and representativeness, as highly ranked articles typically have greater public visibility and can better reflect the focal issues during a given period [37]. However, search engine ranking algorithms sort results according to proprietary criteria such as click-through rates, recency, and relevance, and often exhibit a bias toward large media outlets, which may lead to the underrepresentation of local or niche reports [38]. Nevertheless, since our primary research objective was to identify the major events driving peaks in public attention, such ranking biases are unlikely to have a substantial impact on our findings. In addition, as Baidu no longer provides a historical news search function, we had to rely on general web searches as a substitute, which may have introduced some variability in the results. Despite these limitations, we applied identical keywords and selection criteria for both peak and non-peak periods, ensuring methodological comparability within the study.” (see the new manuscript L232-245).

**Original version Line 224:** “For Chinese news articles, only the first 450-500 words were selected, whereas for English news articles, only the first 250-300 words were used in the analysis.”

**Review comments:** Please explain in detail the rationale for the differing excerpt lengths and discuss any implications this may have for cross-language comparisons.

**Response:** We corrected a typographical error, noting that the counting unit for Chinese should be character rather than letter. We also added an explanation from a morphemic perspective on why we selected different quantities for Chinese and English, and the importance of this choice in cross-linguistic research.

“We chose a different quantity of characters/ words between Chinese and English as these two languages use different counting units to represent basic information content. An English word is typically composed several letters and may carry one or multiple morphemes, whereas a single Chinese character usually represents one morpheme. Thus, one English word often corresponds to multiple Chinese characters. The ratio typically ranges from about 1.5 to 1.9 Chinese characters per English word [40]. Therefore, in cross-language comparative studies, it is necessary to consider the comparability of text length and employ such a method to align equivalent information content.” (see the new manuscript L253-262).

Original version Line 439: “Although China's top-down communication approach effectively mobilises attention around strategic policy moments, it may limit the space for diverse societal voices.”

Review comments: Expand this limitations discussion: 1) algorithmic bias on both platforms, 2) potential censorship/self-censorship in Chinese news, 3) lack of granular demographic data.

**Response:** We have expanded on each of the above three limitations separately.

“Baidu Index uses a weighted full-data index algorithm with undisclosed calculation details, whereas Google Trends uses a relative normalization plus sampling algorithm, and their proprietary algorithms may affect data comparability between platforms [25]. Methodological choices, including differences in temporal granularity between Baidu Index (weekly) and Google Trends (monthly) and the selection of top-ranked news articles, may influence comparability. These factors can affect the detection of short-lived attention spikes, favour highly visible sources, and, in China, introduce variation due to the lack of a historical news search in Baidu. While these methodological constraints do not invalidate the observed cross-national patterns, they highlight the importance of interpreting the results as comparative indicators of broad trends rather than precise measures of absolute public attention.” (see the new manuscript L488-500).

“In China, news media are typically subject to censorship by national and local propaganda authorities—including the deliberate downplaying of certain topics—resulting in informational gaps when the public searches for content [60]. Additionally, due to concerns about professional safety and public opinion risks, media organizations may proactively avoid reporting on sensitive topics. This self‑censorship tends to choose neutral, safe themes and diminishes issue diversity, potentially leading to underexposure of certain issues and consequent data biases [60,61].” (see the new manuscript L516-523).

“For example, certain regions or demographic groups could exhibit markedly different search patterns, but these are averaged out in nationwide data and thus overlooked; at the same time, it becomes difficult to identify the determinants of such variation—factors like age, gender, education level cannot be disentangled and verified for their influence on search behavior [58,59].” (see the new manuscript L508-513).
